# Utility of Interchangeable Coordination Modes of *N*,*N*′-Dialkyl-2,6-pyridinediamide Tridentate Pincer Ligands for Solvent Extraction of Pd(II) and Zr(IV) from High-Level Radioactive Liquid Waste

**DOI:** 10.1021/acs.inorgchem.4c03844

**Published:** 2024-12-10

**Authors:** Tasuku Orino, Yueming Cao, Ririka Tashiro, Tomoyuki Takeyama, Robert Gericke, Satoru Tsushima, Koichiro Takao

**Affiliations:** † Laboratory for Zero-Carbon Energy, Institute of Integrated Research, Institute of Science Tokyo, 2-12-1 N1-32, O-okayama, Meguro-ku 152-8550, Tokyo, Japan; ‡ Department of Applied Chemistry, Sanyo-Onoda City University, 1-1-1, Daigakudori, Sanyo-Onoda 756-0884, Yamaguchi, Japan; § Institute of Resource Ecology, Helmholtz-Zentrum Dresden-Rossendorf (HZDR), Bautzner Landstrasse 400, Dresden 01328, Germany

## Abstract

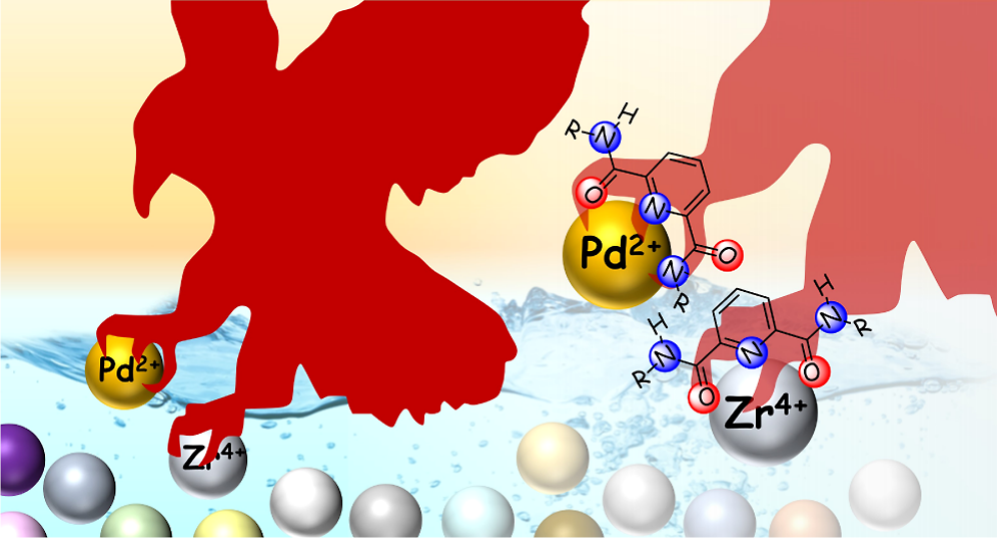

A new class of ligands, *N*,*N*′-dialkyl-2,6-pyridinediamide (DRPDA), has been designed with the specific intention of exhibiting interchangeable diversity in coordination modes, including organometallic interactions, for the purpose of solvent extraction of elements relevant to the proper treatment of high-level radioactive liquid waste (HLLW) generated after nuclear fuel reprocessing. Consequently, DRPDA has been observed to extract Pd(II) and Zr(IV) from HNO_3_(aq) to 1-octanol in nearly quantitative yields when the selected ligand is sufficiently hydrophobic. However, concomitance of some of other HLLW components were also found. The extraction selectivity toward Pd(II) and Zr(IV) was markedly enhanced by employing *n*-dodecane instead of 1-octanol as evidenced by good distribution ratios (*D*_M_) of Pd(II) (*D*_Pd_ = 72.5) and Zr(IV) (*D*_Zr_ = 12.9), which is several orders of magnitude greater than *D*_M_’s of other HLLW components (10^–3^–10^–2^), where addition of 20 vol % 1-octanol is still required to accelerate the extraction kinetics. Despite direct contact with the highly acidic aqueous phase, deprotonation from one of the amide NH moieties of DRPDA proceeds to form [Pd(DRPDA^–^)(NO_3_)] as a good extractables in the current biphasic system. This Pd(II) complex with a rather unique asymmetric N^–^^N^O tridentate coordination was characterized by SCXRD, elemental analysis and ^1^H NMR, and theoretically corroborated by DFT calculations and NBO analysis. In contrast, DRPDA also interacts with Zr^4+^ in different tridentate O^N^O mode without any deprotonation. Based on mechanistic differences in the extraction chemistry we clarified, Pd(II) and Zr(IV) coextracted to the organic phase were recovered stepwise by using appropriate stripping agents such as 1.0 M HCl(aq) and 0.10 M HNO_3_(aq), respectively.

## Abstract 2

Reducing *N*-alkyl substitution of 2,6-pyridinediamide allows diverse coordination manners for harvesting Pd^2+^ and Zr^4+^ from high-level radioactive liquid wastes.

## Introduction

Nuclear power is an overwhelmingly dense energy source that is indispensable for sustaining the current human society. Furthermore, it is garnering increasing attention as a viable power without CO_2_ emission at least during electricity production. Although nuclear fuel recycling is a promising option to gain sustainability of the nuclear energy systems in terms of circular economy,^1^ it inevitably generates radioactive wastes which presents cumbersome problems related to radiotoxicity.^2−4^ Among these challenges, high-level radioactive liquid waste (HLLW) yielded after separation of U and Pu from the spent nuclear fuels represents a particularly complex issue. This waste consists of a range of radionuclides, some of which exhibit exceptionally strong and/or long-lasting radioactivity. The long-lived fission products (LLFPs; ^99^Tc, ^126^Sn, ^79^Se, ^93^Zr, ^135^Cs, ^107^Pd, ^129^I) and minor actinides (MAs; Np, Am, Cm) are of particular interest in chemical separation from HLLW and nuclear transmutation to short-lived or stable nuclides, a process known as partitioning and transmutation (P&T).^5−9^ Moreover, a number of precious metals including Pd, Rh, and Ru are relatively abundant in HLLW. Consequently, HLLW represents a promising alternative source of platinum group metals (PGMs),^10−16^ although issues associated with their radioactivity must be addressed before it can be considered a viable option for practical use.

In this context, various chemical separation principles, most typically solvent extraction, have been studied up to present date.^17−22^ An aqueous phase taken here commonly contains HNO_3_ as a prerequisite simulating HLLW.^2^ NO_3_^–^ is frequently employed as a charge-compensator or salting-out agent, and therefore may enhance extraction of a target metal ion (M) being separated. In contrast, protonation of a selected extractant may occur, which often competes with formation of an extractable complex of M to prohibit chemical separation expected. As a result, extraction chemistry from HNO_3_(aq) is not always straightforward to understand and the design of an optimal system for the desired separation of the HLLW components is still challenging.

**Figure 1:**
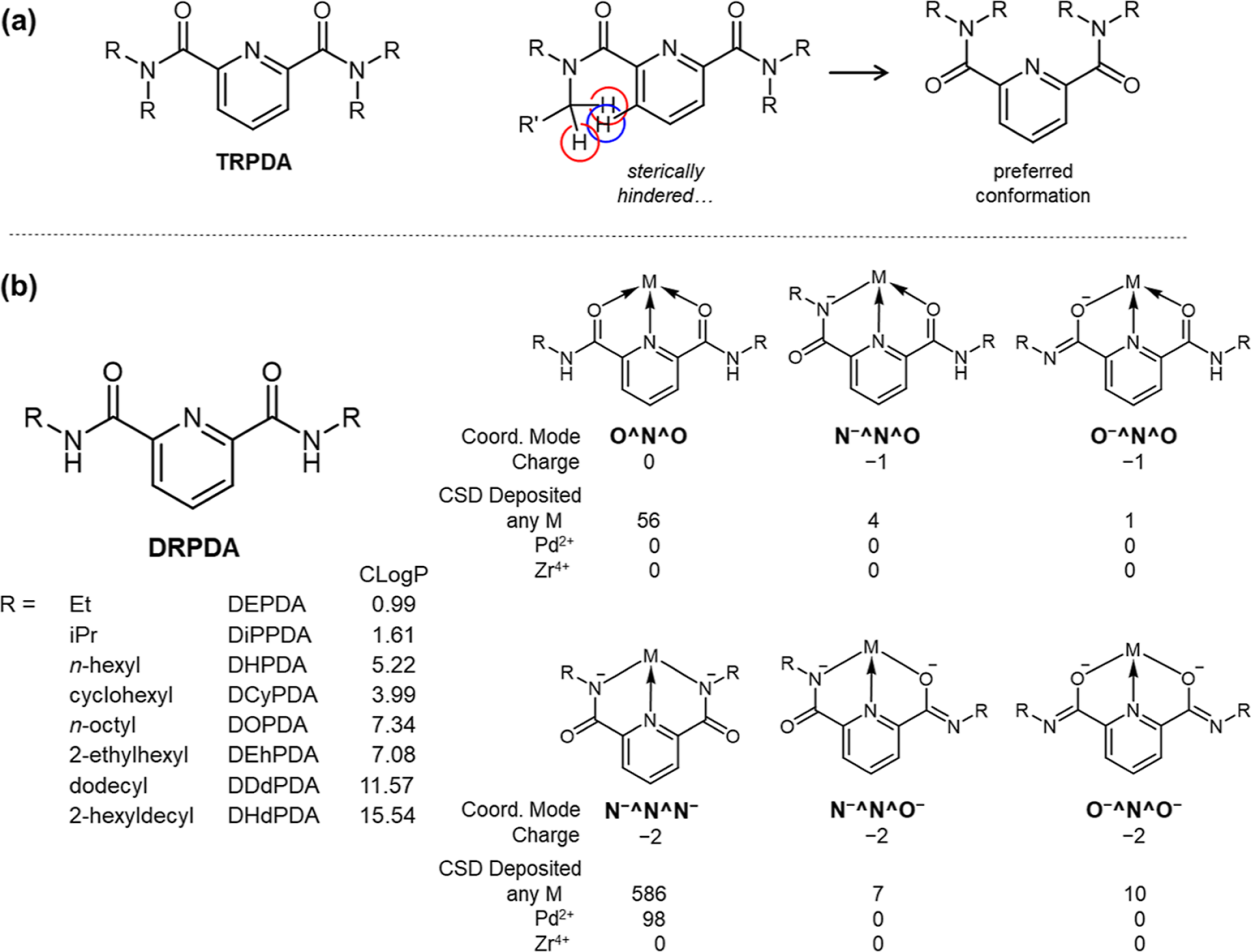
Schematic structures of (a) *N*,*N*,*N*′,*N*′-tetraalkyl-2,6-pyridinediamide (TRPDA) previously employed for solvent extraction of PGMs from HNO_3_(aq) (R = C_*n*_H_2*n*+1_, R′ = C_(*n*–1)_H_2*n*–1_, *n* = 1–4) and (b) DRPDA studied here together with *C* log *P*(32) as a measure of hydrophobicity and its possible coordination modes and the number of reported structures of DPRDA complexes of any M and Pd^2+^ deposited on Cambridge Structure Database (CSD). No structures of Zr^4+^-DRPDA complexes were found in the structure search. Lists of CSD-deposited M-DPRDA complexes in different coordination manners reported to date are available in Supporting Information.

In order to achieve enhanced compatibility with HNO_3_(aq), neutral extractants should be more suitable than anionic ones, given their lower propensity for protonation. For instance, dialkyl sulfides and trialkylated phosphate esters are utilized for this purpose. While S and P in these compounds play a crucial role in the formation of strong complex with M, the presence of these incombustible elements is not favored for pyrolysis for final waste disposal. It is recommended to use organic substances consisting only of incinerable elements such as C, H, O, and N as pronounced by the CHON principle.^12,22,23^ Our group has also dedicated efforts to explore and establish chemical separation principles of various M present in HLLW, especially for PGMs.^24−31^ Through these former works, we have found *N*,*N*,*N*′,*N*′-tetraalkyl-2,6-pyridinediamide (TRPDA, Figure 1a) conforming the CHON principle efficiently extract PGMs from HNO_3_(aq) to 1-octanol, where aid of a hydrophobic counteranion such as (CF_3_SO_2_)_2_N^–^ is required as an phase transfer catalyst.

At the outset of our molecular design of TRPDA, it was anticipated that the compound would engage in a planar tridentate coordination with M through two amide O atoms and one pyridyl N atom. Despite the success of TRPDA in extracting PGMs from HNO_3_(aq) to 1-octanol, the structural details of the extracted species remain uncertain. It seems more reasonable to assume that such a coordination mode suffers from steric hindrance between H atoms of the terminal alkyl chains (R) and those at the 3- and 5-positions of the central pyridyl group. Although the O^N^O coordination of TRPDA was observed in several metal complexes of UO_2_^2+^ and trivalent lanthanides (Ln(III)),^33,34^ this class of ligands still displays a preference for another conformation, as illustrated in Figure 1a^35−37^ unless the expected O^N^O arrangement is sterically controlled.^38^ Therefore, there is still considerable scope for improvement in our ligand design.

To address the aforementioned issue on the ligand structure, we propose an updated concept of *N*,*N*′-dialkylated derivatives of 2,6-pyridinediamides (DRPDA) as shown in Figure 1b. This approach involves the removal of one R group from each amide N atom, which will effectively eliminate the steric hindrance present in TRPDA. In our preliminary structure survey, DRPDA was observed to function as a tridentate planar ligand with the O^N^O manner in coordination compounds of several divalent d-block metals and Ln(III), which aligns with our expectations. However, another tridentate pincer-type coordination, N^–^^N^N^–^, was observed to occur with much greater frequency in reported structures following deprotonation from both amide NH groups of DRPDA as summarized in Figure 1b and Supporting Information.^39^ The doubly deprotonated status of DRPDA^2–^ in the N^–^^N^N^–^ coordination should be thermodynamically disfavored to occur upon contact with an aqueous phase especially under the presence of a strong acid like HNO_3_. If such an organometallic interaction is, however, still available as another soft donating interaction in an organic/aqueous biphasic system, it will open up a new avenue of solvent extraction chemistry. Indeed, solvent extraction of organometallic compounds were examined previously,^40−43^ although there are few applications to HNO_3_-based aqueous systems simulating HLLW treatment. Furthermore, DRPDA may exhibit a variety of interchangeable coordination modes as illustrated in Figure 1b. However, the O^N^O and N^–^^N^N^–^ coordination modes, which were previously discussed, are the most frequently observed in actual cases. It would be beneficial to gain a deeper understanding how DRPDA works for the solvent extraction of M toward the HLLW treatment for P&T.

The objective of this study was to evaluate the efficacy and selectivity of DRPDA in the extraction chemistry of the HLLW components, with a particular focus on the recovery of Pd(II) and Zr(IV) as LLFPs that are pertinent to the backend stream of the nuclear fuel cycle and potential future resource options. Coordination chemistry of these elements with DRPDA was thoroughly investigated to obtain the mechanistic insights into their solvent extraction. Based on this foundation, we have successfully demonstrated the efficient recovery of Pd(II) and Zr(IV) from HNO_3_(aq) with excellent selectivity from other HLLW components.

Adopting novel solvent systems, including ionic liquids (ILs)^22,24,44,45^ and fluorous solvents^19,46,47^ as nonaqueous phases alternative to ordinary organic diluents such as hydrocarbons represents a significant recent trend in the research and development of solvent extraction principles for HLLW. Indeed, there are several successful cases for separation of selected HLLW components, which have demonstrated unique reaction mechanisms that have not been observed in conventional extraction systems.^24^ However, the majority of ILs exhibit higher viscosity and higher mutual solubility with aqueous phase, which is a consequence of their nature as salts. This can complicate the handling of extraction systems and result in a higher water content in the IL phase and its greater loss to the aqueous phase compared to those of organic solvents that are traditionally employed. Fluorinated solvents such as 3-nitrobenzotrifluoride (F-3) and trifluoromethyl phenyl sulfone (FS-13) are not always optimal for use in nuclear engineering especially for their repeated use in chemical separation processes under strong radiation because of radiolytic degradation. Moreover, neither approach align with the CHON principle. While these challenges are of great significance in the exploration of the frontiers of separation chemistry, we have limited our current investigation to conventional organic solvents such as 1-octanol and *n*-dodecane unless otherwise specified. This approach allows us to focus on the impact of the revised molecular design of DPRDA and to enhance the realism and practicality of our solvent extraction systems.

## Results and Discussion

### Solvent Extraction of HLLW Components in 1-Octanol/HNO_3_(aq) Systems

Kinetic aspects of solvent extraction of Pd(II) (5 mM) from 3.0 M HNO_3_(aq) to 1-octanol dissolving DRPDA (30 mM) were first examined. Here, Pd(II) was selected as a representative of the class of LLFPs to be separated from HLLW and subjected to transmutation. As a result, the partitioning of Pd(II) in this biphasic system was completed within 5 min when any DRPDAs bearing either isopropyl or *n*-hexyl side chains on the amide N atoms were used (Figure S1, Supporting Information). The extractabilities (*E*%) with DiPPDA and DHPDA were 87.1 ± 0.2% and 99.8 ± 0.3%, respectively, regardless of any sampling points at different contact time from 5 to 60 min. The difference in *E*% suggests that the hydrophobicity of DRPDA influences the efficiency of Pd(II) extraction in the current system. Based on the observed extraction kinetics, we have decided to shake 1-octanol/HNO_3_(aq) biphasic systems for a minimum of 10 min in subsequent experiments to ensure equilibrium.

**Figure 2:**
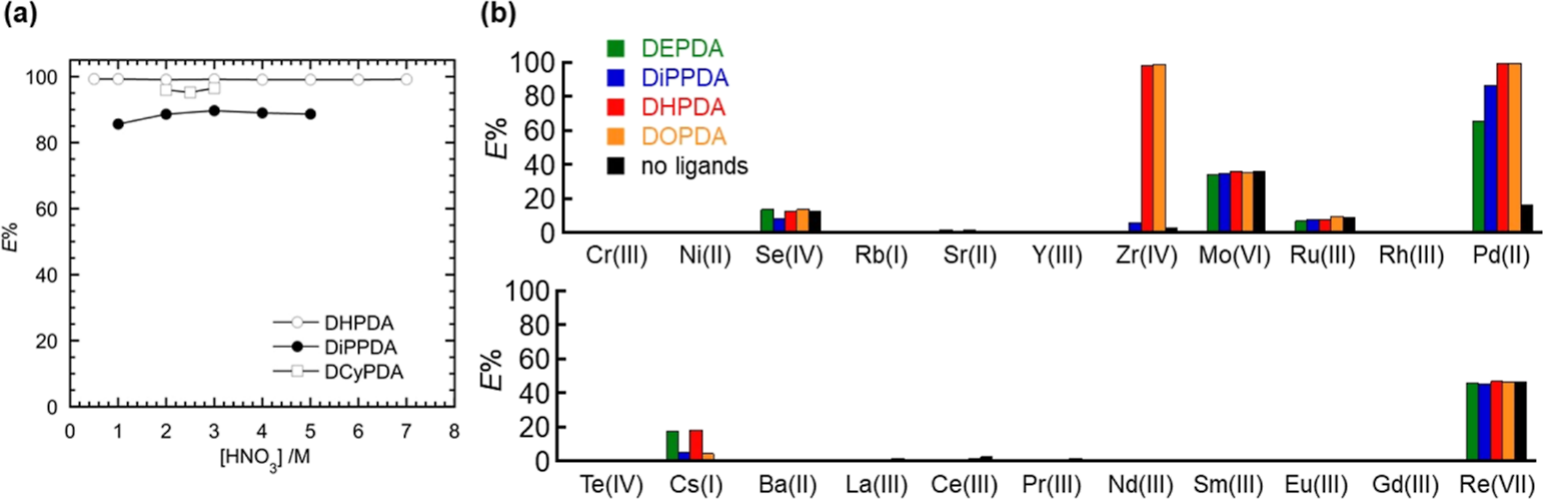
Extractabilities (*E*%) of (a) 5 mM Pd(II) from HNO_3_(aq) to 1-octanol containing 30 mM DiPPDA, DHPDA, or DCyPDA (see Figure 1b) at different [HNO_3_] after shaking for 10 min at room temperature and (b) HLLW components (M, 0.1 mM each) from 3.0 M HNO_3_(aq) to 1-octanol containing different DRPDAs (5 mM) at room temperature.

The impact of [HNO_3_] in the aqueous phase on the Pd(II) extraction was investigated. Figure 2a illustrates *E*% of Pd(II) as a function of [HNO_3_] in the 1-ocanol/HNO_3_(aq) biphasic systems under presence of 30 mM DRPDA in the organic phase. The *E*% of Pd(II) with DiPPDA, DHPDA, and DCyPDA were found to be 85.6–89.7%, 99.6–99.9%, and 95.2–96.5%, respectively. As mentioned above, the use of DRPDA with higher hydrophobicity (see Figure 1b)^32^ resulted in a higher *E*%. Notably, *E*% of Pd(II) remained largely unaffected by [HNO_3_] levels, despite a range of 0.50 to 7.0 M. This trend is much different from the former findings in which the Pd(II) extraction tends to be decreased with an increase in [HNO_3_].^25−27^ This unique feature is likely associated with the underlying reaction mechanism of the extraction process. The mechanistic details will be discussed in subsequent subsections.

It is necessary to assess the selectivity of the current extraction system, given that HLLW dissolves various elements, including fission products (FPs) and MAs generated in nuclear reactors. A solution of 3.0 M HNO_3_(aq) was used to dissolve 0.10 mM Pd(II), along with other components of HLLW, including Cr(III), Ni(II), Se(IV), Rb(I), Sr(II), Y(III), Zr(IV), Mo(VI), Ru(III), Rh(III), Te(VI), Cs(I), Ba(II), La(III), Ce(III), Pr(III), Nd(III), Sm(III), Eu(III), Gd(III), Re(VII) (0.10 mM each) was contacted with 1-octanol containing 5 mM DRPDA decorated by different terminal side chains on the N atoms. Figure 2b summarizes *E*% of all HLLW components examined in this system. Consequently, the *E*% of Pd(II) increases from 65.8% (DEPDA) to 99.2% (DOPDA) with an increase in the length of the side chains of DRPDA employed, exhibiting the same trends due to the hydrophobic nature of the selected extractant. Furthermore, it was observed that Zr(IV) was efficiently extracted by DHPDA (98.5%) and DOPDA (99.1%), whereas it was hardly extractable when DEPDA (n.d.) and DiPPDA (5.6%) were employed. As outlined in Introduction, Zr is another LLFP desirable to be separated and transmuted due to the presence of its long-lived radioisotope, ^93^Zr (*T*_1/2_ = 1.53 × 10^6^ years). Consequently, the extraction of Zr(IV) is also a priority in this work, although the mutual separation between Pd(II) and Zr(IV) remains a topic for further investigation. This issue will be addressed once the detailed reaction mechanisms of their solvent extraction have been elucidated.

Furthermore, the coextraction of Pd(II) and Zr(IV) is accompanied by the presence of several additional species, including Se(IV), Mo(VI), Ru(III), Cs(I), and Re(VII) as shown in Figure 2b. It can be reasonably assumed that Se(IV), Mo(VI), and Re(VII) are present in the current system as anionic oxo species, namely SeO_3_^2–^, MoO_4_^2–^, and ReO_4_^–^, respectively. Given that the center atoms of these anions do not form direct chemical bonds with DRPDAs, it can be postulated that they are extracted to the organic phase as conjugate acids such as H_2_SeO_3_. Indeed, Se(IV), Mo(VI), and Re(VII) exhibited minimal variation in *E*% when different DRPDA were used, and were in fact extracted to 1-octanol even in the absence of any extractants (Figure S2, Supporting Information). These results corroborate the above mechanistic hypothesis. While the details remain unclear, Ru(III) was also extractable by 1-octanol alone. The behavior of Cs(I) in this system is difficult to explain, as no clear dependency on variation of DRPDA and no extraction under absence of DRPDA were observed in Figure 2b. It is unlikely that coordination interactions of DRPDA to Cs^+^ occur, to the best of our knowledge.

Although the actual selectivity remains imperfect at this stage, and therefore requires improvement, the molecular design of DRPDA we proposed in this work has been demonstrated to be an efficient extractant for transferring Pd(II) and Zr(IV) from HNO_3_(aq), simulating HLLW, to an organic phase. To address the selectivity issue observed, the use of a nonpolar solvent such as hydrocarbons may prove beneficial. This is because H_2_SeO_3_, H_2_MoO_4_, and HReO_4_ are, to varying degrees, hydrophilic, forming noncovalent interactions, such as hydrogen bonding, with 1-octanol, which possesses an –OH group. As a preliminary test, the extraction behavior of the HLLW components (0.10 mM each) from 3.0 M HNO_3_(aq) to *n*-dodecane (*n*-C_12_H_26_), selected as a typical nonpolar solvent, was assessed in place of 1-octanol. No notable extractions of any species, with the exception of Pd(II) (*E*% = 10.8%), was observed in a *n*-dodecane/HNO_3_(aq) biphasic system devoid of any extractants (Figure S3, Supporting Information). Although mechanistic aspects of the Ru(III) and Cs(I) extractions observed in use of 1-octanol (Figure 2b) remain unclear, these species are in fact nonextractable to *n*-dodecane. It can be reasonably assumed that the extractive separation of Pd(II) and Zr(IV) from other HLLW components would be achievable using *n*-dodecane, which is a common organic solvent frequently utilized in various extraction-based chemical separation processes, including the spent fuel reprocessing for nuclear fuel recycling.

### Development of Lipophilic DRPDA Soluble in *n*-Dodecane and Extraction Behavior of HLLW Components in *n*-Dodecane/HNO_3_(aq) Systems

Unfortunately, any DRPDAs tested in Figure 2b are insoluble (DEPDA, DiPPDA) or sparingly soluble (< 5 mM; DHPDA, DOPDA) in *n*-dodecane. It is therefore imperative to develop more lipophilic DRPDAs through the lengthening and/or branching of terminal side chains on the N atoms. The synthesis of DRPDAs with R = *n*-dodecyl (DDdPDA), 2-ethylhexyl (DEhPDA), and 2-hexyldecyl (DHdPDA) has been accomplished with moderate yields (65–72%). The typical Schotten–Baumann reaction between pyridine-2,6-dicarbonyl dichloride and a primary amine having selected R, under the presence of an appropriate base, yielded the desired products. While the solubility of DDdPDA in *n*-dodecane is too low (< 5 mM), DEhPDA and DHdPDA exhibit higher solubility as much as 14 mM and over 30 mM, respectively. The introduction of branching side chains proved an effective strategy for enhancing the lipophilicity of DRPDA, thereby enabling this class of extractant to be solubilized in *n*-dodecane.

Another challenge was encountered in the extraction kinetics. Figure S4(a) in Supporting Information illustrates the time-dependent extraction of Pd(II) from 3.0 M HNO_3_(aq) to *n*-dodecane dissolving 5 mM DHdPDA. Pd(II) gradually transferred from the aqueous phase to the organic one with elapse of time. However, the distribution in this biphasic system remained incomplete even after 60 min contact. The extraction kinetics in the current system is considerably slower than those observed in the 1-octanol/HNO_3_(aq) system, where the process is completed within 5 min or less (Figure S1, Supporting Information). This was also observed in the case of DEhPDA (Figure S4b, Supporting Information). Although the use of DEhPDA would appear to result in faster extraction process than DHdPDA, the presence of an oily deposit that is immiscible with both HNO_3_(aq) and *n*-dodecane was observed. It is imperative that any potential for the formation of a third phase is eliminated in solvent extraction processes. Accordingly, DHdPDA has been selected as an extractant to be used in the solvent extraction experiments hereafter. The formation of a third phase was not confirmed in any of the conditions tested in this work when DHdPDA was used. The 2-hexyldecyl group contains an asymmetric C atom, which results in DHdPDA being optically isomeric or diastereomeric. However, for the sake of simplicity such isomerization has not been considered in the current extraction chemistry.

The slow extraction in *n*-dodecane/HNO_3_(aq) with DHdPDA can be improved by the addition of 1-octanol as a phase modifier. As shown in Figure S4a, the rate of Pd(II) extraction in this system increased with an elevated 1-octanol concentration, reaching completion within 20 min at a 20 vol % loading. In contrast, no significant extraction of the majority of HLLW components was observed following the loading of 1-octanol up to 20 vol % into *n*-dodecane in the absence of DRPDA as illustrated in Figure S5 (Supporting Information). It is noteworthy that the extraction of Pd(II) to this organic phase has occurred despite the absence of extractants. In light of the findings presented in Figure S3 (Supporting Information), it can be assumed that 1-octanol displays a certain capacity to extract Pd(II) from HNO_3_(aq). Given the rapid extraction of Pd(II) (Figure S4a) and negligible potentials for extracting other HLLW components, *n*-dodecane with 20 vol % 1-octanol was selected as an organic solvent for subsequent solvent extraction experiments in this work. To ensure the partitioning equilibrium of M of interest between the aqueous and organic phases, the biphasic samples were shaken for a minimum of 60 min.

**Figure 3:**
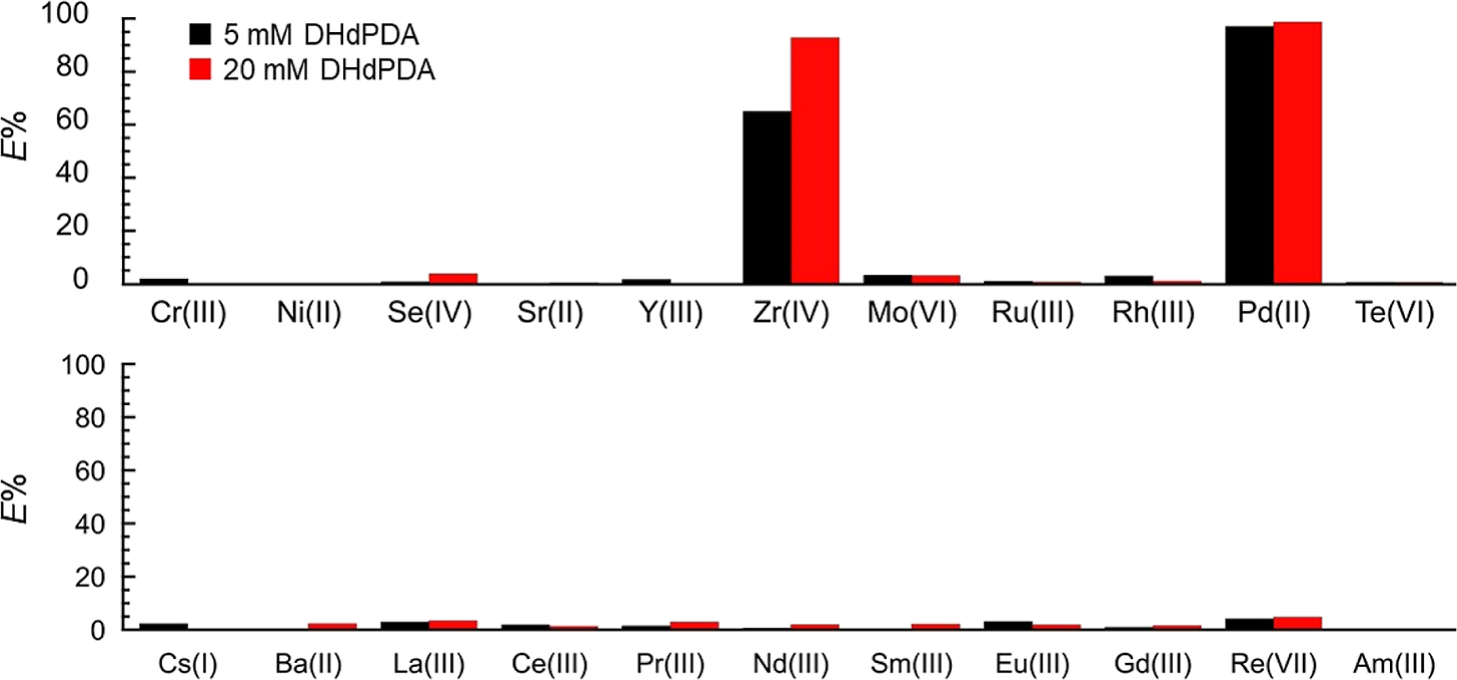
Extractability (*E*%) of HLLW components (M, 0.1 mM each) from 3.0 M HNO_3_(aq) to *n*-dodecane phase containing 20 vol % 1-octanol and DHdPDA after 60 min shaking at room temperature. ^241^Am(III) was studied in different batches from other HLLW components due to its radioactivity, and trace-level experiments have been performed.

Extraction selectivity toward the HLLW components of interest from 3.0 M HNO_3_(aq) to *n*-dodecane containing DHdPDA and 20 vol % 1-octanol has been investigated in a manner analogous to that employed in the 1-octanol/HNO_3_(aq) system illustrated in Figure 2b. As a consequence, the selective extraction of Pd(II) and Zr(IV) was confirmed (Figure 3). Furthermore, undesirable concomitance of Se(IV), Mo(VI), Ru(III), Cs(I), and Re(VII) was successfully suppressed in a manner similar to that observed in the blank test conducted in the absence of extractants (Figure S5, Supporting Information). Note that ^241^Am(III), one of the most prevalent MAs in HLLW, was also found to be negligibly extractable in these systems (Figure 3). An increase in [DHdPDA] from 5 mM to 20 mM resulted in a notable enhancement of the *E*% of Zr(IV) from 65.0% to 92.8%. Conversely, the *E*% of Pd(II) remained relatively constant at 97.1% and 98.6% at [DHdPDA] = 5 and 20 mM, respectively. The former tendency is a common occurrence in the field of extraction chemistry, while the latter implies that DHdPDA exhibits a strong affinity with Pd^2+^, allowing for nearly quantitative extraction. These trends were also observed in the subsequent discussion of the underlying mechanisms. Table 1 provides a summary of the extraction behavior of all the studied HLLW components from 3.0 M HNO_3_(aq) to *n*-dodecane containing 20 mM DHdPDA and 20 vol % 1-octanol, expressed in terms of *E*%, distribution ratios (*D*_M_) and separation factors toward Pd(II) (SF_Pd/M_) and Zr(IV) (SF_Zr/M_). The elements listed in this table, with the exception of Pd(II) and Zr(IV), are poorly extractable, as evidenced by low *D*_M_ values ranging from 10^–3^ to 10^–2^. These values are at least 3 orders of magnitude smaller than those observed for *D*_Pd_ and *D*_Zr_. Accordingly, the selectivity of this extraction system for Pd(II) and Zr(IV) was demonstrated in terms of separation factors, SF_Pd/M_ and SF_Zr/M_, in Table 1. As documented above, the mutual separation of these extractable species is necessary. To achieve this, the reaction mechanisms of the Pd(II) and Zr(IV) extraction processes must be first understood.

**Table 1:** Extractabilities (*E*%), Distribution Ratios (*D*_M_), and Separation Factors (SF_M/M′_) of HLLW Components (M) between 3.0 M HNO_3_(aq) and *n*-Dodecane +20 vol % 1-Octanol Containing 20 mM DHdPDA after Shaking for 60 min at Room Temperature^a^

M	*E*%	*D*_M_	SF_Pd/M_	SF_Zr/M_
Cr(III)	n.d^b^	<10^–2^	>10^3^	>10^3^
Ni(II)	n.d^b^	<10^–2^	>10^3^	>10^3^
Se(IV)	3.7	3.9 × 10^–2^	1.9 × 10^3^	3.3 × 10^2^
Sr(II)	0.3	2.8 × 10^–3^	2.5 × 10^4^	4.5 × 10^3^
Y(III)	0.2	2.4 × 10^–3^	3.0 × 10^4^	5.4 × 10^3^
Zr(IV)	92.8	12.9	5.6	
Mo(VI)	3.1	3.2 × 10^–2^	2.3 × 10^3^	4.1 × 10^2^
Ru(III)	0.7	6.5 × 10^–3^	1.1 × 10^4^	2.0 × 10^3^
Rh(III)	0.9	8.9 × 10^–3^	8.2 × 10^3^	1.5 × 10^3^
Pd(II)	98.6	72.5		0.18
Te(VI)	0.5	4.9 × 10^–3^	1.5 × 10^4^	2.6 × 10^3^
Cs(I)	n.d^b^	<10^–2^	>10^3^	>10^3^
Ba(II)	2.2	2.2 × 10^–2^	3.2 × 10^3^	5.7 × 10^2^
La(III)	3.3	3.4 × 10^–2^	2.1 × 10^3^	3.8 × 10^2^
Ce(III)	1.2	7.2 × 10^–3^	1.0 × 10^4^	1.8 × 10^3^
Pr(III)	3.0	3.1 × 10^–2^	2.4 × 10^3^	4.2 × 10^2^
Nd(III)	1.9	1.9 × 10^–2^	3.8 × 10^3^	6.7 × 10^2^
Sm(III)	2.2	2.2 × 10^–2^	3.3 × 10^3^	5.9 × 10^2^
Eu(III)	1.8	1.8 × 10^–2^	4.0 × 10^3^	7.1 × 10^2^
Gd(III)	1.6	1.6 × 10^–2^	4.6 × 10^3^	8.1 × 10^2^
Re(VII)	4.8	5.0 × 10^–2^	1.5 × 10^3^	2.6 × 10^2^
Am(III)^c^	n.d^b^	<10^–2^	>10^3^	>10^3^

a Variation in volume of the aqueous phase through extraction was compensated by Rb(I) concentrations before and after the extraction experiment.

b No extraction detected.

c Studied individually due to radioactivity of ^241^Am, and trace-level experiments have been done.

### Reaction Mechanism of Pd(II) Extraction with DRPDA

We further examined the extraction behavior of Pd(II) under various conditions to gain insight into the underlying chemical processes and to identify potential methods for separating Pd(II) from Zr(IV) when they are coextracted into the organic phase as illustrated in Figure 3.

**Figure 4:**
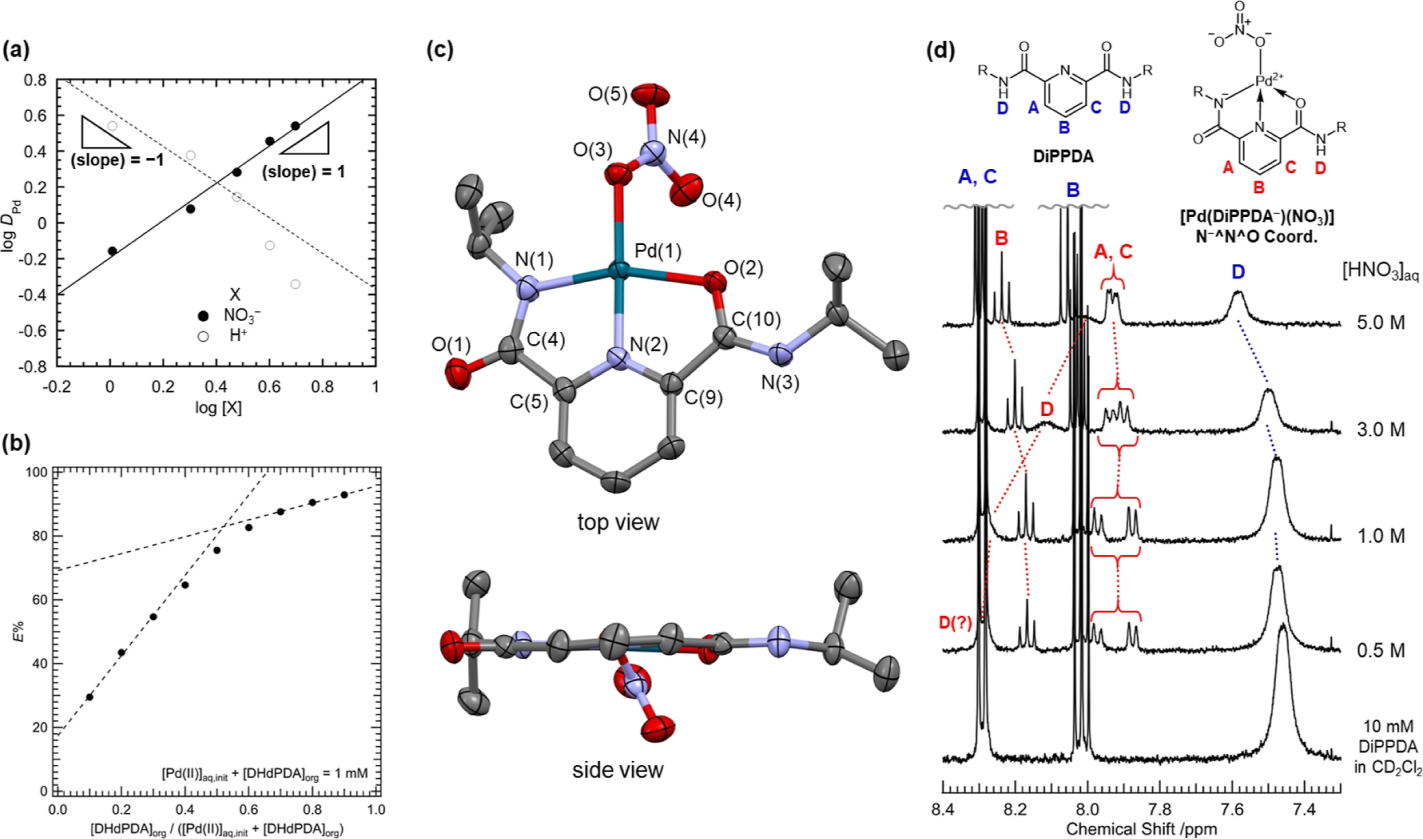
Mechanistic study on Pd(II) extraction with DRPDA. (a) Distribution ratio of Pd(II) (*D*_Pd_) from HNO_3_(aq) to CH_2_Cl_2_ containing 30 mM DiPPDA at different [X] (X = NO_3_^–^, H^+^), where HNO_3_ and NaNO_3_ were appropriately mixed to maintain concentrations of the counterions of X to the fixed values (i.e., [H^+^] was fixed to 1.0 M for [NO_3_^–^] variation; [NO_3_^–^] was fixed to 5.0 M for [H^+^] variation). (b) Job plot of Pd(II) extraction from 3.0 M HNO_3_(aq) to *n*-dodecane +20 vol % 1-octanol at different DHdPDA fraction under [Pd(II)]_aq,init_ + [DHdPDA]_org_ = 1 mM. (c) Top and side views of molecular structure of [Pd(DiPPDA^–^)(NO_3_)] determined by X-ray crystallography. Thermal ellipsoids are drawn in 80% probability level. Hydrogen atoms were omitted for clarity. Selected structural parameters were summarized in Table S2 (Supporting Information). (d) ^1^H NMR spectra of CD_2_Cl_2_ solutions containing 10 mM DiPPDA after extraction of Pd(II) (9 mM in total) from aqueous phases with different [HNO_3_]_aq_. At [HNO_3_]_aq_ = 0.5 and 1.0 M, signal D of [Pd(DiPPDA^–^)(NO_3_)] should be superposed on signal A and C of free DiPPDA. Assignments of ^1^H signals follow notations and color codes on the schematic structures of the free ligand (blue) and its Pd(II) complex (red).

Figure 4a shows the extraction behavior of Pd(II) under varying concentrations of potential reactants. Note that CH_2_Cl_2_ has been used as the organic solvent in Figure 4a instead of *n*-dodecane + 20 vol % 1-octanol. This is due to the fact that the extraction of Pd(II) in the original system is too high to allow for the clear observation of systematic trends in its extraction under different conditions (see Figure 3). The accessibility of the deuterated version of this solvent also permits the use of NMR spectroscopy to gain insights into the underlying mechanisms. In order to deliberately inhibit the extraction of Pd(II), DiPPDA was selected as an extractant in this instance. The extraction kinetics in this CH_2_Cl_2_/HNO_3_(aq) system was examined in advance, and preliminarily confirmed that the process was completed within 5 min (Figure S6 (Supporting Information)), with a much slower extraction observed when DHPDA was used instead of DiPPDA. As illustrated in Figure 2a, the Pd(II) extraction does not demonstrate any clear trend with respect to variation in [HNO_3_]. It is therefore necessary to assess the dependencies on [H^+^] and [NO_3_^–^] separately. To achieve this, HNO_3_ and NaNO_3_ were appropriately mixed, thus maintaining the concentrations of the counterion of the selected variable, log[X] (X = H^+^, NO_3_^–^), as illustrated in Figure 4a. As a result, log *D*_Pd_ increased with an increase in log[NO_3_^–^], while the opposite tendency was observed with variation of log[H^+^]. These trends indicate that NO_3_^–^ and H^+^ should be included and excluded in and from the observed Pd(II) extraction, respectively.

The following general expressions related to a liquid–liquid extraction (eqs 1 and 2) are typically considered to estimate the reaction stoichiometry.
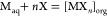
1
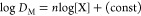
2where electric charges on any species are omitted for clarity. M and X are a metal ion of interest and a species involved in this extraction reaction, respectively. The subscripted “aq” and “org” denote their location in the aqueous and organic phases, respectively. In the log[NO_3_^–^] dependency of Figure 4a, a set of the data points is reasonably approximated with a straight line. The estimated slope for log *D*_Pd_–log [NO_3_^–^] was 1.04, implying that one NO_3_^–^ is involved in the current Pd(II) extraction.

In contrast, a curved trend was observed in the log *D*_Pd_–log [H^+^] plot of Figure 4a, and therefore, the ordinary slope analysis based on eq 2 was difficult to be applied for this data set. We should also be aware that HNO_3_ is extracted from the aqueous phase to the organic one as shown in Figure S7 (Supporting Information). This trend is enhanced more under the presence of DiPPDA due to its pyridyl N with p*K*_a_ ∼ 5. As a result, [DiPPDA] available for the Pd(II) extraction will be reduced with an increase in the aqueous acidity. Assuming that the acid extraction by DiPPDA is not very different at [HNO_3_] ≤ 3 M (Figure S7, Supporting Information), the log *D*_Pd_–log [H^+^] plot of Figure 4a seems to follow the dashed line drawn with a slope = −1. The stoichiometry of H^+^ in the current Pd(II) extraction will be discussed later.

The dependency of the Pd(II) extraction on [DiPPDA] was also investigated. Although the slope of the best-fit line was around 1, it was actually influenced by experimental conditions as shown in Figure S8 (Supporting Information). Therefore, it would be challenging to directly apply eq 2 to precisely know the stoichiometric ratio between Pd^2+^ and DiPPDA in the current extraction system. In lieu of this, the Job plot for the Pd(II) extraction from 3.0 M HNO_3_(aq) to *n*-dodecane + 20 vol % 1-octanol at different DHdPDA fraction has been done, where sum of the initial aqueous concentration of Pd(II) ([Pd(II)]_aq,init_) and [DHdPDA] in the organic phase was kept at 1 mM. As a result, a 1:1 complexation of Pd(II) with DHdPDA was suggested as shown in Figure 4b. We have further studied the Pd(II) extraction at different [DHPDA]/[Pd(II)]_aq,init_ in the 1-octanol/HNO_3_(aq) system to know whether or not such a stoichiometry is still maintained in a different extraction system. As [DHPDA]/[Pd(II)]_aq,init_ in Figure S9 (Supporting Information) increased, *E*% of Pd(II) exhibited monotonic increase, and reached >99% at [DHPDA]/[Pd(II)]_init_ > 1.2. This trend indicates that the solvent extraction of Pd(II) with DHPDA occurs in a nearly preparative manner, resulting in the formation of a 1:1 complex between these two components.

A summary of all stoichiometric data obtained from Figure 4a,b reveals that the current extraction reaction of Pd(II) with DRPDA should be written as follows.
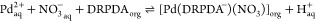
3

Although the stoichiometry of H^+^ in eq 3 has not been precisely determined yet, it should be 1 based on the above discussion for Figure 4a at the lower acidity region and also for electric neutrality of the formed Pd(II) complex to be extracted. Note that NO_3_^–^ and H^+^ are present in the reactant and product phases, respectively. This is the reason why the *E*% of Pd(II) was independent of [HNO_3_] in Figure 2a. The liberation of H^+^ represents a further distinctive feature of this extraction reaction. The most promising source of the liberated H^+^ in the current extraction system is the NH group of DRPDA, making it anionic to afford DRPDA^–^. Although Pd^2+^ is known to exclusively form the N^–^^N^N^–^ coordination shown in Figure 1b after deprotonation from both amide groups of DPRDA, it is somewhat unexpected that such H^+^ release is also observable even upon contact with the acidic aqueous phase with HNO_3_. It is evident that such deprotonation from amide NH will be markedly unfavorable under acidic aqueous conditions, as evidenced by p*K*_a_ of *N*-monosubstituted amides, which ranges from 18 to 26.^48−50^ If this occurs, the resulting singly deprotonated species, DRPDA^–^, will adopt the N^–^^N^O coordination of Figure 1b, which has not been observed previously in Pd(II) complexes.

Following a series of trials, we have successfully achieved the crystallization of [Pd(DiPPDA^–^)(NO_3_)] from the isolated CH_2_Cl_2_ phase following the extraction of Pd(II) from HNO_3_(aq). Figure 4c shows the molecular structure of [Pd(DiPPDA^–^)(NO_3_)] as determined by X-ray crystallography. This chemical composition was corroborated by elemental analysis. It is noteworthy that the anticipated N^−^^N^O coordination of DRPDA^–^ is indeed observed in its molecular structure, through direct bonding interactions involving the deprotonated N(1) with the Pd(1) center, in conjunction with coordination bonds from N(2) of the pyridyl backbone and O(2) of an additional terminal amide nondeprotonated. Moreover, this complex is identical to that proposed in the reaction mechanism of the Pd(II) extraction (eq 3). An additional coordination of NO_3_^–^ to the Pd^2+^ center occurs, resulting in the formation of a square planar geometry, which is a typical feature observed in Pd(II) complexes. However, this square geometry is somewhat distorted due to the bite angles of the chelating DiPPDA narrower than 90°; N(1)–Pd(1)–N(2): 81.22(6)° and N(2)–Pd(1)–O(2): 80.05(5)°. Although the N^−^^N^O coordination was initially unexpected, the planarity of the sp^2^ conjugated system throughout the 2,6-pyridinediamide moiety was successfully achieved as anticipated in the original concept for the molecular design of DRPDA. Although this molecular structure is also permitted by the N^–^^N^O^–^ coordination of Figure 1b after the additional deprotonation from the other NH, it is reasonably rejected by the charge balance of this Pd(II) complex isolated here, its chemical formula determined by elemental analysis, and extraction stoichiometry in eq 3.

This study represents the first confirmation of the N^−^^N^O coordination of DRPDA^–^ to Pd^2+^. Selected structural parameters are summarized in Table S2 (Supporting Information). The Pd(1)–N(1) distance (2.0054(14) Å) is, somewhat shorter than, but still comparable with, Pd–N^–^ interactions (ca. 2.04 Å) observed in previously reported Pd(II) complexes bearing N^–^^N^N^–^ coordinating DRPDA^2–^ after deprotonation from NH moieties of both amide groups.^51−56^ The N(2) atom of the pyridyl group is bound to Pd(1) in a shorter distance (1.9203(14) Å) compared with Pd(1)–N(1) despite its noncharged status. A similar trend is observed in the known ^–^ analogues of Pd(II) complexes, with a distance of ca. 1.93 Å. Pd^2+^ also interacts with O atoms of DiPPDA^–^ (Pd(1)–O(2): 2.0937(11) Å) and NO_3_^–^ (Pd(1)–O(3): 2.0482(12) Å). These Pd–O distances are longer than the Pd–N distances. This trend aligns with the HSAB principle,^57^ whereby Pd^2+^ is a soft acid that prefers to be more strongly coordinated by a soft N donor compared to a hard O donor. The identical crystalline compound was also isolated from a stoichiometric mixture of Pd^2+^ and DiPPDA in a 50:50 v/v HNO_3_(aq)-MeOH system, indicating that the N^−^^N^O coordination of DRPDA^–^ to Pd^2+^ in the current extraction system is commonly favored regardless of whether the system is in an aqueous or organic phase.

Nature of chemical bonding formed in [Pd(DiPPDA^–^)(NO_3_)] was theoretically explored by DFT calculations. The optimized structure shown in Figure S10 (Supporting Information) is in accordance with that of Figure 4c determined through experiments, where Pd(1)–N(1): 2.015 Å, Pd(1)–N(2): 1.947 Å, Pd(1)–O(2): 2.173 Å, and Pd(1)–O(3): 2.072 Å are found in the computational result. The planarity observed in the 2,6-pyridinediamide moiety of DiPPDA^–^ was also reproduced in the calculated structure. A natural bond orbital (NBO) analysis was conducted on the N^–^^N^O-type complex, resulting in the identification of Wiberg bond indices (WBI) of 0.511, 0.446, and 0.195 for Pd(1)–N(1), Pd(1)–N(2), and Pd(1)–O(2), respectively. The NBO analysis indicated that the Pd–N bond is stronger for the amide N than for the pyridine N, thereby supporting the prevalence of the N^–^^N^O-type. A natural bonding orbital was identified, comprising 72% N (23% s, 77% of p) and 28% Pd orbitals (13% of s and 87% of d), with an occupancy of 1.960 e^–^ (Figure S11a, Supporting Information). Furthermore, an antibonding orbital of the same mixture (28% N, 72% Pd) is identified, with an occupancy of 0.42633 e^–^ (Figure S11b, Supporting Information). The latter is attributed to significant back-donation from the O(2) and N(2) atoms, with donor–acceptor interactions of 42.06 and 18.92 kcal·mol^–1^, respectively, toward the Pd–N antibonding orbital. In Pd(II) complexes, the typical back-donation observed occurs in the opposite direction,^58^ namely from Pd to the ligand antibonding orbital. This results in an electron-poor Pd and weakened ligand intramolecular bonds, which contribute significantly to the peculiar catalytic behavior of Pd(II) compounds. This is not the case in the current system, where the formation of a stable Pd(II)-DRPDA^–^ complex has been observed. This complex is robust to harsh solvent extraction processes under strongly acidic conditions.

The deprotonated amide group (O=C–N^–^–R) of DRPDA^–^ can be tautomerized to form iminolate (O^–^–C=N–R), thereby enabling an alternative coordination mode, O^–^^N^O (Figure 1b). Structure optimization of the Pd(II) complex with O^−^^N^O coordinating DiPPDA^–^ was in fact conducted (Figure S12, Supporting Information), however its occurrence is unfavorable due to a higher formation energy (+14.3 kcal·mol^–1^) compared with its N^−^^N^O tautomer of Figure S11 (Supporting Information) as demonstrated experimentally (Figure 4c). In fact, the O^−^^N^O structure is exceedingly uncommon, with only a K^+^ salt having been observed to date.^59^

It is not always the case that an isolated crystalline deposit describes in full the molecular structure observed in the parent solution. To clarify what Pd(II) species is actually extracted, ^1^H NMR spectra of CD_2_Cl_2_ solutions with 10 mM DiPPDA were obtained following the extraction of Pd(II) from HNO_3_(aq) (Figure 4d). At [HNO_3_]_aq_ = 1.0 M, a set of chemically inequivalent ^1^H signals arising from the pyridyl moiety of DiPPDA were observed at 7.87 ppm (doublet), 7.97 ppm (doublet), and 8.18 ppm (triplet), which are distinguishable from the free form of the ligand appeared at 8.02 and 8.29 ppm. This result indicates that an asymmetric chemical environment is provided for DiPPDA following complexation with Pd^2+^ through solvent extraction. Despite the acidity of the aqueous phase contacted through the solvent extraction affecting the peak positions, the same set of ^1^H signals were consistently detected across different [HNO_3_]_aq_ conditions. Furthermore, an additional broad signal attributable to the remaining >NH in this Pd(II) complex also appeared apart from that of the free ligand (7.4–7.6 ppm). This signal was upfield-shifted from 8.3 to 8.0 ppm with an increase in [HNO_3_]_aq_, which is a typical trend for hydrogen-bonded ^1^H upon contact with different acidities. Occurrence of [Pd(DHdPDA^–^)(NO_3_)] extracted from 3.0 M HNO_3_(aq) to *n*-dodecane + 20 vol % 1-octanol was also confirmed by ^1^H NMR as displayed in Figure S13 (Supporting Information). The observed signal set is comparable with that in Figure 4d in terms of peak integrals, while the signals were rather broadened or, especially for H_A_ and H_C_, coalesced in the *n*-dodecane-based system due to chemical exchange between the N^−^^N^O and O^N^N^–^ coordination modes shown in Figure S13c. The results obtained from the current NMR experiments provide a reasonable explanation for the formation of [Pd(DRPDA^–^)(NO_3_)] (Figure 4c) as an extractable species.

### Reaction Mechanism of Zr(IV) Extraction with DRPDA

**Figure 5:**
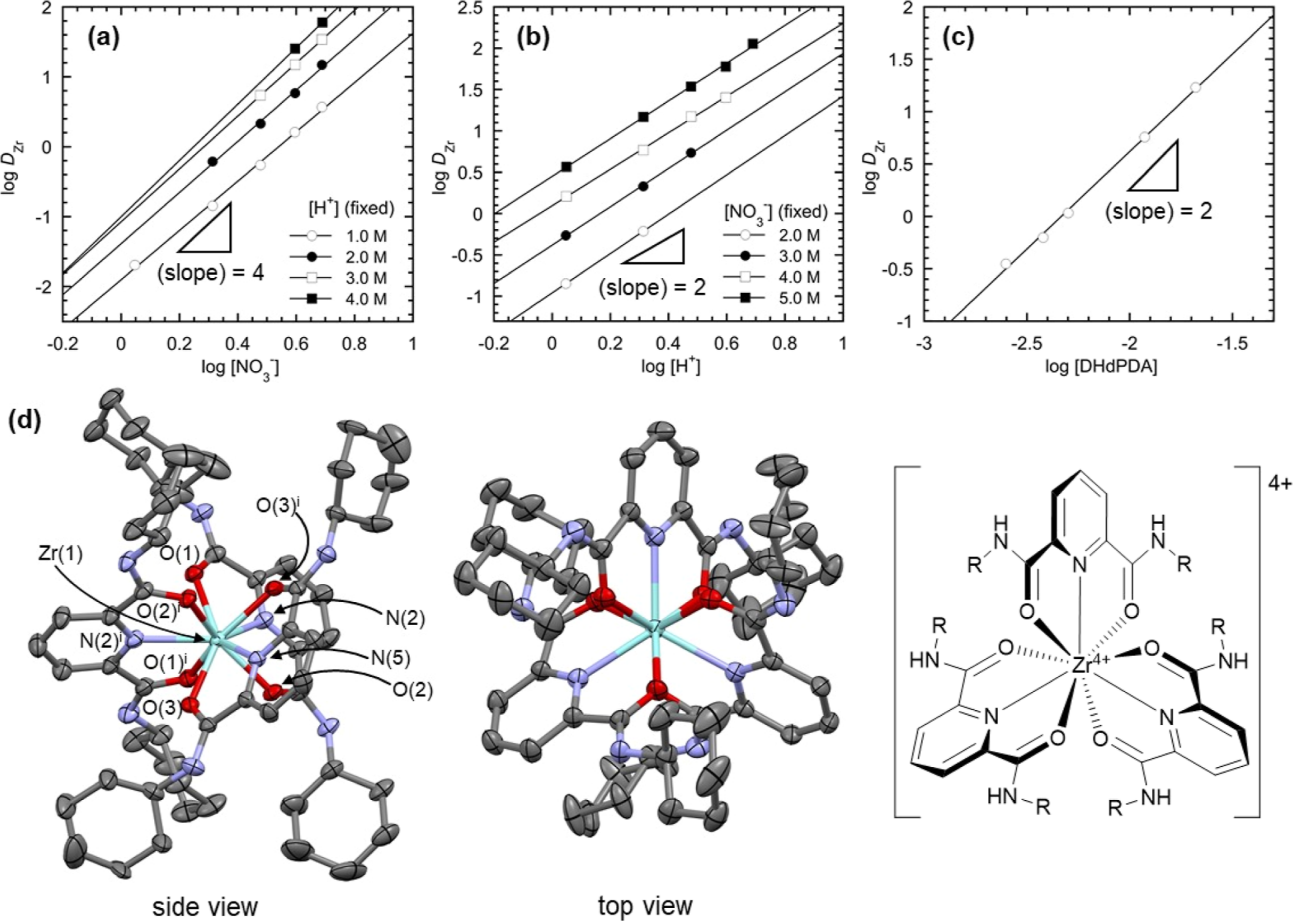
Mechanistic study on Zr(IV) extraction with DRPDA. Distribution ratio of Zr(IV) (*D*_Zr_) from HNO_3_(aq) to *n*-dodecane containing 20 mM DHdPDA and 20 vol % 1-octanol at different [X] (X = NO_3_^–^ (a), H^+^ (b)), where HNO_3_ and NaNO_3_ were appropriately mixed to maintain concentrations of the counterparts of X (i.e., H^+^, NO_3_^–^, respectively) to the fixed values described in the legends. (c) *D*_Zr_ from 3.0 M HNO_3_(aq) to *n*-dodecane +20 vol % 1-octanol at different [DHdPDA]. (d) Side and top views of molecular structure of Δ-[Zr(DCyPDA)_3_]^4+^ in its pentahydrated tetranitrate salt [Zr(DCyPDA)_3_](NO_3_)_4_·5H_2_O, determined by X-ray crystallography together with its schematic drawing. Thermal ellipsoids are drawn in 50% probability level. Hydrogen atoms were omitted for clarity. Symmetry operation: (i) 1–*x*, *y*, 1/2–*z*. Bond distances Zr(1)–O(1): 2.213(2) Å, Zr(1)–O(2): 2.2008(19) Å, Zr(1)–O(3): 2.213(2) Å, Zr(1)–N(2): 2.363(2) Å, Zr(1)–N(5): 2.350(3) Å, C_yl_ = O: 1.27 Å (mean), C_yl_–N: 1.31 Å (mean).

The extraction behavior of Zr(IV) was also studied in a manner analogous to that employed for Pd(II) in the preceding section. Figure 5a–c shows log *D*_Zr_ under different logarithmic concentrations of species potentially involved in the reaction, where *n*-dodecane containing 20 vol % 1-octanol and DHdPDA was employed as an organic phase. In the log[NO_3_^–^] dependency under fixed [H^+^] conditions (Figure 5a), the slopes of the best fit lines at different [NO_3_^–^] are observed to range from 3.53 to 4.03. This implies that the stoichiometric ratio of NO_3_^–^ in the Zr(IV) extraction should be 4. In a similar manner to this, Figure 5b,c indicate that two H^+^ and two DHdPDA, in comparison to Zr^4+^, are involved in this reaction. Indeed, the slope value of the best fit line of log *D*_Zr_–log[HNO_3_] plot (Figure S14, Supporting Information) is 5.78, which is close to the sum of two H^+^ and four NO_3_^–^ determined individually in Figure 5a,b, respectively. It should be noted that DHdPDA is an extractant, while NO_3_^–^ serves as a charge compensator for Zr^4+^. In contrast, the presence of H^+^ in the reactant part is a distinct feature that differs significantly from the Pd(II) extraction reaction of eq 3. It seems unlikely that protonation of DHdPDA is involved in this process, given that the positive charge that needs to be compensated in this extraction reaction is enhanced despite the lack of further NO_3_^–^ available in this stoichiometry. Given the strong hydrolysis tendency of Zr^4+^,^60,61^ it can be assumed that a hydrolyzed species of Zr^4+^ is an actual reactant in the current Zr(IV) extraction. Based on the aqueous thermodynamics of Zr^4+^ and the stoichiometric ratios obtained from Figure 5a–c, the following reactions are proposed as the most reliable means of describing the current Zr(IV) extraction.
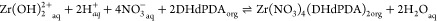
4
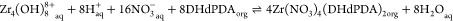
5

The aquo species, Zr^4+^(aq), is the most predominant at pH ∼ 0 as shown in a species distribution as a function of pH (Figure S15a, Supporting Information) based on reported stability constants.^60^ Nevertheless, the hydrolysis of Zr^4+^ must be considered in order to explain the involvement of H^+^ in the reactant portion. Among the potential hydrolyzed products of Zr^4+^ in Figure S15b (Supporting Information), only Zr(OH)_2_^2+^ and Zr_4_(OH)_8_^8+^ align with the stoichiometric ratio Zr^4+^/H^+^/NO_3_^–^/DHdPDA = 1:2:4:2 determined here. Although it is currently challenging to ascertain which reaction is indeed accurate, it seems that eq 4 of Zr(OH)_2_^2+^ may be a more plausible explanation than eq 5 of Zr_4_(OH)_8_^8+^ given its higher abundance at pH ∼ 0 (Figure S15b, Supporting Information).

Additionally, several attempts were made to isolate a crystalline Zr(IV)-DRPDA, with a view to understanding its extraction chemistry. Zr(IV) was extracted from 3.0 M HNO_3_(aq) to a CH_2_Cl_2_ phase containing 20 mM DCyPDA (R = cyclohexyl, Figure 1b). Slow evaporation of the separated organic phase afforded colorless precipitate, which were not suitable for X-ray structure determination. The recovered solid material was recrystallized from a mixture of 3.0 M HNO_3_(aq) and MeOH by evaporation of the solvent to finally afford crystalline deposits suitable for X-ray crystallography. Figure 5d depicts the molecular structure of [Zr(DCyPDA)_3_]^4+^ present in the obtained colorless crystals of [Zr(DCyPDA)_3_](NO_3_)_4_·5H_2_O. Although NO_3_^–^ is only available as a counteranion of this Zr(IV) complex, no discrete atomic sites have been modeled due to significant disorder of NO_3_^–^. Indeed, the contribution from four NO_3_^–^ ions together with four additionally missing water molecules was successfully removed after solvent mask treatment.^62^ The number of e^–^ added to this crystal structure was 684 e^–^ at 1832 Å^3^ void in the crystal lattice. This value is in close alignment with the 672/4 e^–^/Z arising from (4 × 32 e^–^) = 128 e^–^ of four NO_3_^–^ and (4 × 10 e^–^) = 40 e^–^ of four H_2_O per molecule. The central Zr(1) atom is nine-coordinated by three tridentate DCyPDA molecules in the noncharged O^N^O coordination mode (Figure 1b), resulting in a tricapped trigonal prismatic coordination geometry. Each DCyPDA is located along a diagonal line of the rectangular surface of the trigonal prism, thereby imparting a helical character to this Zr(IV) complex. The crystal lattice contains both Δ and Λ enantiomers of [Zr(DCyPDA)_3_]^4+^, resulting in its racemic mixture. The mean distances for the Zr–O and Zr–N bonds are 2.21 and 2.36 Å, respectively. This represents the first structure of a Zr(IV)-DRPDA complex to be reported.

Note that the isolated [Zr(DCyPDA)_3_]^4+^ of Figure 5d does not directly elucidate the actual extraction chemistry of Zr(IV) shown in eq 4 or 5. This can be attributed to the repeated deposition and dissolution processes employed to obtain single crystals of [Zr(DCyPDA)_3_](NO_4_)_4_·5H_2_O suitable for X-ray structure determination. It remains unclear which Zr(IV) complex is formed as an extractable species. Nevertheless, the O^N^O coordination of noncharged DCyPDA was verified in the crystal structure depicted in Figure 5d. Given the hardness of Zr^4+^ as a Lewis acid,^57^ it can be postulated that the same coordination mode of DHdPDA should also occur in [Zr(NO_3_)_4_(DHdPDA)_2_] of eqs 4 and 5 through the extraction of Zr(IV) from HNO_3_(aq) to *n*-dodecane-based organic phase of Figure 5a–c. In contrast to the Pd(II) case (Figure 4d), ^1^H NMR spectroscopy was not a suitable method for further exploration of the coordination chemistry of extracted Zr(IV) in the organic phase due to rapid chemical exchange, which averaged the NMR signals of Zr(IV) species and DRPDA present there.^63,64^

### Selective Recovery of Pd(II) and Zr(IV) from Simulated HLLW

Although the detailed molecular structure of the extracted species of Zr(IV) [Zr(NO_3_)_4_(DHdPDA)_2_], remains unclear, the reaction mechanisms of solvent extraction of both Pd(II) and Zr(IV) from HNO_3_(aq) to *n*-dodecane containing 20 vol % 1-octanol and DRPDA have been successfully concluded. The effective decontamination of these materials from other HLLW components has already been demonstrated in Figure 3. The next step is to consider how to separately recover Pd(II) and Zr(IV) present in the organic phase. One of the critical differences in their mechanistic aspects is the dependency on [HNO_3_]_aq_. *E*% of Pd(II) remains constant at any [HNO_3_]_aq_ (Figure 2a; eq 3), whereas that of Zr(IV) is significantly affected by [HNO_3_]_aq_ (Figure S14 (Supporting Information); eqs 4, 5). Consequently, Zr(IV) can be stripped in the presence of a diluted HNO_3_(aq), while Pd(II) remains exclusively present in the organic phase. For instance, it can be extrapolated from Figure S14b (Supporting Informtion) that nearly quantitative recovery of Zr(IV) upon contact with 0.1 M HNO_3_(aq) is to be expected, given that log *D*_Zr_ is close to −8. Following the stripping of Zr(IV), Pd(II) remaining in the organic phase could be back-extracted to HCl(aq), given that Pd(II) is known to form a stable limiting complex, [PdCl_4_]^2–^, which is highly soluble in an aqueous phase.^9^ It is necessary to ascertain whether Pd^2+^ actually exhibits a greater preference for Cl^–^ compared with DRPDA in the current system.

**Figure 6:**
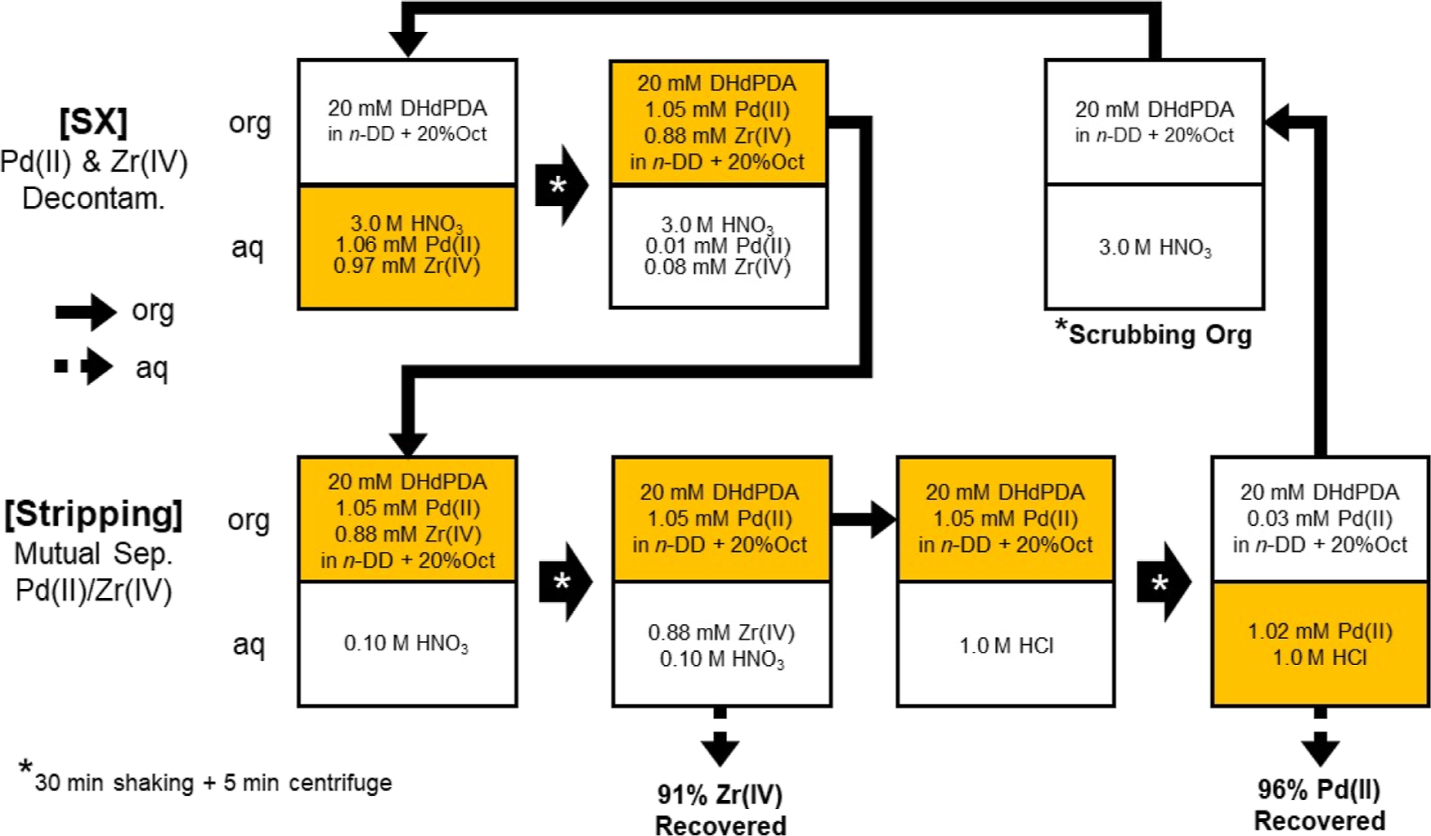
Process flow of decontamination of Pd(II) and Zr(IV) in 3.0 M HNO_3_(aq) with solvent extraction to *n*-dodecane + 20 vol % 1-octanol phase containing 20 mM DHdPDA and mutual separation of them using stripping agents appropriately selected. SX: solvent extraction, *n*-DD: *n*-dodecane, Oct: 1-octanol. Note that each extraction/stripping stage in this work consisted of a single contact of the aqueous and organic phases. Multistep counter-current extraction can be applied to gain purity and recovery as high as requested.

Figure 6 illustrates the process flow for the decontamination of Pd(II) and Zr(IV) from other HLLW components in 3.0 M HNO_3_(aq) with solvent extraction to *n*-dodecane + 20 vol % 1-octanol containing 20 mM DHdPDA. This is followed by mutual separation of the aforementioned components using stripping agents that have been selected on an appropriate basis. This sequential separation process was employed for demonstration purposes. At the initial stage of the process, 1.06 mM Pd(II) and 0.97 mM Zr(IV) in 3.0 M HNO_3_(aq) were extracted concurrently with the *n*-dodecane phase containing 20 vol % 1-octanol and 20 mM DHdPDA in 99.0% and 90.7% yield, respectively, after 30 min of agitation at room temperature. The separated organic phase was contacted with 0.10 M HNO_3_(aq), where Zr(IV) was selectively transferred to the aqueous phase in a quantitative manner due to the significant variation in [HNO_3_] (Figure S14, Supporting Information). In contrast, the back-extraction of Pd(II) was not observed at this stage as expected. Following the removal of the aqueous layer, the organic phase was subjected to further treatment with 1.0 M HCl(aq). The stripping of Pd(II) was successfully achieved in 97.1% yield. Consequently, 96.2% Pd(II) and 90.7% Zr(IV) were separately recovered in this sequential stripping process. Note that each extraction/stripping stage in this work consisted of a single contact of the aqueous and organic phases. In order to achieve the desired purity and recovery of Pd(II) and Zr(IV), multistep counter-current extraction can be adopted as usual.^2^

Prior to reusing the extracting solvent in the next separation sequence of Figure 6, it is advisible to wash the organic phase with HNO_3_(aq). This is because HCl or Cl^–^ could be extracted through the second stripping stage for Pd(II) recovery, which would disrupt the subsequent separation cycle. Indeed, the *E*% of both Pd(II) and Zr(IV) exhibited notable decline in the second cycle when the organic phase, following the Pd(II) stripping with 1.0 M HCl(aq), was reused without any further treatment (Figure S16a, Supporting Information). In contrast, such trends were effectively prevented by preliminary scrubbing of the used organic solvent with 3.0 M HNO_3_(aq) (Figure S16b, Supporting Information).

## Conclusion

In this work, *N*,*N*′-dialkyl-2,6-pyridinediamide (DRPDA) was employed in solvent extraction chemistry to facilitate the removal of selected HLLW components from HNO_3_(aq) and their subsequent transfer into nonaqueous molecular solvents such as 1-octanol and *n*-dodecane. While at the outset of this research, the molecular design of DRPDA was introduced solely to secure the O^N^O planar tridentate coordination by removing the steric hindrance present in the previously employed *N*,*N*,*N*′,*N*′-tetraalkyl derivative (TRPDA) for PGMs extraction. However, the broader diversity of coordination modes of DRPDA ultimately facilitated the efficient and selective separation of Pd(II) and Zr(IV) from other HLLW components in HNO_3_(aq). An exotic N^−^^N^O coordination of DRPDA has been observed in Pd(II) coordination chemistry for the first time, which efficiently drives solvent extraction of Pd(II) in the current system after deprotonation from one of its amide NH groups. It is noteworthy that this reaction occurs even upon contact with an aqueous phase containing [HNO_3_] = 0.50–7.0 M. This observation suggests that the chemical bonding between Pd^2+^ and N^–^ of DRPDA^–^ is sufficiently strong to facilitate deprotonation of the amide NH group, which has a p*K*_a_ range of 18–26. Due to involvement of NO_3_^–^ and H^+^ in the reactant and product parts of the extraction reaction of Pd(II), respectively, concentration effects of these ions are canceled out, making extraction efficiency of Pd(II) independent of the [HNO_3_] variation tested here. Additionally, Zr(IV) is also extracted by DRPDA with high efficiency, with the formation of the intended O^N^O coordination of noncharged DRPDA. Based on the above findings, separation of Pd(II) and Zr(IV) from any other HLLW components including Am(III), one of typical MAs, was successfully achieved by employing *n*-dodecane-based nonaqueous phase containing DHdPDA and 20 vol % 1-octanol. The former bears 2-hexyldecy groups on both amide N atoms, rendering it hydrophobic enough to solubilize itself in *n*-dodecane, while the latter is required to enhance extraction kinetics. Co-extracted Pd(II) and Zr(IV) were mutually separated through the stripping processes, where diluted HNO_3_(aq) and HCl(aq) were stepwise used to recover Zr(IV) and Pd(II), respectively.

In conclusion, it has been demonstrated that the tridentate coordination modes of DRPDA are interchangeable with each other, providing appropriate complexation with some specific metal ions such as Pd^2+^ and Zr^4+^. This enables efficient and selective solvent extraction of these metal ions. Effective utilization of organometallic interactions in solvent extraction chemistry, as demonstrated in this work, will facilitate the development of new chemical separation principles not only in the HLLW treatments of the nuclear fuel cycle, but also in other extraction-based separation processes.

## Experimental Section

^1^H and ^13^C NMR spectra were recorded using a JEOL JNM ECX-400 spectrometer (^1^H: 399.78 MHz, ^13^C: 100.53 MHz). CHN elemental analyses were performed using a Yanaco CHN Corder MT-6. Acid extraction in an organic phase was quantified by DKK-TOA AUT-701 automatic titrator. Molecular and crystal structures of DRPDA complexes of various metal ions were determined by single crystal X-ray diffraction using Rigaku XtaLab mini II with graphite-monochromated Mo Kα radiation (λ = 0.71075 Å). The diffraction data obtained were analyzed using the Olex2^62^ software package in conjunction with SHELX.^65^ The structure was solved by SHELXT and expanded using Fourier techniques. All non-hydrogen atoms were anisotropically refined by SHELXL 2017/1.^66^ Hydrogen atoms were refined as riding on their parent atoms with *U*_iso_(H) = 1.2 *U*_eq_(C, H). The final cycle of the full-matrix least-squares refinement of *F*^2^ was based on the observed reflections and parameters and converged with the unweighted and weighted agreement factors, *R* and *wR*, respectively. Concentrations of each element studied here in aqueous phases were determined by ICP-AES (Thermo Scientific iCAP7200Duo) or ICP–MS (Agilent ICP–MS 7700x). Milli-Q water was used for preparation of aqueous samples. All the chemicals were of reagent grade, and used as received.

### General Procedure for Synthesis of DRPDA Ligands

A selected primary amine (ca. 10% excess, TCI) was dropwise added to a mixture of pyridine-2,6-dicarbonyl dichloride (TCI) and K_2_CO_3_ (excess) in THF at 0 °C. This mixture was stirred vigorously overnight. After suction filtration, the filtrate was evaporated to dryness. The residue was dissolved in CH_2_Cl_2_, and washed with 2 M HCl(aq), 2 M Na_2_CO_3_(aq), and brine. The separated organic phase was dried over MgSO_4_. The separated supernatant was evaporated to dryness to yield DRPDA. Most of the products obtained were colorless or opaque solids, unless otherwise specified. The compounds obtained were characterized by ^1^H and ^13^C NMR spectroscopy.

#### *N*,*N*′-Diethyl-2,6-pyridinediamide (DEPDA)

Yield: 75%. ^1^H NMR (CDCl_3_, 298 K, δ/ppm vs TMS): 8.34 (d, 2H, 3,5 H), 8.02 (t, H, 4 H), 7.66 (t, 2H, NH), 3.57 (br, 4H, NC*H*_2_CH_3_), 1.31 (t, 6H, NCH_2_C*H*_3_). ^13^C NMR (CDCl_3_, δ/ppm vs TMS): 163.46, 149.05, 139.04, 124.98, 34.59, 14.97.

#### *N*,*N*′-Diisopropyl-2,6-pyridinediamide (DiPPDA)

Yield: 37%. ^1^H NMR (CDCl_3_, 298 K, δ/ppm vs TMS): 8.35 (d, *J* = 8.0 Hz, 2H), 8.01 (t, *J* = 7.7 Hz, 1H), 7.57 (bd, *J* = 7.7 Hz, 2H), 4.36–4.27 (m, 2H), 1.31 (d, *J* = 6.8 Hz, 12H). ^13^C NMR (CDCl_3_, 298 K, δ/ppm vs TMS): 162.6, 149.0, 138.8, 124.9, 41.6, 22.6.

#### *N*,*N*′-Di-*n*-hexyl-2,6-pyridinediamide (DHPDA)

Yield: 64% after trituration with *n*-hexane. ^1^H NMR (CDCl_3_, 298 K, δ/ppm vs TMS): 8.36 (d, *J* = 7.9 Hz, 2H), 8.02 (t, *J* = 7.9 Hz, 1H), 7.72 (bt, 2H), 3.51 (q, *J* = 6.9 Hz, 4H), 1.77–1.55 (m, 4H), 1.49–1.21 (m, 14H), 0.96–0.85 (m, 8H). ^13^C NMR (CDCl_3_, 298 K, δ/ppm vs TMS): 163.5, 148.9, 138.9, 124.8, 39.6, 31.4, 29.6, 26.6, 22.4, 13.9.

#### *N*,*N*′-Dicyclohexyl-2,6-pyridinediamide (DCyPDA)

Yield: 65%. ^1^H NMR (CDCl_3_, 298 K, δ/ppm vs TMS): 8.32 (d, 2H), 7.98 (t, 1H), 7.66 (bd, 2H), 3.98 (br, 2H), 1.14–2.00 (br, 20H). ^13^C NMR (CDCl_3_, 298 K, δ/ppm vs TMS): 162.55, 149.21, 138.95, 125.15, 48.31, 33.07, 25.63, 24.80.

#### *N*,*N*′-Di-*n*-octyl-2,6-pyridinediamide (DOPDA)

Yield: 62% after trituration with *n*-hexane. ^1^H NMR (CDCl_3_, 298 K, δ/ppm vs TMS): 8.35 (d, *J* = 7.6 Hz, 2H), 8.02 (t, *J* = 7.8 Hz, 1H), 7.71 (bt, 2H), 3.51 (q, *J* = 6.9 Hz 4H), 1.72–1.56 (m, 6H), 1.46–1.20 (m, 20H) 0.88 (t, *J* = 6.8 Hz, 6H). ^13^C NMR (CDCl_3_, 298 K, δ/ppm vs TMS): 163.5, 148.9, 138.9, 124.8, 39.7, 31.7, 29.7, 29.24, 29.17, 27.0, 22.6, 14.0.

#### *N*,*N*′-Di(2-ethylhexyl)-2,6-pyridinediamide (DEhPDA)

Yellow oil. Yield: 72%. ^1^H NMR (CDCl_3_, 298 K, δ/ppm vs TMS): 8.36 (d, *J* = 7.9 Hz, 2H), 8.03 (t, *J* = 7.9 Hz, 1H), 7.79 (bt, 2H), 3.45 (m, 4H), 1.66–1.54 (m, 2H), 1.50–1.20 (m, 16H) 1.00–0.78 (m, 12H). ^13^C NMR (CDCl_3_, 298 K, δ/ppm vs TMS): 163.4, 148.9, 139.0, 124.8, 42.3, 39.5, 31.2, 28.9, 24.4, 22.9, 14.0, 10.9.

#### *N*,*N*′-Di-*n*-dodecyl-2,6-pyridinediamide (DDdPDA)

Yield: 65% after silica-gel column chromatography (EtOAc). ^1^H NMR (CDCl_3_, 298 K, δ/ppm vs TMS): 8.35 (d, *J* = 7.9 Hz, 2H), 8.02 (t, *J* = 7.9 Hz, 1H), 7.81 (bt, 2H), 3.50 (q, 4H), 1.66 (quintet, 4H), 1.45–1.20 (br, 38H) 0.88 (t, *J* = 6.8 Hz, 6H). ^13^C NMR (CDCl_3_, 298 K, δ/ppm vs TMS): 163.4, 148.9, 139.0, 124.9, 39.7, 31.9, 27.7, 29.6, 29.6, 29.6, 29.3, 27.1, 22.7, 14.1.

#### *N*,*N*′-Di(2-hexyldecyl)-2,6-pyridinediamide (DHdPDA)

Yield: 69% after silica-gel column chromatography (EtOAc). ^1^H NMR (CDCl_3_, 298 K, δ/ppm vs TMS): 8.35 (d, *J* = 7.7 Hz, 2H), 8.03 (t, *J* = 7.7 Hz, 1H), 7.65 (bt, 2H), 3.45 (q, *J* = 6.0 Hz 4H), 1.40–1.10 (m, 50H), 0.92–0.78 (m, 12H). ^13^C NMR (CDCl_3_, 298 K, δ/ppm vs TMS): 163.4, 148.9, 139.0, 124.9, 42.8, 38.1, 32.1, 31.9, 31.8, 30.0, 29.7, 29.6, 29.6, 29.3, 26.7, 22.6, 14.1.

## Solvent Extraction

A starting material of each M (Table S1, Supporting Information) was dissolved in HNO_3_(aq) presaturated with an organic solvent (1-octanol, CH_2_Cl_2_, or *n*-dodecane). The organic solvents were also preliminarily saturated with HNO_3_(aq), and used for prepration of organic phases. An aliquot of the aqueous solution was contacted with an organic phase containing DRPDA in 1:1 v/v, followed by shaking at 1800 rpm at room temperature, 298 ± 1 K. After centrifugation, the aqueous concentration of M was determined by ICP–MS. Extractability (*E*%), distribution ratio (*D*_M_), and separation factor of M from M′ (SF_M/M′_) are defined as follows.
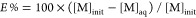
6
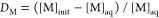
7
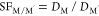
8where [M]_init_ and [M]_aq_ denote the M concentrations in the aqueous phase at the initial state and after the extraction, respectively.

*Caution*! ^241^Am is an α- and γ-emitting radioactive isotope (specific activity: 1.27 × 10^11^ Bq·g^–1^ with *T*_1/2_ = 432 years). Therefore, it has to be handled in dedicated facilities with appropriate equipment for radioactive materials to avoid health risks caused by radiation exposure. Due to these precautions, extraction behavior of Am(III) was studied separately from other nonradioactive surrogates of HLLW components, and performed in a dedicated glovebox at the authorized control area of IRE, HZDR. Am(III) distribution in the biphasic system of Figure 3 and Table 1 was determined by γ-ray spectrometry using a HP-Ge detector, where initial radioactivity of ^241^Am stock solution of 3.0 M HNO_3_(aq) was 132 kBq·mL^–1^.

### Synthesis and Characterization of Metal Complexes with DRPDA

#### [Pd(DiPPDA^–^)(NO_3_)] from Organic Phase

A 3 M HNO_3_(aq) of Pd(NO_3_)_2_ (50 mM) was contacted with a CH_2_Cl_2_ phase of DiPPDA (10 mM) in 1:1 v/v, followed by shaking for 10 min at 1800 rpm. After centrifugation, concentrating the separated organic phase by slow evaporation allowed to give brown crystals of [Pd(DiPPDA^–^)(NO_3_)]. Crystallographic data (CCDC 2355904). C_13_H_18_N_4_O_5_Pd, *F*_w_ = 416.71, 0.214 × 0.114 × 0.054 mm^3^, monoclinic, *C*2/*c* (#15), *a* = 15.8620(4) Å, *b* = 10.9511(3) Å, *c* = 19.0769(5) Å, β = 105.888(3)°, *V* = 3187.19(15) Å^3^, *Z* = 8, *T* = 123 K, *D*_calcd_ = 1.737 g·cm^–3^, μ = 1.195 mm^–1^, GOF = 1.041, *R* (*I* > 2σ) = 0.0239, *wR* (all) = 0.0571. Anal. Calcd. for C_13_H_18_N_4_O_5_Pd: C, 37.47; H, 4.35; N 13.44. Found: C, 37.38; H, 4.30; N 13.21.

#### [Pd(DiPPDA^–^)(NO_3_)] from Aqueous-MeOH System

A 0.5 M HNO_3_(aq) of Pd(NO_3_)_2_ (9 mM) was mixed with a MeOH solution of DiPPDA (9 mM) in 1:1 v/v, followed by slow evaporation of solvents to afford brown crystals of [Pd(DiPPDA^–^)(NO_3_)]_3_. Crystallographic data (CCDC 2355905). C_13_H_18_N_4_O_5_Pd, *F*_w_ = 416.71, 0.382 × 0.277 × 0.156 mm^3^, monoclinic, *C*2/*c* (#15), *a* = 15.9825(8) Å, *b* = 11.0138(6) Å, *c* = 19.1210(9) Å, β = 104.794(5)°, *V* = 3254.3(3) Å^3^, *Z* = 8, *T* = 293 K, *D*_calcd_ = 1.701 g·cm^–3^, μ = 1.171 mm^–1^, GOF = 1.079, *R* (*I* > 2σ) = 0.0269, *wR* (all) = 0.0711. Anal. Calcd. for C_13_H_18_N_4_O_5_Pd: C, 37.47; H, 4.35; N, 13.44. Found: C, 37.38; H, 4.38; N, 13.25.

#### [Zr(DCyPDA)_3_](NO_3_)_4_·5H_2_O

A 3 M HNO_3_(aq) of 50 mM ZrO(NO_3_)_2_·2H_2_O was contacted with a CH_2_Cl_2_ phase of DCyPDA (20 mM) in 1:1 v/v, followed by shaking for 2 h at 1800 rpm. Colorless precipitate deposited in this mixture was separated from supernatants by centrifugation and decantation. The solid residue was washed with CH_2_Cl_2_ and 3 M HNO_3_(aq), dissolved in MeOH, and filtered. The clear filtrate (800 μL) mixed with H_2_O (200 μL) was concentrated by slow evaporation to give colorless crystals of [Zr(DCyPDA)_3_](NO_3_)_4_·5H_2_O. Crystallographic data (CCDC 2355906). C_57_H_81_N_13_O_24_Zr, *F*_w_ = 1431.63, 0.273 × 0.199 × 0.136 mm^3^, monoclinic, *C*2/*c* (#15), *a* = 12.7625(11) Å, *b* = 20.4547(17) Å, *c* = 27.397(2) Å, β = 98.515(8)°, *V* = 7073.3(11) Å^3^, *Z* = 4, *T* = 93 K, *D*_calcd_ = 1.344 g·cm^–3^, μ = 0.238 mm^–1^, GOF = 1.038, *R* (*I* > 2σ) = 0.0770, *wR* (all) = 0.2061. In this crystal structure, no NO_3_^–^ ions have been modeled as discrete atomic sites despite its sole possibility as counteranions of [Zr(DCyPDA)_3_]^4+^ available in the mother liquor. This is most probably due to disorder of them in the crystal lattice. To resolve this issue, contribution of electron densities arising from 4 NO_3_^–^ plus 4 missing H_2_O molecules was removed using solvent mask function on Olex2,^62^ thereby producing a set of diffraction intensities arising from found molecular structures used for improving the structure refinements. After this treatment, 684 e^–^ in 1832 Å^3^ void were found in a crystal lattice. The number of e^–^ reasonably agree with that of the missing NO_3_^–^ and H_2_O with *Z* = 4 resulting 672 e^–^/cell. Elemental analysis for this compound was omitted, because only several tiny crystals of this compound deposited.

## Quantum Chemical Calculations

Quantum chemical calculations were performed using the Gaussian 16 program (Gaussian Inc.) rev.C01^67^ employing density functional theory (DFT) by using a conductor like polarizable continuum model. Structure optimizations were performed at the B3LYP level followed by vibrational frequency analysis at the same level to confirm that no imaginary frequency is present. Dunning’s correlation consistent triple-ζ basis set was employed on all elements except for Pd for which SDD effective core potential (ECP) and corresponding basis set has been applied. The spin–orbit effects and basis set superposition error corrections were neglected. NBO analysis was performed using NBO 7.0.10.^68^ All the calculations were performed on a TSUBAME 4.0 supercomputing system at Institute of Science Tokyo.

## Supporting Information Available

Extraction behavior of HLLW components in different biphasic systems, optimized molecular structures and natural bond orbitals of Pd(II)-DiPPDA complexes obtained from DFT calculations, NMR spectra of extracted Pd(II) species with DHdPDA, hydrolytic species distribution of Zr(IV) at different pH in aqueous system, list of starting materials of M’s, and CSD structure search results for different coordination mode of DRPDAs (PDF)
