# Supplementary material for: Utility of Interchangeable Coordination Modes of N,N′-Dialkyl-2,6-pyridinediamide Tridentate Pincer Ligands for Solvent Extraction of Pd(II) and Zr(IV) from High-Level Radioactive Liquid Waste
Source: Inorg Chem. 2024 Dec 10;63(52):24647–61. doi: 10.1021/acs.inorgchem.4c03844 (PMC11688663; doi:10.1021/acs.inorgchem.4c03844)

Supporting Information

Utility of Interchangeable Coordination Modes of *N,N'*-Dialkyl-2,6-Pyridinediamide Tridentate Pincer-Ligands for Solvent Extraction of Pd(II) and Zr(IV) from High-level Radioactive Liquid Waste

Tasuku Orino,[1] Yueming Cao,[1] Ririka Tashiro,[1] Tomoyuki Takeyama,[1,2] Robert Gericke,[3] Satoru Tsushima,[1,3] Koichiro Takao[1]*

[1]Laboratory for Zero-Carbon Energy, Institute of Integrated Research, Institute of Science Tokyo, 2-12-1 N1-32, O-okayama, Meguro-ku, 152-8550 Tokyo, Japan.

[2]Department of Applied Chemistry, Sanyo-Onoda City University, 1-1-1, Daigakudori, Sanyo-Onoda, Yamaguchi 756-0884, Japan.

[3]Institute of Resource Ecology, Helmholtz-Zentrum Dresden-Rossendorf (HZDR), Bautzner Landstrasse 400, 01328 Dresden, Germany.

*Correspondence should be addressed. E-mail: ktakao@zc.iir.isct.ac.jp

Table of Contents.

		page
**Figure S1.**	Extraction kinetics of 5 mM Pd(II) from 3.0 M HNO_3(aq) to 1-octanol containing 30 mM DRPDA at room temperature.	S4
**Figure S2.**	Extractability (E%) of Se(IV), Mo(VI), and Re(VII) (0.1 mM each) from 3.0 M HNO_3(aq) to 1-octanol without DRPDA after 10 min shaking at room temperature.	S4
**Figure S3.**	Extractability (E%) of HLLW components (M, 0.1 mM each) from 3.0 M HNO_3(aq) to different organic phases after 60 min shaking at room temperature.	S5
**Figure S4.**	Extraction kinetics of 1 mM Pd(II) from 3.0 M HNO_3(aq) to *n*-dodecane containing 5 mM DEhPDA and 0-20 vol% 1-octanol.	S5
**Figure S5.**	Extractability (E%) of HLLW components (M, 0.1 mM each) from 3.0 M HNO_3(aq) to *n*-dodecane phase containing 0-20 vol% 1-octanol after 60 min shaking at room temperature.	S6
**Figure S6.**	Extraction kinetics of 1 mM Pd(II) from 3.0 M HNO_3(aq) to CH_2Cl_2 containing 30 mM DiPPDA or DHPDA.	S6
**Figure S7.**	Acidity in the CH_2Cl_2 phase with or without 30 mM DiPPDA after contacting an aqueous phase with different [HNO_3].	S7
**Figure S8.**	Distribution ratio of Pd(II) (D_{Pd}) from HNO_3(aq) to CH_2Cl_2 under different [DiPPDA] conditions.	S7
**Figure S9.**	E% of Pd(II) from 3.0 M HNO_3(aq) to 1-octanol at different [DHPDA]/[Pd(II)].	S8
**Figure S10.**	Molecular structure of [Pd(DiPPDA$^-$)(NO_3)] optimized by the DFT calculation. Bond distances are in green ink.	S9
**Figure S11.**	Natural bond orbitals of [Pd(DiPPDA$^-$)(NO_3)].	S9
**Figure S12.**	Molecular structure of another tautomer of [Pd(DiPPDA$^-$)(NO_3)] with O$^-$^N^O coordinating DiPPDA$^-$ optimized by the DFT calculation. Bond distances are in green ink.	S9
**Figure 13.**	^{1}H NMR spectra of *n*-dodecane + 20 vol% 1-octanol phase containing 20 mM DHdPDA (a) and that after extraction of 20 mM Pd(II) from 3.0 M HNO_3(aq) (b) together with chemical exchange between N$^-$^N^O and O^N^N$^-$ coordination modes in [Pd(DHdPDA$^-$)(NO_3)] (c).	S10
**Figure S14.**	Distribution ratio of Zr(IV) (D_{Zr}) from HNO_3(aq) to *n*-dodecane containing 20 mM DHdPDA and 20 vol% 1-octanol at different [HNO_3].	S11
**Figure S15.**	Speciation diagrams of Zr(IV) in aqueous solution at I = 3.0 M, 298 K, and different pH.	S12

**Figure S16.**	Extraction behavior of Pd(II) and Zr(IV) from 3.0 M HNO_3(aq) to the recycled organic phases (a) as received after stripping of Pd(II) with 1.0 M HCl(aq) and (b) scrubbed with 3.0 M HNO_3(aq) prior to reuse.	S13
**Table S1.**	Starting Materials of HLLW Components Studied Here Except for Am	S14
**Table S2**	Selected Structural Parameters of [Pd(DiPPDA$^-$)(NO_3)] of Figure 4(c)	S15
**CSD Search**	O^N^O Coordination	S16
	N$^-$^N^O Coordination	S31
	O$^-$^N^O Coordination	S33
	N$^-$^N^N$^-$ Coordination	S35
	Pd(II) Complexes with N$^-$^N^N$^-$ Coordination	S110
	N$^-$^N^O$^-$ Coordination	S124
	O$^-$^N^O$^-$ Coordination	S126

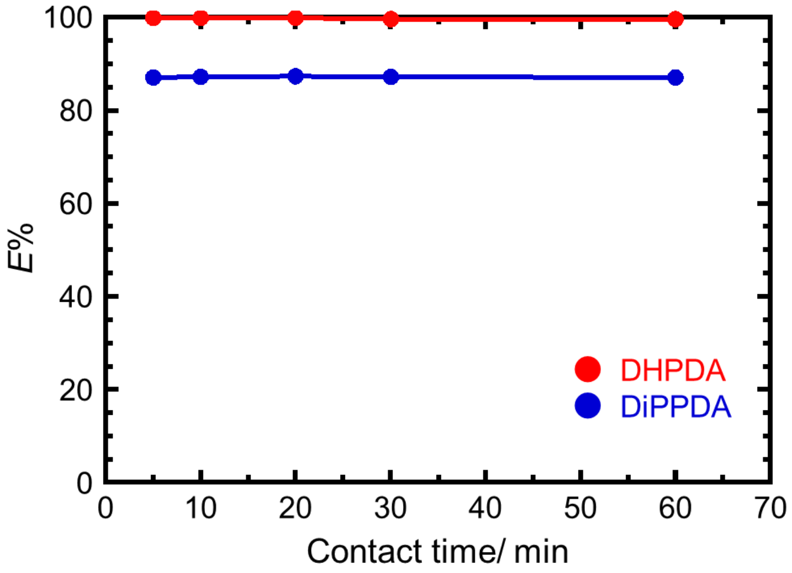

Figure S1. Extraction kinetics of 5 mM Pd(II) from 3.0 M HNO_3(aq) to 1-octanol containing 30 mM DRPDA at room temperature. Each run has been repeated three times, and averaged.

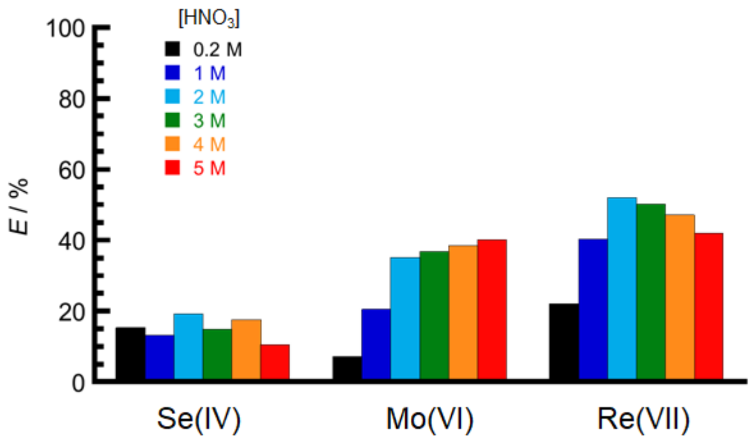

Figure S2. Extractability (*E*%) of Se(IV), Mo(VI), and Re(VII) (0.1 mM each) from 3.0 M HNO_3(aq) to 1-octanol without DRPDA after 10 min shaking at room temperature.

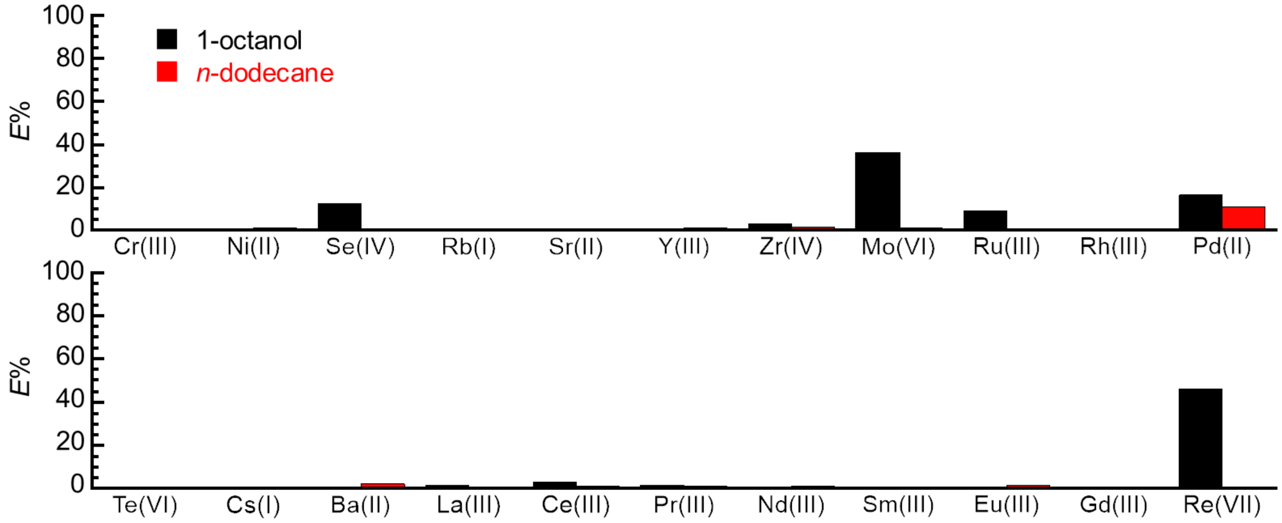

Figure S3. Extractability (*E*%) of HLLW components (M, 0.1 mM each) from 3.0 M HNO_3(aq) to different organic phases (black: 1-octanol, red: *n*-dodecane) after 60 min shaking at room temperature.

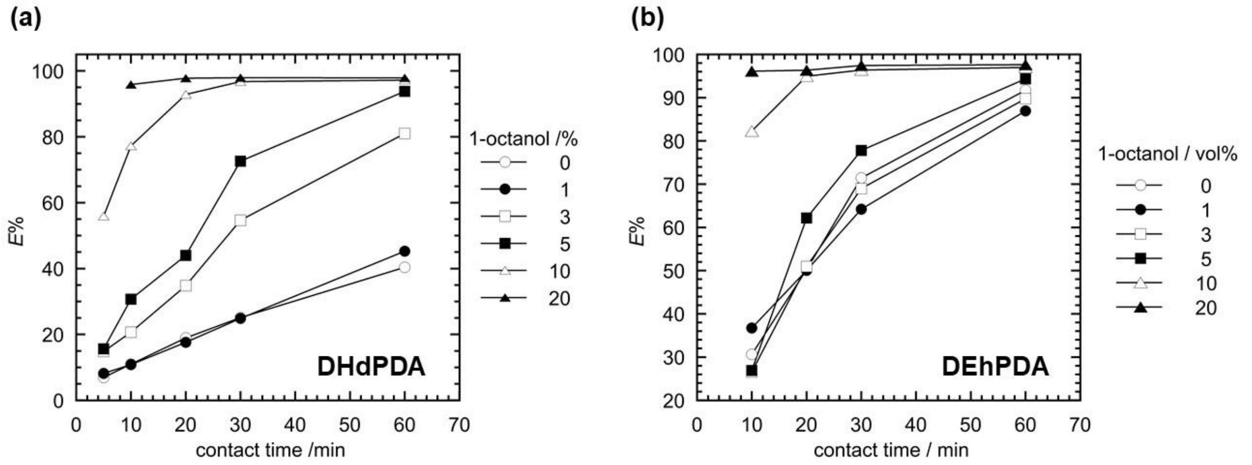

Figure S4. Extraction kinetics of 1 mM Pd(II) from 3.0 M HNO_3(aq) to *n*-dodecane containing 5 mM DHdPDA (a) or DEhPDA (b) and 0-20 vol% 1-octanol. Each run has been repeated three times, and averaged.

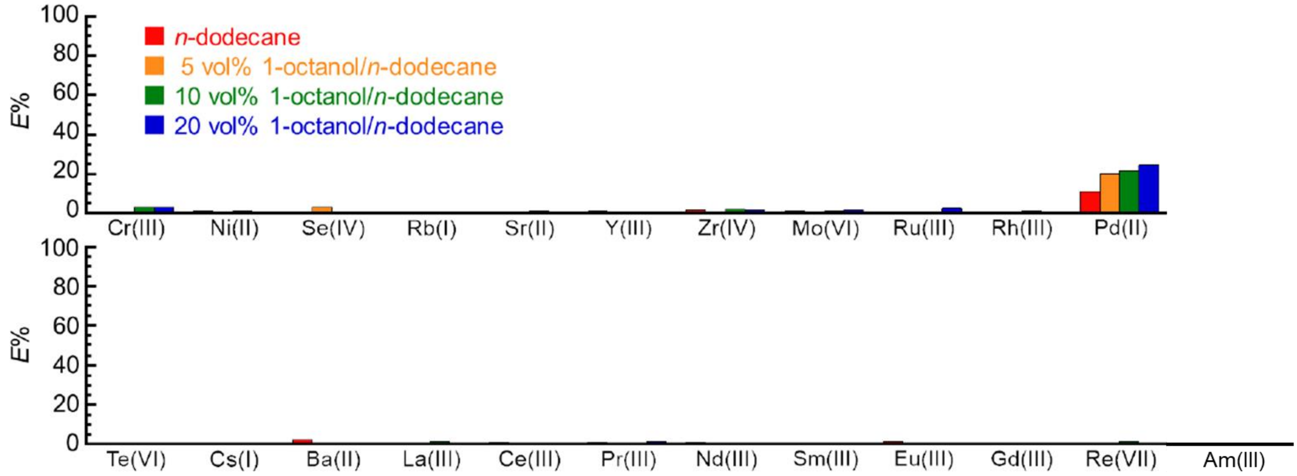

Figure S5. Extractability (*E*%) of HLLW components (M, 0.1 mM each) from 3.0 M HNO_3(aq) to *n*-dodecane phase containing 0-20 vol% 1-octanol after 60 min shaking at room temperature. ^{241}Am(III) was studied only in 20 vol% 1-octanol/*n*-dodecane system due to its radioactivity, and trace-level experiments have been performed separately from other HLLW components.

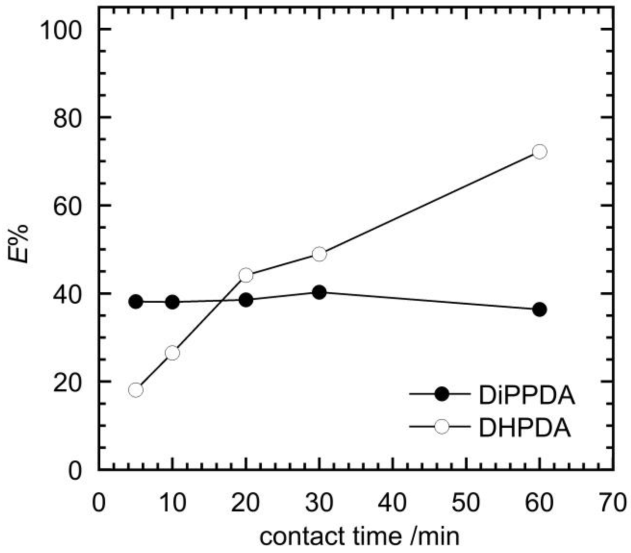

Figure S6. Extraction kinetics of 1 mM Pd(II) from 3.0 M HNO_3(aq) to CH_2Cl_2 containing 30 mM DiPPDA or DHPDA. Each run has been repeated three times, and averaged.

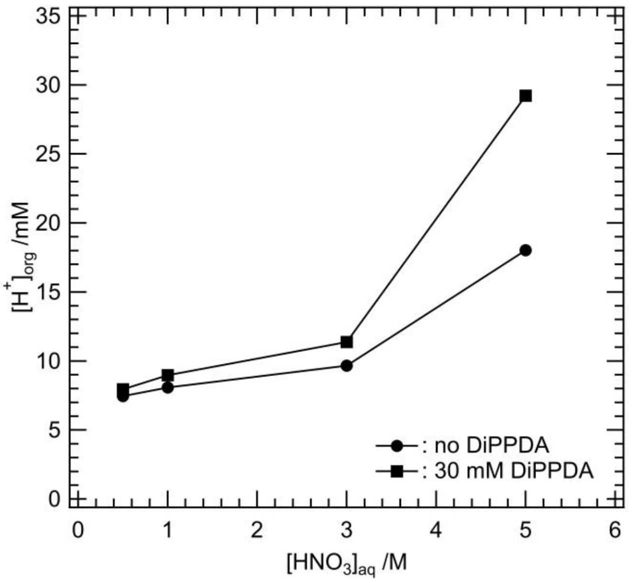

Figure S7. Acidity in the CH_2Cl_2 phase ($[H^+]_{org}$) with or without 30 mM DiPPDA after contacting an aqueous phase with different $[HNO_3]$.

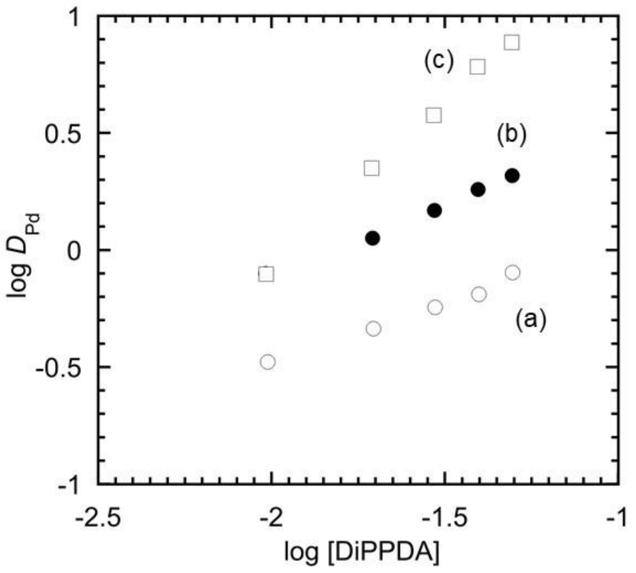

Figure S8. Distribution ratio of Pd(II) (D_{Pd}) from HNO_3(aq) to CH_2Cl_2 under different [DiPPDA] conditions at (a) $[HNO_3]$ = 3.0 M, (b) $[HNO_3]$ = 3.0 M + $[NaNO_3]$ = 2.0 M, and (c) $[HNO_3]$ = 1.0 M + $[NaNO_3]$ = 4.0 M.

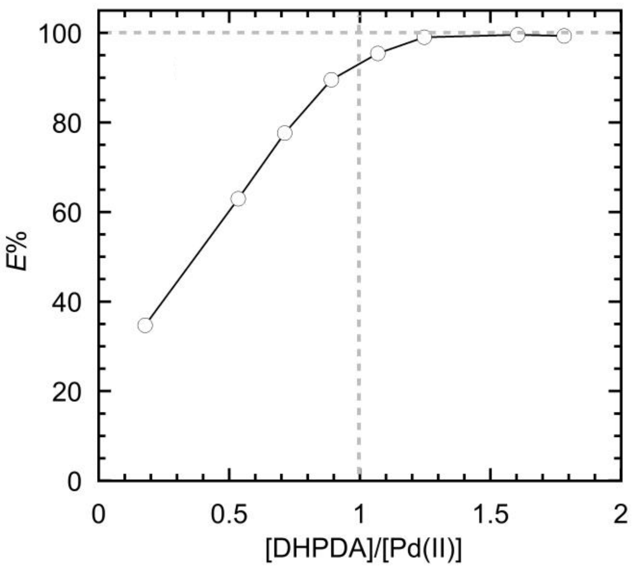

Figure S9. *E*% of Pd(II) from 3.0 M HNO_3(aq) to 1-octanol at different [DHPDA]/[Pd(II)]. $[Pd(II)]_{aq,init}$ = 5.6 mM, [DHPDA] = 1-10 mM,

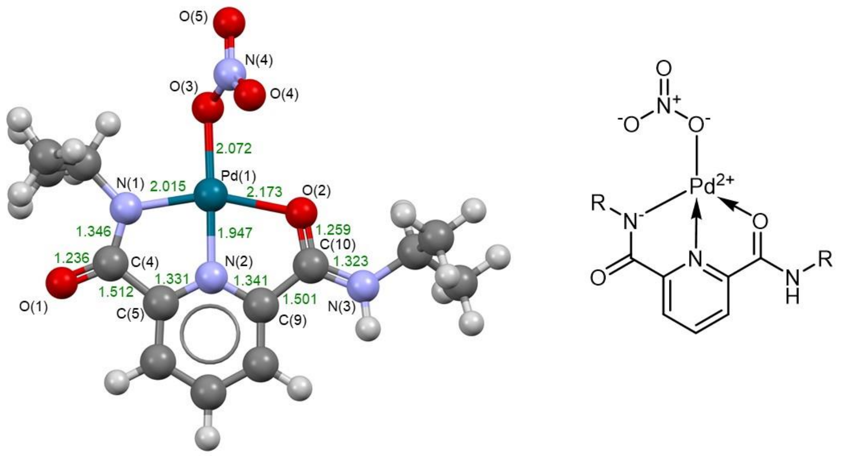

Figure S10. Molecular structure of [Pd(DiPPDA⁻)(NO_3)] optimized by the DFT calculation. Bond distances are in green ink.

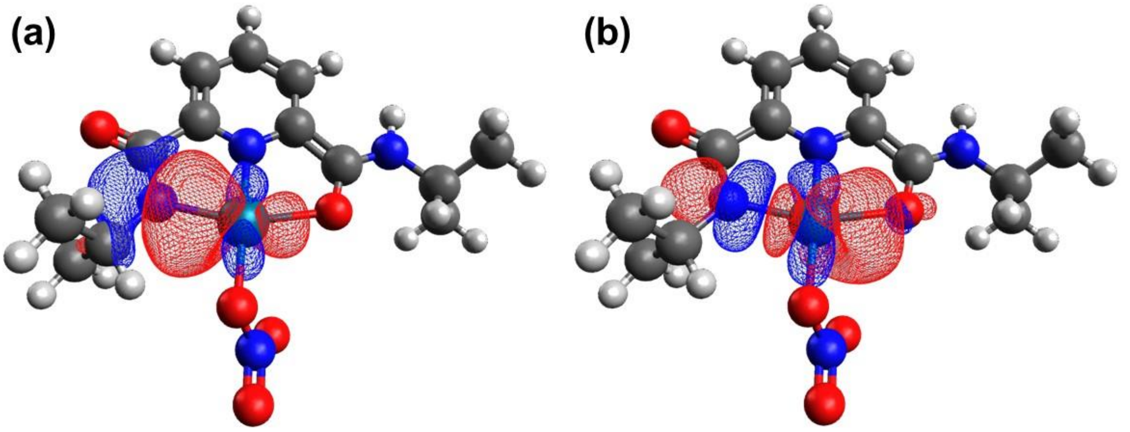

Figure S11. Natural bond orbitals of [Pd(DiPPDA⁻)(NO_3)]. (a) Pd−N bonding, (b) Pd−N antibonding.

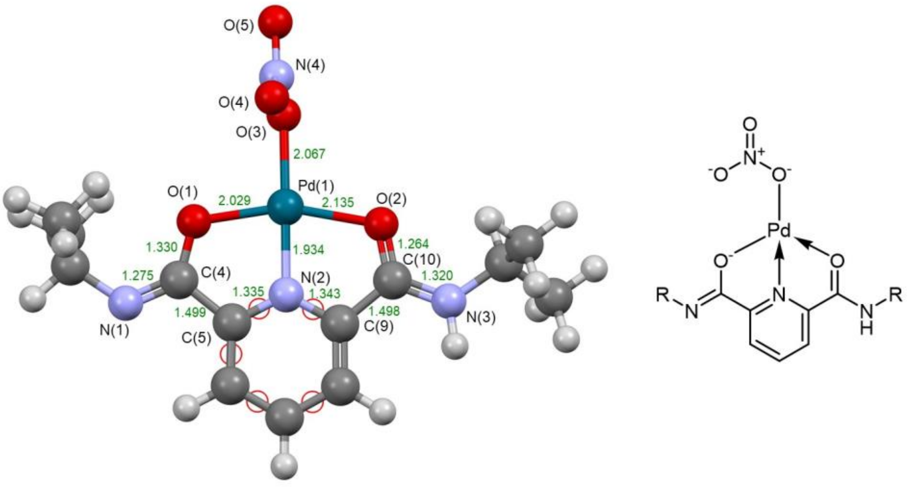

Figure S12. Molecular structure of another tautomer of [Pd(DiPPDA⁻)(NO_3)] with O⁻^N^O coordinating DiPPDA⁻ optimized by the DFT calculation. Bond distances are in green ink.

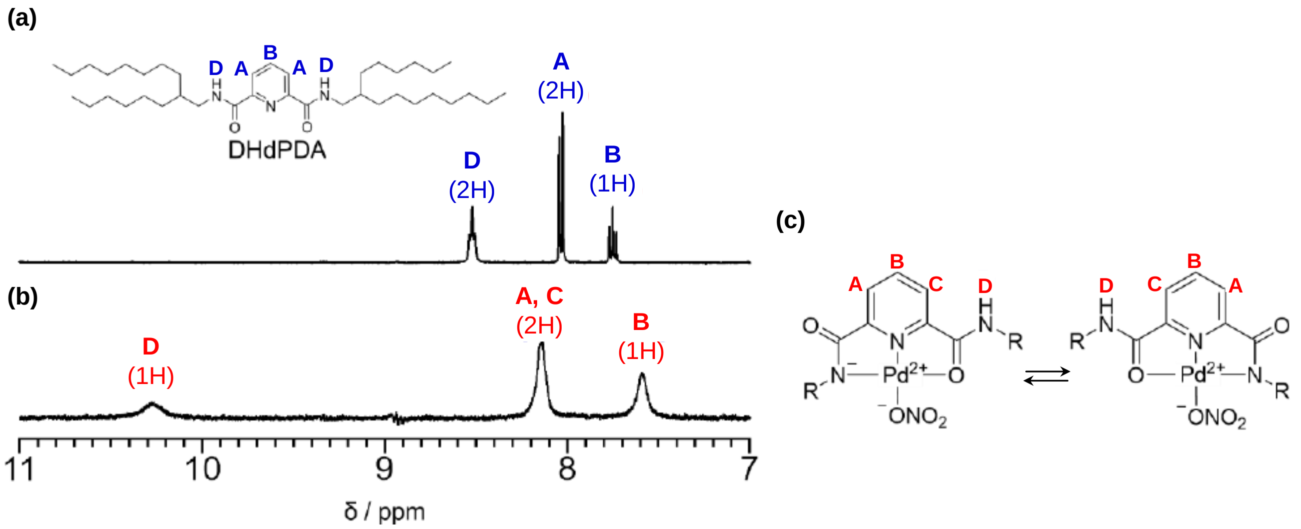

Figure S13. ^{1}H NMR spectra of *n*-dodecane + 20 vol% 1-octanol phase containing 20 mM DHdPDA (a) and that after extraction of 20 mM Pd(II) from 3.0 M HNO_3(aq) (b) together with chemical exchange between N^-^N^O and O^N^N^- coordination modes in [Pd(DHdPDA$^-$)(NO_3)] (c).

Additional Comments for Figures 4(d) and S13:

NMR peak positions of the coordinated DHdPDA in Figure S13 (red, H_A, H_C: 8.1-8.2 ppm, H_B: 7.59 ppm, H_D: 10.28 ppm) are different from those of DiPPDA in Figure 4(d) (red, H_A, H_C: 7.90-7.93 ppm, H_B: 8.20 ppm, H_D: 8.28 ppm). Note that different DRPDAs were used in these NMR experiments. As a matter of fact, such difference in chemical shifts have already been found even in the free forms of these ligands; H_A: 8.30 ppm, H_B: 8.09 ppm, H_D: 7.46 ppm for free DiPPDA in CD_2Cl_2 of Figure 4(d) (blue) *vs.* H_A: 8.03 ppm, H_B: 7.77 ppm, H_D: 8.52 ppm for DHdPDA in *n*-dodecane + 20 vol% 1-octanol of Figure S13 (blue). While we do not know why such differences appeared, it is not very unusual that different compounds exhibit different NMR chemical shifts.

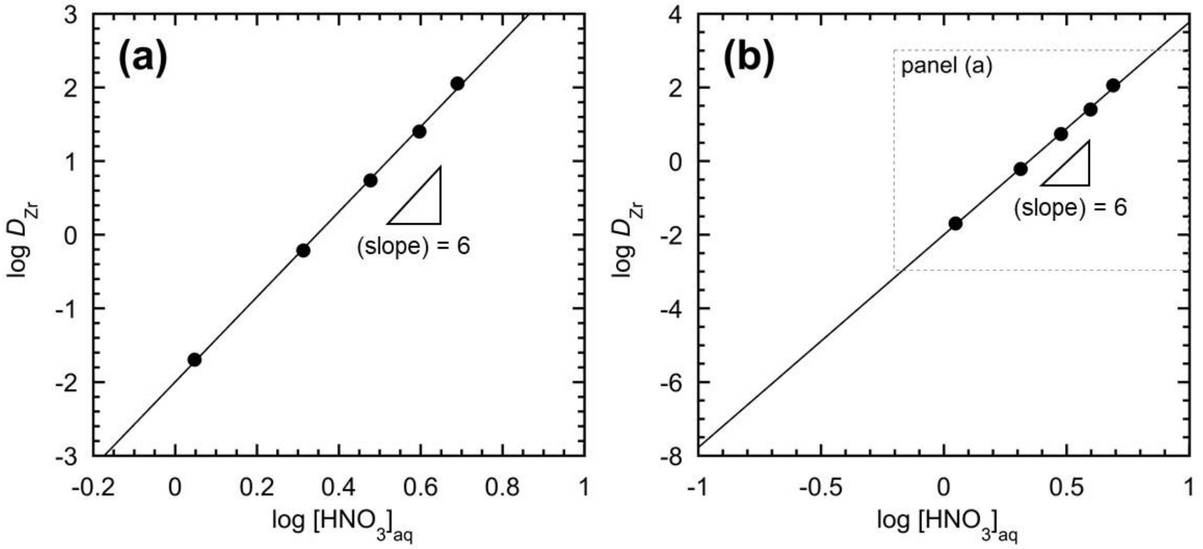

Figure S14. Distribution ratio of Zr(IV) (D_{Zr}) from HNO_3(aq) to *n*-dodecane containing 20 mM DHdPDA and 20 vol% 1-octanol at different $[HNO_3]$. (a) $-0.2 < \log [HNO_3]_{aq} < 1$, (b) $-1 < \log [HNO_3]_{aq} < 1$.

(a)

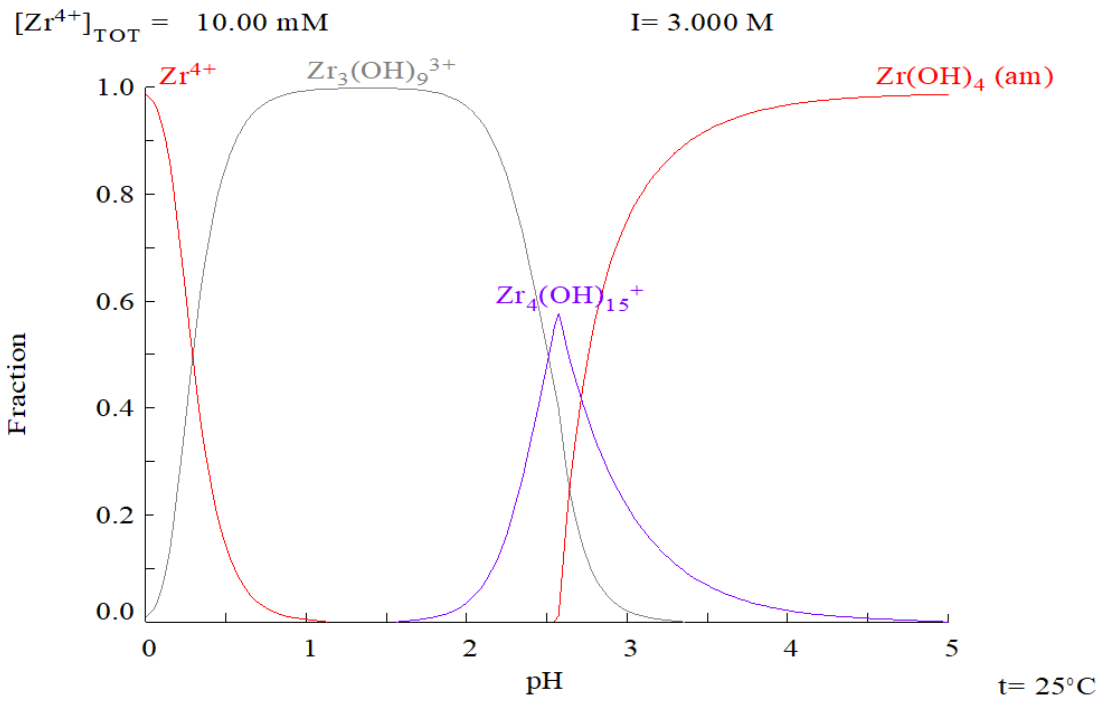

(b)

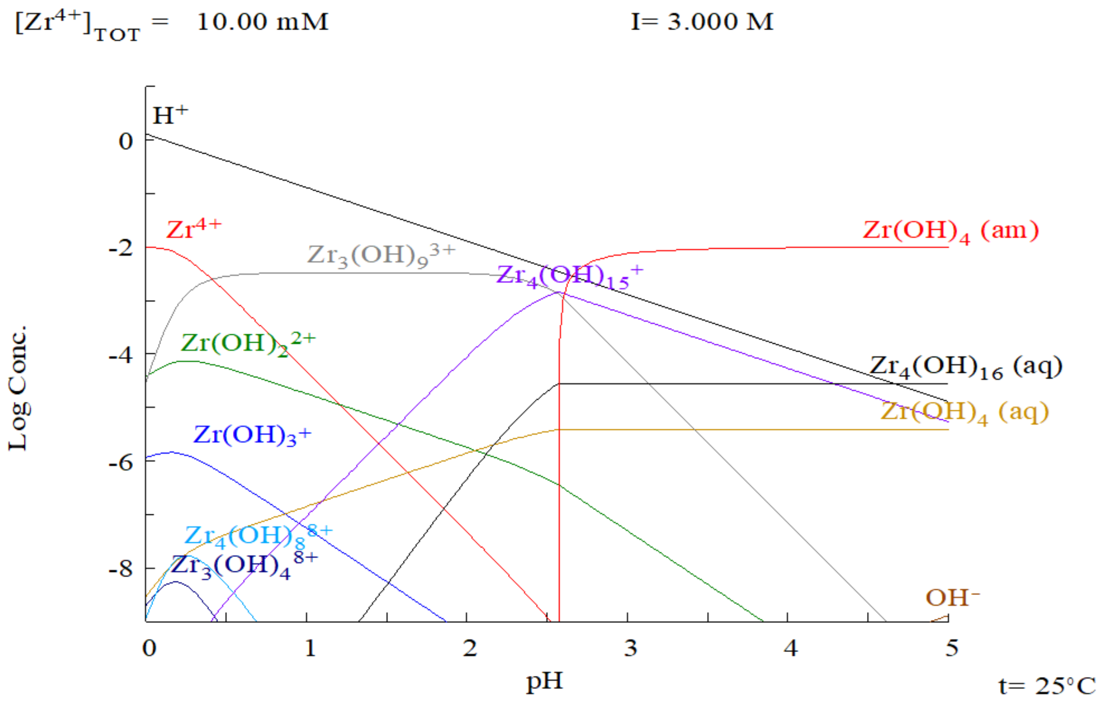

Figure S15. Speciation diagrams of Zr(IV) in aqueous solution at *I* = 3.0 M, 298 K, and different pH. (a) Mole fraction of Zr(IV), (b) logarithmic concentrations of occurring species.

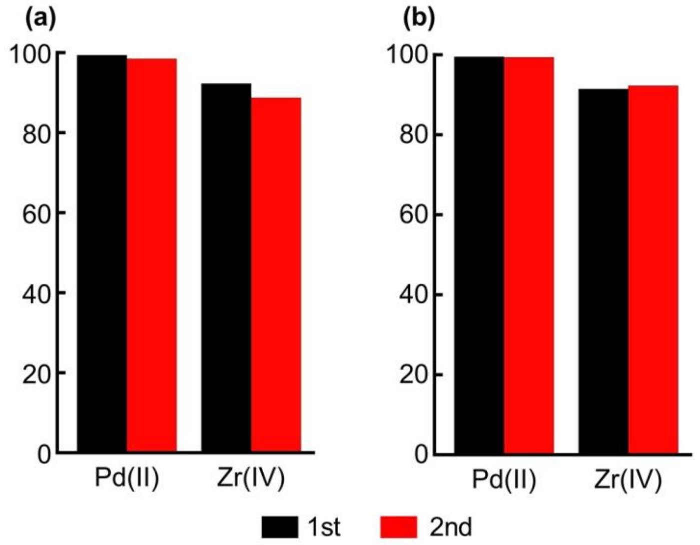

Figure S16. Extraction behavior of Pd(II) and Zr(IV) from 3.0 M HNO_3(aq) to the recycled organic phases as received after stripping of Pd(II) with 1.0 M HCl(aq) (a) and scrubbed with 3.0 M HNO_3(aq) prior to reuse (b).

Table S1. Starting Materials of HLLW Components Studied Here Except for Am

M^{n+}		M^{n+}		M^{n+}	
Cr(III)	$Cr(NO_3)_3\cdot 9H_2O$	Mo(IV)	$Na_2MoO_4\cdot 2H_2O$	Ce(III)	$Ce(NO_3)_3\cdot 6H_2O$
Fe(II)	$Fe(NO_3)_3\cdot 9H_2O$	Ru(III)	$Ru(NO)(NO_3)_3$	Pr(III)	$Pr(NO_3)_3\cdot 6H_2O$
Ni(II)	$Ni(NO_3)_3\cdot 6H_2O$	Rh(III)	$Rh(NO_3)_3$	Nd(III)	$Nd(NO_3)_3\cdot 6H_2O$
Se(VI)	Na_2SeO_3	Pd(II)	$Pd(NO_3)_2\cdot 2H_2O$	Sm(III)	$Sm(NO_3)_3\cdot 6H_2O$
Rb(I)	$RbNO_3$	Te(VI)	H_6TeO_6	Eu(III)	$Eu(NO_3)_3\cdot 6H_2O$
Sr(II)	$Sr(NO_3)_2$	Cs(I)	$CsNO_3$	Gd(III)	$Gd(NO_3)_3\cdot 6H_2O$
Y(III)	$Y(NO_3)_3\cdot 2H_2O$	Ba(II)	$Ba(NO_3)_2$	Re(VII)	$NaReO_4$
Zr(IV)	$ZrO(NO_3)_2\cdot 2H_2O$	La(III)	$La(NO_3)_3\cdot 6H_2O$		

Table S2. Selected Structural Parameters of [Pd(DiPPDA$^-$)(NO_3)] of Figure 4(c)

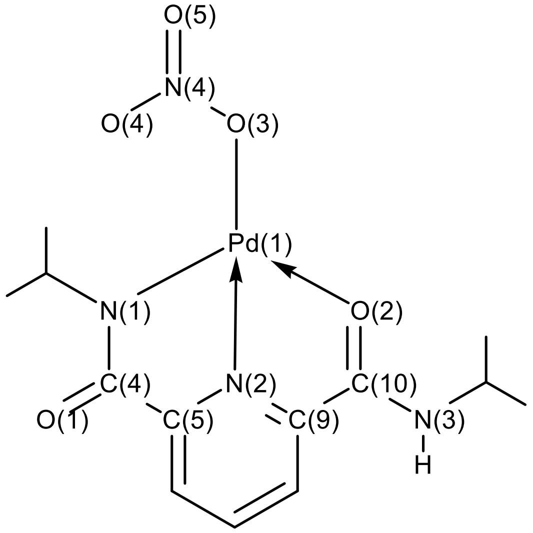

bond distances			
Pd(1)−N(1)	2.0054(14) Å	C(4)−O(1)	1.239(2) Å
Pd(1)−N(2)	1.9203(14) Å	C(4)−N(1)	1.346(2) Å
Pd(1)−O(2)	2.0937(11) Å	C(10)−O(2)	1.2643(19) Å
Pd(1)−O(3)	2.0482(12) Å	C(10)−N(3)	1.312(2) Å
bond angles			
N(1)−Pd(1)−N(2)	81.22(6)°	N(1)−C(4)−O(1)	127.77(16)°
N(2)−Pd(1)−O(2)	80.05(5)°	N(1)−C(4)−C(5)	112.70(14)°
O(2)−Pd(1)−O(3)	99.88(5)°	C(4)−C(5)−N(2)	113.49(15)°
O(3)−Pd(1)−N(1)	98.92(5)°	N(2)−C(9)−C(10)	110.71(14)°
Σ(around Pd(1))	360.07°	C(9)−C(10)−O(2)	118.59(15)°
		N(3)−C(10)−O(2)	121.89(15)°

Search Overview

Search:	search1
Date/Time done:	Mon Jul 8 09:53:10 2024
Database(s):	CSD version 5.43 updates (Mar 2022)<br>CSD version 5.43 (November 2021)<br>CSD version 5.43 updates (Sep 2022)<br>CSD version 5.43 updates (Nov 2022)
Restriction Info:	No refcode restrictions applied
Filters:	None
Percentage Completed:	100%
Number of Hits:	56

Single query used. Search found structures that:

match

Query 1

Query 1

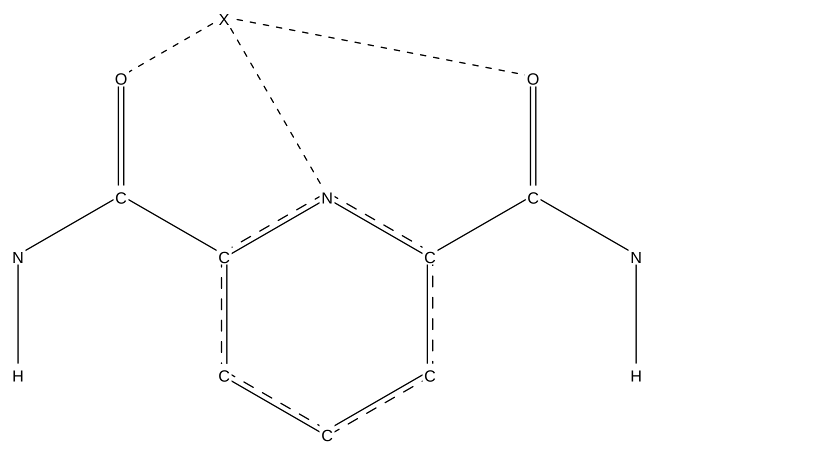

Search: search1 (Mon Jul 8 09:53:10 2024): Hits 1-4

AZIFAT

Reference: King-Him Yim, Chi-Tung Yeung, M.R.Probert, Wesley Ting Kwok Chan, L.E.Mackenzie, R.Pal, Wing-Tak Wong, G.-L.Law (2021) *Communications Chemistry* ,**4,**116

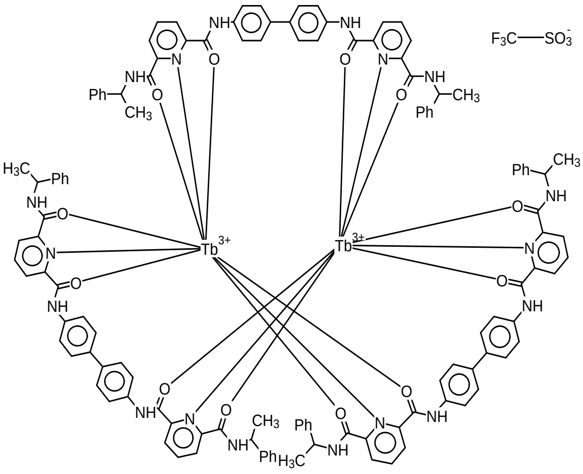

F_3C—SO_3^-

AZIJIF

Reference: King-Him Yim, Chi-Tung Yeung, M.R.Probert, Wesley Ting Kwok Chan, L.E.Mackenzie, R.Pal, Wing-Tak Wong, G.-L.Law (2021) *Communications Chemistry* ,**4,**116

$Eu_4[\mu_2\text{-}C_{22}H_{16}N_6O_4Ph_2Me_2\text{-}\kappa^2N,N',\kappa^4O,O',O'',O''']_6^{12+}$

^-O_3S—CF_3

BEPYAZ

Reference: Xiao-Zhen Li, Li-Peng Zhou, Liang-Liang Yan, Ya-Min Dong, Zhuan-Ling Bai, Xiao-Qi Sun, Juan Diwu, Shuao Wang, J.-C.Bunzli, Qing-Fu Sun (2018) *Nat.Commun.* ,**9,**547

$La_4[\mu_3\text{-}C_{48}H_{33}N_9O_6Ph_3Me_3\text{-}\kappa^3N,N',N'',\kappa^6O,O',O'',O''',O'''',O''''']_4^{12+}$

ClO_4^-

H_2O

BEPYED

Reference: Xiao-Zhen Li, Li-Peng Zhou, Liang-Liang Yan, Ya-Min Dong, Zhuan-Ling Bai, Xiao-Qi Sun, Juan Diwu, Shuao Wang, J.-C.Bunzli, Qing-Fu Sun (2018) *Nat.Commun.* ,**9,**547

$Cd_4[\mu_3\text{-}C_{48}H_{33}N_9O_6Ph_3Me_3\text{-}\kappa^3N,N',N'',\kappa^6O,O',O'',O''',O'''',O''''']_4^{8+}$

H_2O

ClO_4^-

Search: search1 (Mon Jul 8 09:53:10 2024): Hits 5-8

BIGFIJ

Reference: Shi-Xia Luo, Xiao-Jing Chen, Man-Qi Gu, Bi-Xue Zhu, Huai-Wu Zhu, Gang Wei (2015) *Huaxue Shiji(Chin.)(Chemical Reagents)* ,**37,**692

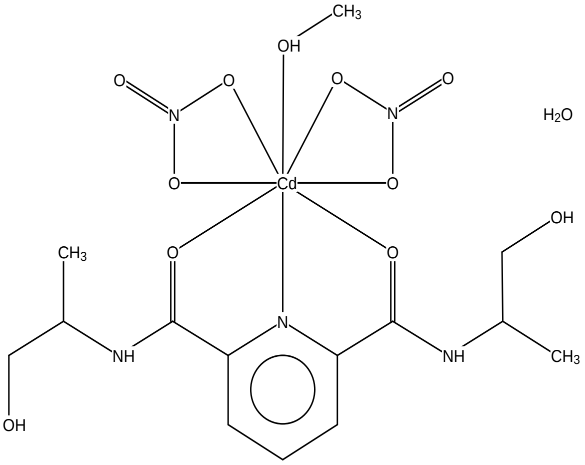

H_2O

BISGIV

Reference: O.Kotova, J.A.Kitchen, C.Lincheneau, R.D.Peacock, T.Gunnlaugsson (2013) *Chem.-Eur.J.* ,**19,**16181

$Eu[C_{29}H_{21}N_3O_2Me_2\text{-}\kappa^1N,\kappa^2O,O']_3^{3+}$

ClO_4^-

HO—CH3

H_2O

BISGOB

Reference: O.Kotova, J.A.Kitchen, C.Lincheneau, R.D.Peacock, T.Gunnlaugsson (2013) *Chem.-Eur.J.* ,**19,**16181

$Eu[C_{29}H_{21}N_3O_2Me_2\text{-}\kappa^1N,\kappa^2O,O']_3^{3+}$

ClO_4^-

HO—CH3

H_2O

DEHWAR

Reference: A.Brown, K.M.Mennie, O.Mason, N.G.White, P.D.Beer (2017) *Dalton Trans.* ,**46,**13376

ClO_4^-

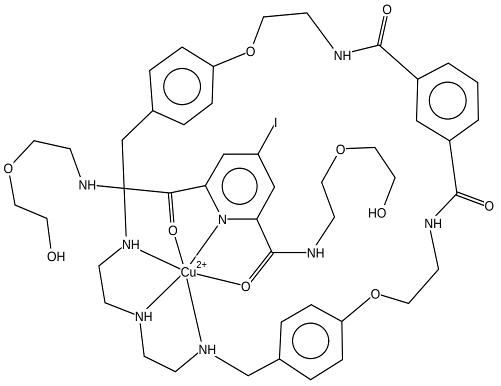

Search: search1 (Mon Jul 8 09:53:10 2024): Hits 9-12

DORKAY

Reference: J.-F.Ayme, G.Gil-Ramirez, D.A.Leigh, J.-F.Lemonnier, A.Markevicius, C.A.Muryn, Gen Zhang (2014) *J.Am.Chem.Soc.* ,**136,** 13142

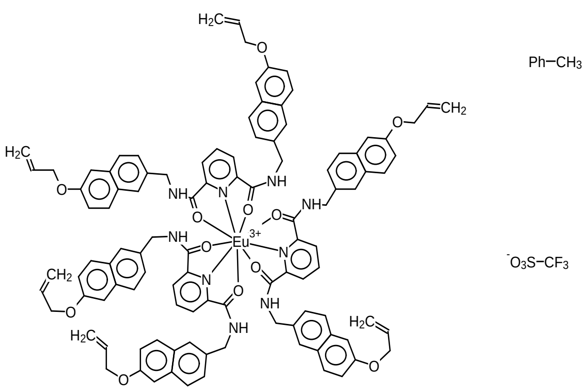

EHIHOV

Reference: Yiwei Song, F.Schaufelberger, Z.Ashbridge, Lucian Pirvu, I.J.Vitorica-Yrezabal, D.A.Leigh (2021) *Chemical Science* ,**12,**1826

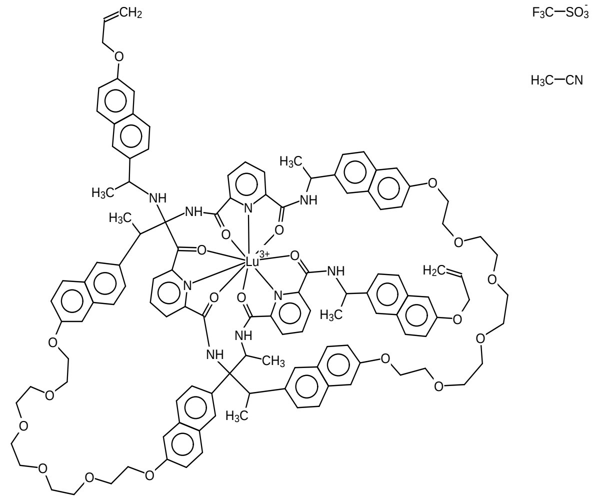

EQANOB

Reference: Jie Zhang, Shaofeng Liu, Antai Li, Hongqi Ye, Zhibo Li (2016) *New J.Chem.* ,**40,**7027

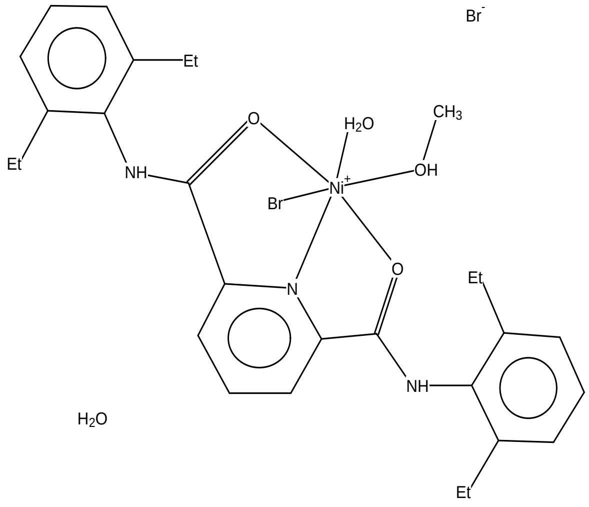

EQAQAQ

Reference: Jie Zhang, Shaofeng Liu, Antai Li, Hongqi Ye, Zhibo Li (2016) *New J.Chem.* ,**40,**7027

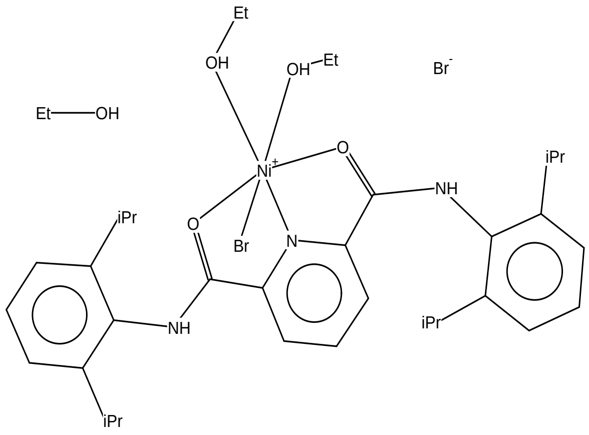

Search: search1 (Mon Jul 8 09:53:10 2024): Hits 13-16

FALLIR

Reference: Xiao-Lu Hu, Xuan-Di Tang, Yuan-Yuan Wang, Tian-Tian Huang, Qi Yue, En-Qing Gao (2020) *J.Solid State Chem.* , **294,**121871

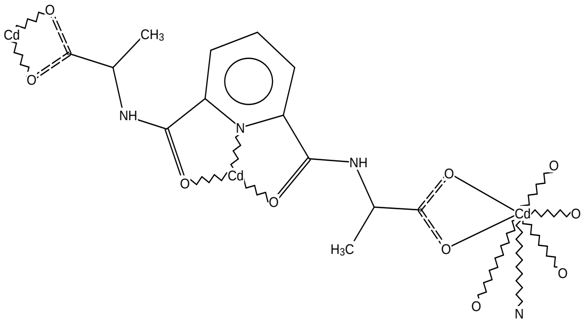

FEKYIE

Reference: M.P.Brandi-Blanco, D.Choquesillo-Lazarte, C.G.Garcia-Collado, J.M.Gonzalez-Perez, A.Castineiras, J.Niclos-Gutierrez (2005) *Inorg.Chem.Commun.* ,**8,**231

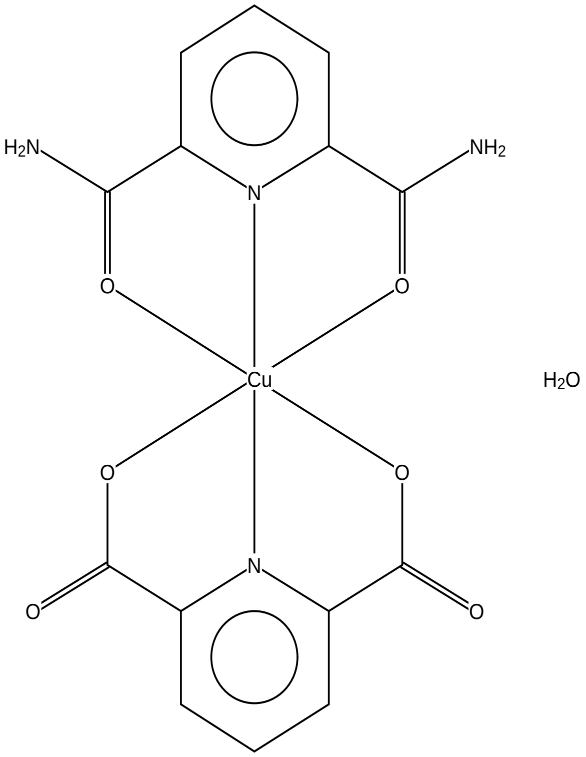

HAKPUH

Reference: G.Gil-Ramirez, S.Hoekman, M.O.Kitching, D.A.Leigh, I.J.Vitorica-Yrezabal, Gen Zhang (2016) *J.Am.Chem.Soc.* ,**138,**13159

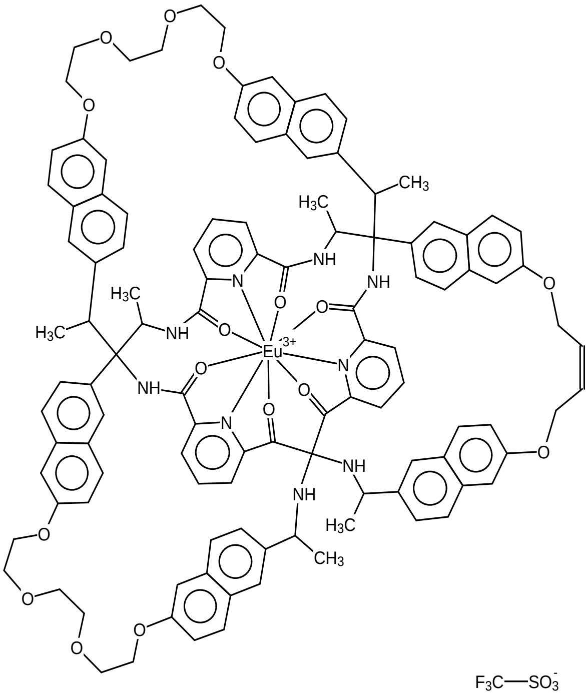

HAWPOM

Reference: K.T.Hua, Jide Xu, E.E.Quiroz, S.Lopez, A.J.Ingram, V.A.Johnson, A.R.Tisch, A.de Bettencourt-Dias, D.A.Straus, G.Muller (2012) *Inorg.Chem.* ,**51,**647

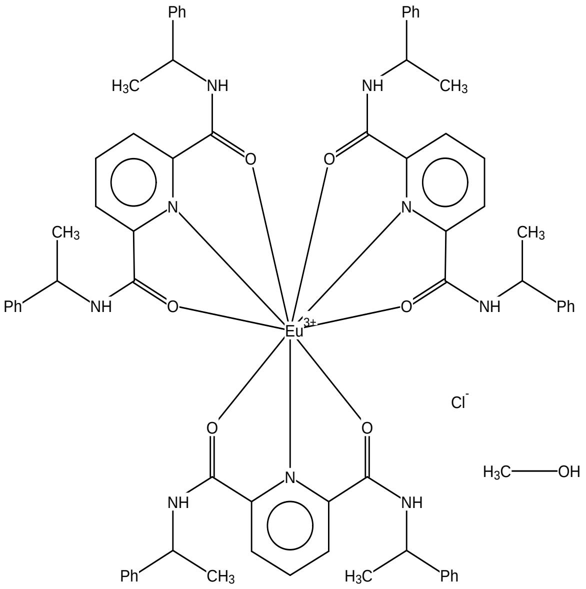

Search: search1 (Mon Jul 8 09:53:10 2024): Hits 17-20

HAWPUS

Reference: K.T.Hua, Jide Xu, E.E.Quiroz, S.Lopez, A.J.Ingram, V.A.Johnson, A.R.Tisch, A.de Bettencourt-Dias, D.A.Straus, G.Muller (2012) *Inorg.Chem.* ,**51,**647

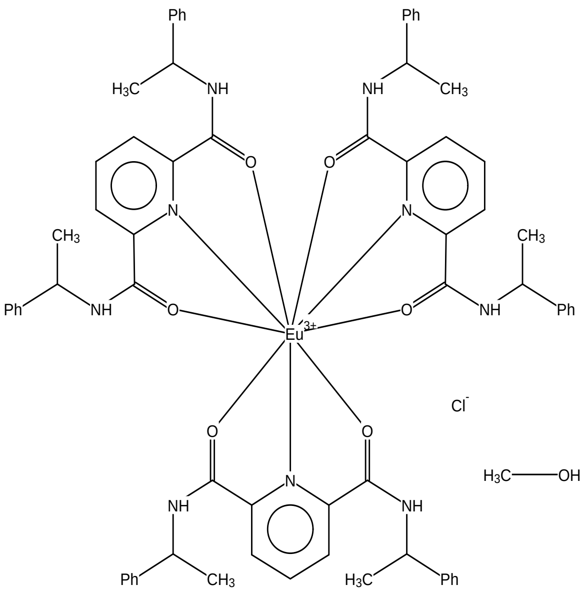

HAWQAZ

Reference: K.T.Hua, Jide Xu, E.E.Quiroz, S.Lopez, A.J.Ingram, V.A.Johnson, A.R.Tisch, A.de Bettencourt-Dias, D.A.Straus, G.Muller (2012) *Inorg.Chem.* ,**51,**647

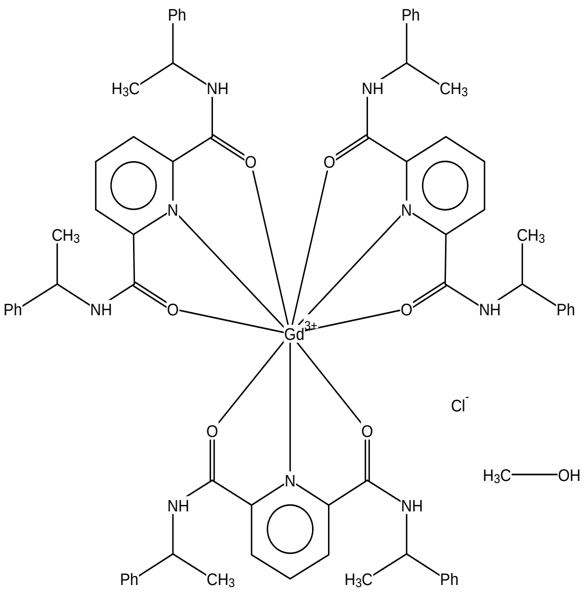

HAWQED

Reference: K.T.Hua, Jide Xu, E.E.Quiroz, S.Lopez, A.J.Ingram, V.A.Johnson, A.R.Tisch, A.de Bettencourt-Dias, D.A.Straus, G.Muller (2012) *Inorg.Chem.* ,**51,**647

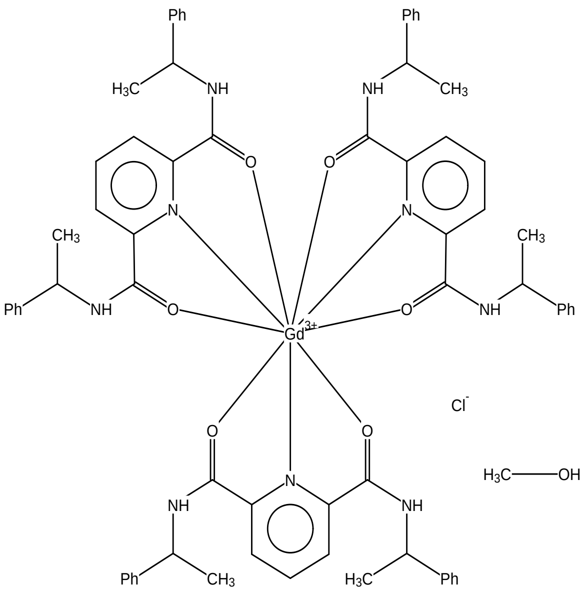

HAWQIH

Reference: K.T.Hua, Jide Xu, E.E.Quiroz, S.Lopez, A.J.Ingram, V.A.Johnson, A.R.Tisch, A.de Bettencourt-Dias, D.A.Straus, G.Muller (2012) *Inorg.Chem.* ,**51,**647

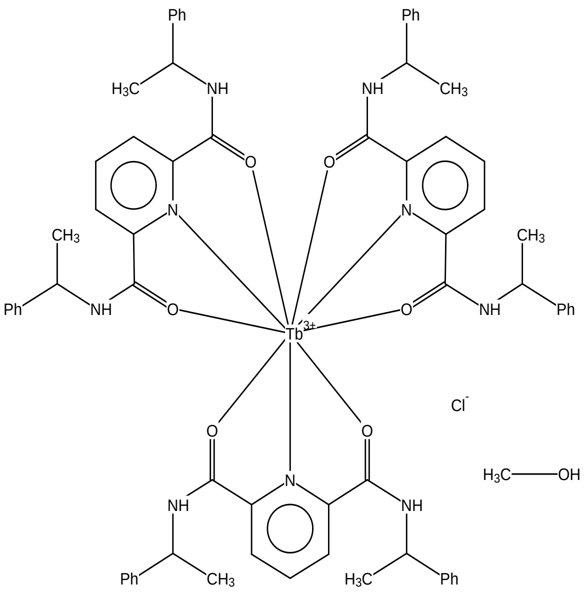

HAWQON

Reference: K.T.Hua, Jide Xu, E.E.Quiroz, S.Lopez, A.J.Ingram, V.A.Johnson, A.R.Tisch, A.de Bettencourt-Dias, D.A.Straus, G.Muller (2012) *Inorg.Chem.* ,**51,**647

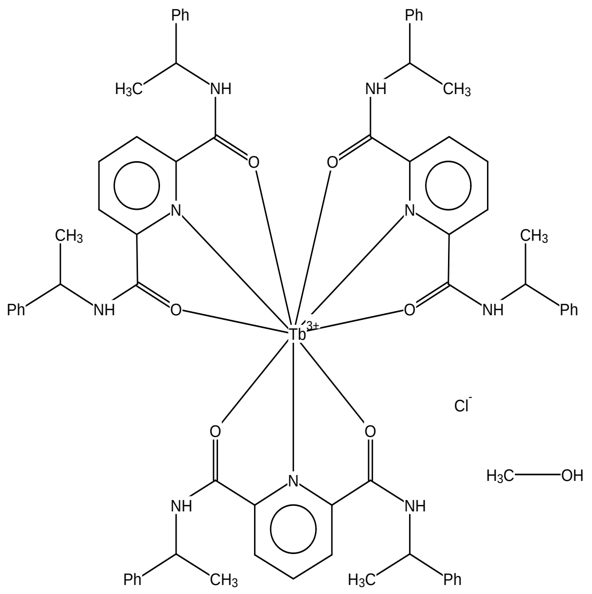

HAWQUT

Reference: K.T.Hua, Jide Xu, E.E.Quiroz, S.Lopez, A.J.Ingram, V.A.Johnson, A.R.Tisch, A.de Bettencourt-Dias, D.A.Straus, G.Muller (2012) *Inorg.Chem.* ,**51,**647

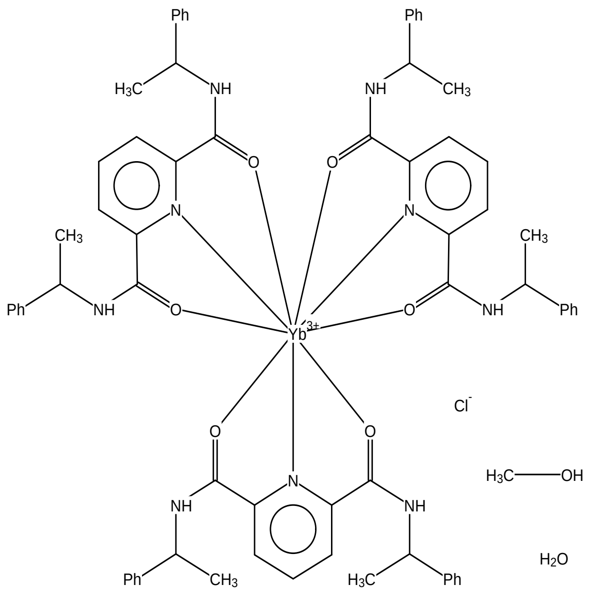

HAWRAA

Reference: K.T.Hua, Jide Xu, E.E.Quiroz, S.Lopez, A.J.Ingram, V.A.Johnson, A.R.Tisch, A.de Bettencourt-Dias, D.A.Straus, G.Muller (2012) *Inorg.Chem.* ,**51,**647

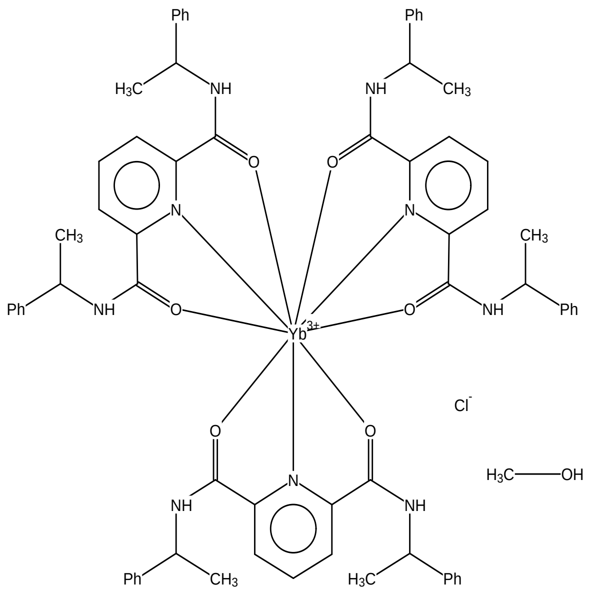

HOLMEA

Reference: I.L.Karle, D.Ranganathan, S.Kurur (1999) *J.Am.Chem.Soc.* ,**121,**7156

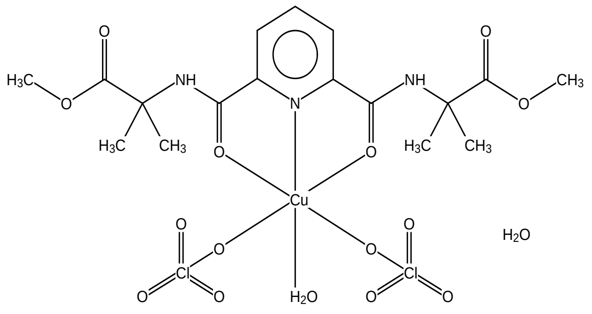

IKAPIW

Reference: Tang xianhui (2021)
CSD Communication(Private Communication) ,

Eu_4--
--[μ_2-$C_{34}H_{18}Cl_2N_6O_4Ph_2(OEt)_2$-$\kappa^2$N,N',$\kappa^4$O,O',O'',O''']$_5$[$H_2O$]$_6^{12+}$

F_3C—SO_3^-

IKAPIW01

Reference: Xianhui Tang, Dandan Chu, Wei Gong, Yong Cui, Yan Liu (2021) *Angew.Chem.,Int.Ed.* ,**60,**9099

Eu_4--
--[μ_2-$C_{34}H_{18}Cl_2N_6O_4Ph_2(OEt)_2$-$\kappa^2$N,N',$\kappa^4$O,O',O'',O''']$_5$[$H_2O$]$_6^{12+}$

F_3C—SO_3^-

ILOROQ

Reference: T.Le Borgne, J.-M.Benech, S.Floquet, G.Bernardinelli, C.Aliprandini, P.Bettens, C.Piguet (2003) *Dalton Trans.* ,3856

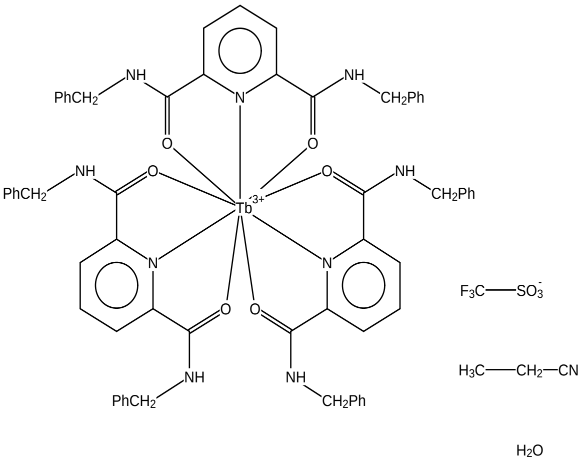

IVIKOQ

Reference: K.V.Deriabin, N.A.Ignatova, S.O.Kirichenko, A.S.Novikov, M.A.Kryukova, V.Yu.Kukushkin, R.M.Islamova (2021)
CSD Communication(Private Communication) ,

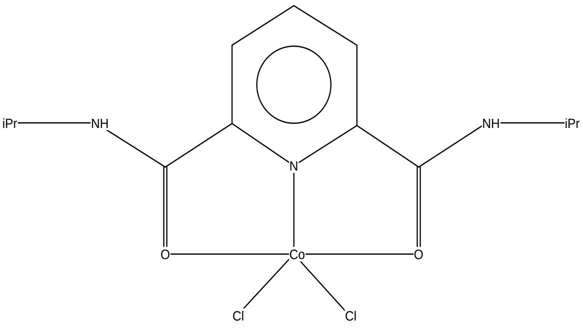

JIQFOF

Reference: J.P.Leonard, P.Jensen, T.McCabe, J.E.O'Brien, R.D.Peacock, P.E.Kruger, T.Gunnlaugsson (2007) *J.Am.Chem.Soc.* , **129,**10986

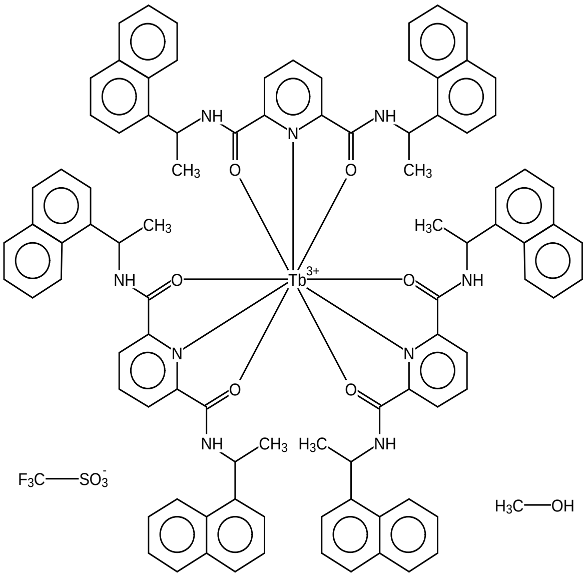

JIQFUL

Reference: J.P.Leonard, P.Jensen, T.McCabe, J.E.O'Brien, R.D.Peacock, P.E.Kruger, T.Gunnlaugsson (2007) *J.Am.Chem.Soc.* , **129,**10986

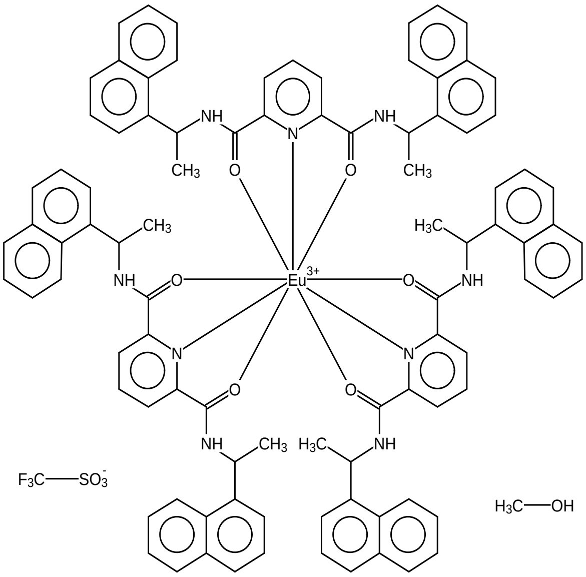

JIQGAS

Reference: J.P.Leonard, P.Jensen, T.McCabe, J.E.O'Brien, R.D.Peacock, P.E.Kruger, T.Gunnlaugsson (2007) *J.Am.Chem.Soc.* , **129,**10986

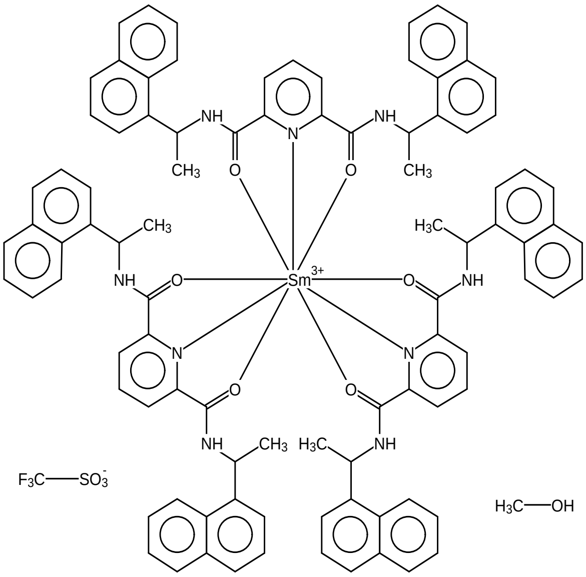

JIQGEW

Reference: J.P.Leonard, P.Jensen, T.McCabe, J.E.O'Brien, R.D.Peacock, P.E.Kruger, T.Gunnlaugsson (2007) *J.Am.Chem.Soc.* , **129,**10986

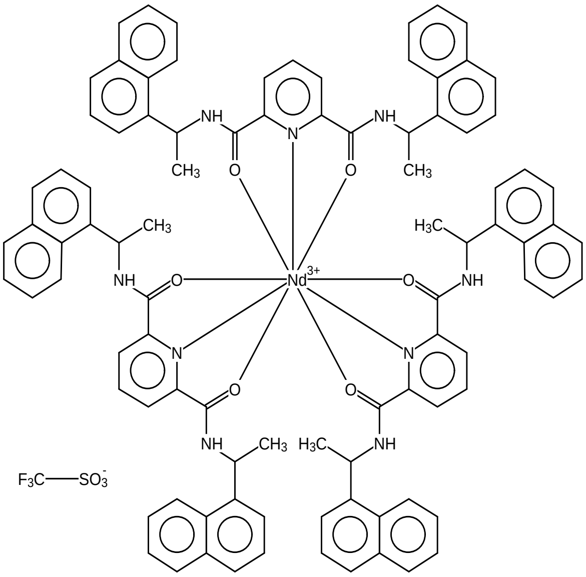

Search: search1 (Mon Jul 8 09:53:10 2024): Hits 33-36

JUNRER

Reference: Gen Zhang, G.Gil-Ramirez, A.Markevicius, C.Browne, I.J.Vitorica-Yrezabal, D.A.Leigh (2015) *J.Am.Chem.Soc.* ,**137,**10437

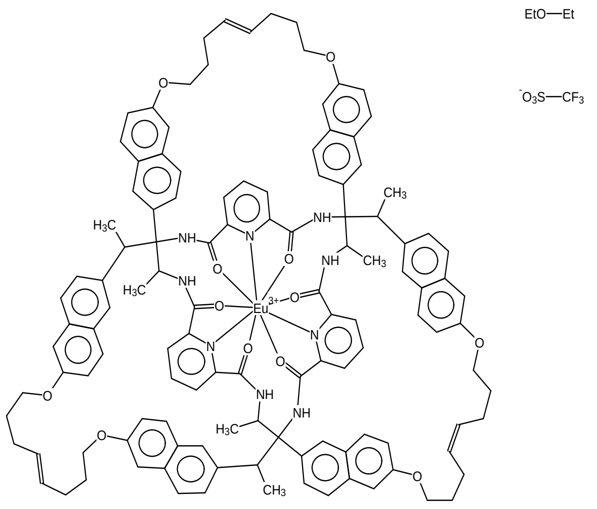

JUNRIV

Reference: Gen Zhang, G.Gil-Ramirez, A.Markevicius, C.Browne, I.J.Vitorica-Yrezabal, D.A.Leigh (2015) *J.Am.Chem.Soc.* ,**137,**10437

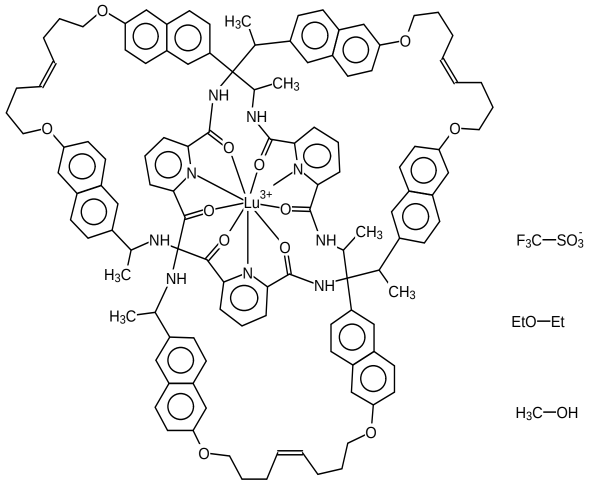

KABRUE

Reference: K.V.Deriabin, N.A.Ignatova, S.O.Kirichenko, A.S.Novikov, R.M.Islamova (2020) *Polymer* ,**212,**123119

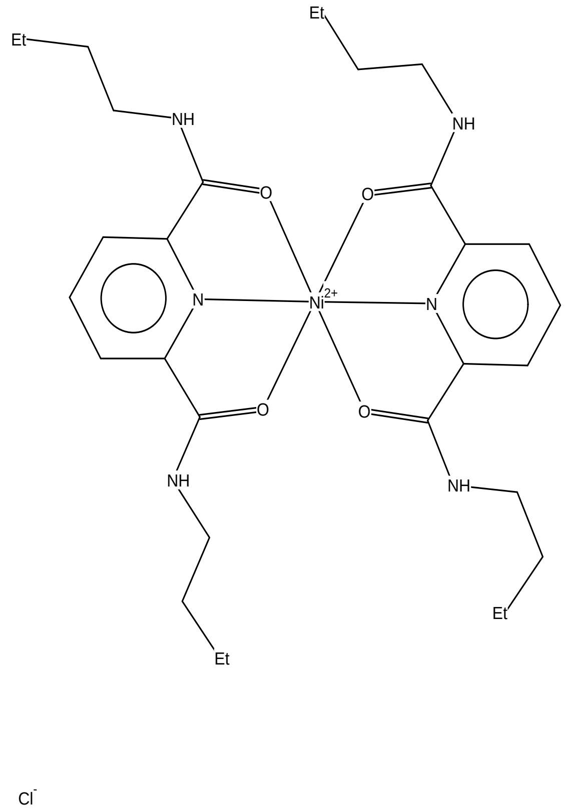

MOBMOI

Reference: Qian-Qian Yan, Li-Peng Zhou, Hai-Yue Zhou, Zhuo Wang, Li-Xuan Cai, Xiao-Qing Guo, Xiao-Qi Sun, Qing-Fu Sun (2019) *Dalton Trans.* ,**48,**7080

ClO_4^-

$Eu_4[\mu_3\text{-}C_{69}H_{51}N_9O_6\text{-}\kappa^3N,N',N'',\kappa^6O,O',O'',O''',O'''',O''''']_4^{12+}$

Search: search1 (Mon Jul 8 09:53:10 2024): Hits 37-40

QEPMUU

Reference: A.-S.Chauvin, J.-C.G.Bunzli, F.Bochud, R.Scopelliti, P.Froidevaux (2006) *Chem.-Eur.J.* ,**12,**6852

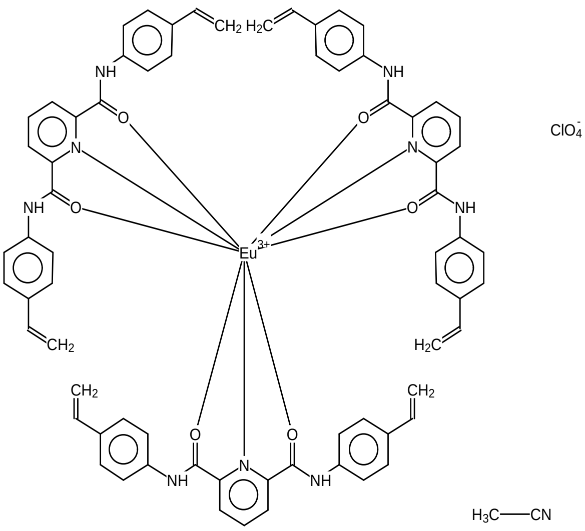

RULNIX

Reference: Liang-Liang Yan, Chun-Hong Tan, Guang-Lu Zhang, Li-Peng Zhou, J.-C.Bunzli, Qing-Fu Sun (2015) *J.Am.Chem.Soc.* ,**137,** 8550

$Eu_4[\mu_3\text{-}C_{48}H_{33}N_9O_6Ph_3Me_3\text{-}\kappa^3N,N',N'',\kappa^6O,O',O'',O''',O'''',O''''']_4^{12+}$

ClO_4^-

H_2O

RUZHEZ

Reference: A.Szumna, D.T.Gryko, J.Jurczak (2000) *J.Chem.Soc.,Perkin Trans.2* ,1553

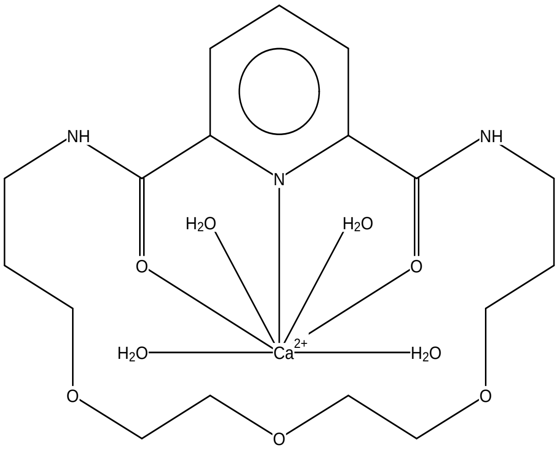

ClO_4^-

SEKNEE

Reference: Xiao-Zhen Li, Li-Peng Zhou, Liang-Liang Yan, Da-Qiang Yuan, Chen-Sheng Lin, Qing-Fu Sun (2017) *J.Am.Chem.Soc.* ,**139,**8237

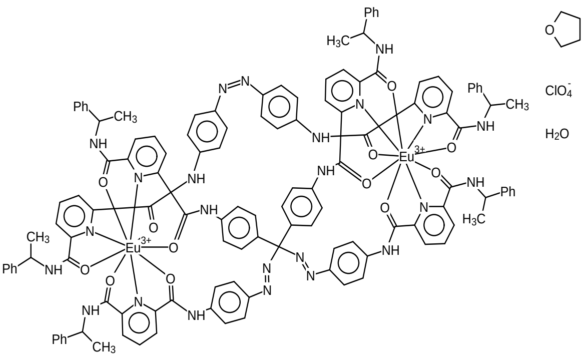

Search: search1 (Mon Jul 8 09:53:10 2024): Hits 41-44

TITBAC

Reference: Ingrid Lehman-Andino, Jing Su, K.E.Papathanasiou, T.M.Eaton, Jiwen Jian, David Dan, T.E.Albrecht-Schmitt, C.J.Dares, E.R.Batista, Ping Yang, J.K.Gibson, K.Kavallieratos (2019) *Chem.Commun.* ,**55,**2441

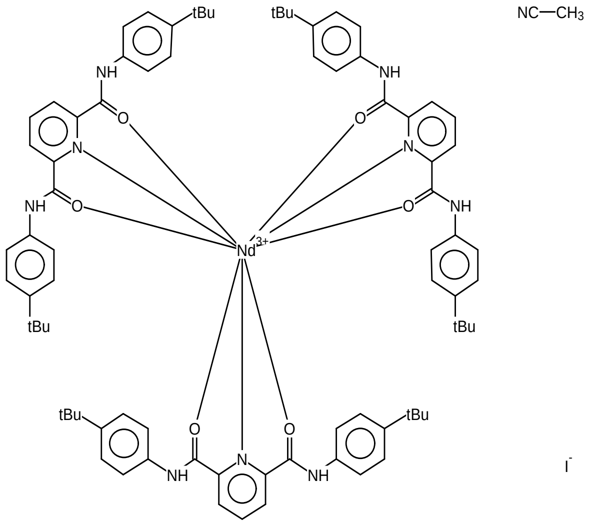

VEVHAG

Reference: S.Tanase, P.M.Gallego, R.de Gelder, Wen Tian Fu (2007) *Inorg.Chim.Acta* ,**360,**102

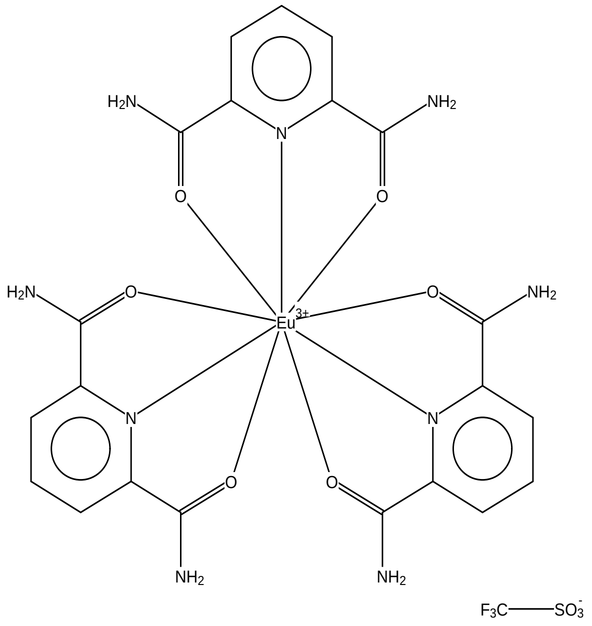

VEVHEK

Reference: S.Tanase, P.M.Gallego, R.de Gelder, Wen Tian Fu (2007) *Inorg.Chim.Acta* ,**360,**102

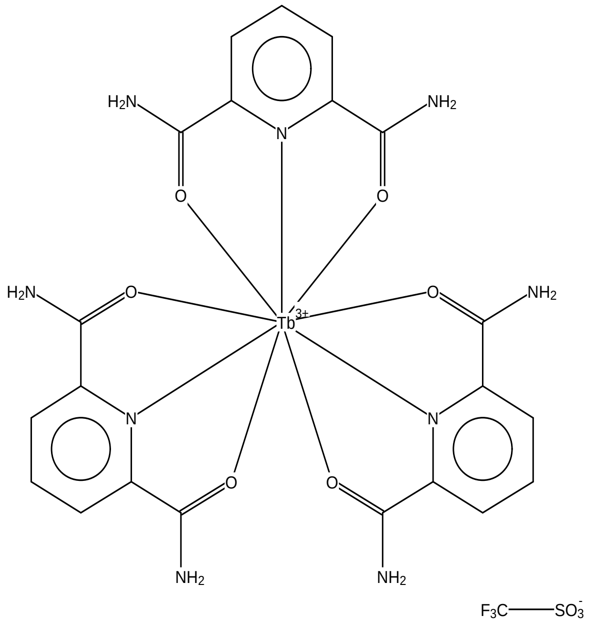

WALTIQ

Reference: Xiao-Lu Hu, Xuan-Di Tang, Yuan-Yuan Wang, Tian-Tian Huang, Qi Yue, En-Qing Gao (2020) *J.Solid State Chem.* , **294,**121871

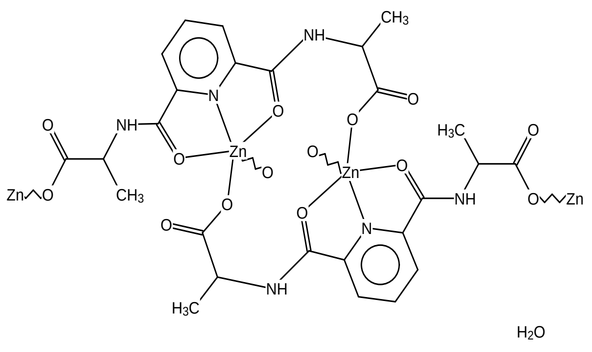

Search: search1 (Mon Jul 8 09:53:10 2024): Hits 45-48

WORSAA

Reference: M.Starck, L.E.MacKenzie, A.S.Batsanov, D.Parker, R.Pal (2019) *Chem.Commun.* ,**55,**14115

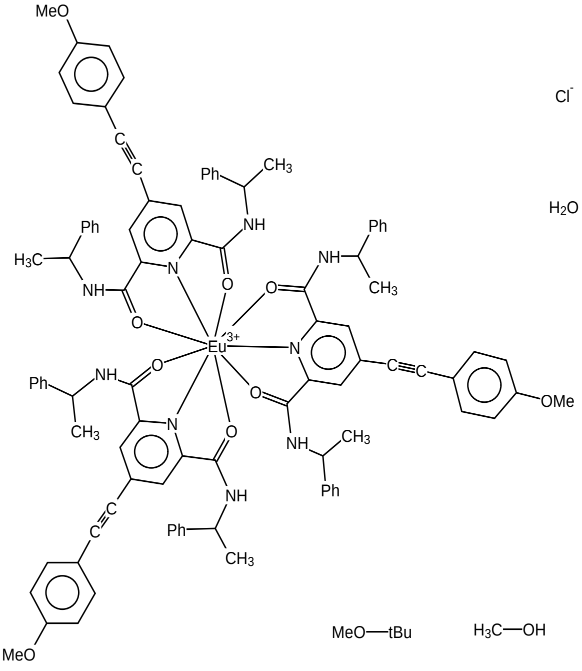

WORSEE

Reference: M.Starck, L.E.MacKenzie, A.S.Batsanov, D.Parker, R.Pal (2019) *Chem.Commun.* ,**55,**14115

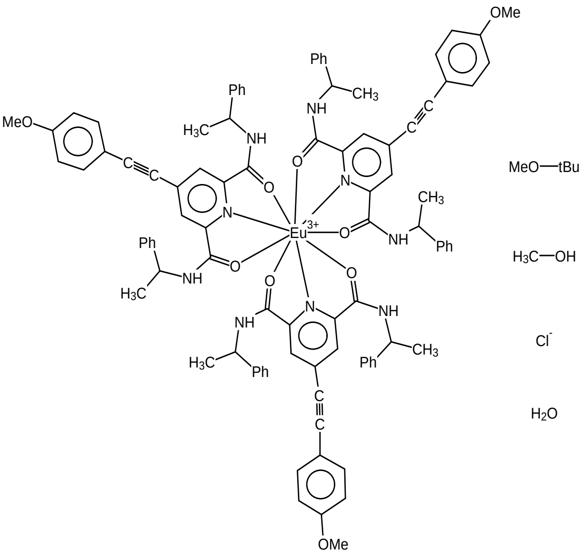

XESCOQ

Reference: Chi-Tung Yeung, King-Him Yim, Ho-Yin Wong, R.Pal, Wai-Sum Lo, Siu-Cheong Yan, Melody Yee-Man Wong, D.Yufit, D.E.Smiles, L.J.McCormick, S.J.Teat, D.K.Shuh, Wing-Tak Wong, G.-L.Law (2017) *Nat.Commun.* ,**8,**1128

$Eu_4[\mu_2\text{-}C_{30}H_{18}N_6O_6Ph_2Me_2\text{-}\kappa^2N,N',\kappa^4O,O',O'',O''']_6^{12+}$

F_3C—SO_3^-

XOWSOT

Reference: Chi-Tung Yeung, Wesley Ting Kwok Chan, Siu-Cheong Yan, Kwan-Leung Yu, King-Him Yim, Wing-Tak Wong, G.-L.Law (2015) *Chem.Commun.* ,**51,**592

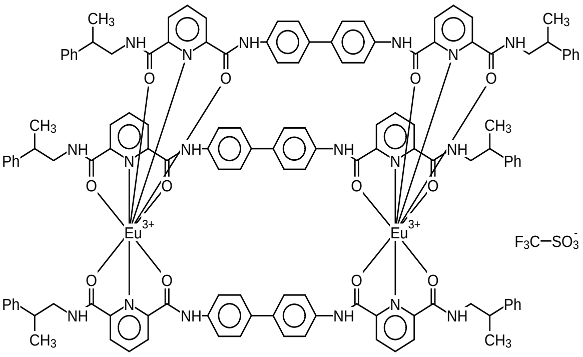

Search: search1 (Mon Jul 8 09:53:10 2024): Hits 49-52

XOWTIO

Reference: Chi-Tung Yeung, Wesley Ting Kwok Chan, Siu-Cheong Yan, Kwan-Leung Yu, King-Him Yim, Wing-Tak Wong, G.-L.Law (2015) *Chem.Commun.* ,**51,**592

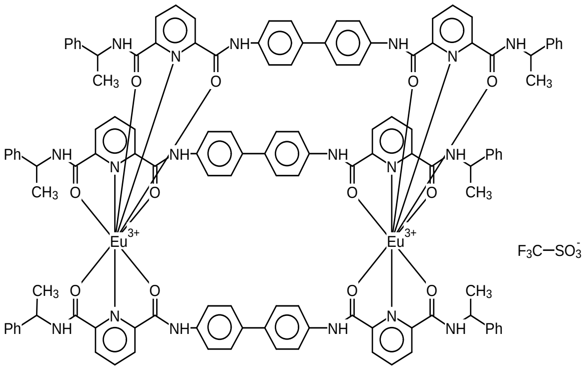

$F_3C{-}SO_3^-$

YIDLUV

Reference: Zhuo Wang, Li-Peng Zhou, Tong-Han Zhao, Li-Xuan Cai, Xiao-Qing Guo, Peng-Fei Duan, Qing-Fu Sun (2018) *Inorg.Chem.* ,**57,** 7982

$Pd_6Eu_2[\mu_3\text{-}C_{32}H_{22}N_6O_3Me\text{-}\kappa^3N,N',N'',\kappa^2O,O']_6[C_{10}H_8N_2\text{-}\kappa^2N,N']_6^{12+}$

$^-O_3S{-}CF_3$

ZOYRUC

Reference: G.L.Guillet, J.B.Gordon, G.N.Di Francesco, M.W.Calkins, E.Cizmar, K.A.Abboud, M.W.Meisel, R.Garcia-Serres, L.J.Murray (2015) *Inorg.Chem.* ,**54,**2691

Fe_2--
--$[\mu_2\text{-}C_{59}H_{29}N_9O_6(Pr^i)_6(OEt)_6\text{-}\kappa^2N,N',\kappa^4O,O',O'',O'''][\mu_2\text{-}Cl]_2[C_4H_8O][H_2O]^{2+}$

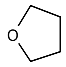

$^-O_3S{-}CF_3$

ZOYSEN

Reference: G.L.Guillet, J.B.Gordon, G.N.Di Francesco, M.W.Calkins, E.Cizmar, K.A.Abboud, M.W.Meisel, R.Garcia-Serres, L.J.Murray (2015) *Inorg.Chem.* ,**54,**2691

Co_3--
--$[\mu_3\text{-}C_{59}H_{29}N_9O_6(Pr^i)_6(OEt)_6\text{-}\kappa^3N,N',N'',\kappa^6O,O',O'',O''',O'''',O'''''][\mu_3\text{-}HO][\mu_2\text{-}O_3S(CF_3)][\mu_2\text{-}HO][O_3S(CF_3)][CNMe]^{2+}$

$^-O_3S{-}CF_3$

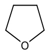

$H_3C{-}CN$

Search: search1 (Mon Jul 8 09:53:10 2024): Hits 53-56

ZOYSOX

Reference: G.L.Guillet, J.B.Gordon, G.N.Di Francesco, M.W.Calkins, E.Cizmar, K.A.Abboud, M.W.Meisel, R.Garcia-Serres, L.J.Murray (2015) *Inorg.Chem.* ,**54,**2691

Fe_3--[μ_3-$C_{59}H_{29}N_9O_6(Pr^i)_6(OEt)_6$-$\kappa^3$N,N',N'',$\kappa^6$O,O',O'',O''',O'''',O'''''][μ_3-O][CNMe]$_3^{4+}$

NC—CH_3

Ph—CH_3

^-O_3S—CF_3

FALLIR01

Reference: Xiao-Lu Hu, Xuan-Di Tang, Yuan-Yuan Wang, Tian-Tian Huang, Qi Yue, En-Qing Gao (2021) *J.Solid State Chem.* , **294,**121871

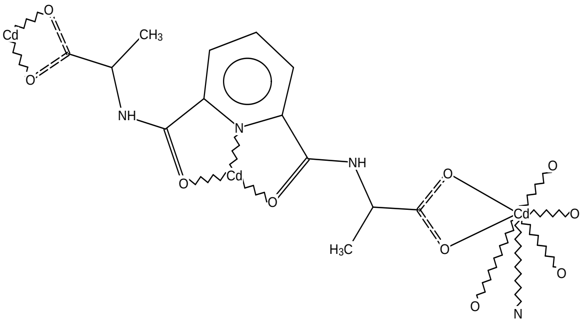

WALTIQ01

Reference: Xiao-Lu Hu, Xuan-Di Tang, Yuan-Yuan Wang, Tian-Tian Huang, Qi Yue, En-Qing Gao (2021) *J.Solid State Chem.* , **294,**121871

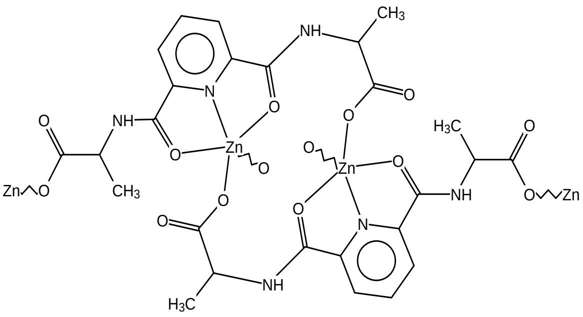

H_2O

LERBOD

Reference: Qiang-Yu Zhu, Li-Peng Zhou, Li-Xuan Cai, Shao-Jun Hu, Xiao-Zhen Li, Qing-Fu Sun (2022) *Inorg.Chem.* ,

F_3C—SO_3^-

$Pt_6Yb_4[\mu_2$-$C_{15}H_9N_3O_2(Pr^i)$-$\kappa^1C,\kappa^1N,\kappa^2O,O']_{12}[PEt_3]_{12}^{12+}$

Search Overview

N^{-}^N^O Coordination

Search:	search11
Date/Time done:	Wed May 29 14:03:03 2024
Database(s):	CSD version 5.43 updates (Mar 2022)<br>CSD version 5.43 (November 2021)<br>CSD version 5.43 updates (Sep 2022)<br>CSD version 5.43 updates (Nov 2022)
Restriction Info:	No refcode restrictions applied
Filters:	None
Percentage Completed:	100%
Number of Hits:	4

Single query used. Search found structures that:

match

Query 1

Query 1

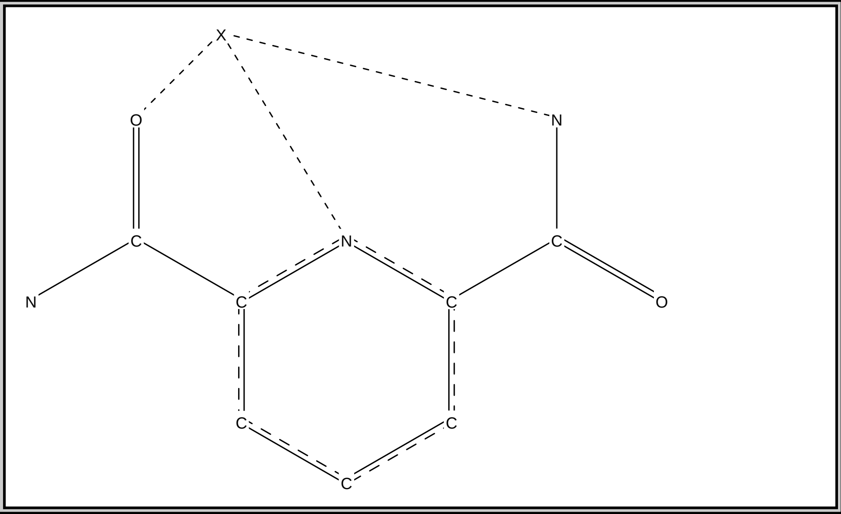

Search: search11 (Wed May 29 14:03:03 2024): Hits 1-3

BIFHII

Reference: A.N.Dwyer, M.C.Grossel, P.N.Horton (2004) *Supramol.Chem.* ,**16**,405

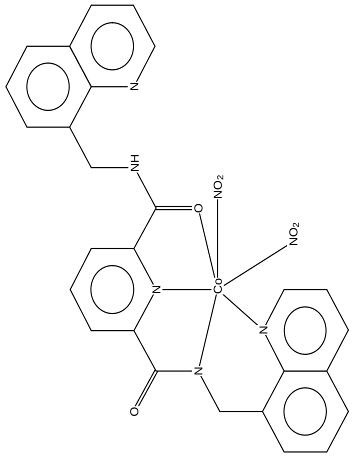

HUTNIW

Reference: Yinghua Li, Weibin Fan, Zilong Zhang, Xingkun Xie, Shiqun Xiang, Deguang Huang (2020) *Dalton Trans.* ,**49**,12189

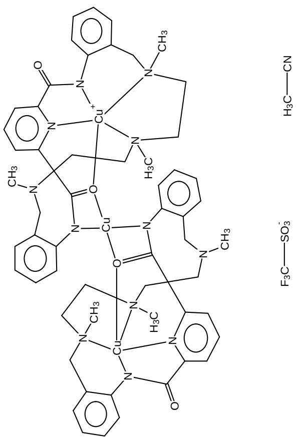

VOFDOM

Reference: C.McManus, P.Mondal, M.Lovisari, B.Twamley, A.R.McDonald (2019) *Inorg.Chem.* ,**58**,4515

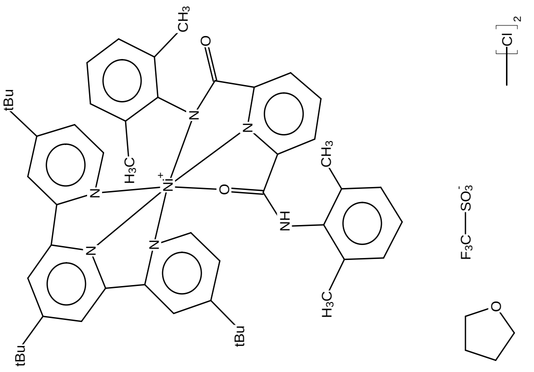

Search Overview

O$^-$^N^O Coordination

Search: search2

Date/Time done: Thu Jun 6 18:37:37 2024

Database(s): CSD version 5.43 updates (Mar 2022)
CSD version 5.43 (November 2021)
CSD version 5.43 updates (Sep 2022)
CSD version 5.43 updates (Nov 2022)

Restriction Info: No refcode restrictions applied

Filters: None

Percentage Completed: 100%

Number of Hits: 1

Single query used. Search found structures that:

match

Query 1

Query 1

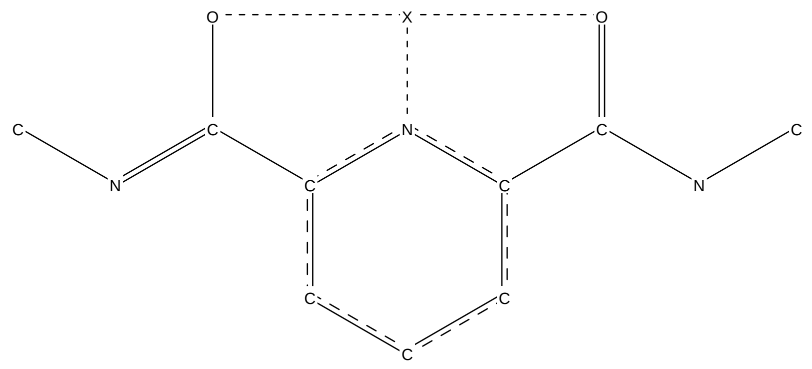

EDOPUJ

Reference: C.Wallenhorst, K.V.Axenov, G.Kehr, J.S.M.Samec, R.Frohlich, G.Erker (2007) *Z.Naturforsch.,B:Chem.Sci.* ,**62**,783

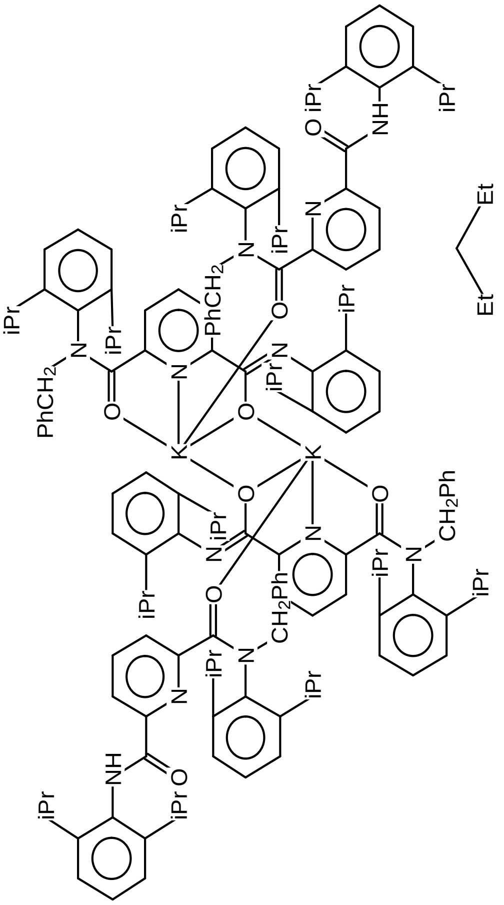

Search Overview

N⁻^N^N⁻ Coordination

Search:	search13
Date/Time done:	Wed May 29 14:10:34 2024
Database(s):	CSD version 5.43 updates (Mar 2022)<br>CSD version 5.43 (November 2021)<br>CSD version 5.43 updates (Sep 2022)<br>CSD version 5.43 updates (Nov 2022)
Restriction Info:	No refcode restrictions applied
Filters:	None
Percentage Completed:	100%
Number of Hits:	586

Single query used. Search found structures that:

match

Query 1

Query 1

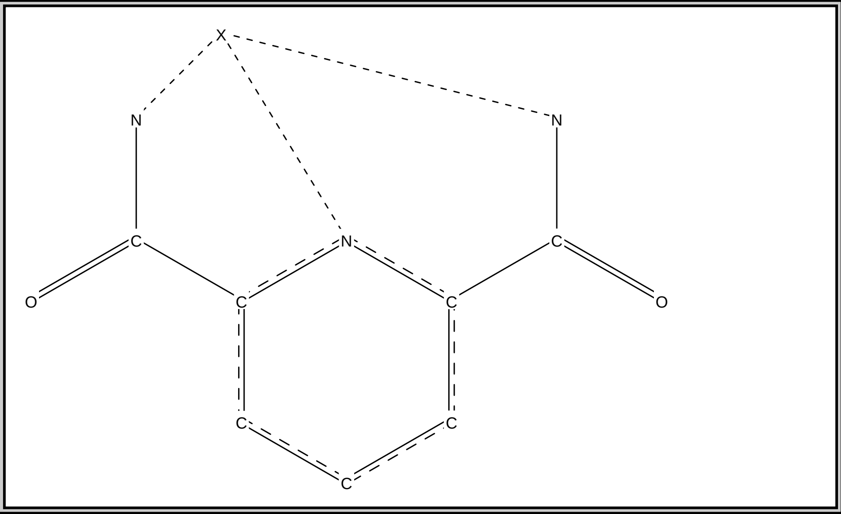

ENESEY

Reference: Lixian Wen (2021) *CSD Communication(Private Communication)* ,

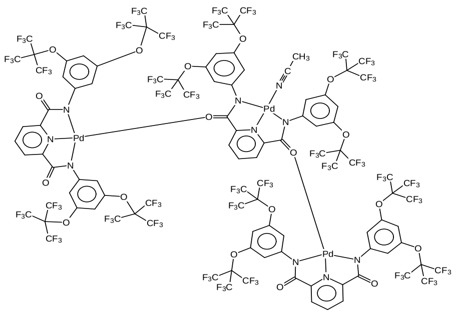

QAWQOY

Reference: C.J.Bouchey, W.B.Tolman (2022) *Inorg.Chem.* ,**61,**2662

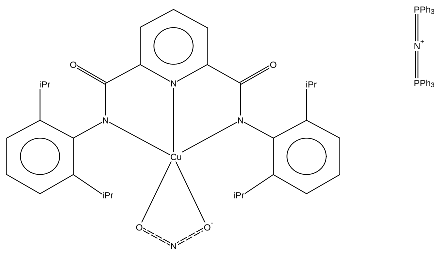

XATMUE

Reference: D.Prabha, D.Singh, P.Kumar, R.Gupta (2021) *Inorg.Chem.* ,**60,**17889

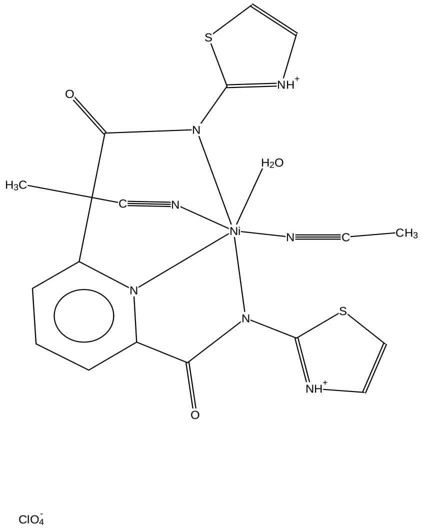

XATNAL

Reference: D.Prabha, D.Singh, P.Kumar, R.Gupta (2021) *Inorg.Chem.* ,**60,**17889

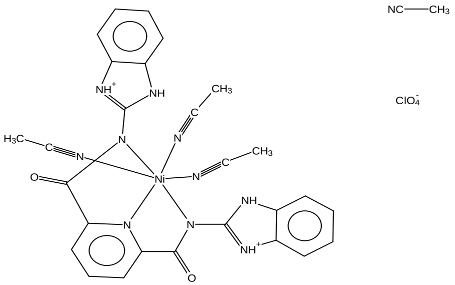

XATNEP

Reference: D.Prabha, D.Singh, P.Kumar, R.Gupta (2021) *Inorg.Chem.* ,**60,**17889

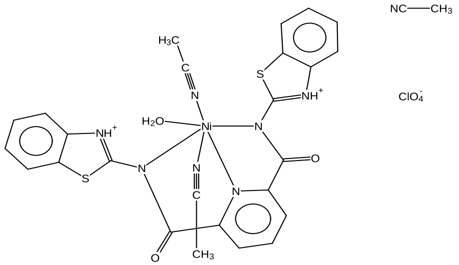

ADEJUQ

Reference: T.Moriuchi, K.Morimoto, Y.Sakamoto, T.Hirao (2012) *Eur.J.Inorg.Chem.* ,4669

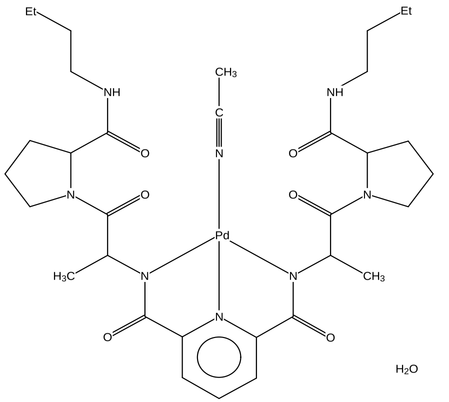

ADEKAX

Reference: T.Moriuchi, K.Morimoto, Y.Sakamoto, T.Hirao (2012) *Eur.J.Inorg.Chem.* ,4669

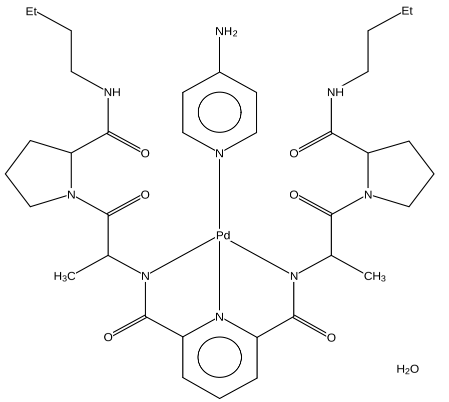

AHOTIC

Reference: Jianfeng Wu, Lang Zhao, Mei Guo, Jinkui Tang (2015) *Chem.Commun.* ,**51,**17317

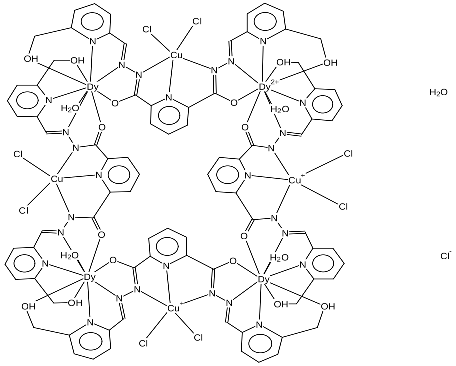

Search: search13 (Wed May 29 14:10:34 2024): Hits 9-16

ALESAL

Reference: A.K.Singh, V.Balamurugan, R.Mukherjee (2003) *Inorg.Chem.* ,**42**,6497

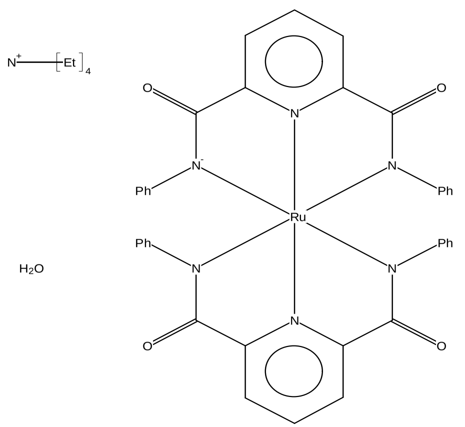

ANICEI

Reference: V.Mahesh Krishnan, Dimitar Y.Shopov, C.J.Bouchey, W.D.Bailey, R.Parveen, B.Vlaisavljevich, W.B.Tolman (2021) *J.Am.Chem.Soc.* ,**143**,3295

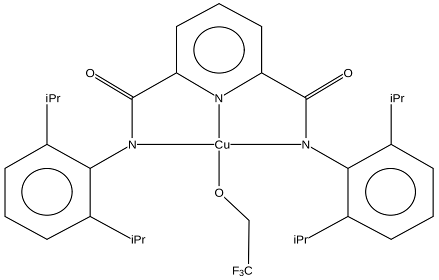

ANICIM

Reference: V.Mahesh Krishnan, Dimitar Y.Shopov, C.J.Bouchey, W.D.Bailey, R.Parveen, B.Vlaisavljevich, W.B.Tolman (2021) *J.Am.Chem.Soc.* ,**143**,3295

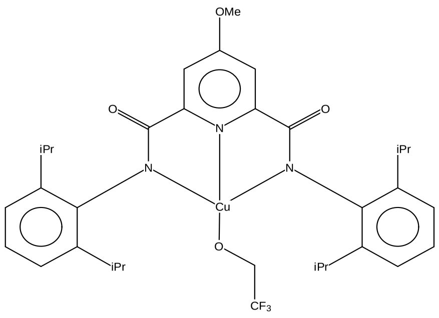

ANICOS

Reference: V.Mahesh Krishnan, Dimitar Y.Shopov, C.J.Bouchey, W.D.Bailey, R.Parveen, B.Vlaisavljevich, W.B.Tolman (2021) *J.Am.Chem.Soc.* ,**143**,3295

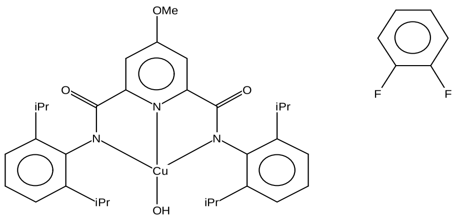

ANICUY

Reference: V.Mahesh Krishnan, Dimitar Y.Shopov, C.J.Bouchey, W.D.Bailey, R.Parveen, B.Vlaisavljevich, W.B.Tolman (2021) *J.Am.Chem.Soc.* ,**143**,3295

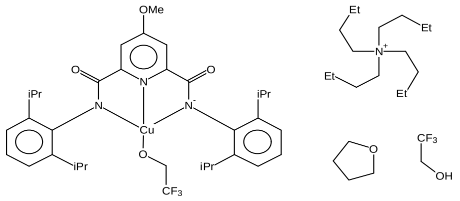

ANIDAF

Reference: V.Mahesh Krishnan, Dimitar Y.Shopov, C.J.Bouchey, W.D.Bailey, R.Parveen, B.Vlaisavljevich, W.B.Tolman (2021) *J.Am.Chem.Soc.* ,**143**,3295

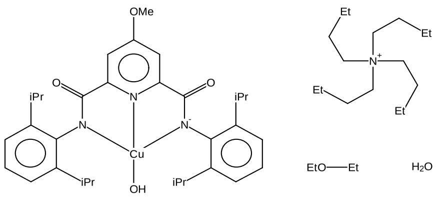

ANIDEJ

Reference: V.Mahesh Krishnan, Dimitar Y.Shopov, C.J.Bouchey, W.D.Bailey, R.Parveen, B.Vlaisavljevich, W.B.Tolman (2021) *J.Am.Chem.Soc.* ,**143**,3295

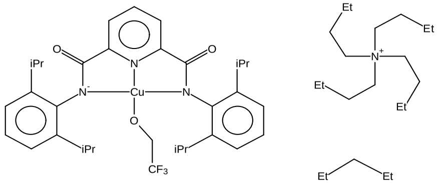

AQUXER

Reference: T.Corona, Anna Company (2016) *Dalton Trans.* ,**45**,14530

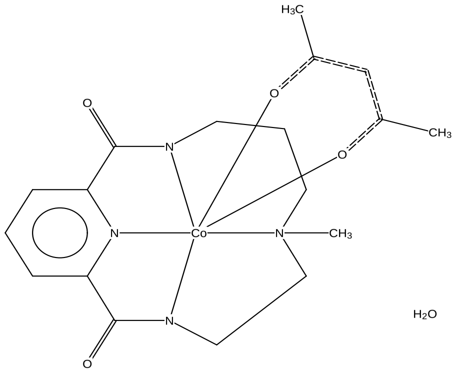

AQUYAO

Reference: T.Corona, Anna Company (2016) *Dalton Trans.* ,**45,**14530

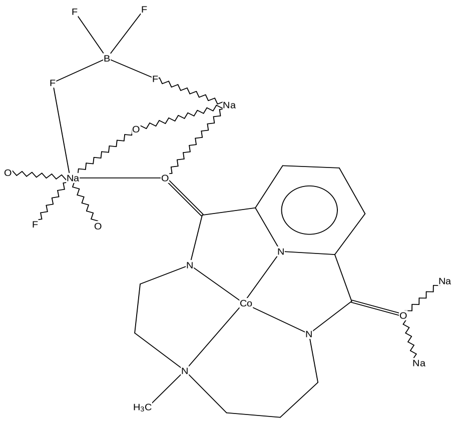

AVUSEP

Reference: S.L.Jain, P.Bhattacharyya, H.L.Milton, A.M.Z.Slawin, J.A.Crayston, J.D.Woollins (2004) *Dalton Trans.* ,862

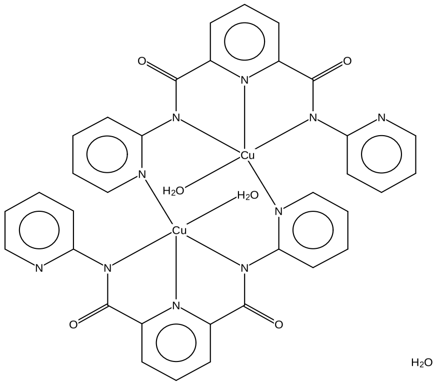

AWUNEN

Reference: Debanjan Dhar, G.M.Yee, T.F.Markle, J.M.Mayer, W.B.Tolman (2017) *Chemical Science* ,**8,**1075

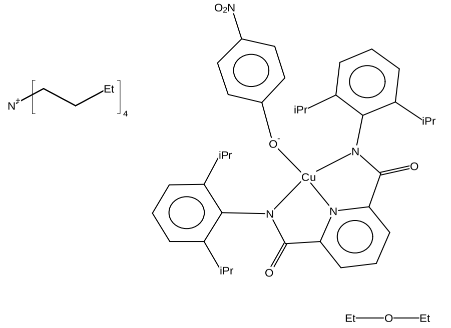

AZOXAO

Reference: I.V.Korendovych, R.J.Staples, W.M.Reiff, E.V.Rybak-Akimova (2004) *Inorg.Chem.* ,**43,**3930

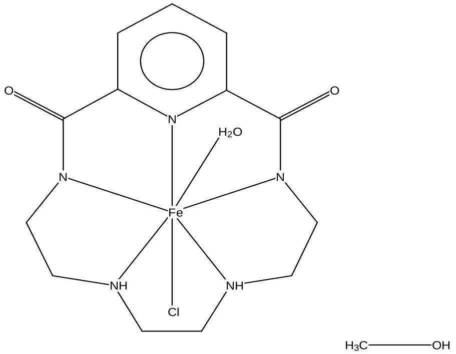

BANYIB

Reference: P.Kumar, V.Kumar, R.Gupta (2017) *RSC Advances* ,**7,** 7734

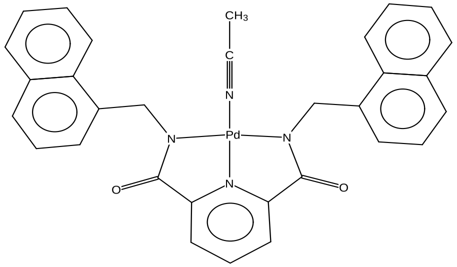

BEHLEI

Reference: B.Nisar Ahamed, P.Van Velthem, K.Robeyns, C.-A.Fustin (2017) *ACS Macro Letters* ,**6,**468

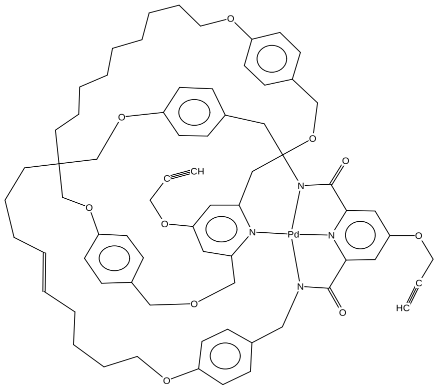

BENKUA

Reference: S.Brooker, G.S.Dunbar, G.B.Jameson (1999) *Polyhedron* , **18,**679

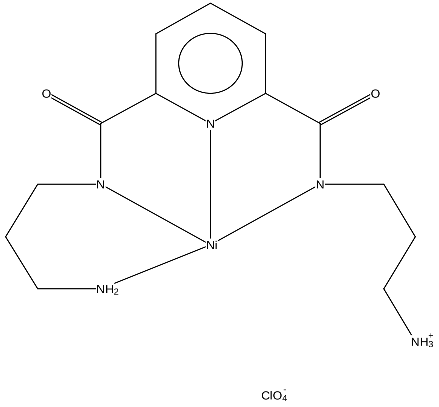

BEXMUP

Reference: V.Carta, S.Hessam M.Mehr, M.J.MacLachlan (2018) *Inorg.Chem.* ,**57,**3243

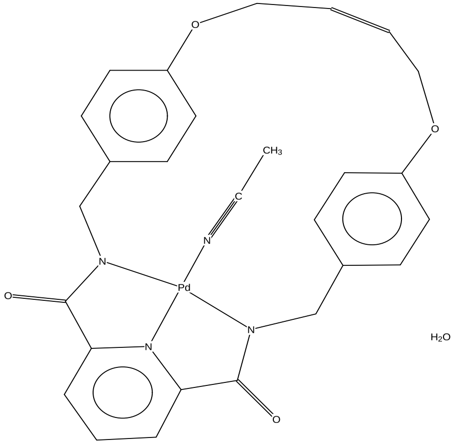

BEXNEA
Reference: V.Carta, S.Hessam M.Mehr, M.J.MacLachlan (2018) *Inorg.Chem.*, **57**,3243

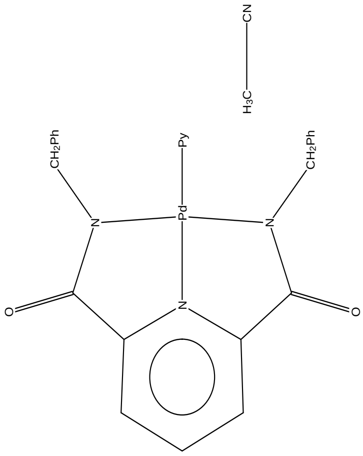

BEXNOK
Reference: V.Carta, S.Hessam M.Mehr, M.J.MacLachlan (2018) *Inorg.Chem.*, **57**,3243

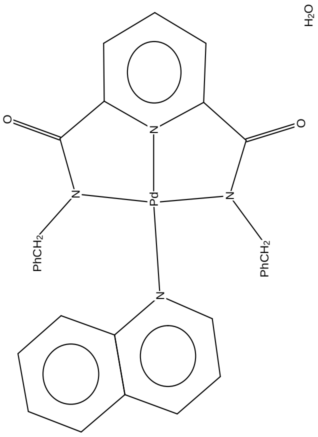

BEXNUQ
Reference: V.Carta, S.Hessam M.Mehr, M.J.MacLachlan (2018) *Inorg.Chem.*, **57**,3243

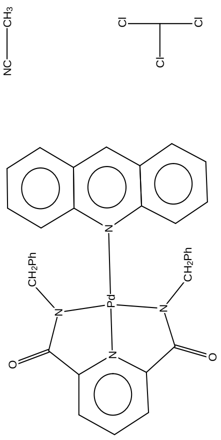

BEXPAY
Reference: V.Carta, S.Hessam M.Mehr, M.J.MacLachlan (2018) *Inorg.Chem.*, **57**,3243

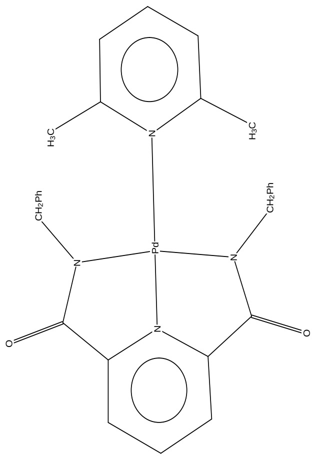

BEXPEC
Reference: V.Carta, S.Hessam M.Mehr, M.J.MacLachlan (2018) *Inorg.Chem.*, **57**,3243

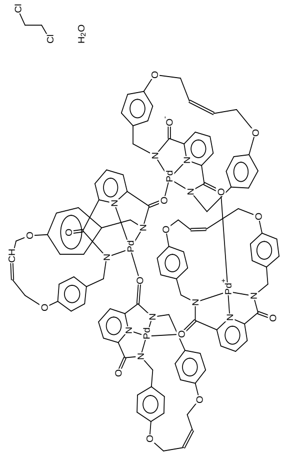

BEXPIG
Reference: V.Carta, S.Hessam M.Mehr, M.J.MacLachlan (2018) *Inorg.Chem.*, **57**,3243

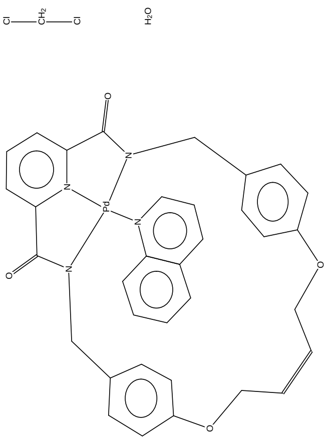

BEXPOM
Reference: V.Carta, S.Hessam M.Mehr, M.J.MacLachlan (2018) *Inorg.Chem.*, **57**,3243

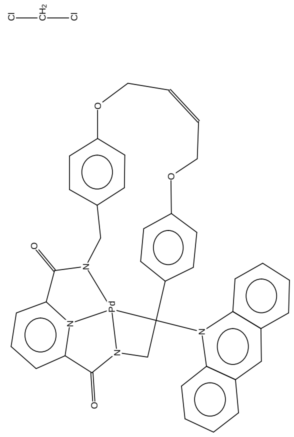

BIFHAA
Reference: A.N.Dwyer, M.C.Grossel, P.N.Horton (2004) *Supramol.Chem.*, **16**,405

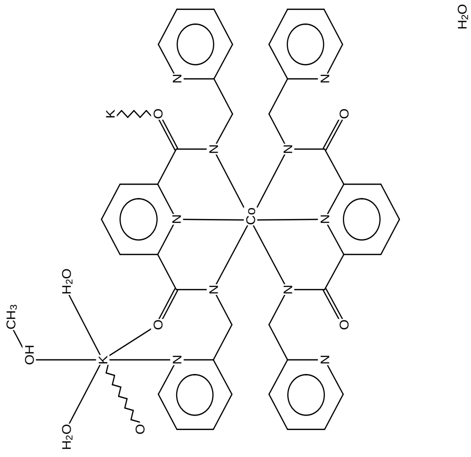

Search: search13 (Wed May 29 14:10:34 2024): Hits 33-40

BIFQUC

Reference: M.Otsuka, H.Satake, S.Murakami, M.Doi, T.Ishida, M.Shibasaki, Y.Sugiura (1996) *Bioorg.Med.Chem.* ,**4,**1703

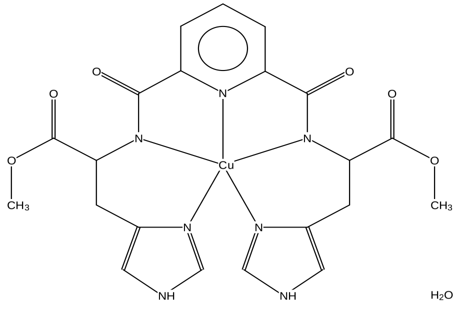

H_2O

BIFQUC01

Reference: H.Kurosaki, R.K.Sharma, S.Aoki, T.Inoue, Y.Okamoto, Y.Sugiura, M.Doi, T.Ishida, M.Otsuka, M.Goto (2001) *J.Chem.Soc.,Dalton Trans.* ,441

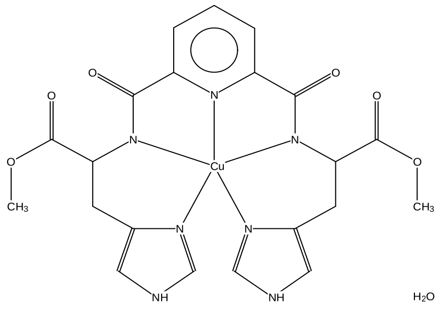

H_2O

BIPLIX

Reference: G.Kumar, R.Gupta (2013) *Inorg.Chem.* ,**52,**10773

n-$Co_2Zn_5[\mu_5\text{-}C_{21}H_{11}N_3O_6]_2$--
--$[\mu_4\text{-}C_{21}H_{11}N_3O_6\text{-}\kappa^3N,N',N'',\kappa^3O,O',O'']$--
--$[\mu_3\text{-}C_{21}H_{11}N_3O_6\text{-}\kappa^3N,N',N'',\kappa^3O,O',O''][\mu_3\text{-}HO]_2[\mu_2\text{-}H_2O][H_2O]_3$

H_2O

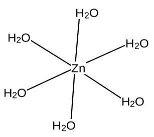

BIPLOD

Reference: G.Kumar, R.Gupta (2013) *Inorg.Chem.* ,**52,**10773

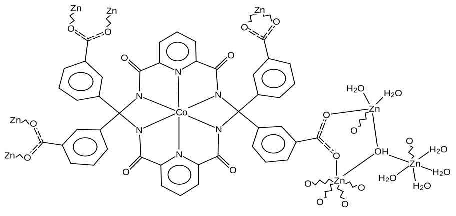

H_2O

BITROO

Reference: Qiaoqiao Teng, Han Vinh Huynh (2018) *Organometallics* , **37,**4119

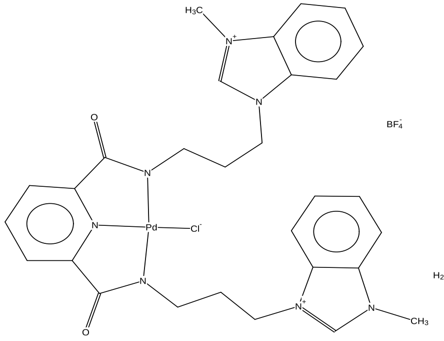

BF_4^-

H_2O

BITRUU

Reference: Qiaoqiao Teng, Han Vinh Huynh (2018) *Organometallics* , **37,**4119

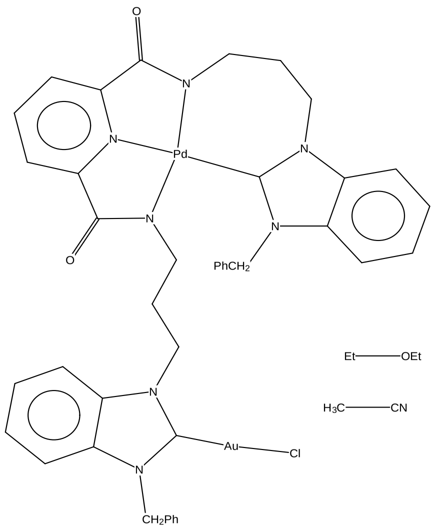

Et——OEt

H_3C——CN

BIWDIV

Reference: A.Mishra, A.Ali, S.Upreti, R.Gupta (2008) *Inorg.Chem.* ,**47,** 154

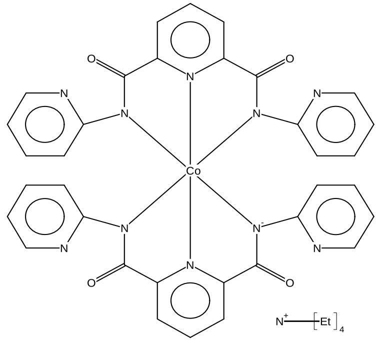

$N^+[Et]_4$

H_2O

BIWDUH

Reference: A.Mishra, A.Ali, S.Upreti, R.Gupta (2008) *Inorg.Chem.* ,**47,** 154

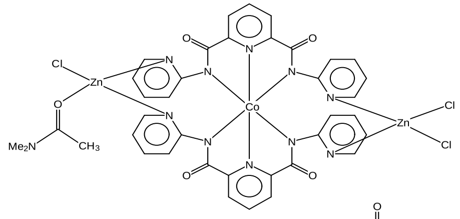

Me_2N–C(=O)–CH_3

Search: search13 (Wed May 29 14:10:34 2024): Hits 41-48

BIWGAQ

Reference: V.Niel, V.A.Milway, L.N.Dawe, H.Grove, S.S.Tandon, T.S.M.Abedin, T.L.Kelly, E.C.Spencer, J.A.K.Howard, J.L.Collins, D.O.Miller, L.K.Thompson (2008) *Inorg.Chem.* ,**47,**176

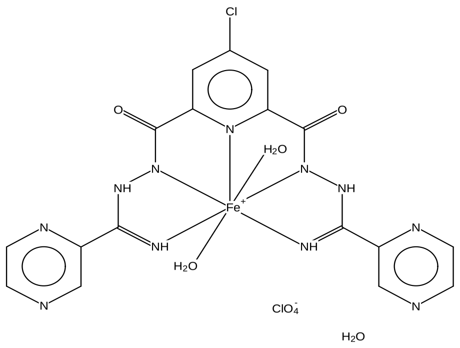

BOXDAU

Reference: D.A.Leigh, P.J.Lusby, R.T.McBurney, A.Morelli, A.M.Z.Slawin, A.R.Thomson, D.B.Walker (2009) *J.Am.Chem.Soc.* ,**131,** 3762

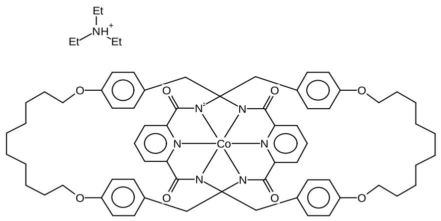

BOXDEY

Reference: D.A.Leigh, P.J.Lusby, R.T.McBurney, A.Morelli, A.M.Z.Slawin, A.R.Thomson, D.B.Walker (2009) *J.Am.Chem.Soc.* ,**131,** 3762

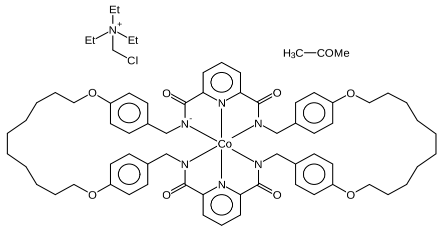

BUTJOR

Reference: Xing-Kun Xie, Xiao-Feng Zhang, Hai-Xiong Liu, Yang Wang, Chang-Neng Chen, De-Guang Huang (2015) *Jiegou Huaxue(Chin.)(Chin.J.Struct.Chem.)* ,**34,**1689

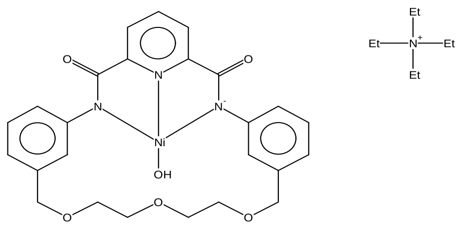

CENMAL

Reference: G.Kumar, H.Aggarwal, R.Gupta (2013) *Cryst.Growth Des.* , **13,**74

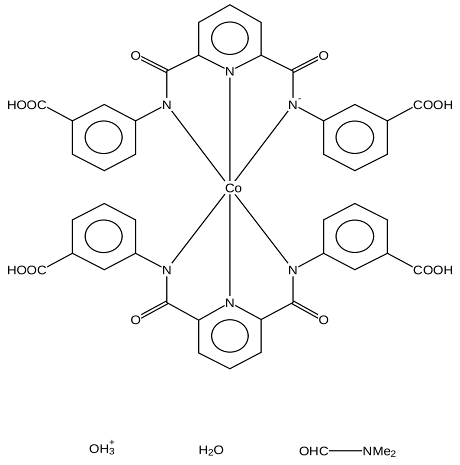

CENMEP

Reference: G.Kumar, H.Aggarwal, R.Gupta (2013) *Cryst.Growth Des.* , **13,**74

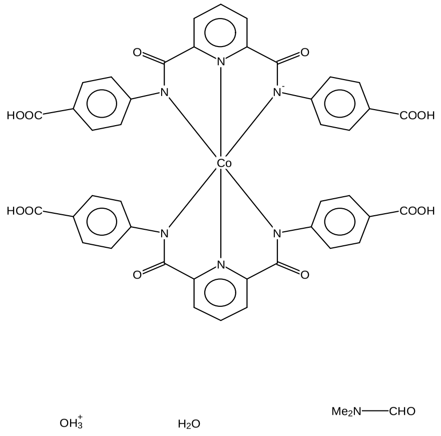

CENMIT

Reference: G.Kumar, H.Aggarwal, R.Gupta (2013) *Cryst.Growth Des.* , **13,**74

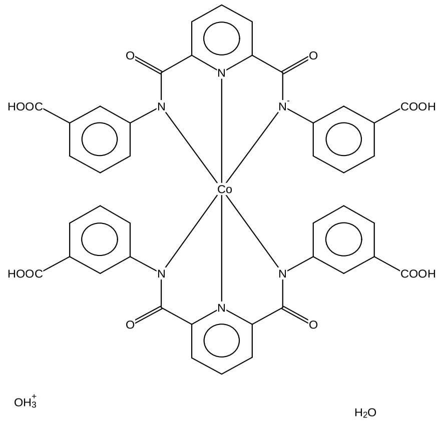

CENMOZ

Reference: G.Kumar, H.Aggarwal, R.Gupta (2013) *Cryst.Growth Des.* , **13,**74

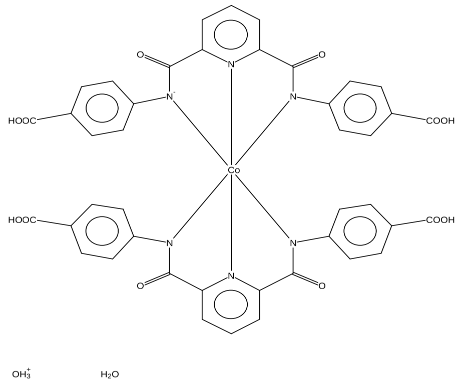

CENMUF

Reference: G.Kumar, H.Aggarwal, R.Gupta (2013) *Cryst.Growth Des.* , **13**,74

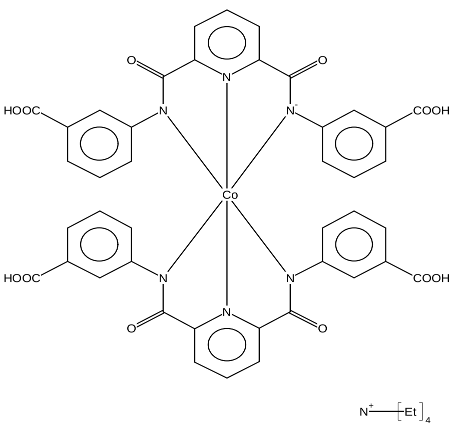

CENNAM

Reference: G.Kumar, H.Aggarwal, R.Gupta (2013) *Cryst.Growth Des.* , **13**,74

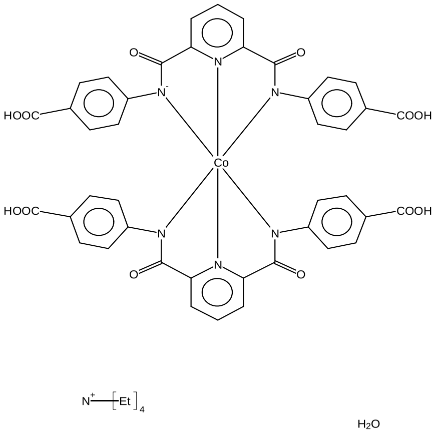

CEXYIQ

Reference: Sung-Ok Kang, T.S.Johnson, V.W.Day, K.Bowman-James (2018) *Supramol.Chem.* ,**30**,305

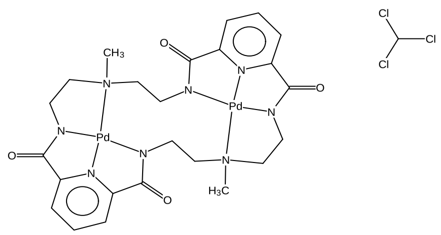

CIGPAJ

Reference: J.C.Noveron, M.M.Olmstead, P.K.Mascharak (1999) *J.Am.Chem.Soc.* ,**121**,3553

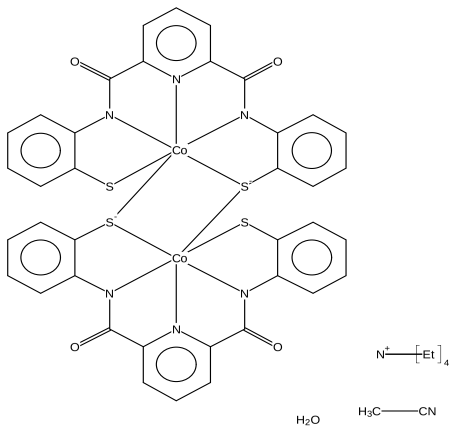

CIGPAJ01

Reference: L.A.Tyler, J.C.Noveron, M.M.Olmstead, P.K.Mascharak (2003) *Inorg.Chem.* ,**42**,5751

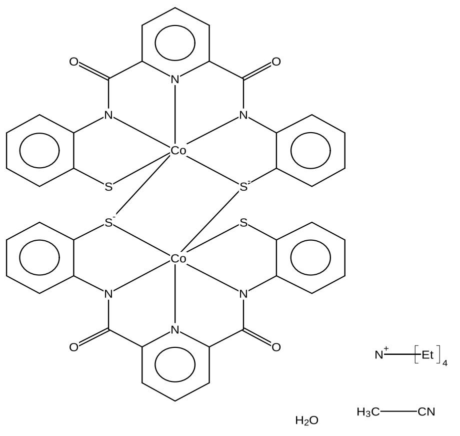

CIGQAK

Reference: J.C.Noveron, M.M.Olmstead, P.K.Mascharak (1999) *J.Am.Chem.Soc.* ,**121**,3553

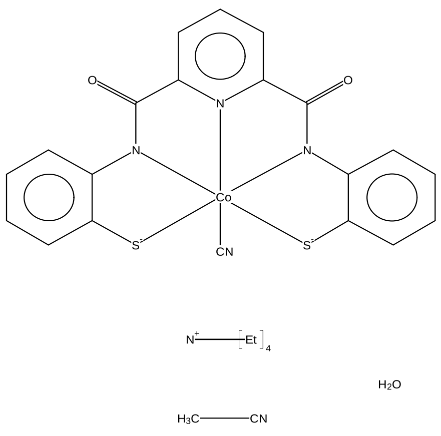

CIGQAK01

Reference: L.A.Tyler, J.C.Noveron, M.M.Olmstead, P.K.Mascharak (2003) *Inorg.Chem.* ,**42**,5751

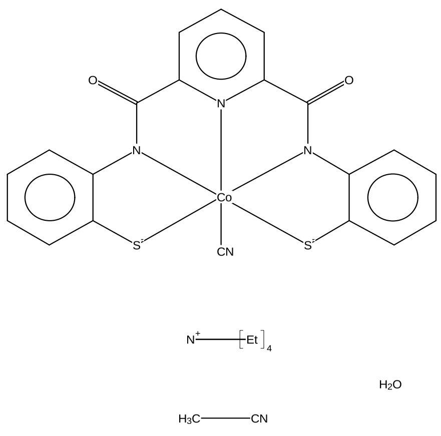

CITDAL

Reference: M.Dasgupta, S.Nag, G.Das, M.Nethaji, S.Bhattacharya (2008) *Polyhedron* ,**27**,139

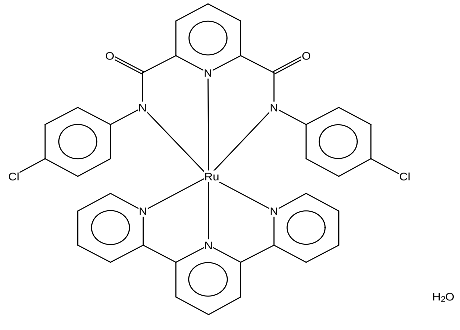

CITDEP

Reference: M.Dasgupta, S.Nag, G.Das, M.Nethaji, S.Bhattacharya (2008) *Polyhedron* ,**27,**139

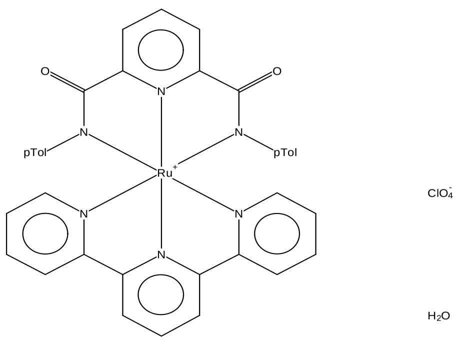

CITGIW

Reference: Xiao-Qing Shen, Hong-Chang Yao, Rui Yang, Zhong-Jun Li, Hong-Yun Zhang, Ben-Lai Wu, Hong-Wei Hou (2008) *Polyhedron* ,**27,**203

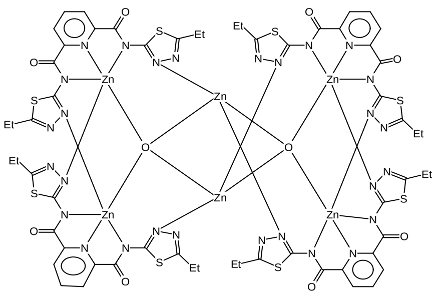

CITGOC

Reference: Xiao-Qing Shen, Hong-Chang Yao, Rui Yang, Zhong-Jun Li, Hong-Yun Zhang, Ben-Lai Wu, Hong-Wei Hou (2008) *Polyhedron* ,**27,**203

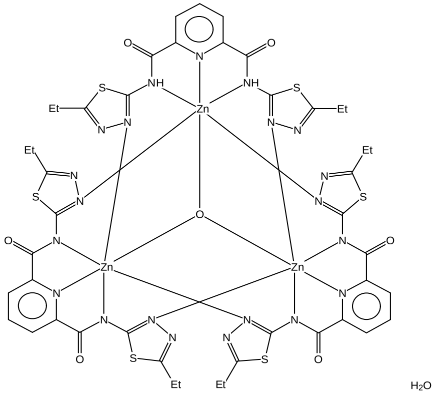

CITGUI

Reference: Xiao-Qing Shen, Hong-Chang Yao, Rui Yang, Zhong-Jun Li, Hong-Yun Zhang, Ben-Lai Wu, Hong-Wei Hou (2008) *Polyhedron* ,**27,**203

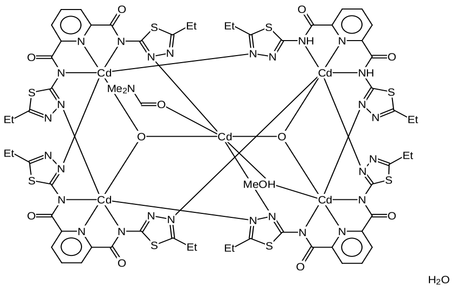

COHHIR

Reference: T.Moriuchi, Y.Takagi, T.Hirao (2008) *Eur.J.Inorg.Chem.* , 3877

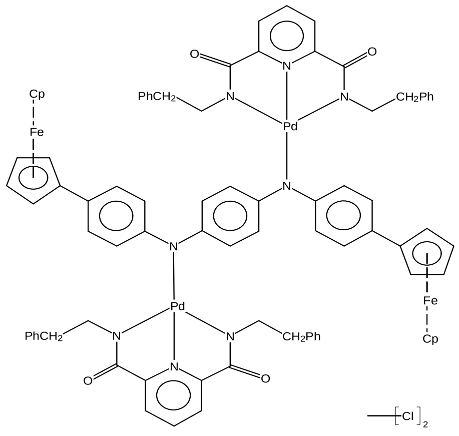

DAJGON

Reference: P.Pirovano, E.R.Farquhar, M.Swart, A.R.McDonald (2016) *J.Am.Chem.Soc.* ,**138,**14362

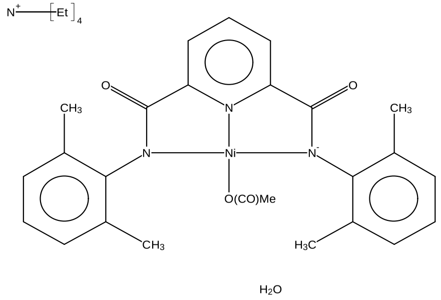

DAJGUT

Reference: P.Pirovano, E.R.Farquhar, M.Swart, A.R.McDonald (2016) *J.Am.Chem.Soc.* ,**138,**14362

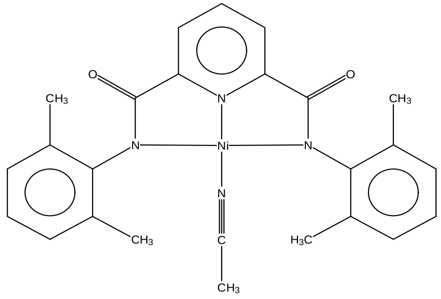

DAJHAA

Reference: P.Pirovano, E.R.Farquhar, M.Swart, A.R.McDonald (2016) *J.Am.Chem.Soc.* ,**138,**14362

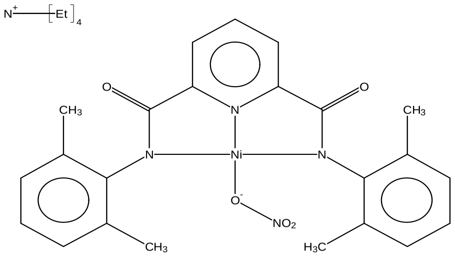

DAWXAC

Reference: A.Ali, G.Hundal, R.Gupta (2012) *Cryst.Growth Des.* ,**12,** 1308

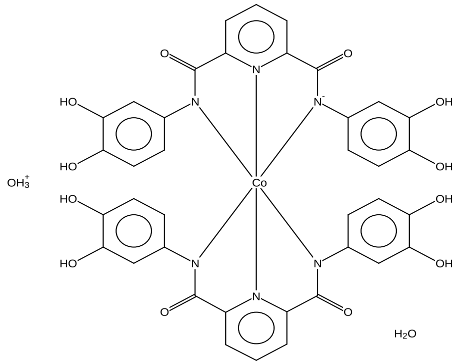

DAWXEG

Reference: A.Ali, G.Hundal, R.Gupta (2012) *Cryst.Growth Des.* ,**12,** 1308

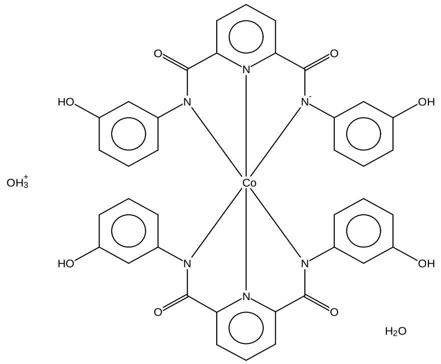

DAWXIK

Reference: A.Ali, G.Hundal, R.Gupta (2012) *Cryst.Growth Des.* ,**12,** 1308

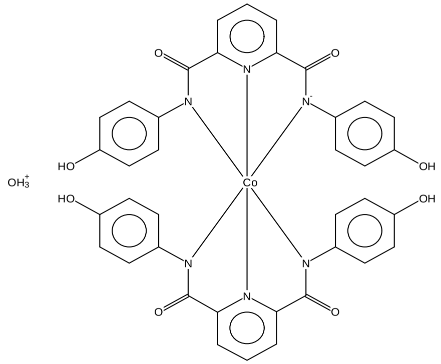

DAWXOQ

Reference: A.Ali, G.Hundal, R.Gupta (2012) *Cryst.Growth Des.* ,**12,** 1308

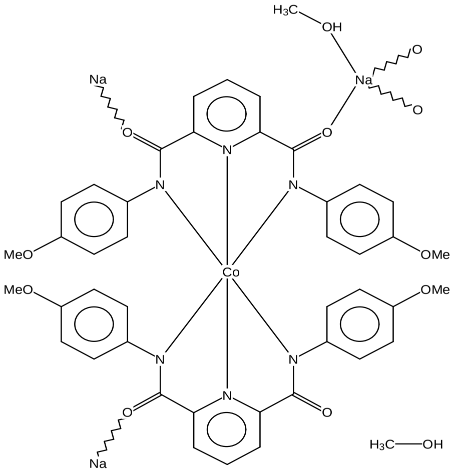

DAWXUW

Reference: A.Ali, G.Hundal, R.Gupta (2012) *Cryst.Growth Des.* ,**12,** 1308

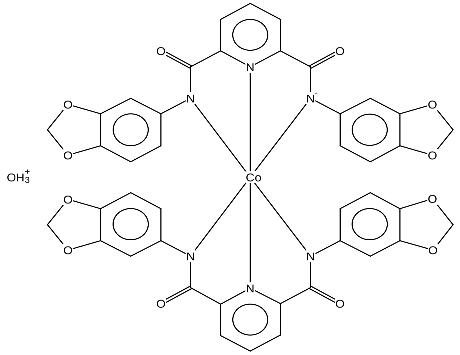

DOGKER

Reference: Qi-Qiang Wang, R.A.Begum, V.W.Day, K.Bowman-James (2013) *J.Am.Chem.Soc.* ,**135,**17193

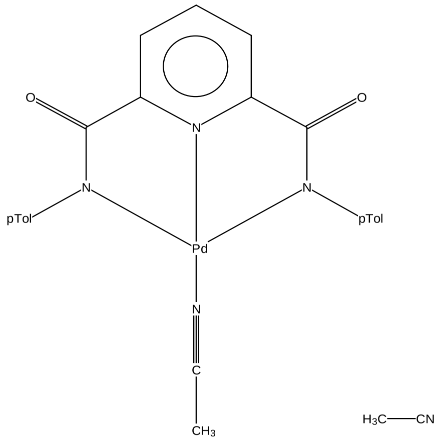

DOGKIV

Reference: Qi-Qiang Wang, R.A.Begum, V.W.Day, K.Bowman-James (2013) *J.Am.Chem.Soc.* ,**135,**17193

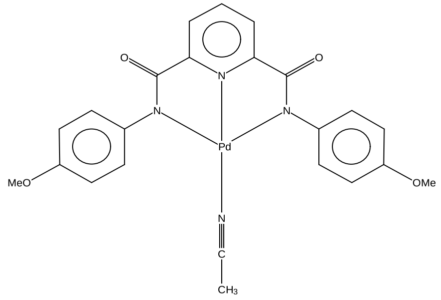

DOGKOB

Reference: Qi-Qiang Wang, R.A.Begum, V.W.Day, K.Bowman-James (2013) *J.Am.Chem.Soc.* ,**135,**17193

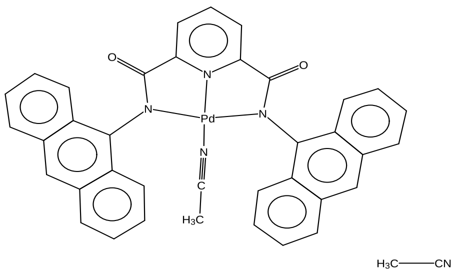

DOGKUH

Reference: Qi-Qiang Wang, R.A.Begum, V.W.Day, K.Bowman-James (2013) *J.Am.Chem.Soc.* ,**135,**17193

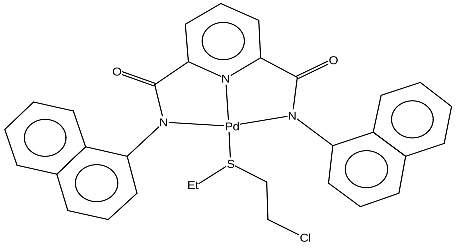

DOGLAO

Reference: Qi-Qiang Wang, R.A.Begum, V.W.Day, K.Bowman-James (2013) *J.Am.Chem.Soc.* ,**135,**17193

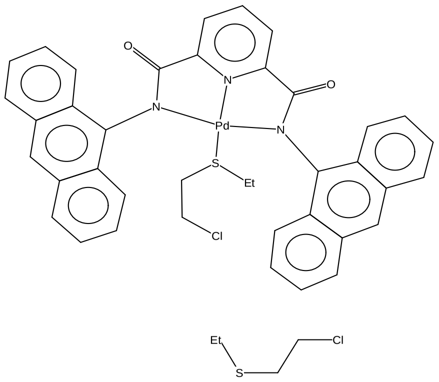

DOHQID

Reference: S.Pandey, G.Kumar, R.Gupta (2019) *Cryst.Growth Des.* , **19,**2723

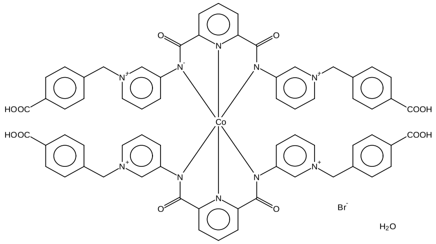

DOHQOJ

Reference: S.Pandey, G.Kumar, R.Gupta (2019) *Cryst.Growth Des.* , **19,**2723

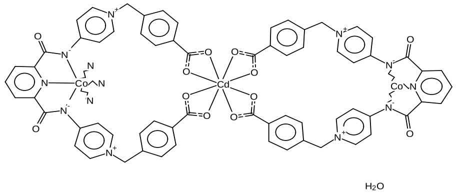

DOHQUP

Reference: S.Pandey, G.Kumar, R.Gupta (2019) *Cryst.Growth Des.* , **19,**2723

n-ZnCo[μ_2-$C_{33}H_{23}N_5O_6$-κ^3N,N',N'',κ^2O,O']$_2^+$

Br^-

H_2O

DOHRAW

Reference: S.Pandey, G.Kumar, R.Gupta (2019) *Cryst.Growth Des.* , **19,**2723

n-CoMn[μ_2-$C_{33}H_{23}N_5O_6$-κ^3N,N',N'',κ^2O,O']$_2$[H_2O]$_2^+$

Br^-

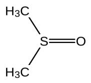

H_2O

DOHREA

Reference: S.Pandey, G.Kumar, R.Gupta (2019) *Cryst.Growth Des.* , **19,**2723

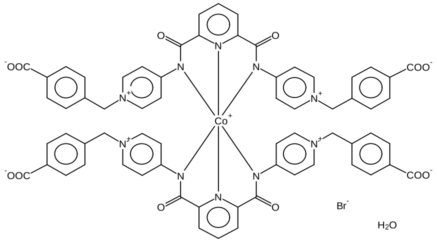

DOHRIE

Reference: S.Pandey, G.Kumar, R.Gupta (2019) *Cryst.Growth Des.* , **19,**2723

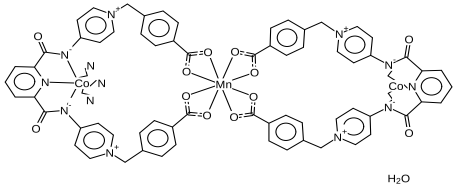

DOHVAZ

Reference: A.Adhikary, J.A.Sheikh, A.D.Konar, S.Konar (2014) *RSC Advances* ,**4**,12408

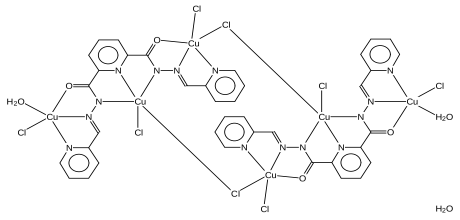

DOWRUF

Reference: D.Unjaroen, R.Gericke, M.Lovisari, D.Nelis, P.Mondal, P.Pirovano, B.Twamley, E.R.Farquhar, A.R.McDonald (2019) *Inorg.Chem.* ,**58**,16838

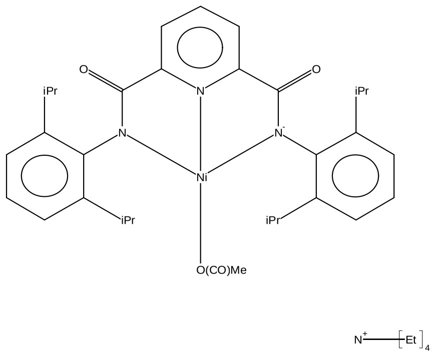

DOWSAM

Reference: D.Unjaroen, R.Gericke, M.Lovisari, D.Nelis, P.Mondal, P.Pirovano, B.Twamley, E.R.Farquhar, A.R.McDonald (2019) *Inorg.Chem.* ,**58**,16838

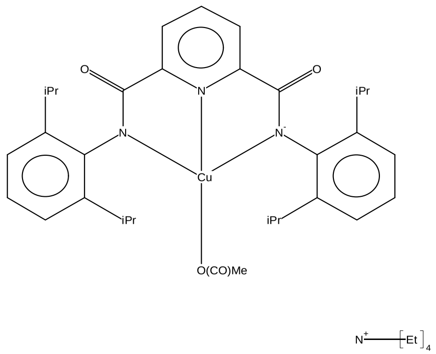

DURSEQ

Reference: P.Kumar, V.Kumar, R.Gupta (2015) *RSC Advances* ,**5**, 97874

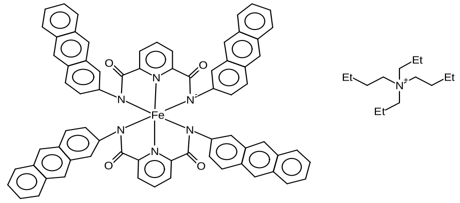

DUVBIH

Reference: B.D.Neisen, P.V.Solntsev, M.R.Halvagar, W.B.Tolman (2015) *Eur.J.Inorg.Chem.* ,5856

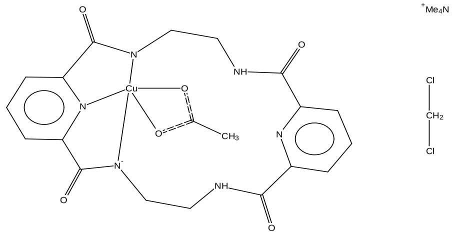

DUVBON

Reference: B.D.Neisen, P.V.Solntsev, M.R.Halvagar, W.B.Tolman (2015) *Eur.J.Inorg.Chem.* ,5856

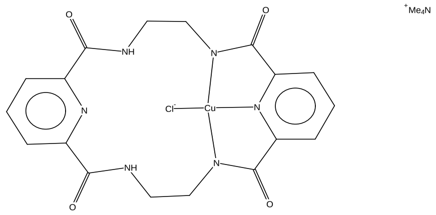

DUVBUT

Reference: B.D.Neisen, P.V.Solntsev, M.R.Halvagar, W.B.Tolman (2015) *Eur.J.Inorg.Chem.* ,5856

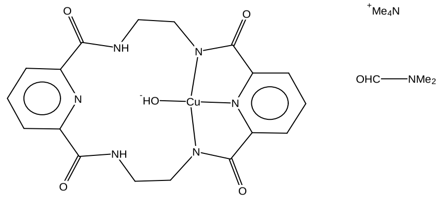

DUVCAA

Reference: B.D.Neisen, P.V.Solntsev, M.R.Halvagar, W.B.Tolman (2015) *Eur.J.Inorg.Chem.* ,5856

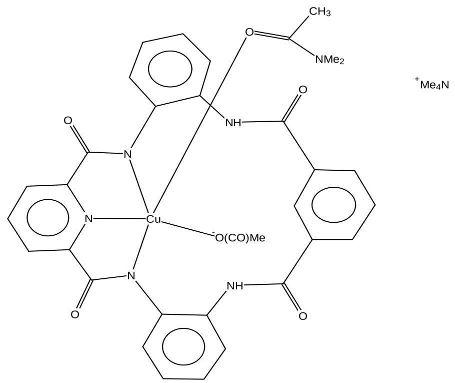

DUVCEE

Reference: B.D.Neisen, P.V.Solntsev, M.R.Halvagar, W.B.Tolman (2015) *Eur.J.Inorg.Chem.* ,5856

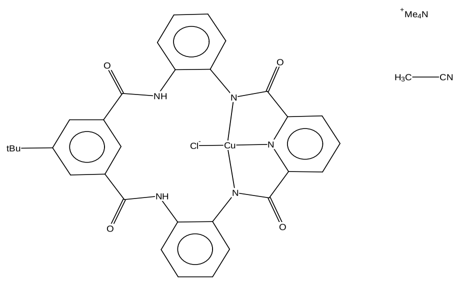

DUVQAO

Reference: B.D.Neisen, P.V.Solntsev, M.R.Halvagar, W.B.Tolman (2015) *Eur.J.Inorg.Chem.* ,5856

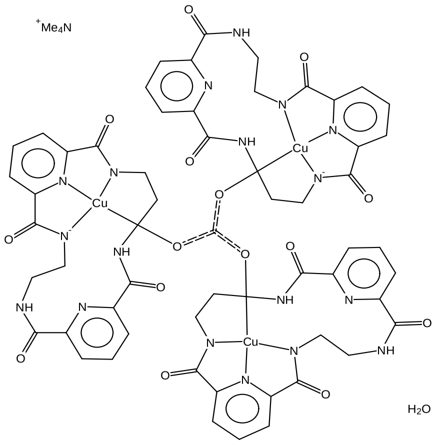

DUVQES

Reference: B.D.Neisen, P.V.Solntsev, M.R.Halvagar, W.B.Tolman (2015) *Eur.J.Inorg.Chem.* ,5856

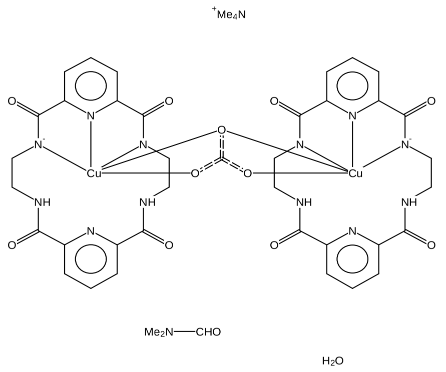

EBIHOP

Reference: K.Ghosh, N.Tyagi, O.Singh (2016) *CSD Communication(Private Communication)* ,

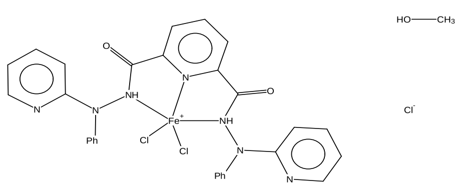

EBIHOP01

Reference: N.Tyagi, O.Singh, K.Ghosh (2017) *Catalysis Communications* ,**95**,83

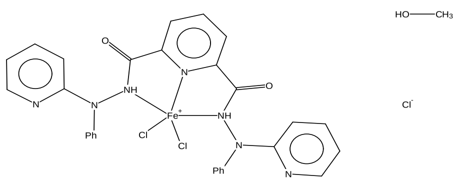

EFAFID

Reference: Fangfang Chen, Ni Wang, H.Lei, Dingyi Guo, Hongfei Liu, Zongyao Zhang, Wei Zhang, Wenzhen Lai, Rui Cao (2017) *Inorg.Chem.* ,**56**,13368

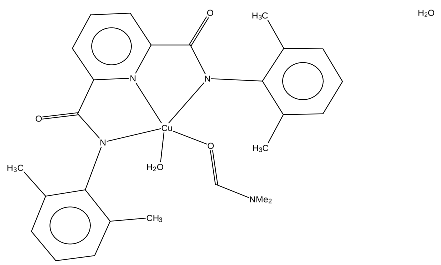

EFAFOJ

Reference: Fangfang Chen, Ni Wang, H.Lei, Dingyi Guo, Hongfei Liu, Zongyao Zhang, Wei Zhang, Wenzhen Lai, Rui Cao (2017) *Inorg.Chem.* ,**56**,13368

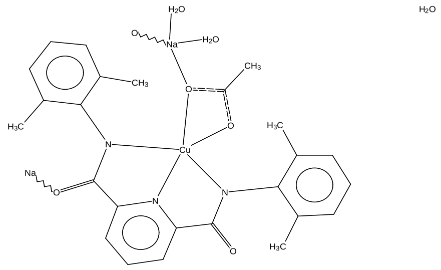

EFAJIE

Reference: D.S.Marlin, M.M.Olmstead, P.K.Mascharak (2001) *Angew.Chem.,Int.Ed.* ,**40**,4752

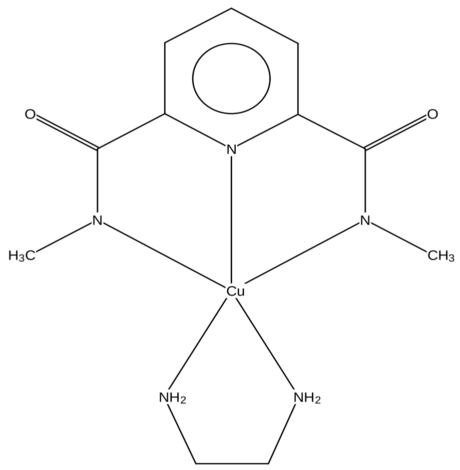

EFAKAA

Reference: Fangfang Chen, Ni Wang, H.Lei, Dingyi Guo, Hongfei Liu, Zongyao Zhang, Wei Zhang, Wenzhen Lai, Rui Cao (2017) *Inorg.Chem.* ,**56,**13368

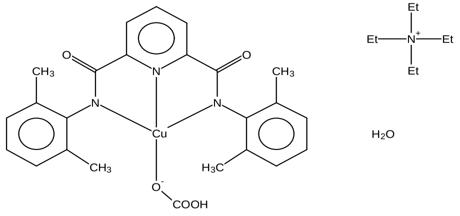

EGEKAF

Reference: D.Bansal, A.Mondal, N.Lakshminarasimhan, R.Gupta (2019) *Dalton Trans.* ,**48,**7918

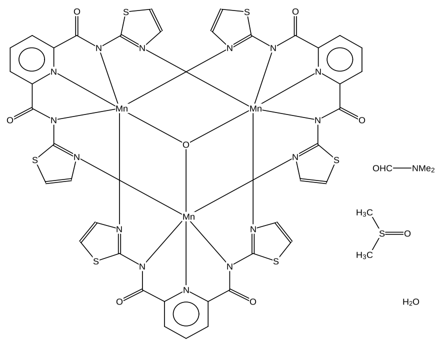

EGEKEJ

Reference: D.Bansal, A.Mondal, N.Lakshminarasimhan, R.Gupta (2019) *Dalton Trans.* ,**48,**7918

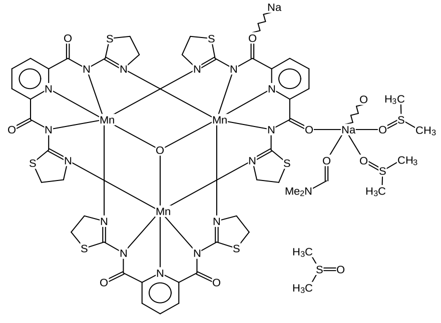

EGEKIN

Reference: D.Bansal, A.Mondal, N.Lakshminarasimhan, R.Gupta (2019) *Dalton Trans.* ,**48,**7918

$Mn_4[\mu_4\text{-}O]$--
--$[\mu_3\text{-}C_{21}H_{13}N_7O_2\text{-}\kappa^5N,N',N'',N''',N'''']_3[HOMe]$

OHC——NMe$_2$

EXEFIW

Reference: V.Maurizot, G.Linti, I.Huc (2004) *Chem.Commun.* ,924

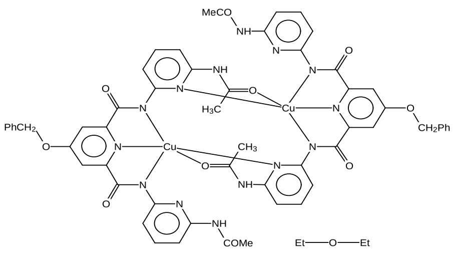

EXEFOC

Reference: V.Maurizot, G.Linti, I.Huc (2004) *Chem.Commun.* ,924

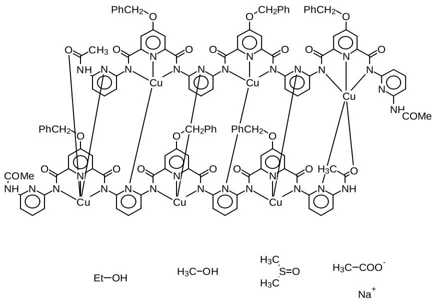

EYIXAM

Reference: Su-Ni Qin, Zi-Lu Chen, Dong-Cheng Liu, Wan-Yun Huang, Fu-Pei Liang (2011) *Transition Met.Chem.* ,**36,**369

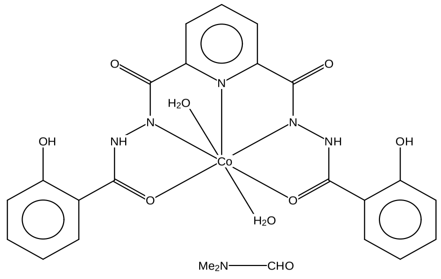

EYIXEQ

Reference: Su-Ni Qin, Zi-Lu Chen, Dong-Cheng Liu, Wan-Yun Huang, Fu-Pei Liang (2011) *Transition Met.Chem.* ,**36,**369

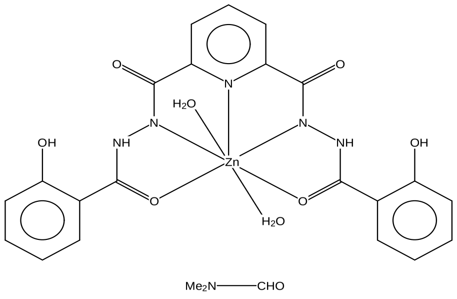

EYIXIU

Reference: Su-Ni Qin, Zi-Lu Chen, Dong-Cheng Liu, Wan-Yun Huang, Fu-Pei Liang (2011) *Transition Met.Chem.* ,**36**,369

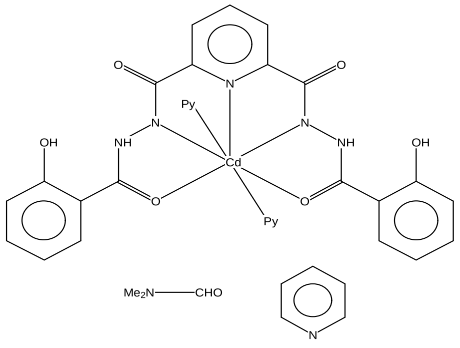

EYIXOA

Reference: Su-Ni Qin, Zi-Lu Chen, Dong-Cheng Liu, Wan-Yun Huang, Fu-Pei Liang (2011) *Transition Met.Chem.* ,**36**,369

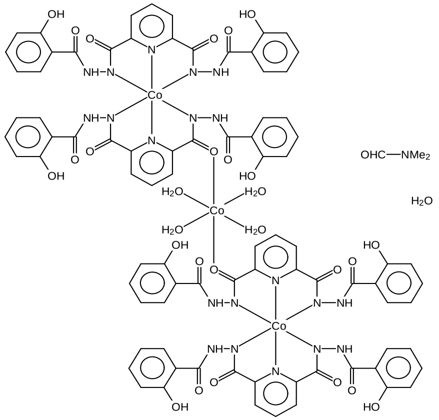

FECWOA

Reference: Y.Furusho, T.Matsuyama, T.Takata, T.Moriuchi, T.Hirao (2004) *Tetrahedron Lett.* ,**45**,9593

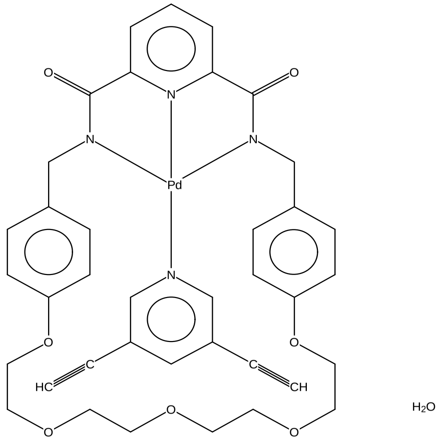

FEDYUI

Reference: F.A.Chavez, J.M.Rowland, M.M.Olmstead, P.K.Mascharak (1998) *J.Am.Chem.Soc.* ,**120**,9015

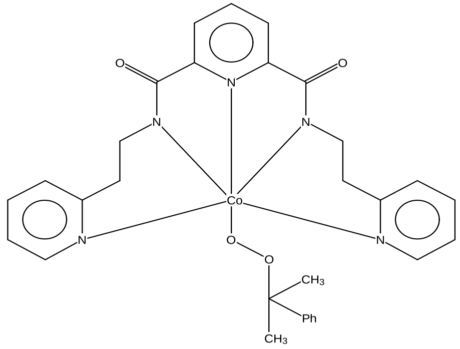

FEFBOH

Reference: F.A.Chavez, J.M.Rowland, M.M.Olmstead, P.K.Mascharak (1998) *J.Am.Chem.Soc.* ,**120**,9015

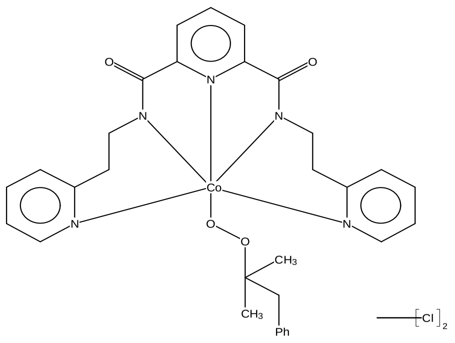

FEFBUN

Reference: F.A.Chavez, J.M.Rowland, M.M.Olmstead, P.K.Mascharak (1998) *J.Am.Chem.Soc.* ,**120**,9015

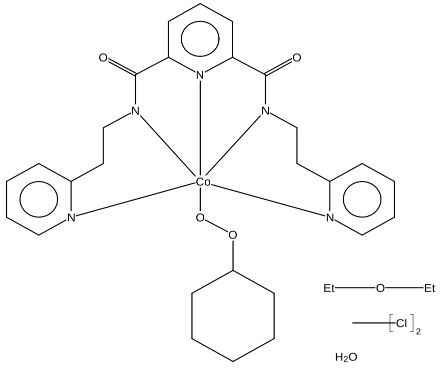

FEFDUP

Reference: F.A.Chavez, J.M.Rowland, M.M.Olmstead, P.K.Mascharak (1998) *J.Am.Chem.Soc.* ,**120**,9015

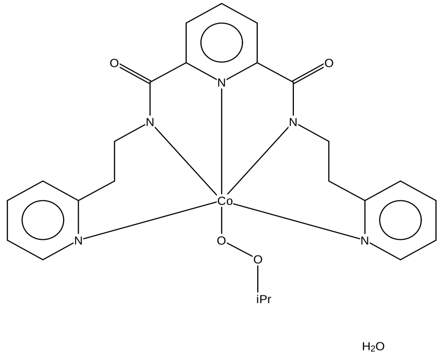

FEFFAX

Reference: F.A.Chavez, J.M.Rowland, M.M.Olmstead, P.K.Mascharak (1998) *J.Am.Chem.Soc.* ,**120**,9015

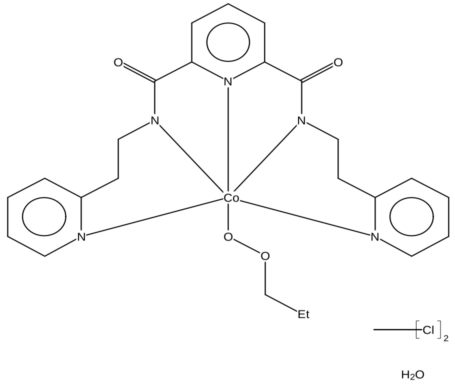

FEFFIF

Reference: F.A.Chavez, J.M.Rowland, M.M.Olmstead, P.K.Mascharak (1998) *J.Am.Chem.Soc.* ,**120**,9015

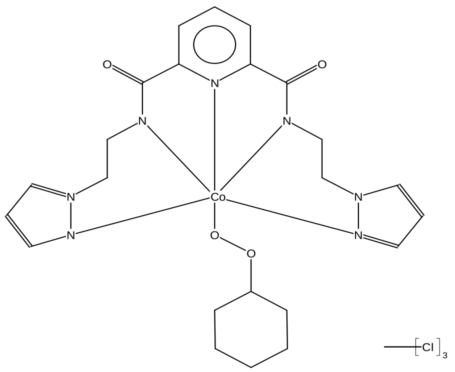

FEMLIV

Reference: S.Srivastava, R.Gupta (2016) *Chem. Sel.* ,**1**,6167

Co_2Na_2--
--[μ_3-$C_{21}H_{13}N_3O_6(COO)_2$-$\kappa^3$N,N',N'',$\kappa^2$O,O']$_2$[$\mu_2$-$C_{19}H_{13}N_3O_2(COO)_4$-$\kappa^3$N,N',N'',$\kappa^1$O]$_2$[CHO(NMe$_2$)]$_6$

FEMLUH

Reference: S.Srivastava, R.Gupta (2016) *Chem. Sel.* ,**1**,6167

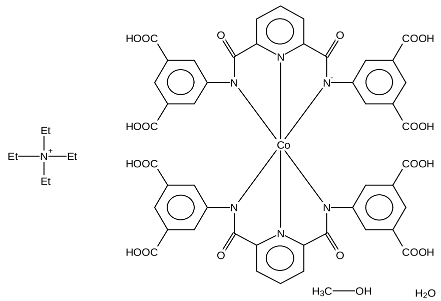

FEMQUM

Reference: S.Srivastava, R.Gupta (2016) *Chem. Sel.* ,**1**,6167

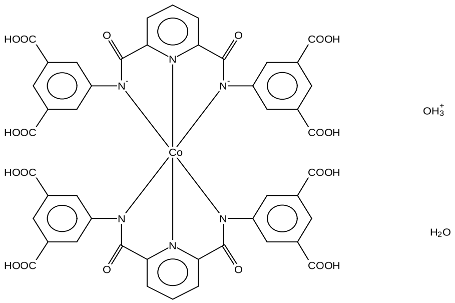

FIKTID

Reference: N.W.Alcock, G.Clarkson, P.B.Glover, G.A.Lawrance, P.Moore, M.Napitupulu (2005) *Dalton Trans.* ,518

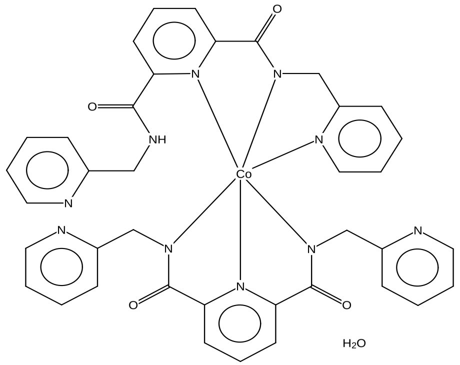

FIKTUP

Reference: N.W.Alcock, G.Clarkson, P.B.Glover, G.A.Lawrance, P.Moore, M.Napitupulu (2005) *Dalton Trans.* ,518

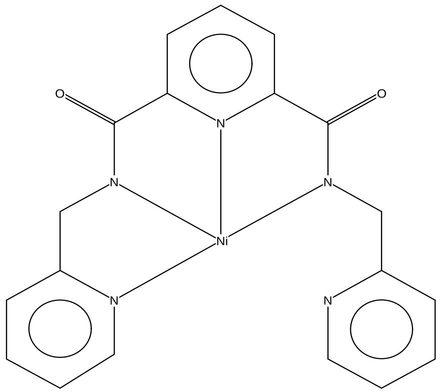

FIKVAX

Reference: N.W.Alcock, G.Clarkson, P.B.Glover, G.A.Lawrance, P.Moore, M.Napitupulu (2005) *Dalton Trans.* ,518

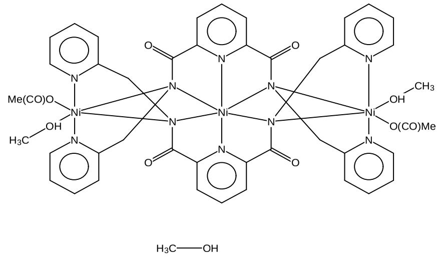

FIKVEB

Reference: N.W.Alcock, G.Clarkson, P.B.Glover, G.A.Lawrance, P.Moore, M.Napitupulu (2005) *Dalton Trans.* ,518

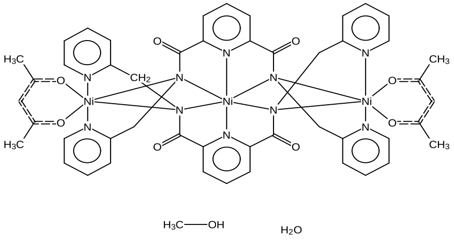

FIKVIF

Reference: N.W.Alcock, G.Clarkson, P.B.Glover, G.A.Lawrance, P.Moore, M.Napitupulu (2005) *Dalton Trans.* ,518

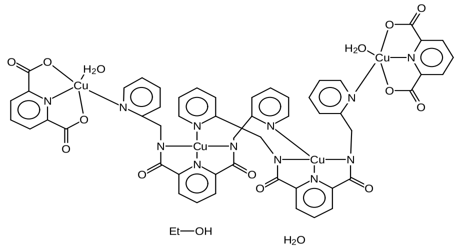

FIRBIT

Reference: K.Ghosh, S.Kumar, R.Kumar, U.P.Singh (2014) *J.Organomet.Chem.* ,**750**,169

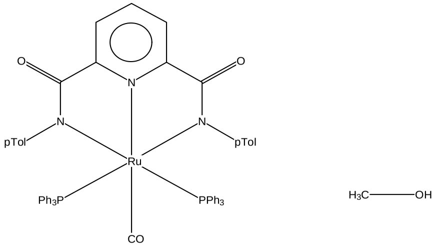

FIVKAZ

Reference: V.Carta, M.A.Soto, M.J.MacLachlan (2019) *Chem.Commun.* ,**55**,1245

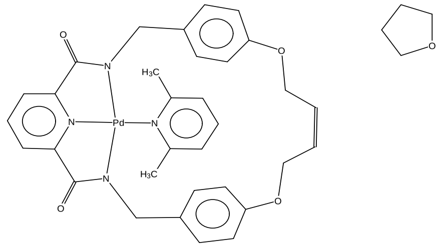

FIVKED

Reference: V.Carta, M.A.Soto, M.J.MacLachlan (2019) *Chem.Commun.* ,**55**,1245

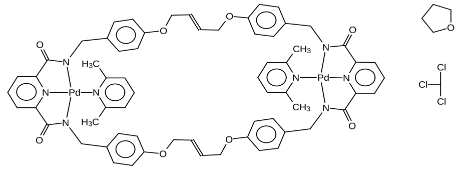

FOJHOD

Reference: J.E.Beves, V.Blanco, B.A.Blight, R.Carrillo, D.M.DSouza, D.Howgego, D.A.Leigh, A.M.Z.Slawin, M.D.Symes (2014) *J.Am.Chem.Soc.* ,**136**,2094

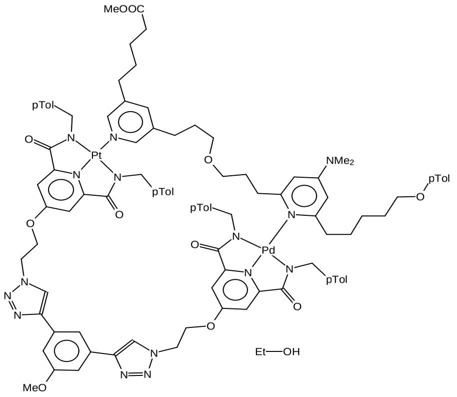

FOZGEJ

Reference: D.Bansal, R.Gupta (2019) *Dalton Trans.* ,**48**,14737

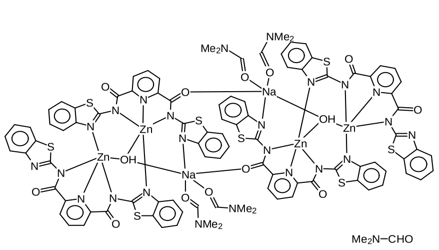

FOZGIN

Reference: D.Bansal, R.Gupta (2019) *Dalton Trans.* ,**48**,14737

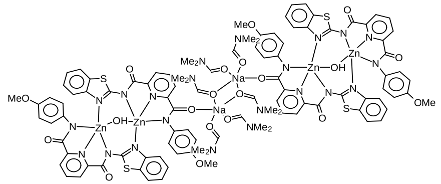

FOZGOT

Reference: D.Bansal, R.Gupta (2019) *Dalton Trans.* ,**48**,14737

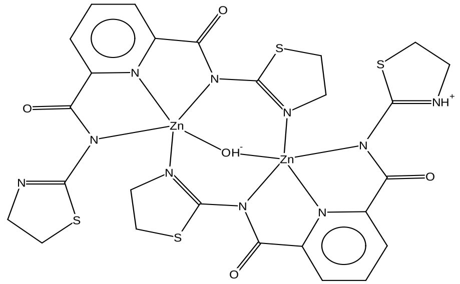

FOZGUZ

Reference: D.Bansal, R.Gupta (2019) *Dalton Trans.* ,**48,**14737

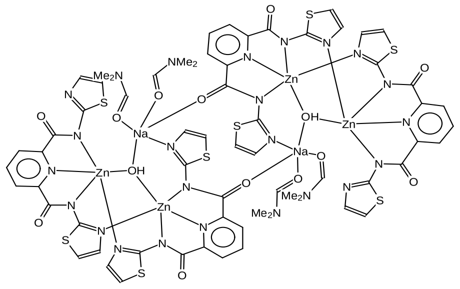

FUTLOW

Reference: Jie Wu, Hong-Wei Hou, Yue-Xin Guo, Yao-Ting Fan, Xia Wang (2009) *Eur.J.Inorg.Chem.* ,2796

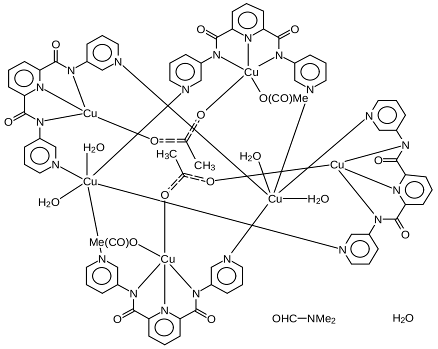

GAMQIV

Reference: H.A.Burkill, N.Robertson, R.Vilar, A.J.P.White, D.J.Williams (2005) *Inorg.Chem.* ,**44**,3337

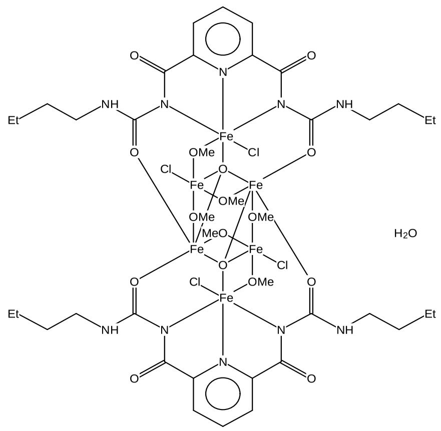

GAMQOB

Reference: H.A.Burkill, N.Robertson, R.Vilar, A.J.P.White, D.J.Williams (2005) *Inorg.Chem.* ,**44**,3337

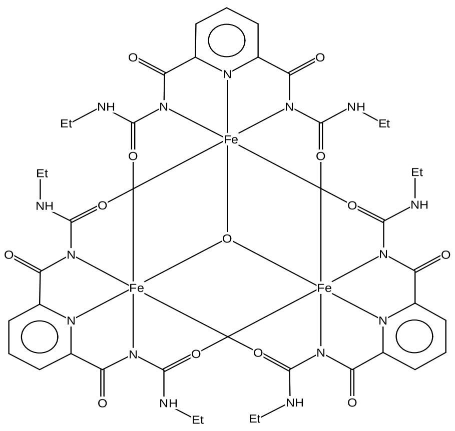

GAPNUH

Reference: T.L.Kelly, V.A.Milway, H.Grove, V.Niel, T.S.M.Abedin, L.K.Thompson, Liang Zhao, R.G.Harvey, D.O.Miller, M.Leech, A.E.Goeta, J.A.K.Howard (2005) *Polyhedron* ,**24,**807

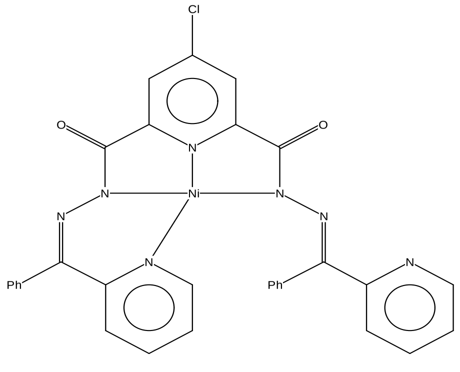

GEDVUJ

Reference: A.D.Spaeth, N.L.Gagnon, Debanjan Dhar, G.M.Yee, W.B.Tolman (2017) *J.Am.Chem.Soc.* ,**139,**4477

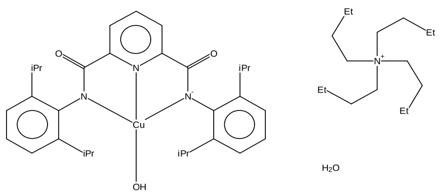

GICJAD

Reference: Qiang Yu, T.E.Baroni, L.Liable-Sands, A.L.Rheingold, A.S.Borovik (1998) *Tetrahedron Lett.* ,**39,**6831

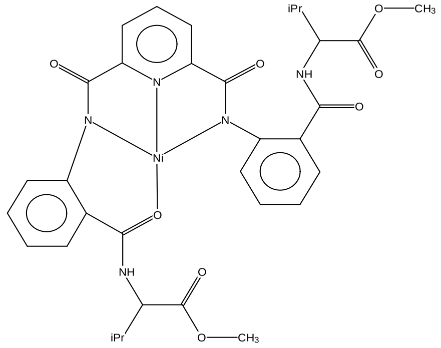

GICRUH

Reference: Qi-Qiang Wang, R.A.Begum, V.W.Day, K.Bowman-James (2013) *Polyhedron* ,**52,**515

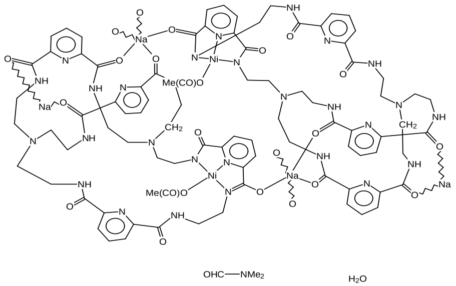

GIGLEQ

Reference: C.N.Noufele, Chien Thang Pham, A.Hagenbach, U.Abram (2018) *Inorg.Chem.* ,**57,**12255

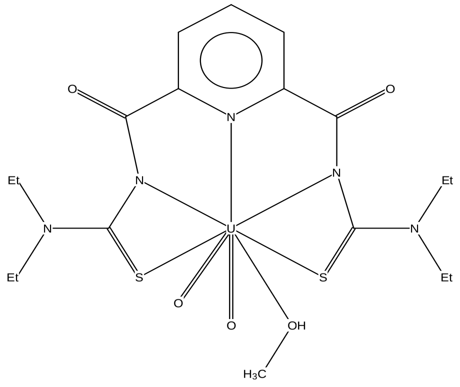

GIGLIU

Reference: C.N.Noufele, Chien Thang Pham, A.Hagenbach, U.Abram (2018) *Inorg.Chem.* ,**57,**12255

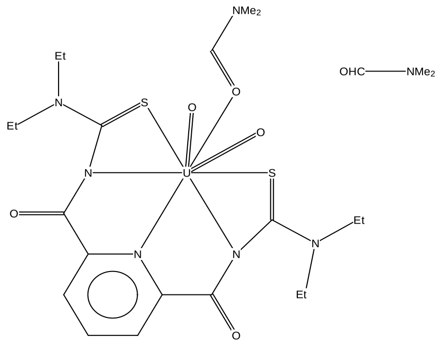

GIGLOA

Reference: C.N.Noufele, Chien Thang Pham, A.Hagenbach, U.Abram (2018) *Inorg.Chem.* ,**57,**12255

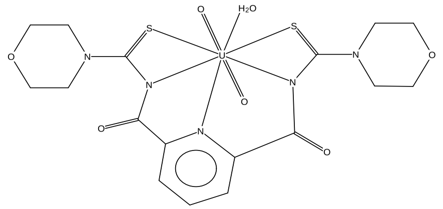

GIGLUG

Reference: C.N.Noufele, Chien Thang Pham, A.Hagenbach, U.Abram (2018) *Inorg.Chem.* ,**57,**12255

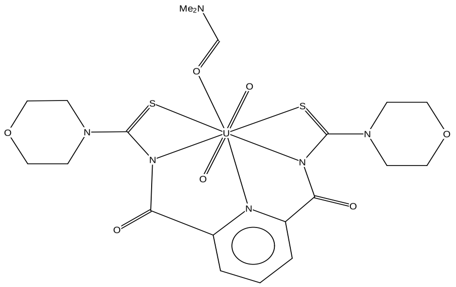

GOGZEH

Reference: A.K.Patra, R.Mukherjee (1999) *Inorg.Chem.* ,**38,**1388

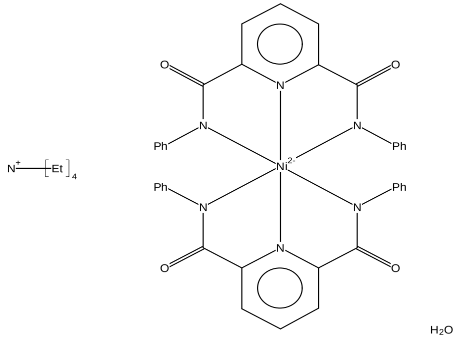

GOGZIL

Reference: A.K.Patra, R.Mukherjee (1999) *Inorg.Chem.* ,**38,**1388

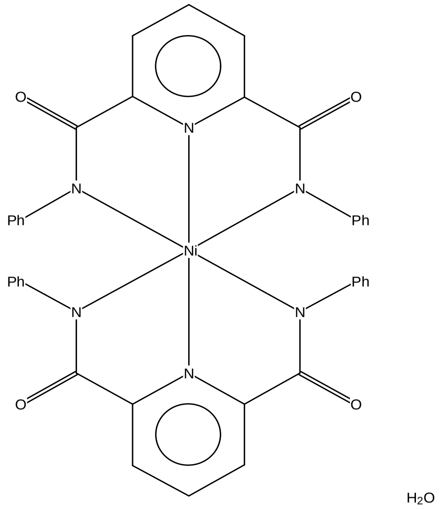

GOPRIP

Reference: M.Ballesteros II, Emily Y.Tsui (2019) *Inorg.Chem.* ,**58,** 10501

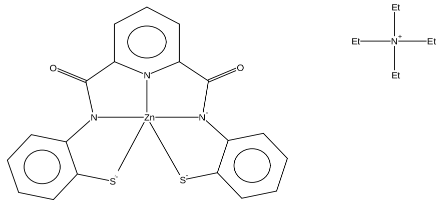

GOPROV

Reference: M.Ballesteros II, Emily Y.Tsui (2019) *Inorg.Chem.* ,**58,** 10501

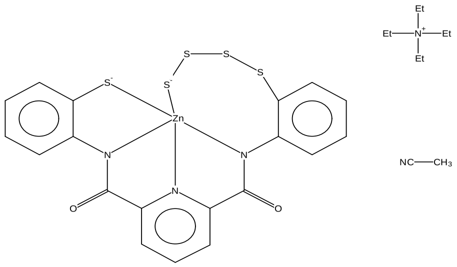

GOPRUB

Reference: M.Ballesteros II, Emily Y.Tsui (2019) *Inorg.Chem.* ,**58,** 10501

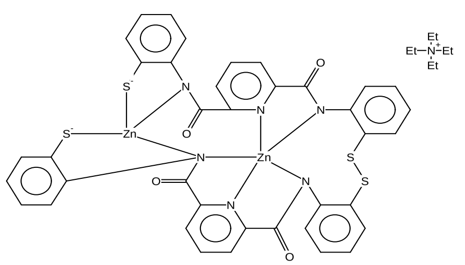

GOSGEC

Reference: Qiaoqiao Teng, Han Vinh Huynh (2015) *Chem.Commun.* , **51,**1248

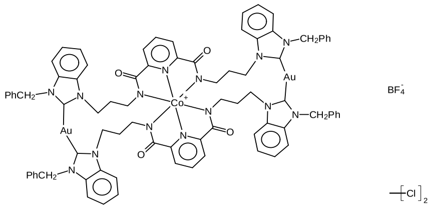

GOTDEA

Reference: G.Kumar, G.Kumar, R.Gupta (2015) *Inorg.Chim.Acta* ,**425,** 260

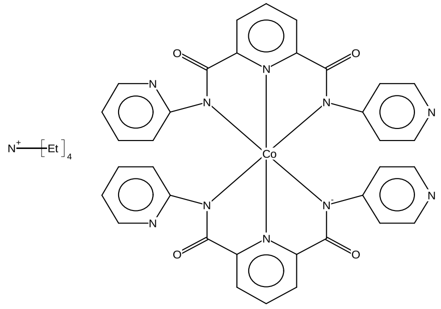

GOTDIE

Reference: G.Kumar, G.Kumar, R.Gupta (2015) *Inorg.Chim.Acta* ,**425,** 260

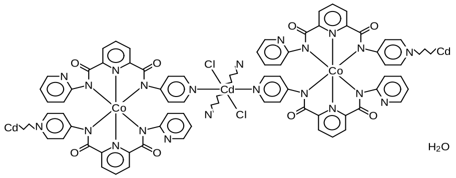

GOTDOK

Reference: G.Kumar, G.Kumar, R.Gupta (2015) *Inorg.Chim.Acta* ,**425,** 260

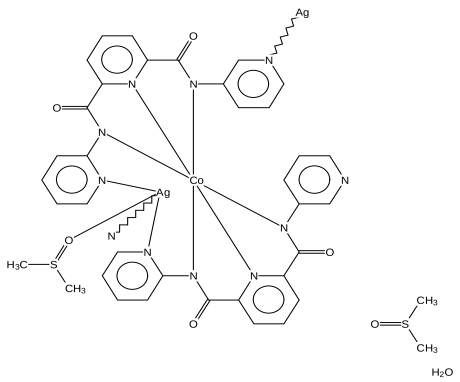

GOTDUQ

Reference: G.Kumar, G.Kumar, R.Gupta (2015) *Inorg.Chim.Acta* ,**425,** 260

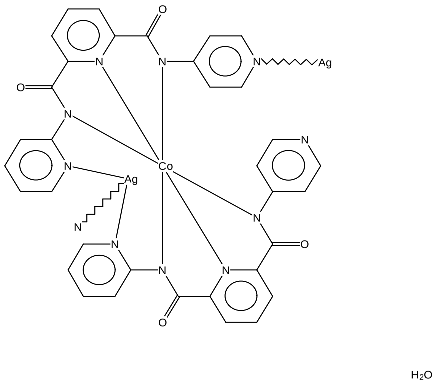

HACYUJ

Reference: S.Engbers, E.A.Trifonova, K.M.Hess, F.Vries, J.E.M.N.Klein (2021) *Eur.J.Inorg.Chem.* ,**2021,**3561

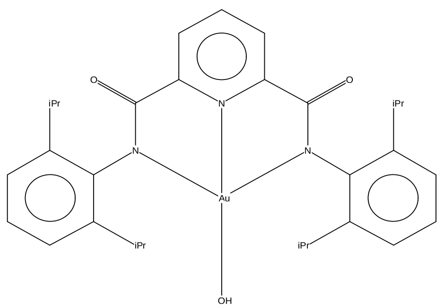

HACZAQ

Reference: S.Engbers, E.A.Trifonova, K.M.Hess, F.Vries, J.E.M.N.Klein (2021) *Eur.J.Inorg.Chem.* ,**2021,**3561

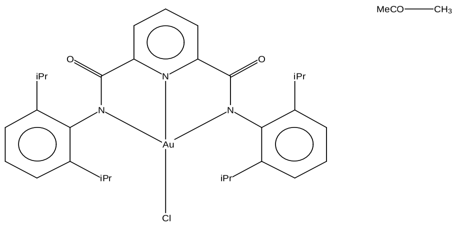

HAVVEG

Reference: Hongwei Hou, Yongli Wei, Yinglin Song, Liwei Mi, Mingsheng Tang, Linke Li, Yaoting Fan (2005) *Angew.Chem.,Int.Ed.* , **44,**6067

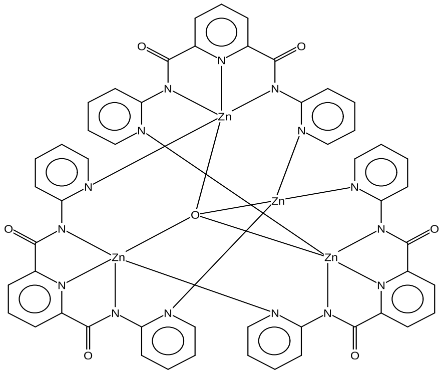

H_2O

HAZKID

Reference: D.B.Dell'Amico, F.Calderazzo, F.Di Colo, G.Guglielmetti, L.Labella, F.Marchetti (2006) *Inorg.Chim.Acta* ,**359,**127

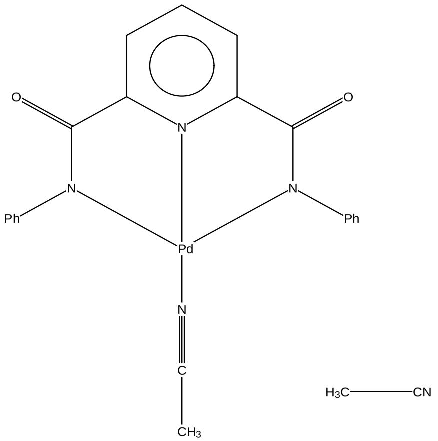

HAZKOJ

Reference: D.B.Dell'Amico, F.Calderazzo, F.Di Colo, G.Guglielmetti, L.Labella, F.Marchetti (2006) *Inorg.Chim.Acta* ,**359,**127

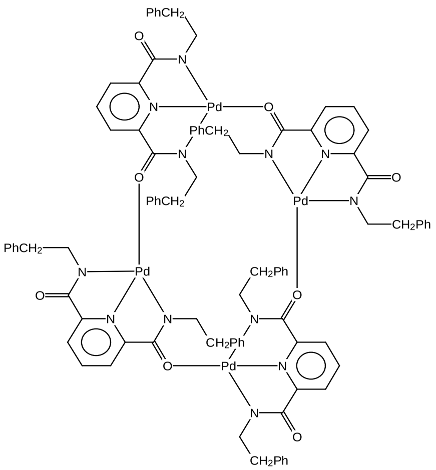

HEHCOO

Reference: G.Kumar, R.Gupta (2012) *Inorg.Chem.Commun.* ,**23,**103

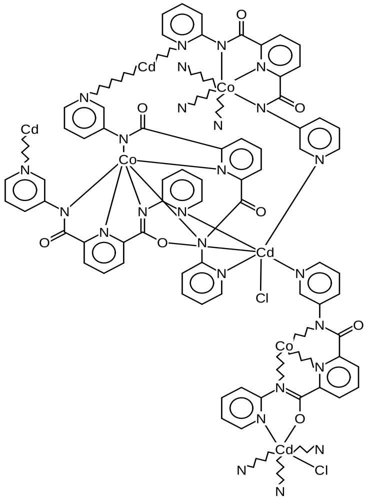

H_2O

HEHCOO01

Reference: G.Kumar, G.Kumar, R.Gupta (2015) *Inorg.Chim.Acta* ,**425,** 260

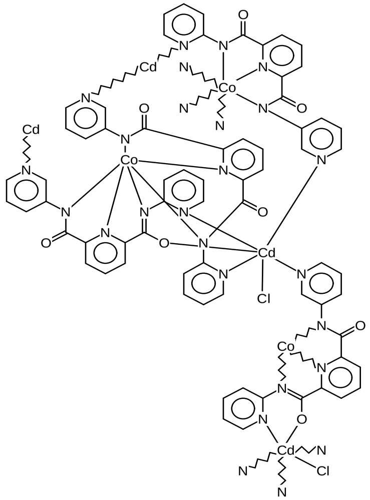

H_2O

HEHCUU

Reference: G.Kumar, R.Gupta (2012) *Inorg.Chem.Commun.* ,**23,**103

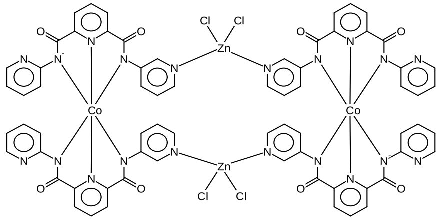

$N^+[Et]_4$ OHC—NMe$_2$

H_2O

HEHDAB

Reference: G.Kumar, R.Gupta (2012) *Inorg.Chem.Commun.* ,**23,**103

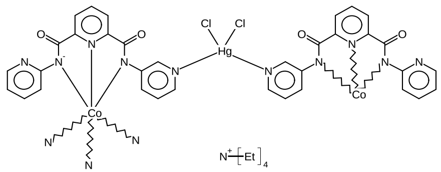

$N^+[Et]_4$

HIHQEW

Reference: Denan Wang, S.V.Lindeman, A.T.Fiedler (2013) *Eur.J.Inorg.Chem.* ,4473

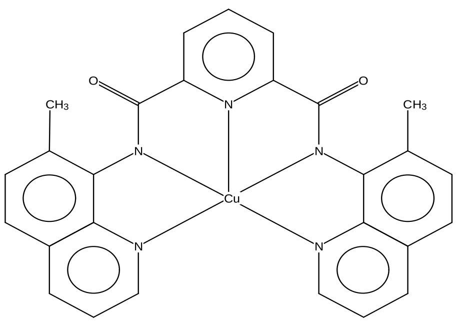

H_2O

HIHTEZ

Reference: Denan Wang, S.V.Lindeman, A.T.Fiedler (2013) *Eur.J.Inorg.Chem.*, 4473

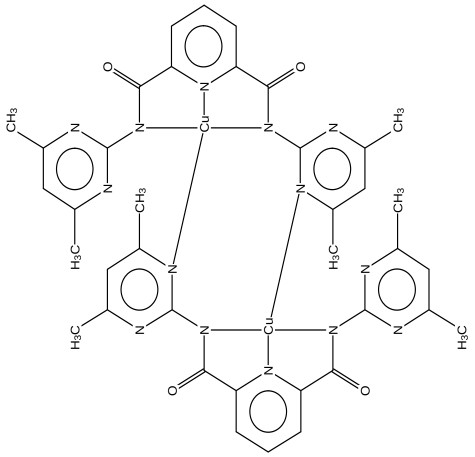

HIHTID

Reference: Denan Wang, S.V.Lindeman, A.T.Fiedler (2013) *Eur.J.Inorg.Chem.*, 4473

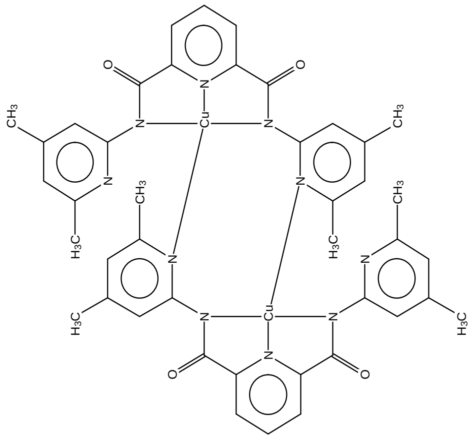

HIHTOJ

Reference: Denan Wang, S.V.Lindeman, A.T.Fiedler (2013) *Eur.J.Inorg.Chem.*, 4473

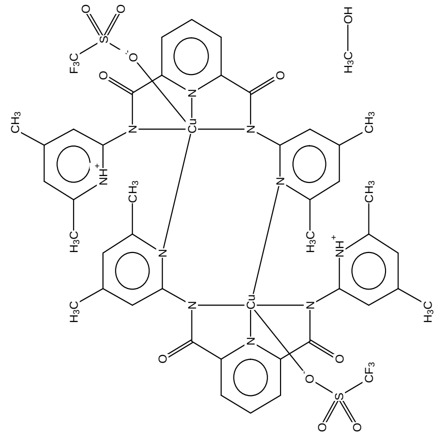

HIHTUP

Reference: Denan Wang, S.V.Lindeman, A.T.Fiedler (2013) *Eur.J.Inorg.Chem.*, 4473

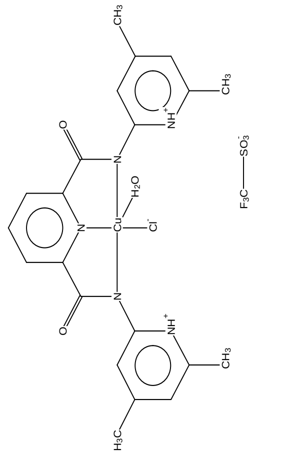

HIHVAX

Reference: Denan Wang, S.V.Lindeman, A.T.Fiedler (2013) *Eur.J.Inorg.Chem.*, 4473

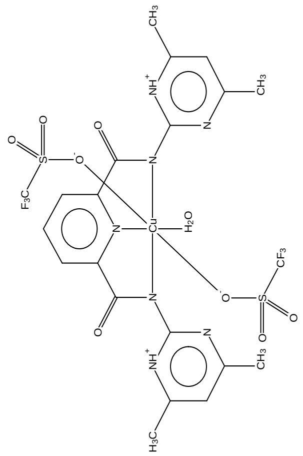

HIHVEB

Reference: Denan Wang, S.V.Lindeman, A.T.Fiedler (2013) *Eur.J.Inorg.Chem.*, 4473

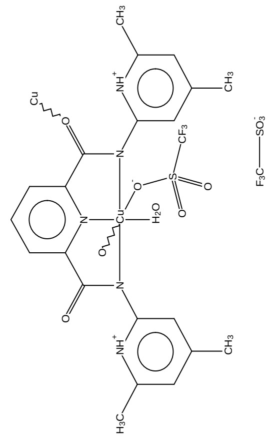

HIHVIF

Reference: Denan Wang, S.V.Lindeman, A.T.Fiedler (2013) *Eur.J.Inorg.Chem.*, 4473

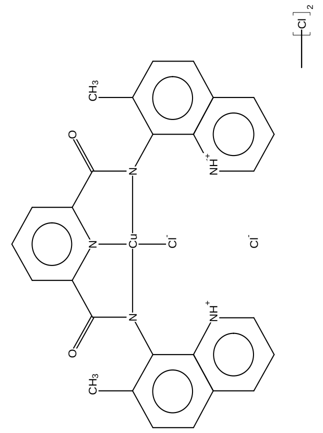

HIHVOL

Reference: Denan Wang, S.V.Lindeman, A.T.Fiedler (2013) *Eur.J.Inorg.Chem.*, 4473

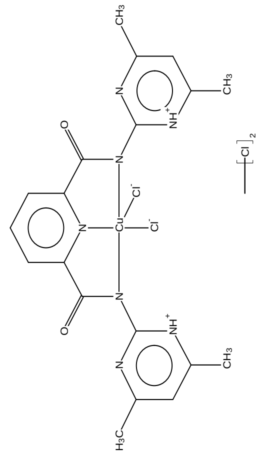

HIHVUR

Reference: Denan Wang, S.V.Lindeman, A.T.Fiedler (2013) *Eur.J.Inorg.Chem.* ,4473

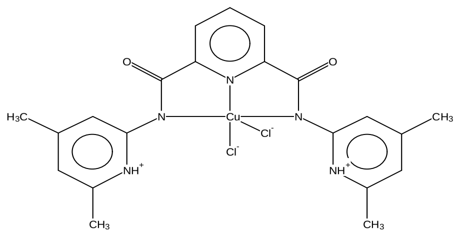

HIKTED

Reference: G.Kumar, F.Hussain, R.Gupta (2018) *Dalton Trans.* ,**47,** 16985

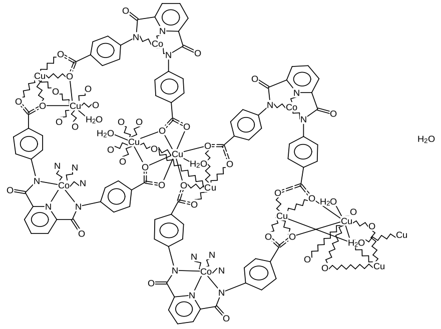

HIYWAQ

Reference: W.D.Bailey, N.L.Gagnon, C.E.Elwell, A.C.Cramblitt, C.J.Bouchey, W.B.Tolman (2019) *Inorg.Chem.* ,**58,**4706

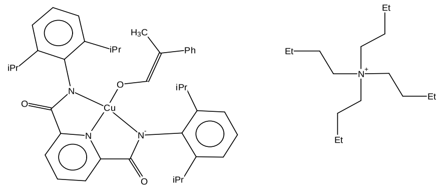

HIZRUF

Reference: N.Zigon, P.Larpent, A.Jouaiti, N.Kyritsakas, M.W.Hosseini (2014) *Chem.Commun.* ,**50,**5040

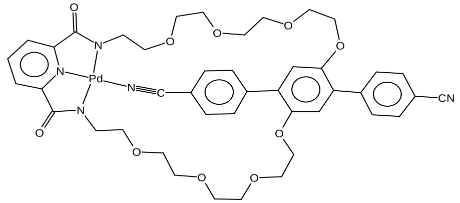

HOCXIJ

Reference: W.D.Bailey, Debanjan Dhar, A.C.Cramblitt, W.B.Tolman (2019) *J.Am.Chem.Soc.* ,**141,**5470

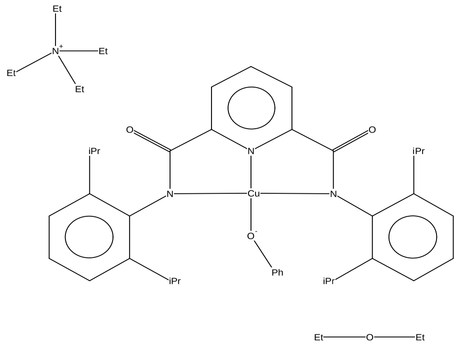

HOCXOP

Reference: W.D.Bailey, Debanjan Dhar, A.C.Cramblitt, W.B.Tolman (2019) *J.Am.Chem.Soc.* ,**141,**5470

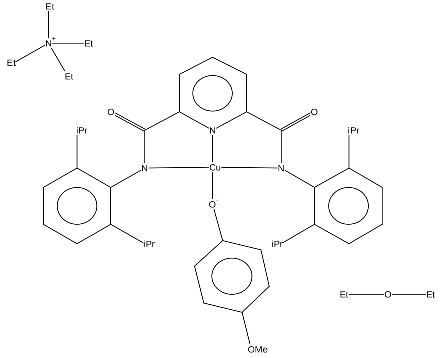

HOCXUV

Reference: W.D.Bailey, Debanjan Dhar, A.C.Cramblitt, W.B.Tolman (2019) *J.Am.Chem.Soc.* ,**141,**5470

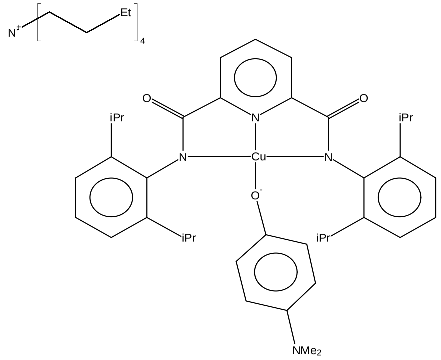

HOHHAP

Reference: D.Bansal, G.Kumar, G.Hundal, R.Gupta (2014) *Dalton Trans.* ,**43,**14865

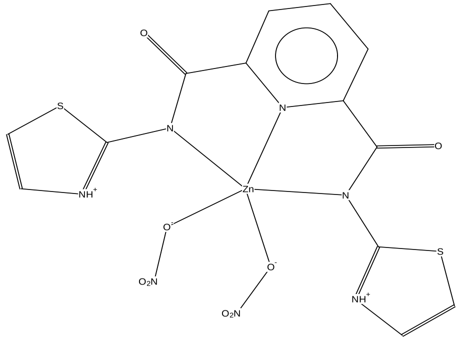

HOHHET

Reference: D.Bansal, G.Kumar, G.Hundal, R.Gupta (2014)
Dalton Trans. ,**43,**14865

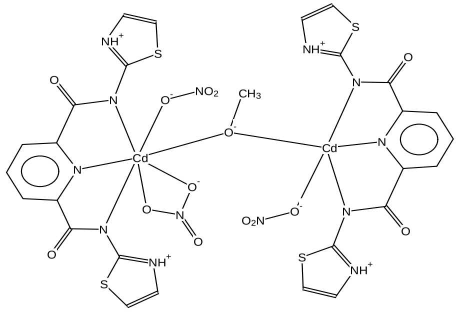

HOHHIX

Reference: D.Bansal, G.Kumar, G.Hundal, R.Gupta (2014)
Dalton Trans. ,**43,**14865

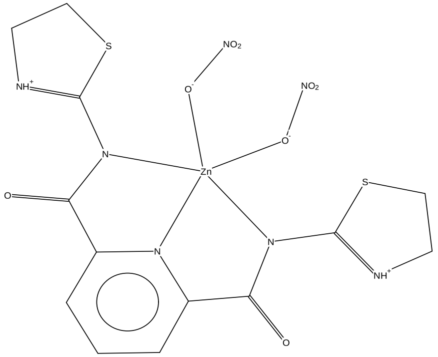

HOHHOD

Reference: D.Bansal, G.Kumar, G.Hundal, R.Gupta (2014)
Dalton Trans. ,**43,**14865

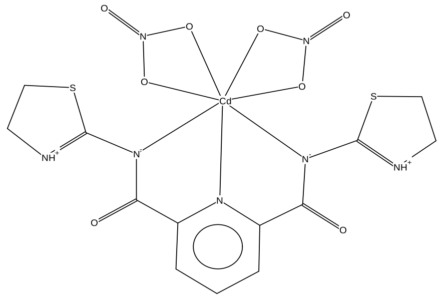

HOHHUJ

Reference: D.Bansal, G.Kumar, G.Hundal, R.Gupta (2014)
Dalton Trans. ,**43,**14865

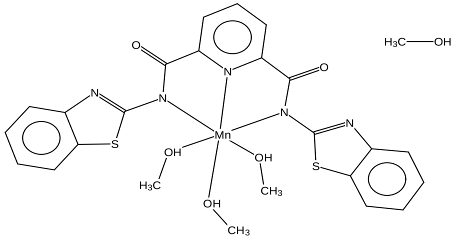

HOKDIW

Reference: S.Srivastava, M.S.Dagur, R.Gupta (2014)
Eur.J.Inorg.Chem. ,4966

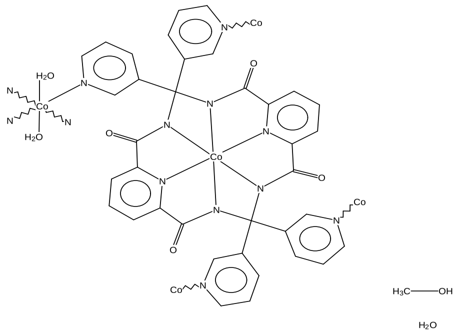

HUCCAM

Reference: A.L.Rheingold, A.S.Borovik (2019)
CSD Communication(Private Communication) ,

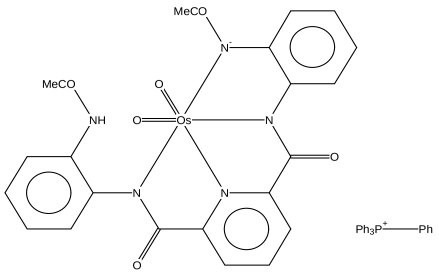

HUCCEQ

Reference: A.L.Rheingold, A.S.Borovik (2019)
CSD Communication(Private Communication) ,

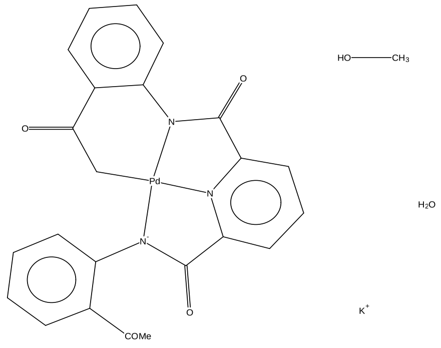

HUCCOA

Reference: A.L.Rheingold, A.S.Borovik (2019)
CSD Communication(Private Communication) ,

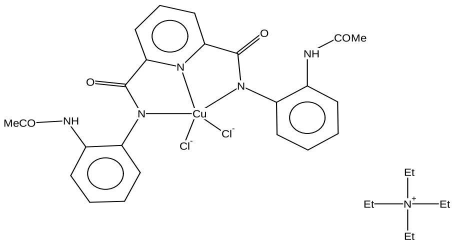

HUCFET

Reference: A.L.Rheingold, A.S.Borovik (2019) *CSD Communication(Private Communication)* ,

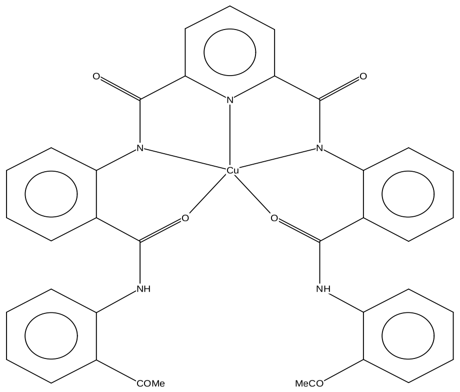

HUTMUH

Reference: Yinghua Li, Weibin Fan, Zilong Zhang, Xingkun Xie, Shiqun Xiang, Deguang Huang (2020) *Dalton Trans.* ,**49,**12189

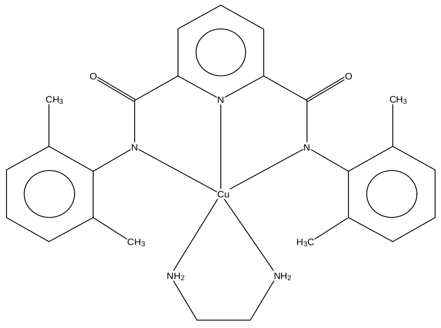

HUTNAO

Reference: Yinghua Li, Weibin Fan, Zilong Zhang, Xingkun Xie, Shiqun Xiang, Deguang Huang (2020) *Dalton Trans.* ,**49,**12189

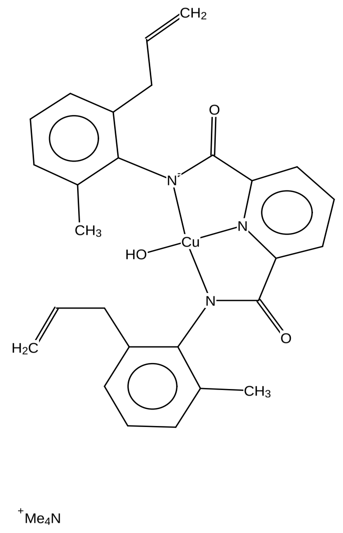

HUTNUI

Reference: Yinghua Li, Weibin Fan, Zilong Zhang, Xingkun Xie, Shiqun Xiang, Deguang Huang (2020) *Dalton Trans.* ,**49,**12189

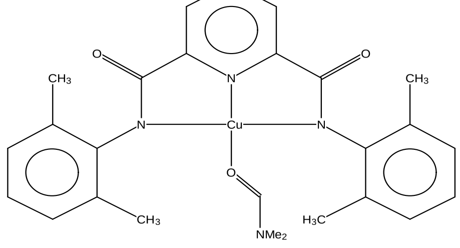

IBEXEU

Reference: P.J.Donoghue, J.Tehranchi, C.J.Cramer, R.Sarangi, E.I.Solomon, W.B.Tolman (2011) *J.Am.Chem.Soc.* ,**133,**17602

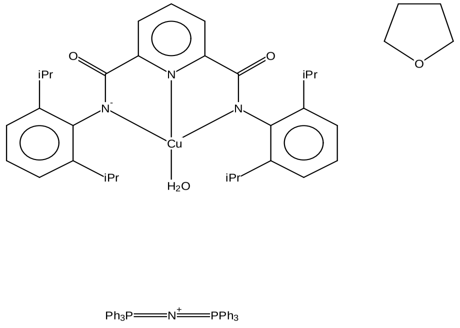

IBEXIY

Reference: P.J.Donoghue, J.Tehranchi, C.J.Cramer, R.Sarangi, E.I.Solomon, W.B.Tolman (2011) *J.Am.Chem.Soc.* ,**133,**17602

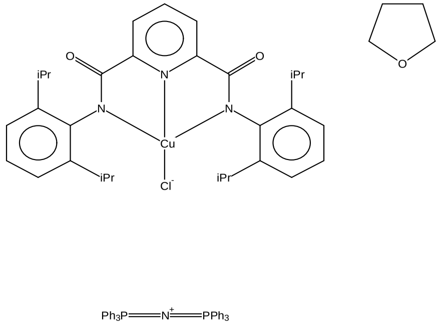

IBEXOE

Reference: P.J.Donoghue, J.Tehranchi, C.J.Cramer, R.Sarangi, E.I.Solomon, W.B.Tolman (2011) *J.Am.Chem.Soc.* ,**133,**17602

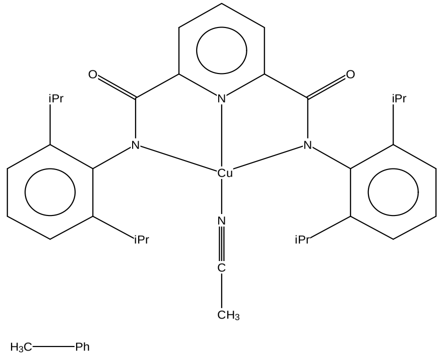

IBEXUK

Reference: P.J.Donoghue, J.Tehranchi, C.J.Cramer, R.Sarangi, E.I.Solomon, W.B.Tolman (2011) *J.Am.Chem.Soc.* ,**133,**17602

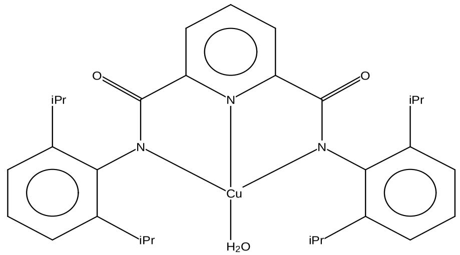

ICEKOS

Reference: Deguang Huang, O.V.Makhlynets, Lay Ling Tan, S.C.Lee, E.V.Rybak-Akimova, R.H.Holm (2011) *Inorg.Chem.* ,**50,**10070

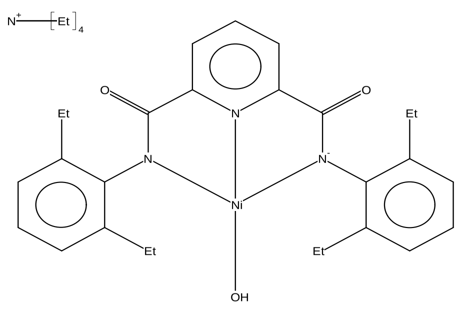

ICEKUY

Reference: Deguang Huang, O.V.Makhlynets, Lay Ling Tan, S.C.Lee, E.V.Rybak-Akimova, R.H.Holm (2011) *Inorg.Chem.* ,**50,**10070

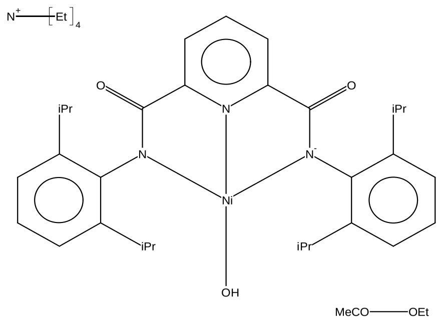

ICEKUY01

Reference: F.Czerny, P.Dohlert, M.Weidauer, E.Irran, S.Enthaler (2015) *Inorg.Chim.Acta* ,**425,**118

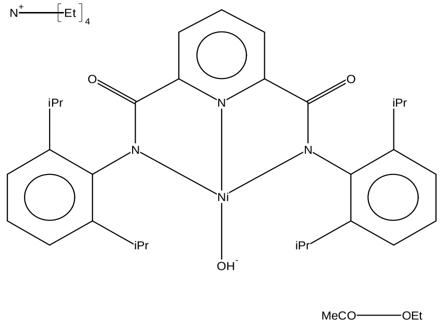

ICELAF

Reference: Deguang Huang, O.V.Makhlynets, Lay Ling Tan, S.C.Lee, E.V.Rybak-Akimova, R.H.Holm (2011) *Inorg.Chem.* ,**50,**10070

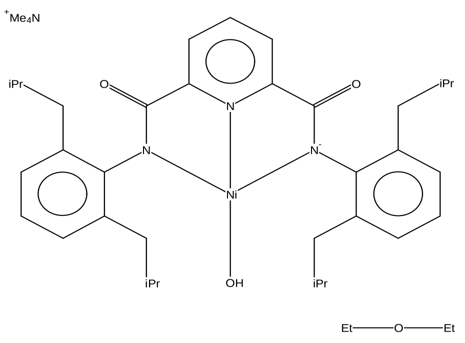

ICELEJ

Reference: Deguang Huang, O.V.Makhlynets, Lay Ling Tan, S.C.Lee, E.V.Rybak-Akimova, R.H.Holm (2011) *Inorg.Chem.* ,**50,**10070

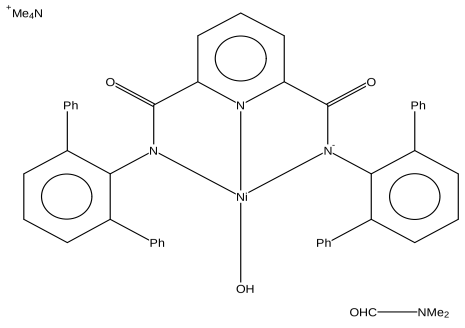

ICELIN

Reference: Deguang Huang, O.V.Makhlynets, Lay Ling Tan, S.C.Lee, E.V.Rybak-Akimova, R.H.Holm (2011) *Inorg.Chem.* ,**50,**10070

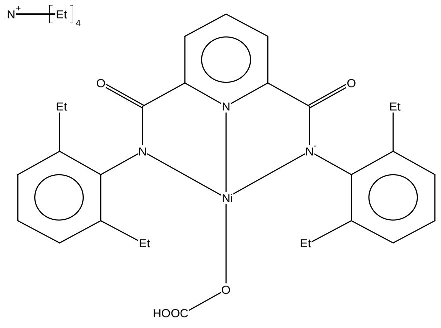

ICELOT

Reference: Deguang Huang, O.V.Makhlynets, Lay Ling Tan, S.C.Lee, E.V.Rybak-Akimova, R.H.Holm (2011) *Inorg.Chem.* ,**50,**10070

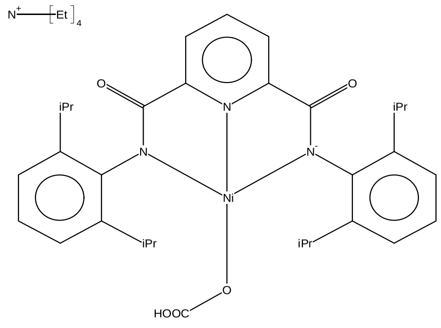

ICELUZ

Reference: Deguang Huang, O.V.Makhlynets, Lay Ling Tan, S.C.Lee, E.V.Rybak-Akimova, R.H.Holm (2011) *Inorg.Chem.* ,**50,**10070

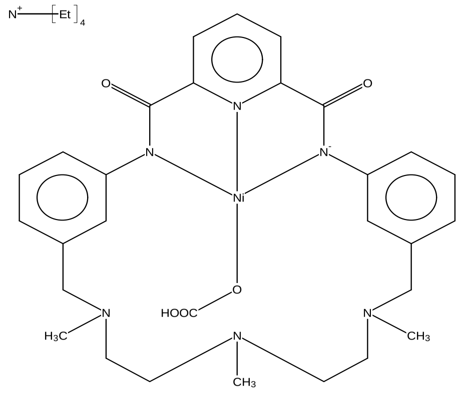

ICEMAG

Reference: Deguang Huang, O.V.Makhlynets, Lay Ling Tan, S.C.Lee, E.V.Rybak-Akimova, R.H.Holm (2011) *Inorg.Chem.* ,**50,**10070

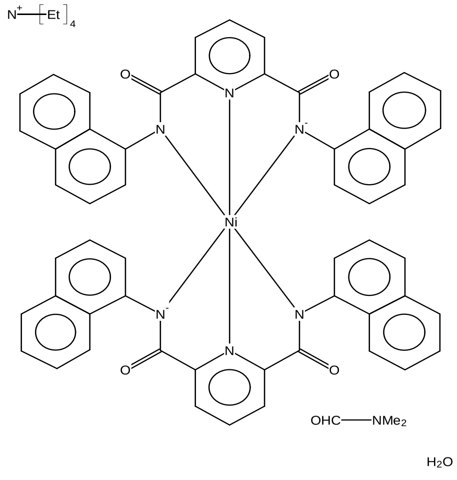

IHAHAB

Reference: Xiao-Qing Shen, Zhong-Jun Li, Zi-Feng Li, Rui Yang, Hong-Yun Zhang (2009) *Inorg.Chim.Acta* ,**362,**4031

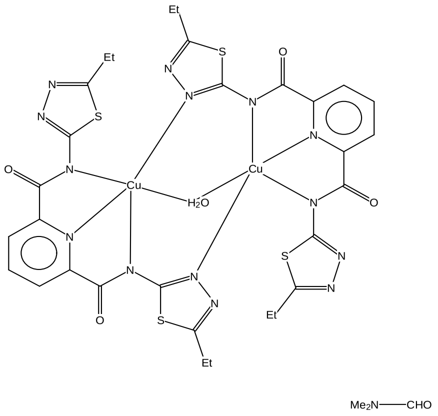

IHAHEF

Reference: Xiao-Qing Shen, Zhong-Jun Li, Zi-Feng Li, Rui Yang, Hong-Yun Zhang (2009) *Inorg.Chim.Acta* ,**362,**4031

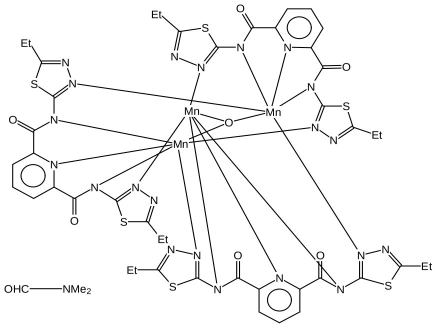

ILUZEV

Reference: G.Kumar, A.P.Singh, R.Gupta (2010) *Eur.J.Inorg.Chem.* , 5103

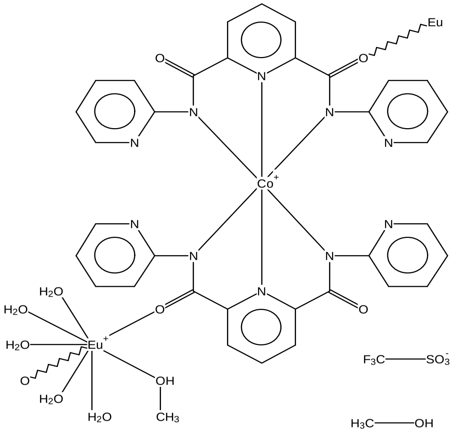

ILUZIZ

Reference: G.Kumar, A.P.Singh, R.Gupta (2010) *Eur.J.Inorg.Chem.* , 5103

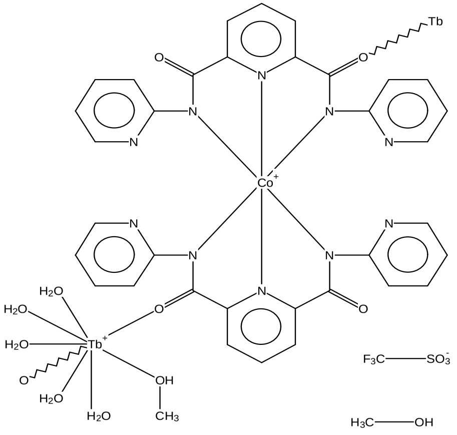

IZAHAS

Reference: Sheng-Gui Liu, Yi-Zhi Li, Jing-Lin Zuo, Xiao-Zeng You (2004) *Acta Crystallogr.,Sect.E:Struct.Rep.Online* ,**60,**m1153

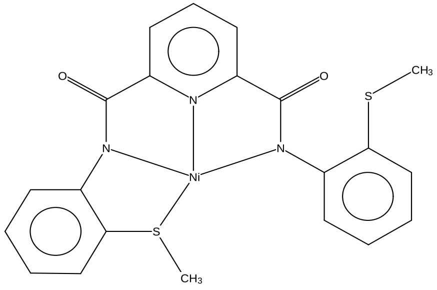

IZUXEG

Reference: X.Shen, T.Moriuchi, T.Hirao (2004) *Tetrahedron Lett.* ,**45,** 4733

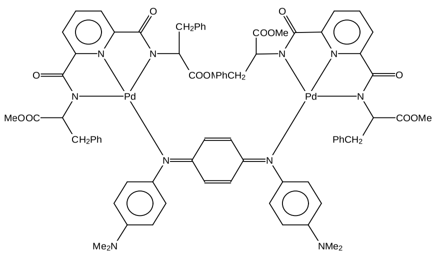

IZUXEG01

Reference: T.Moriuchi, Xiuliang Shen, T.Hirao (2006) *Tetrahedron* ,**62,** 12237

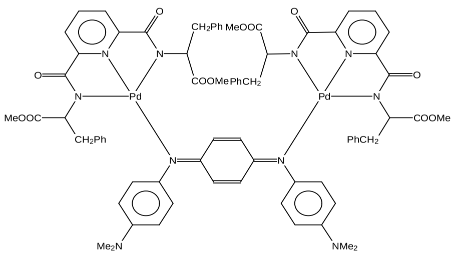

JEQDER

Reference: G.Kumar, F.Hussain, R.Gupta (2017) *Dalton Trans.* ,**46,** 15023

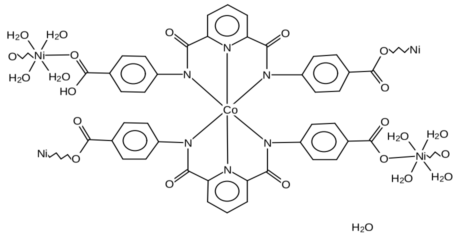

JIPGEV

Reference: B.Walker, D.Leigh, S.Parsons (2007) *CSD Communication(Private Communication)* ,

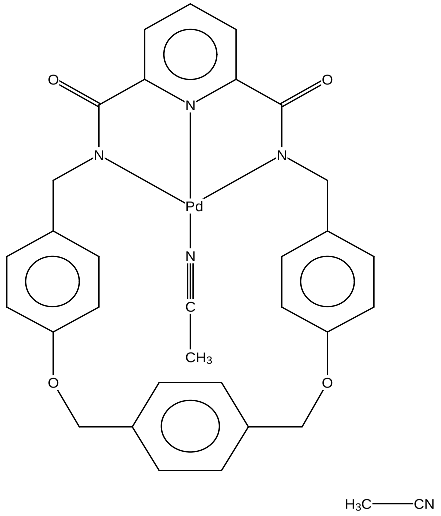

JITZAO

Reference: J.D.Crowley, D.A.Leigh, P.J.Lusby, R.T.McBurney, L.-E.Perret-Aebi, C.Petzold, A.M.Z.Slawin, M.D.Symes (2007) *J.Am.Chem.Soc.* ,**129,**15085

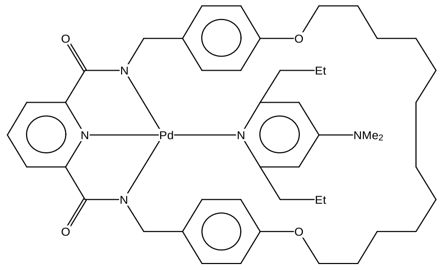

JITZES

Reference: J.D.Crowley, D.A.Leigh, P.J.Lusby, R.T.McBurney, L.-E.Perret-Aebi, C.Petzold, A.M.Z.Slawin, M.D.Symes (2007) *J.Am.Chem.Soc.* ,**129,**15085

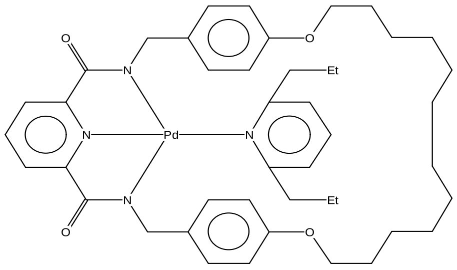

JOSPIQ

Reference: T.Kawamoto, B.S.Hammes, R.Ostrander, A.L.Rheingold, A.S.Borovik (1998) *Inorg.Chem.* ,**37,**3424

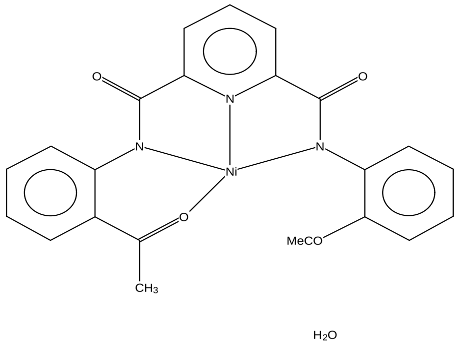

JOSQAJ

Reference: T.Kawamoto, B.S.Hammes, R.Ostrander, A.L.Rheingold, A.S.Borovik (1998) *Inorg.Chem.* ,**37,**3424

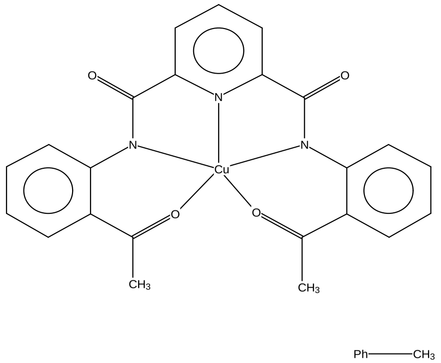

JUTWUS

Reference: P.Ludomenov, D.Leigh, Iain Oswald, S.Parsons (2015) *CSD Communication(Private Communication)* ,

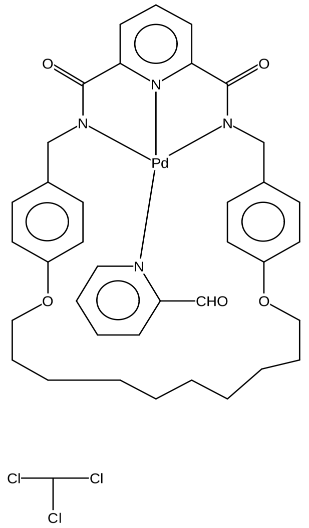

JUXKOE

Reference: Yue-xin Guo, Hong-cui Ma, Li-tong Duan, Wei-guo Zhang, Xiang Cheng, Hong-wei Hou, Yao-ting Fan (2013) *Hecheng Huaxue(Chin.)(Chin.J.Synth.Chem.)* ,**21,**447

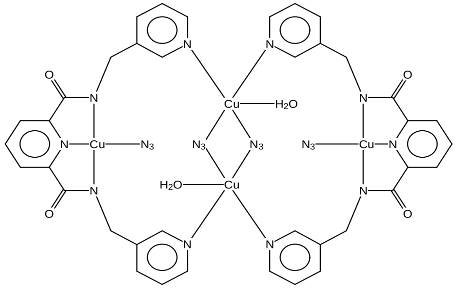

JUYXAE

Reference: D.Bansal, R.Gupta (2016) *Dalton Trans.* ,**45,**502

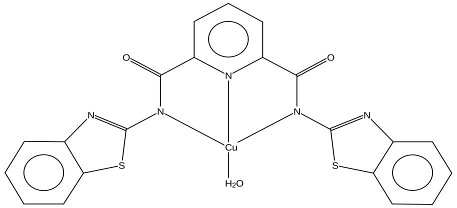

KAJLIT

Reference: Debanjan Dhar, G.M.Yee, A.D.Spaeth, D.W.Boyce, Hongtu Zhang, B.Dereli, C.J.Cramer, W.B.Tolman (2016) *J.Am.Chem.Soc.* ,**138,**356

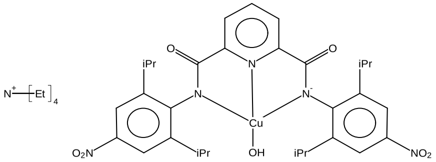

KAJLOZ

Reference: Debanjan Dhar, G.M.Yee, A.D.Spaeth, D.W.Boyce, Hongtu Zhang, B.Dereli, C.J.Cramer, W.B.Tolman (2016) *J.Am.Chem.Soc.* ,**138,**356

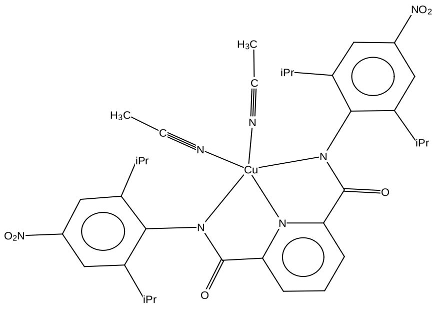

KAJLUF

Reference: Debanjan Dhar, G.M.Yee, A.D.Spaeth, D.W.Boyce, Hongtu Zhang, B.Dereli, C.J.Cramer, W.B.Tolman (2016) *J.Am.Chem.Soc.* ,**138,**356

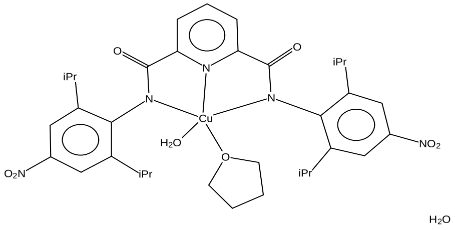

KAJMAM

Reference: Debanjan Dhar, G.M.Yee, A.D.Spaeth, D.W.Boyce, Hongtu Zhang, B.Dereli, C.J.Cramer, W.B.Tolman (2016) *J.Am.Chem.Soc.* ,**138,**356

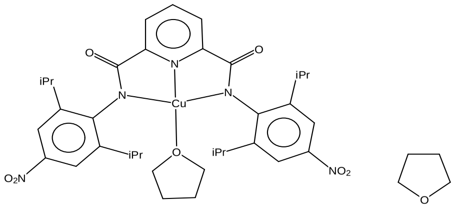

KEDTIZ

Reference: P.Kumar, V.Kumar, R.Gupta (2017) *Dalton Trans.* ,**46,** 10205

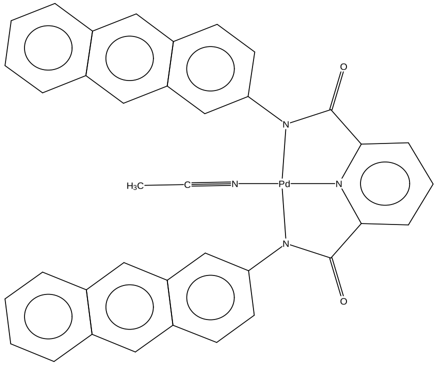

KEQKEY

Reference: N.K.Kaushik, A.Mishra, A.Ali, J.S.Adhikari, A.K.Verma, R.Gupta (2012) *J.Biol.Inorg.Chem.(JBIC)* ,**17,**1217

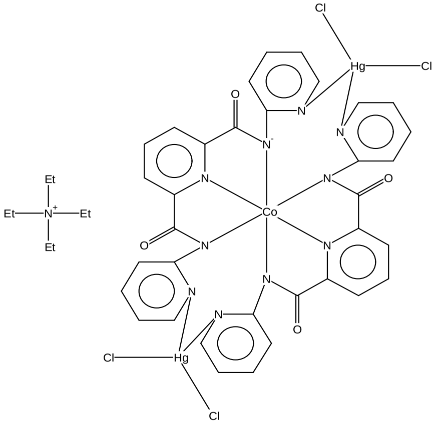

KIBDAC

Reference: G.Kumar, R.Gupta (2012) *Inorg.Chem.* ,**51,**5497

H_2O

n-$CoCd_2$--
--[μ_4-$C_{21}H_{11}N_3O_6$-κ^3N,N',N'',κ^4O,O',O'',O''']$_2$[H_2O]$_5$

n-$CoCd_3$[μ_6-$C_{21}H_{11}N_3O_6$]--
--[μ_4-$C_{21}H_{11}N_3O_6$-κ^3N,N',N'',κ^4O,O',O'',O'''][H_2O]$_{10}$

KIBDEG

Reference: G.Kumar, R.Gupta (2012) *Inorg.Chem.* ,**51**,5497

H_2O

n-Co_2Cd_4--
--[μ_3-$C_{21}H_{11}N_3O_6$-κ^3N,N',N'',κ^4O,O',O'',O''']$_4$[H_2O]$_{13}$

KIWFOO

Reference: T.K.Achar, K.Ramakrishna, T.Pal, S.Porey, P.Dolui, J.P.Biswas, D.Maiti (2018) *Chem.-Eur.J.* ,**24,**17906

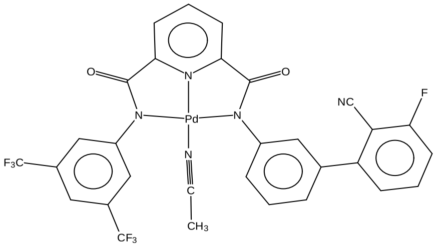

KOBNOF

Reference: S.M.Goldup, D.A.Leigh, P.J.Lusby, R.T.McBurney, A.M.Z.Slawin (2008) *Angew.Chem.,Int.Ed.* ,**47**,3381

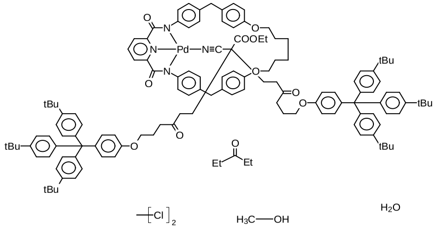

KOBNUL

Reference: S.M.Goldup, D.A.Leigh, P.J.Lusby, R.T.McBurney, A.M.Z.Slawin (2008) *Angew.Chem.,Int.Ed.* ,**47**,3381

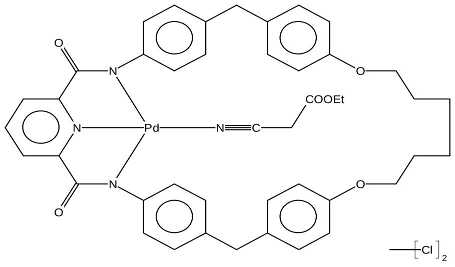

KOBPAT

Reference: S.M.Goldup, D.A.Leigh, P.J.Lusby, R.T.McBurney, A.M.Z.Slawin (2008) *Angew.Chem.,Int.Ed.* ,**47**,3381

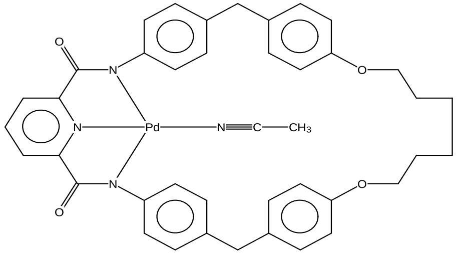

KUGSUC

Reference: M.Ogawa, M.Nagashima, H.Sogawa, S.Kuwata, T.Takata (2015) *Org.Lett.* ,**17,**1664

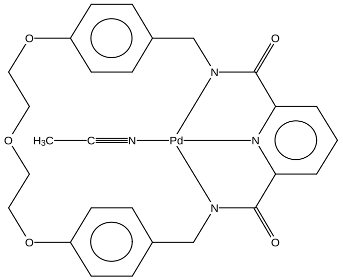

H_2O

KUGTAJ

Reference: M.Ogawa, M.Nagashima, H.Sogawa, S.Kuwata, T.Takata (2015) *Org.Lett.* ,**17,**1664

Pd_4[μ_2-$C_{27}H_{27}N_3O_6$-κ^3N,N',N'',κ^1O]$_4$

[Cl]$_3$

KUTTAX

Reference: P.Mondal, M.Lovisari, B.Twamley, A.R.McDonald (2020) *Angew.Chem.,Int.Ed.* ,**59,**13044

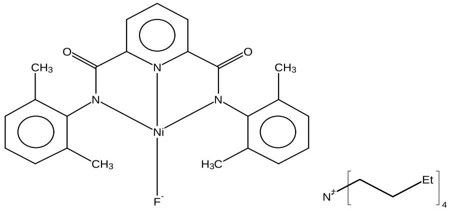

KUVSAX

Reference: S.Srivastava, M.S.Dagur, A.Ali, R.Gupta (2015) *Dalton Trans.* ,**44**,17453

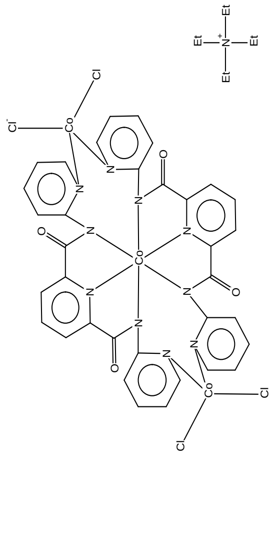

KUVSEB

Reference: S.Srivastava, M.S.Dagur, A.Ali, R.Gupta (2015) *Dalton Trans.* ,**44**,17453

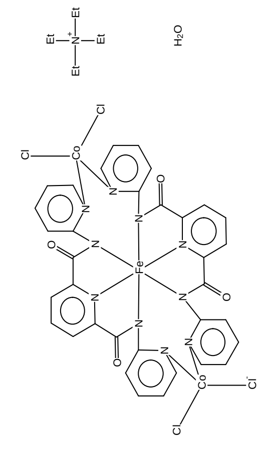

LASVOR

Reference: D.A.Leigh, P.J.Lusby, A.M.Z.Slawin, D.B.Walker (2005) *Angew.Chem.,Int.Ed.* ,**44**,4557

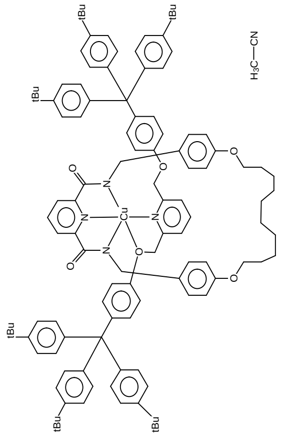

LASVUX

Reference: D.A.Leigh, P.J.Lusby, A.M.Z.Slawin, D.B.Walker (2005) *Angew.Chem.,Int.Ed.* ,**44**,4557

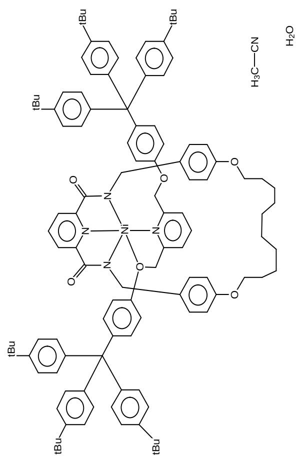

LIYPUG

Reference: A.Adhikary, J.A.Sheikh, A.D.Konar, S.Konar (2014) *RSC Advances* ,**4**,12408

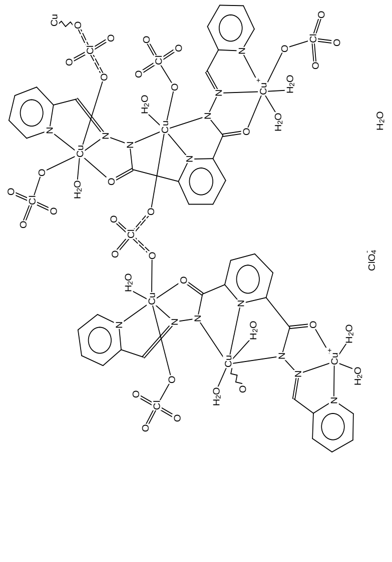

LUZTIL

Reference: Yanchuan Zhao, Lily Chen, T.M.Swager (2016) *Angew.Chem.,Int.Ed.* ,**55**,917

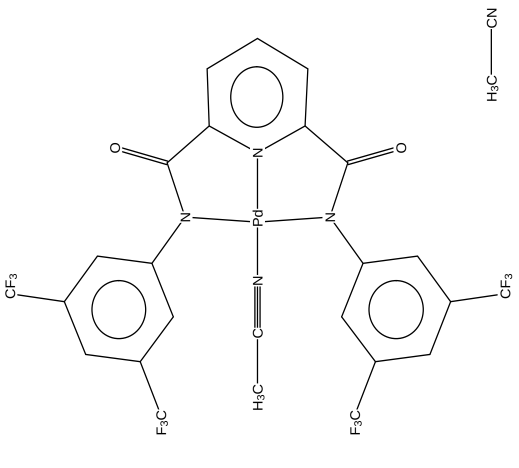

LUZTOR

Reference: Yanchuan Zhao, Lily Chen, T.M.Swager (2016) *Angew.Chem.,Int.Ed.* ,**55**,917

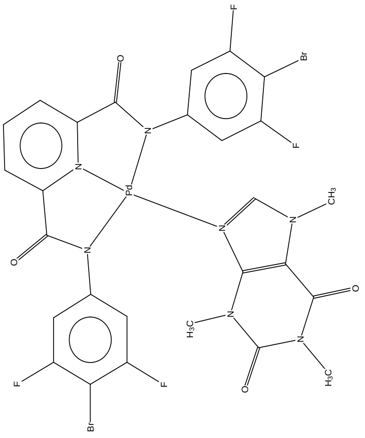

MAPMIZ

Reference: D.S.Marlin, M.M.Olmstead, P.K.Mascharak (1999) *Inorg.Chem.* ,**38**,3258

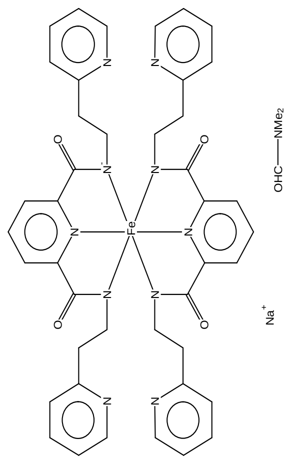

MAPMOF

Reference: D.S.Marlin, M.M.Olmstead, P.K.Mascharak (1999) *Inorg.Chem.* ,**38,**3258

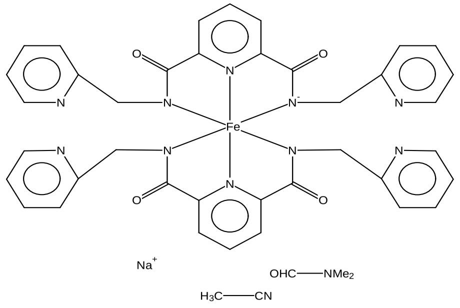

MARVAC

Reference: T.Moriuchi, S.Bandoh, Y.Miyaji, T.Hirao (2000) *J.Organomet.Chem.* ,**599,**135

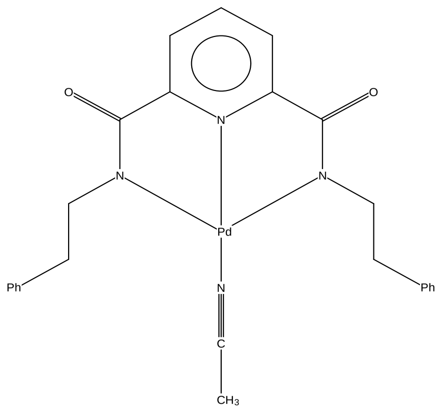

MARVEG

Reference: T.Moriuchi, S.Bandoh, Y.Miyaji, T.Hirao (2000) *J.Organomet.Chem.* ,**599,**135

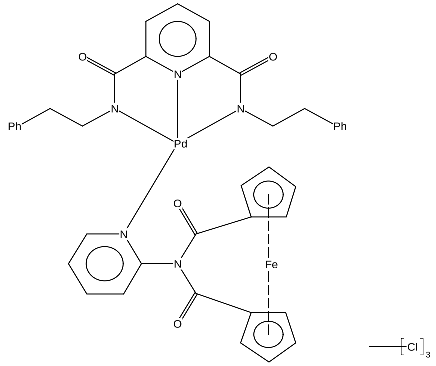

MEQQEF

Reference: A.J.Preston, J.C.Gallucci, J.R.Parquette (2006) *Org.Lett.* , **8,**5259

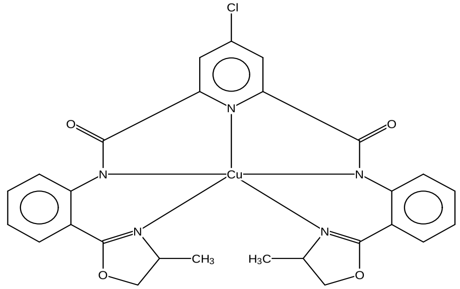

MEQQIJ

Reference: A.J.Preston, J.C.Gallucci, J.R.Parquette (2006) *Org.Lett.* , **8,**5259

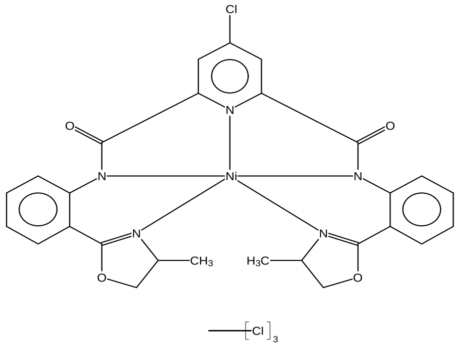

MEQQOP

Reference: A.J.Preston, J.C.Gallucci, J.R.Parquette (2006) *Org.Lett.* , **8,**5259

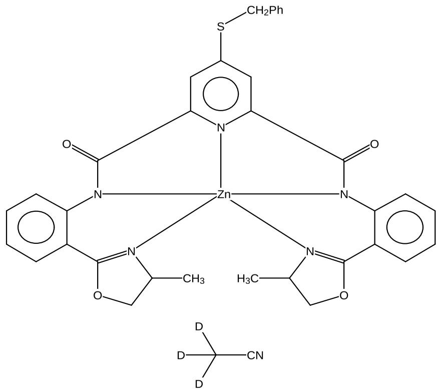

MEQQUV

Reference: A.J.Preston, J.C.Gallucci, J.R.Parquette (2006) *Org.Lett.* , **8,**5259

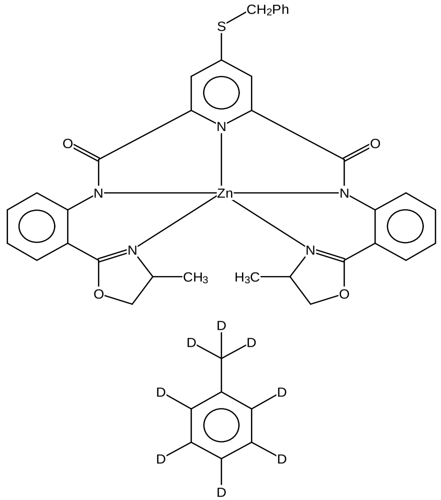

MIHJAQ

Reference: J.Tehranchi, P.J.Donoghue, C.J.Cramer, W.B.Tolman (2013) *Eur.J.Inorg.Chem.* ,4077

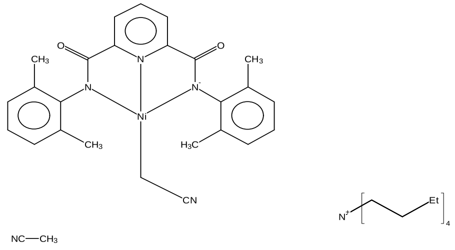

MIHJEU

Reference: J.Tehranchi, P.J.Donoghue, C.J.Cramer, W.B.Tolman (2013) *Eur.J.Inorg.Chem.* ,4077

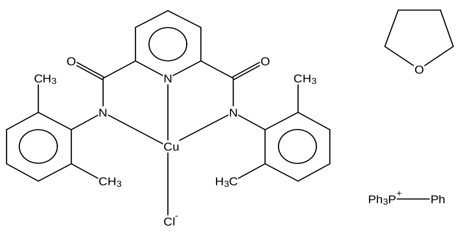

MIHJIY

Reference: J.Tehranchi, P.J.Donoghue, C.J.Cramer, W.B.Tolman (2013) *Eur.J.Inorg.Chem.* ,4077

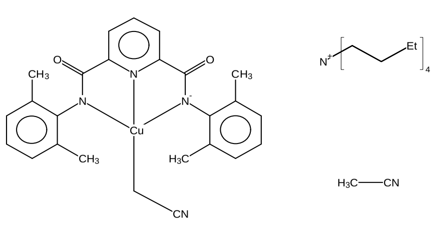

MIJFUI

Reference: M.R.Halvagar, W.B.Tolman (2013) *Inorg.Chem.* ,**52,**8306

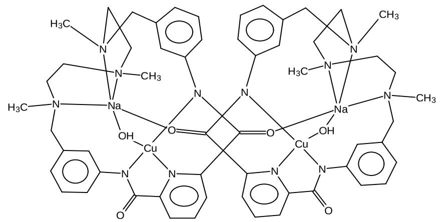

MIJGAP

Reference: M.R.Halvagar, W.B.Tolman (2013) *Inorg.Chem.* ,**52,**8306

Cu_2Na_2--

--[μ_3-$C_{25}H_{23}N_6O_2Me_3$-κ^6N,N',N'',N''',N'''',N''''',κ^1O]$_2$[μ_2-$C_4H_7O_2$-κ^3O,O']$_2$

MIJNUO

Reference: T.Moriuchi, I.Nakayama, K.Nishimura, M.Nishiyama, E.Mochizuki, Y.Kai, T.Hirao (2001) *Chem.Lett.* ,1328

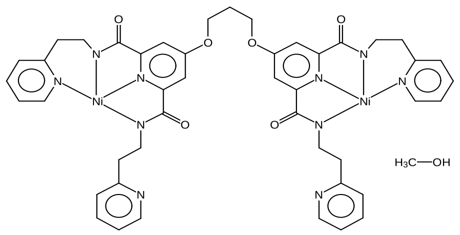

MIMDAQ

Reference: C.E.Elwell, B.D.Neisen, W.B.Tolman (2019) *Inorg.Chim.Acta* ,**485,**131

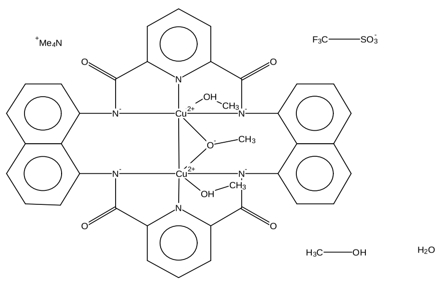

MIMDEU

Reference: C.E.Elwell, B.D.Neisen, W.B.Tolman (2019) *Inorg.Chim.Acta* ,**485,**131

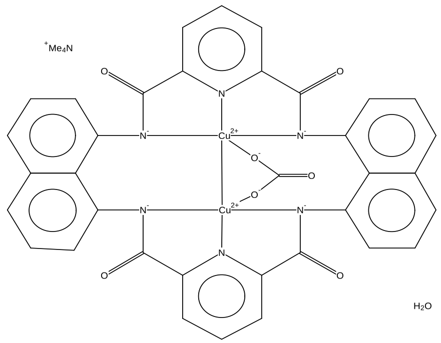

MIMFIA

Reference: C.E.Elwell, B.D.Neisen, W.B.Tolman (2019) *Inorg.Chim.Acta* ,**485,**131

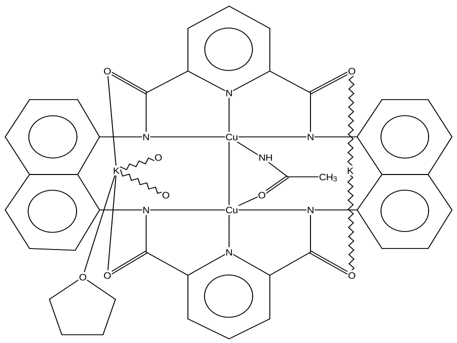

MIMWUB

Reference: B.Walker, D.Leigh, S.Parsons, I.Oswald, R.D.L.Johnstone (2007) *CSD Communication(Private Communication)* ,

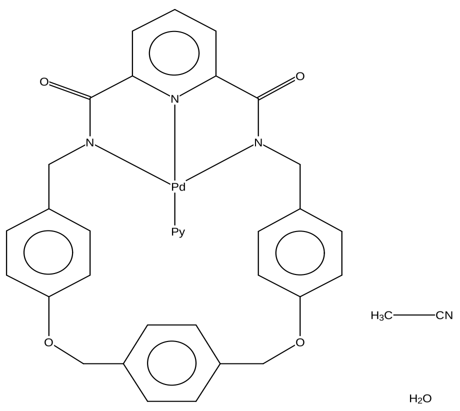

MIQZUI

Reference: M.T.Raisanen, M.Klinga, M.Leskela, T.Repo (2007) *Acta Crystallogr.,Sect.E:Struct.Rep.Online* ,**63**,m3021

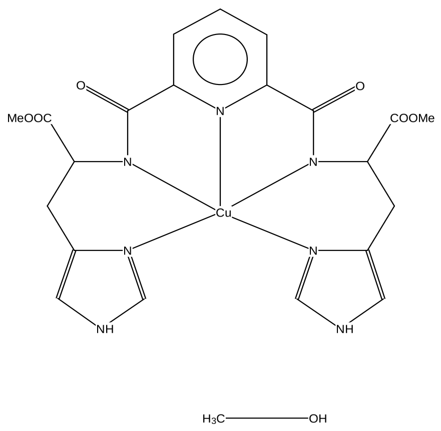

MOWCUZ

Reference: C.E.Elwell, M.Mandal, C.J.Bouchey, L.Que, Jr., C.J.Cramer, W.B.Tolman (2019) *Inorg.Chem.* ,**58**,15872

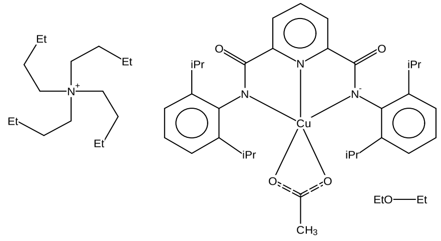

MOXVOM

Reference: D.Bansal, G.Hundal, R.Gupta (2015) *Eur.J.Inorg.Chem.* , 1022

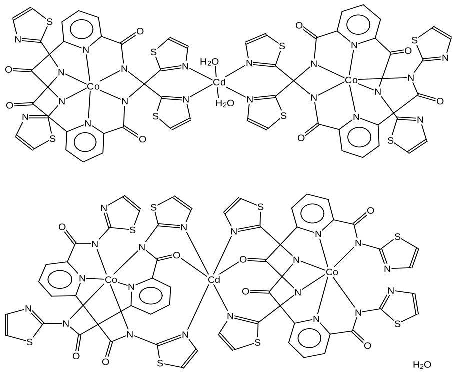

MOXVUS

Reference: D.Bansal, G.Hundal, R.Gupta (2015) *Eur.J.Inorg.Chem.* , 1022

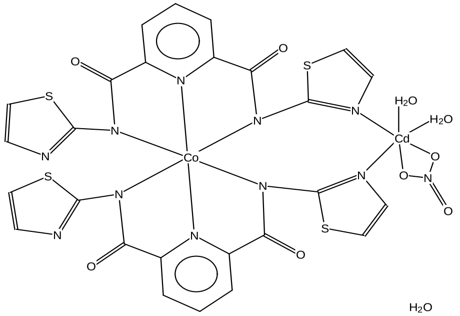

MOXWAZ

Reference: D.Bansal, G.Hundal, R.Gupta (2015) *Eur.J.Inorg.Chem.* , 1022

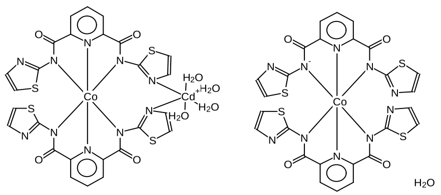

MOXWED

Reference: D.Bansal, G.Hundal, R.Gupta (2015) *Eur.J.Inorg.Chem.* , 1022

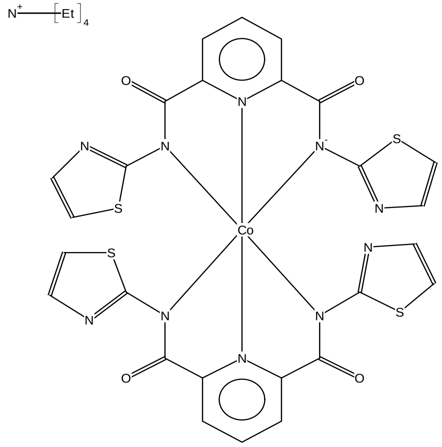

MOXWIH

Reference: D.Bansal, G.Hundal, R.Gupta (2015) *Eur.J.Inorg.Chem.* , 1022

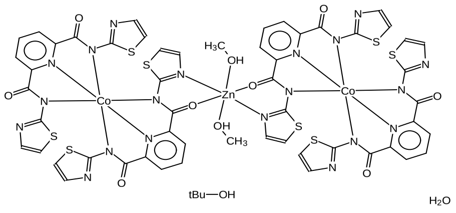

MOXWON

Reference: D.Bansal, G.Hundal, R.Gupta (2015) *Eur.J.Inorg.Chem.* , 1022

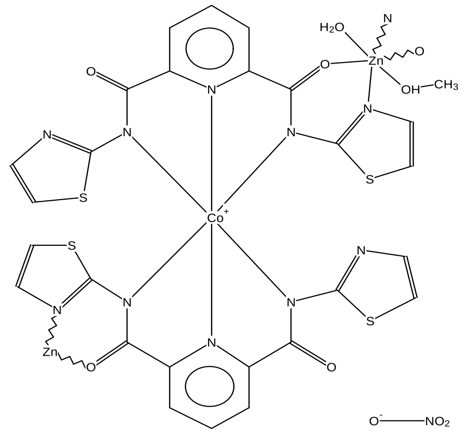

MUPJEM

Reference: J.Swiatek-Kozlowska, E.Gumienna-Kontecka, A.Dobosz, I.A.Golenya, I.O.Fritsky (2002) *J.Chem.Soc.,Dalton Trans.* ,4639

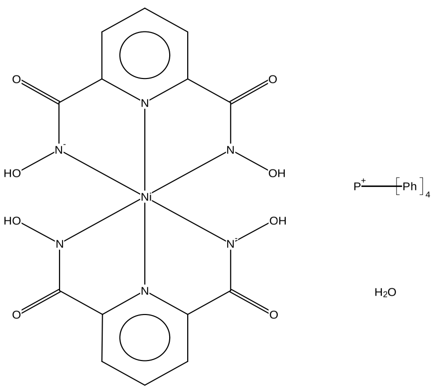

NAHDEI

Reference: Wangchuk Rabten, T.Akermark, M.D.Karkas, Hong Chen, Junliang Sun, P.G.Andersson, B.Akermark (2016) *Dalton Trans.* ,**45,** 3272

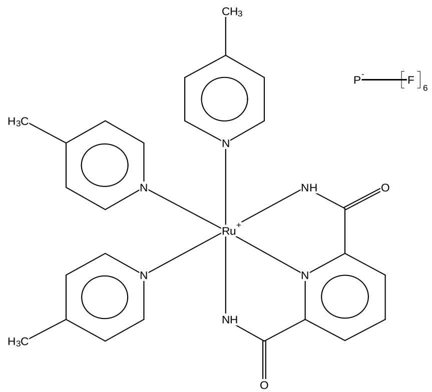

NARLUQ

Reference: D.Bansal, R.Gupta (2017) *Dalton Trans.* ,**46,**4617

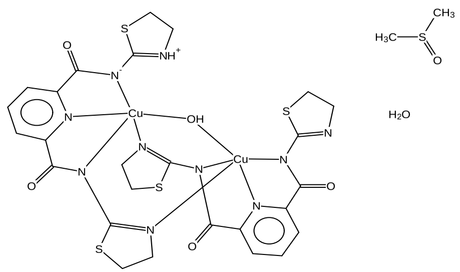

NARMAX

Reference: D.Bansal, R.Gupta (2017) *Dalton Trans.* ,**46,**4617

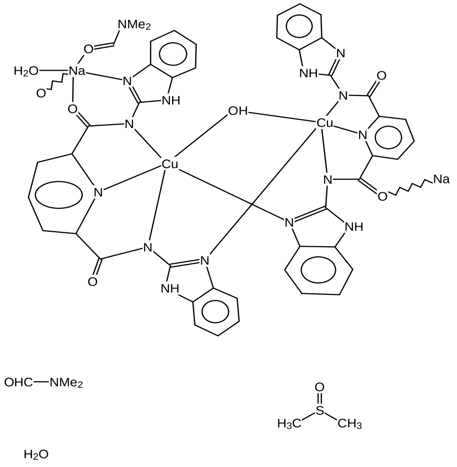

NARMEB

Reference: D.Bansal, R.Gupta (2017) *Dalton Trans.* ,**46,**4617

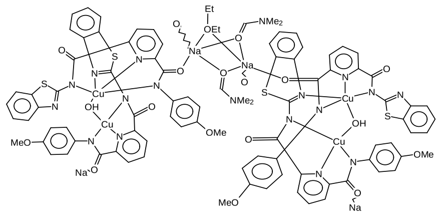

NARPII

Reference: D.Bansal, R.Gupta (2017) *Dalton Trans.* ,**46,**4617

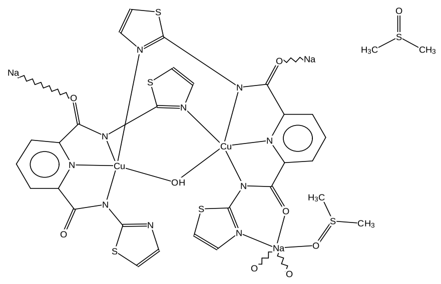

NARSIL

Reference: D.Bansal, R.Gupta (2017) *Dalton Trans.* ,**46,**4617

$Cu_2Na[\mu_3\text{-}C_{21}H_{11}N_5O_2S_2\text{-}\kappa^5N,N',N'',N''',N'''']$--
--$[\mu_3\text{-HO}]$--
--$[\mu_2\text{-}C_{21}H_{11}N_5O_2S_2\text{-}\kappa^4N,N',N'',N''']$--
--$[\mu_2\text{-}OSMe_2]$--
--$[CHO(NMe_2)][OSMe_2]$

OHC—NMe$_2$

NAYLOQ

Reference: G.L.Guillet, F.T.Sloane, M.F.Dumont, K.A.Abboud, L.J.Murray (2012) *Dalton Trans.* ,**41**,7866

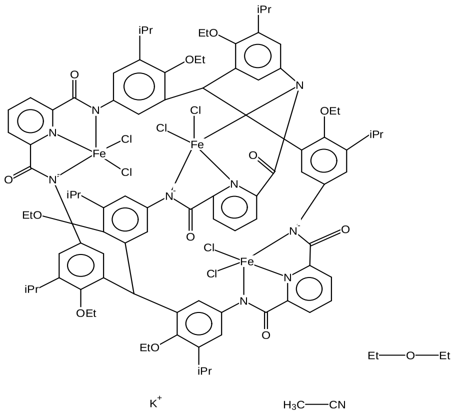

NERXIU

Reference: P.Pirovano, A.R.Berry, M.Swart, A.R.McDonald (2018) *Dalton Trans.* ,**47**,246

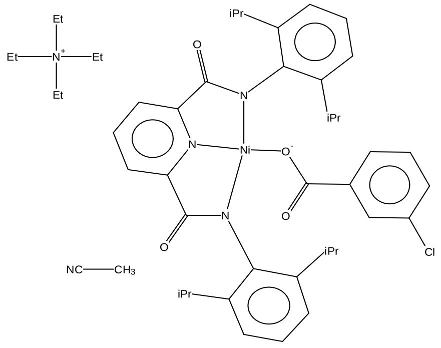

NERXOA

Reference: P.Pirovano, A.R.Berry, M.Swart, A.R.McDonald (2018) *Dalton Trans.* ,**47**,246

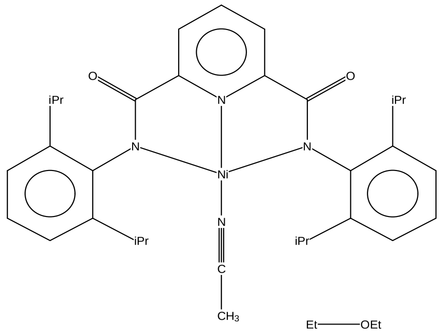

NEVMOQ

Reference: M.Ray, D.Ghosh, Z.Shirin, R.Mukherjee (1997) *Inorg.Chem.* ,**36**,3568

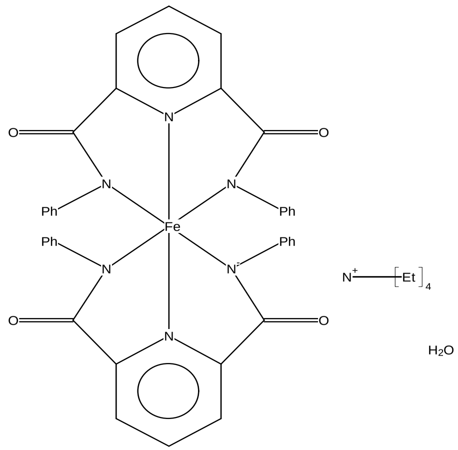

NEVMUW

Reference: M.Ray, D.Ghosh, Z.Shirin, R.Mukherjee (1997) *Inorg.Chem.* ,**36**,3568

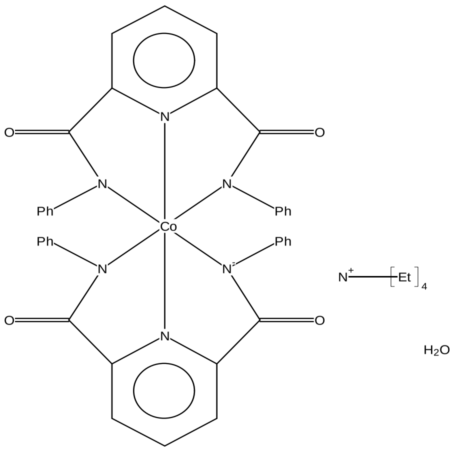

NEVNAE

Reference: Jian Ying Qi, Qi Yun Yang, Zhong Yuan Zhou, A.S.C.Chan (2007) *Acta Crystallogr.,Sect.E:Struct.Rep.Online* ,**63,** m159

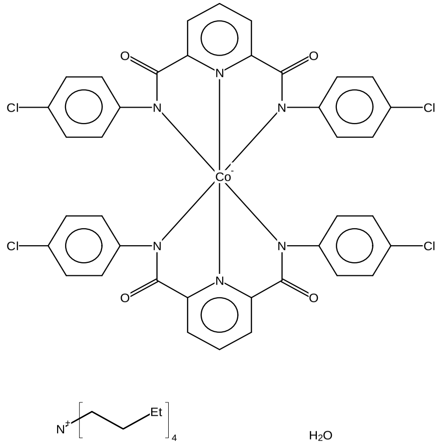

NIDJEQ

Reference: M.J.Rose, A.K.Patra, E.A.Alcid, M.M.Olmstead, P.K.Mascharak (2007) *Inorg.Chem.* ,**46**,2328

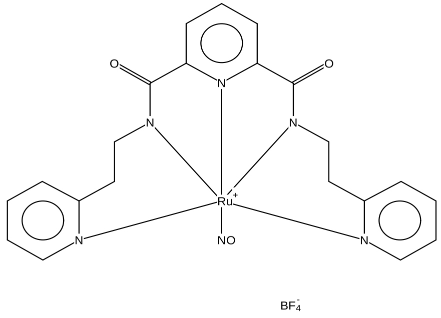

NIDJIU

Reference: M.J.Rose, A.K.Patra, E.A.Alcid, M.M.Olmstead, P.K.Mascharak (2007) *Inorg.Chem.* ,**46**,2328

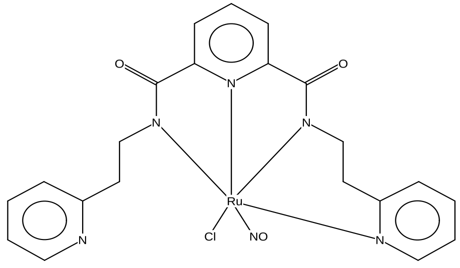

NIDJOA

Reference: M.J.Rose, A.K.Patra, E.A.Alcid, M.M.Olmstead, P.K.Mascharak (2007) *Inorg.Chem.* ,**46,**2328

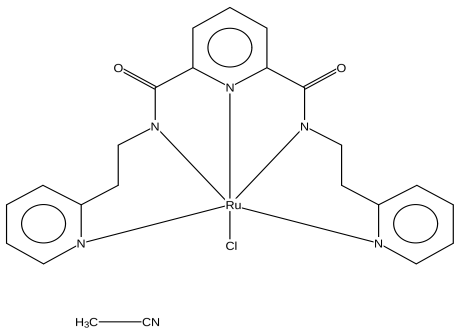

NIJYEK

Reference: D.S.Marlin, M.M.Olmstead, P.K.Mascharak (2001) *Inorg.Chem.* ,**40,**7003

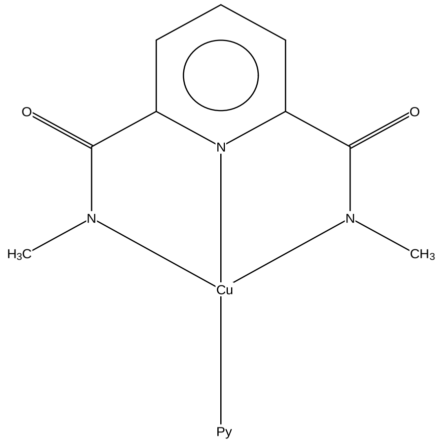

NIJYIO

Reference: D.S.Marlin, M.M.Olmstead, P.K.Mascharak (2001) *Inorg.Chem.* ,**40,**7003

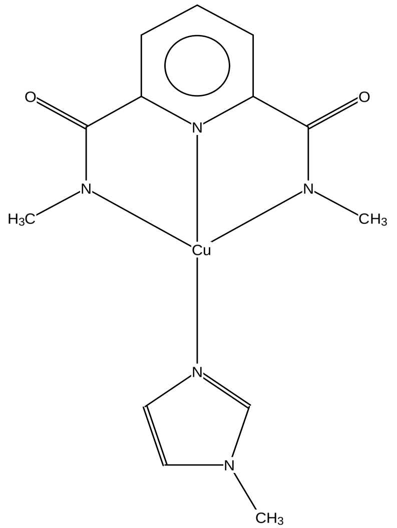

NIKSOP

Reference: D.S.Marlin, M.M.Olmstead, P.K.Mascharak (2001) *Inorg.Chem.* ,**40,**7003

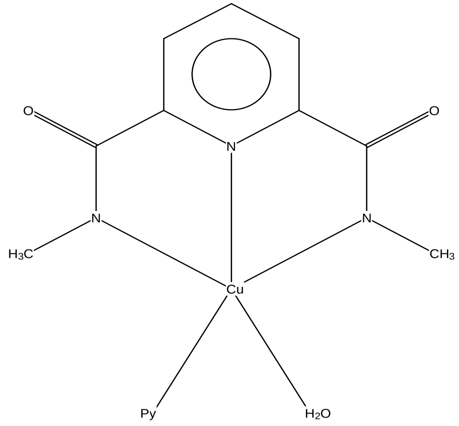

NIKWIN

Reference: D.S.Marlin, M.M.Olmstead, P.K.Mascharak (2001) *Inorg.Chem.* ,**40,**7003

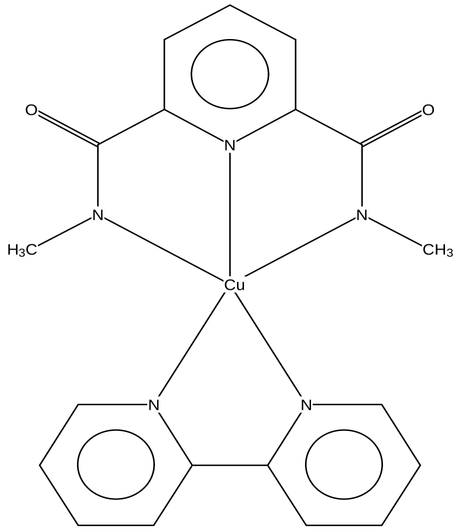

NILLAV

Reference: D.S.Marlin, M.M.Olmstead, P.K.Mascharak (2001) *Inorg.Chem.* ,**40,**7003

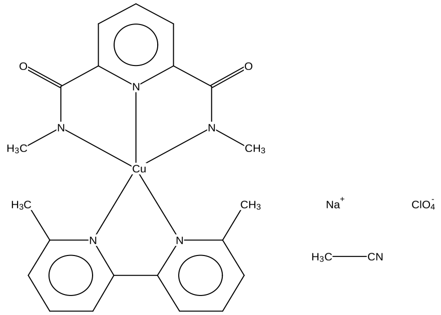

NIMHAS

Reference: D.S.Marlin, M.M.Olmstead, P.K.Mascharak (2001) *Inorg.Chem.* ,**40,**7003

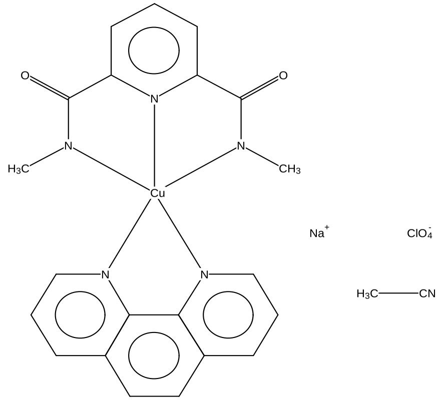

NIMHEW

Reference: D.S.Marlin, M.M.Olmstead, P.K.Mascharak (2001) *Inorg.Chem.* ,**40,**7003

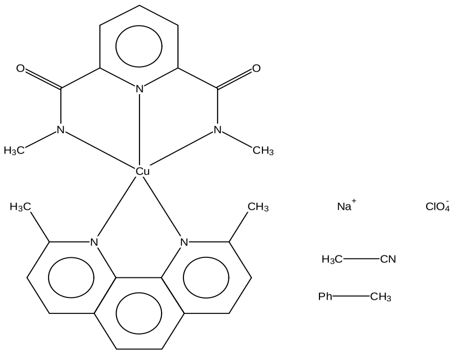

NIMYEQ

Reference: P.Melle, Y.Manoharan, M.Albrecht (2018) *Inorg.Chem.* ,**57**, 11761

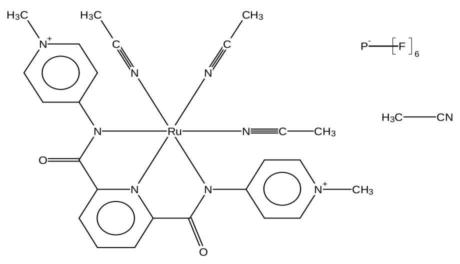

NIMYIU

Reference: P.Melle, Y.Manoharan, M.Albrecht (2018) *Inorg.Chem.* ,**57**, 11761

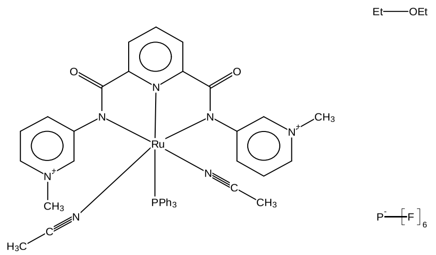

NIMYOA

Reference: P.Melle, Y.Manoharan, M.Albrecht (2018) *Inorg.Chem.* ,**57**, 11761

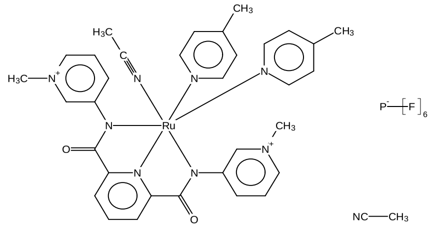

NIMYUG

Reference: P.Melle, Y.Manoharan, M.Albrecht (2018) *Inorg.Chem.* ,**57**, 11761

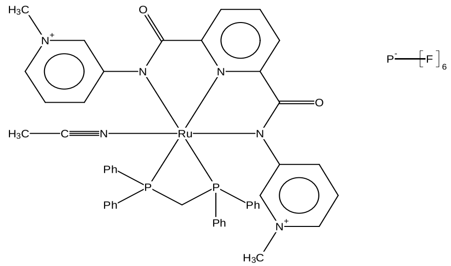

NIMZAN

Reference: P.Melle, Y.Manoharan, M.Albrecht (2018) *Inorg.Chem.* ,**57**, 11761

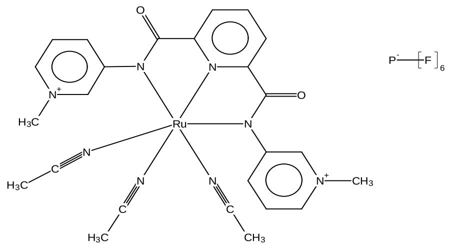

NISBAV

Reference: S.Pandey, P.Kumar, R.Gupta (2018) *Dalton Trans.* ,**47**, 14686

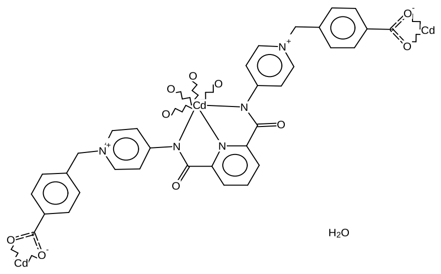

NISBEZ

Reference: S.Pandey, P.Kumar, R.Gupta (2018) *Dalton Trans.* ,**47**, 14686

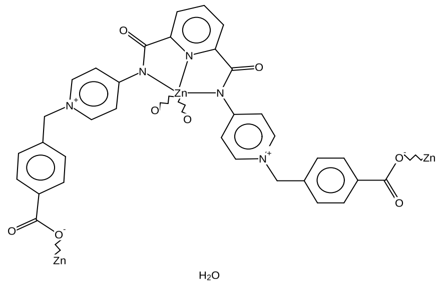

NISBID

Reference: S.Pandey, P.Kumar, R.Gupta (2018) *Dalton Trans.* ,**47**, 14686

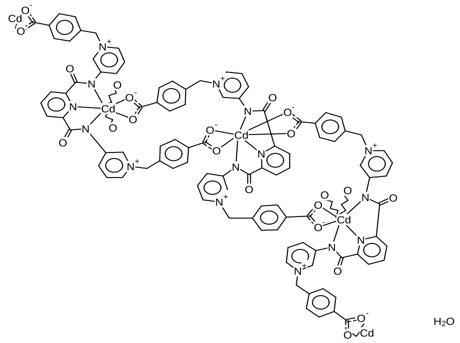

NIVYOH

Reference: S.Ghosh, B.Roehm, R.A.Begum, J.Kut, Md.A.Hossain, V.W.Day, K.Bowman-James (2007) *Inorg.Chem.* ,**46,**9519

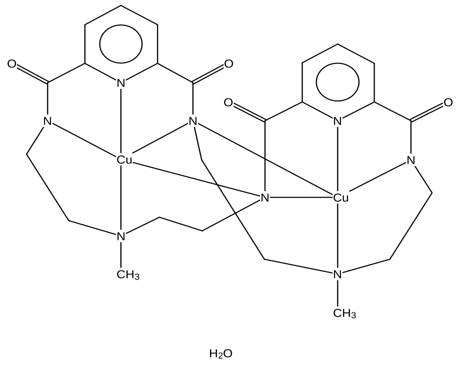

NIVYUN

Reference: S.Ghosh, B.Roehm, R.A.Begum, J.Kut, Md.A.Hossain, V.W.Day, K.Bowman-James (2007) *Inorg.Chem.* ,**46,**9519

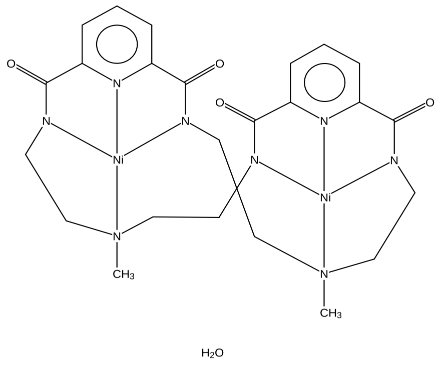

NIVZAU

Reference: S.Ghosh, B.Roehm, R.A.Begum, J.Kut, Md.A.Hossain, V.W.Day, K.Bowman-James (2007) *Inorg.Chem.* ,**46,**9519

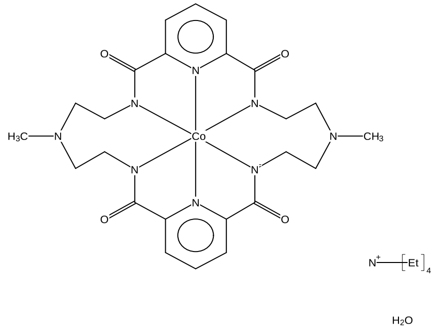

NIVZEY

Reference: S.Ghosh, B.Roehm, R.A.Begum, J.Kut, Md.A.Hossain, V.W.Day, K.Bowman-James (2007) *Inorg.Chem.* ,**46,**9519

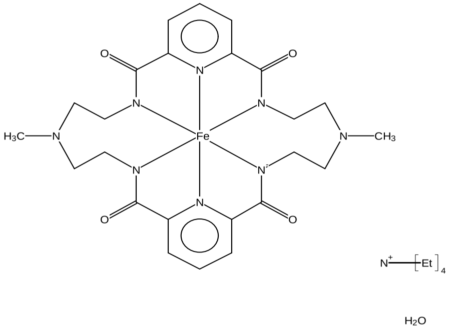

NOQSOC

Reference: Quanfu Cao, Dacheng Li (2009) *Acta Crystallogr.,Sect.E:Struct.Rep.Online* ,**65,**m464

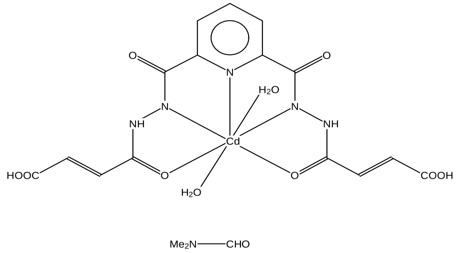

NURJAM

Reference: N.Miyagawa, M.Watanabe, T.Matsuyama, Y.Koyama, T.Moriuchi, T.Hirao, Y.Furusho, T.Takata (2010) *Chem.Commun.* ,**46,** 1920

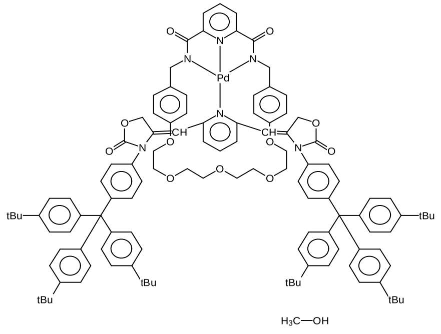

NURJEQ

Reference: N.Miyagawa, M.Watanabe, T.Matsuyama, Y.Koyama, T.Moriuchi, T.Hirao, Y.Furusho, T.Takata (2010) *Chem.Commun.* ,**46,** 1920

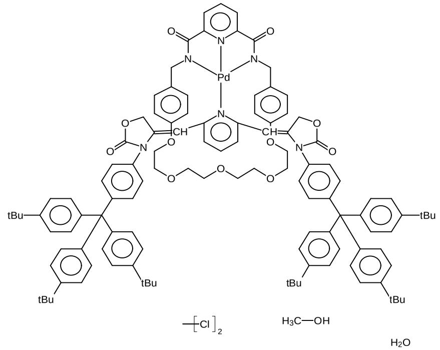

OBOMOL

Reference: S.S.P.Malaza, G.Mehlana, Ocean Cheung, R.Hunter, B.C.E.Makhubela (2021) *CrystEngComm* ,

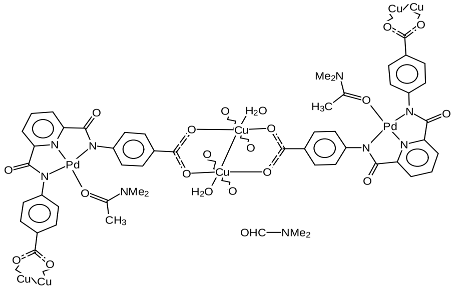

OCOTUV

Reference: L.A.Tyler, M.M.Olmstead, P.K.Mascharak (2001) *Inorg.Chim.Acta* ,**321,**135

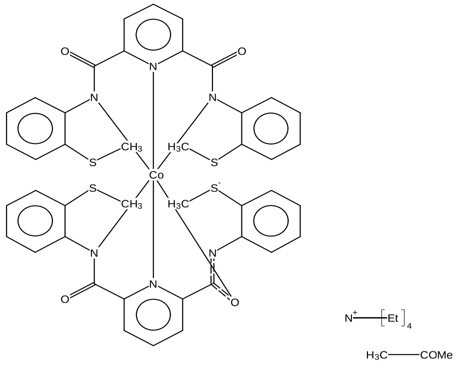

OCOVAD

Reference: L.A.Tyler, M.M.Olmstead, P.K.Mascharak (2001) *Inorg.Chim.Acta* ,**321,**135

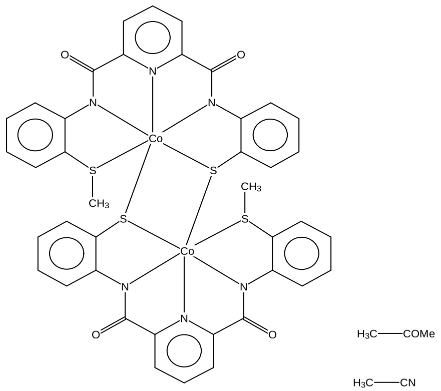

OCOVEH

Reference: L.A.Tyler, M.M.Olmstead, P.K.Mascharak (2001) *Inorg.Chim.Acta* ,**321,**135

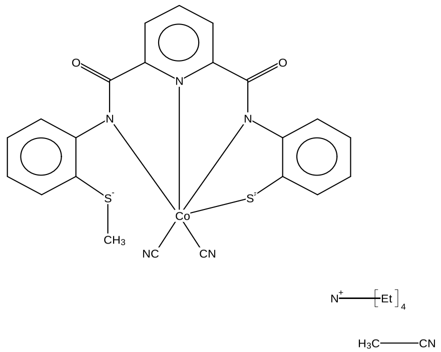

OJEZAG

Reference: D.S.Marlin, P.Maschrak, M.M.Olmstead (2016) *CSD Communication(Private Communication)* ,

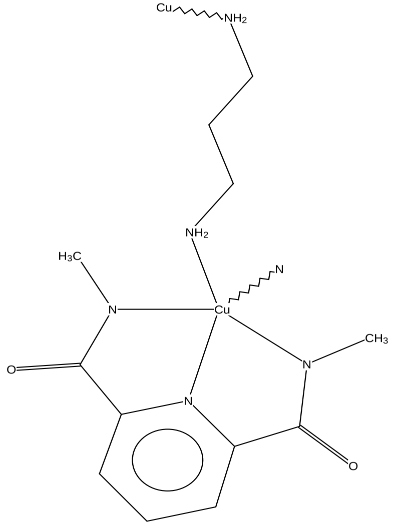

OKARAV

Reference: N.Hussain, V.K.Bhardwaj (2016) *Dalton Trans.* ,**45,**7697

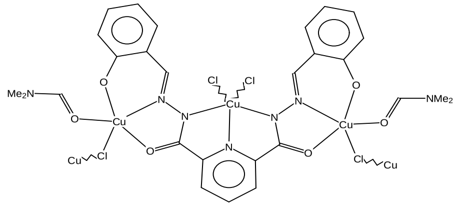

OKAREZ

Reference: N.Hussain, V.K.Bhardwaj (2016) *Dalton Trans.* ,**45,**7697

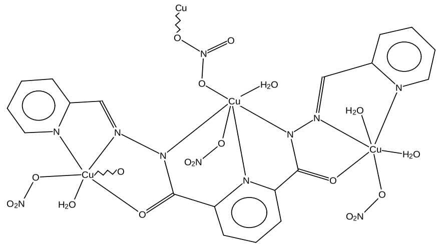

OKIGOF

Reference: Fangfang Pan, Jie Wu, Hongwei Hou, Yaoting Fan (2010) *Cryst.Growth Des.* ,**10,**3835

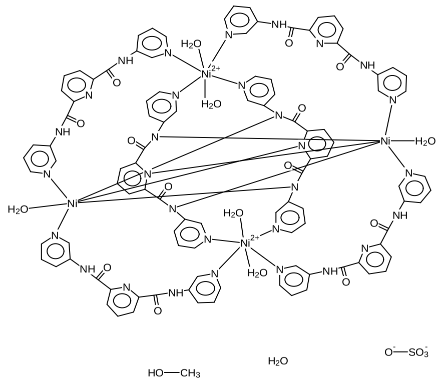

OLANAS

Reference: S.Srivastava, V.Kumar, R.Gupta (2016) *Cryst.Growth Des.* ,**16,**2874

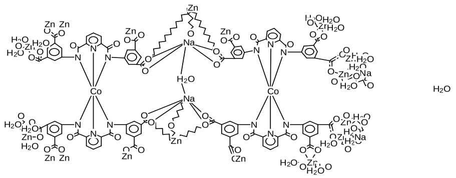

OLANEW

Reference: S.Srivastava, V.Kumar, R.Gupta (2016) *Cryst.Growth Des.* ,**16**,2874

n-$Cd_8Co_2[\mu_8\text{-}C_{23}H_9N_3O_{10}][\mu_7\text{-}C_{23}H_9N_3O_{10}]$--
--$[\mu_6\text{-}C_{23}H_9N_3O_{10}]$--
--$[\mu_4\text{-}C_{21}H_{11}N_3O_6(COO)_2\text{-}\kappa^3N,N',N'',\kappa^5O,O',O'',O''',O'''']$--
--$[\mu_2\text{-}H_2O][H_2O]_{20}$

H_2O

OLANIA

Reference: S.Srivastava, V.Kumar, R.Gupta (2016) *Cryst.Growth Des.* ,**16**,2874

n-$Co_2Mn_8Na_2[\mu_8\text{-}C_{23}H_{10}N_3O_{10}][\mu_7\text{-}C_{23}H_9N_3O_{10}]$--
--$[\mu_7\text{-}C_{23}H_{10}N_3O_{10}]$--
--$[\mu_5\text{-}C_{23}H_9N_3O_{10}]$--
--$[\mu_3\text{-}H_2O]$--
--$[\mu_2\text{-}H_2O][H_2O]_{25}$

H_2O

OMUHUA

Reference: N.P.Chmel, L.E.N.Allan, J.M.Becker, G.J.Clarkson, S.S.Turner, P.Scott (2011) *Dalton Trans.* ,**40**,1722

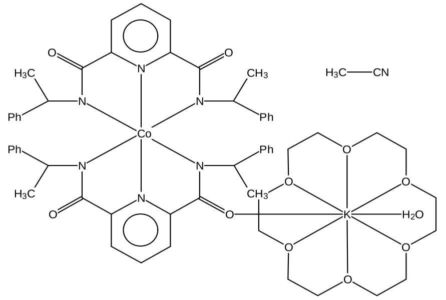

OMUJAI

Reference: N.P.Chmel, L.E.N.Allan, J.M.Becker, G.J.Clarkson, S.S.Turner, P.Scott (2011) *Dalton Trans.* ,**40**,1722

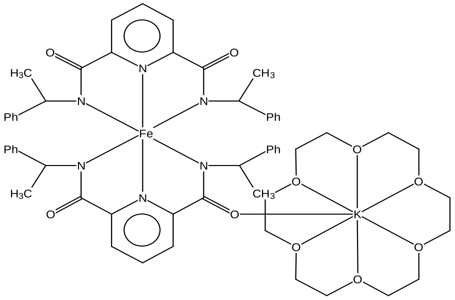

OMUJEM

Reference: N.P.Chmel, L.E.N.Allan, J.M.Becker, G.J.Clarkson, S.S.Turner, P.Scott (2011) *Dalton Trans.* ,**40**,1722

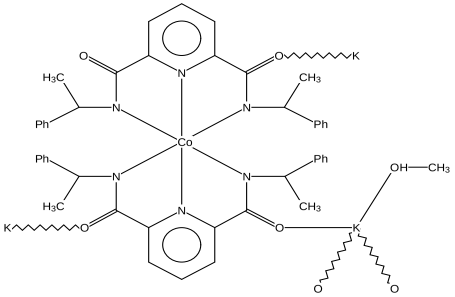

OMUJIQ

Reference: N.P.Chmel, L.E.N.Allan, J.M.Becker, G.J.Clarkson, S.S.Turner, P.Scott (2011) *Dalton Trans.* ,**40**,1722

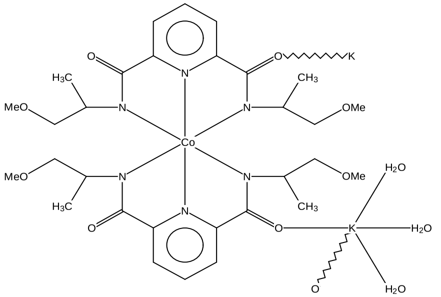

OMUJOW

Reference: N.P.Chmel, L.E.N.Allan, J.M.Becker, G.J.Clarkson, S.S.Turner, P.Scott (2011) *Dalton Trans.* ,**40**,1722

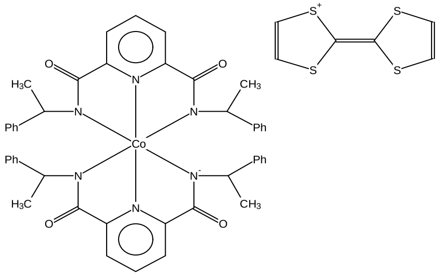

OMUJUC

Reference: N.P.Chmel, L.E.N.Allan, J.M.Becker, G.J.Clarkson, S.S.Turner, P.Scott (2011) *Dalton Trans.* ,**40**,1722

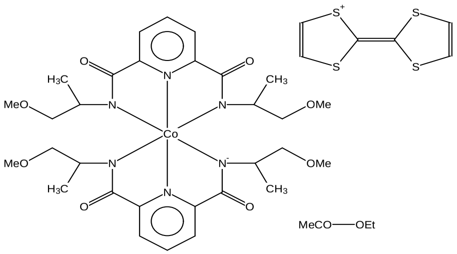

OPEJEA

Reference: B.Nisar Ahamed, R.Duchene, K.Robeyns, C.-A.Fustin (2016) *Chem.Commun.* ,**52**,2149

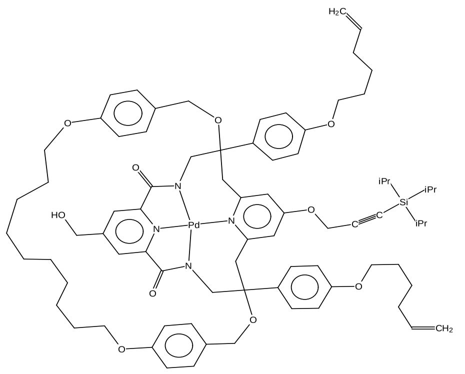

OPEJIE

Reference: B.Nisar Ahamed, R.Duchene, K.Robeyns, C.-A.Fustin (2016) *Chem.Commun.* ,**52**,2149

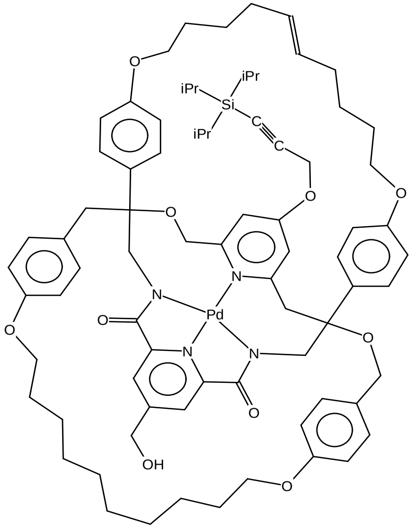

OPEJOK

Reference: B.Nisar Ahamed, R.Duchene, K.Robeyns, C.-A.Fustin (2016) *Chem.Commun.* ,**52**,2149

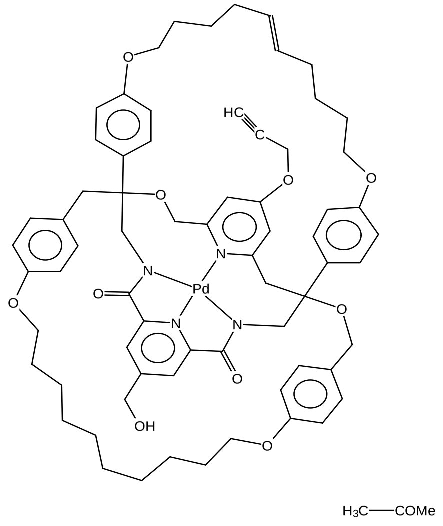

OPIHUT

Reference: Wen Wu, J.T.De Hont, R.Parveen, B.Vlaisavljevich, W.B.Tolman (2021) *Inorg.Chem.* ,**60**,5217

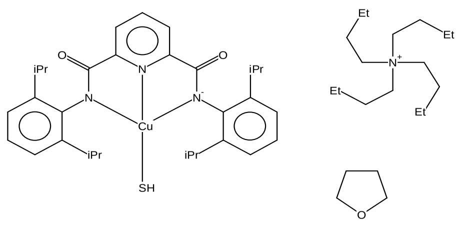

OPIJAB

Reference: Wen Wu, J.T.De Hont, R.Parveen, B.Vlaisavljevich, W.B.Tolman (2021) *Inorg.Chem.* ,**60**,5217

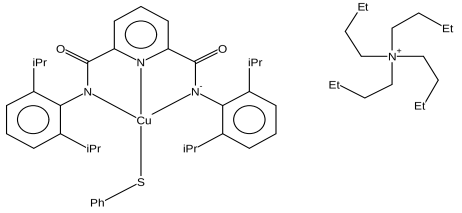

OPOKAG

Reference: Y.Perez, A.L.Johnson, P.R.Raithby (2011) *Polyhedron* ,**30**, 284

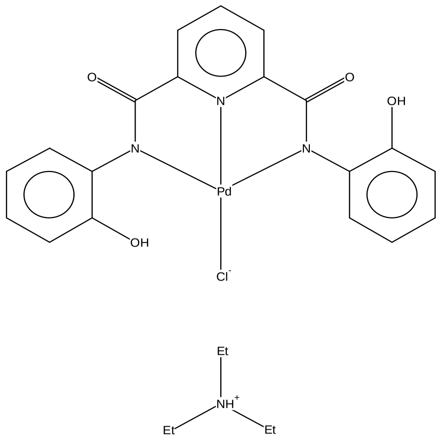

OPOKEK

Reference: Y.Perez, A.L.Johnson, P.R.Raithby (2011) *Polyhedron* ,**30**, 284

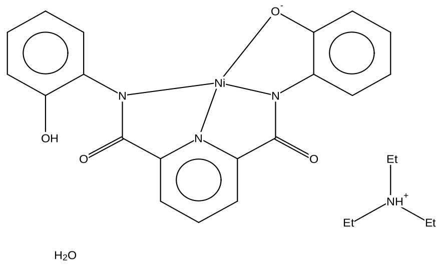

ORIWOD

Reference: K.Majee, J.Patel, S.Rai, B.Das, B.Panda, S.K.Padhi (2016) *Phys.Chem.Chem.Phys.(PCCP)* ,**18**,21640

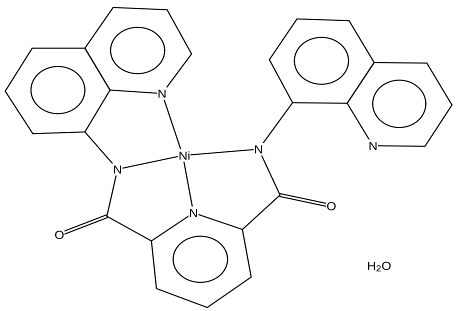

OSIDAW

Reference: P.J.Donoghue, A.K.Gupta, D.W.Boyce, C.J.Cramer, W.B.Tolman (2010) *J.Am.Chem.Soc.* ,**132,**15869

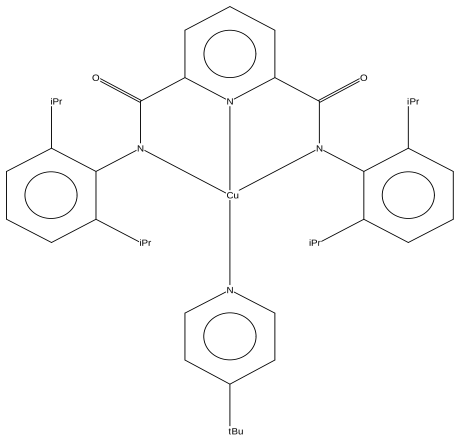

PADNEP

Reference: Deguang Huang, R.H.Holm (2010) *J.Am.Chem.Soc.* ,**132,** 4693

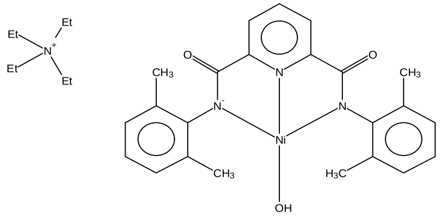

PADNIT

Reference: Deguang Huang, R.H.Holm (2010) *J.Am.Chem.Soc.* ,**132,** 4693

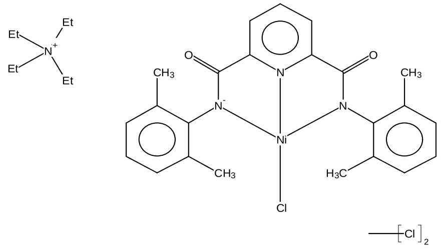

PADNOZ

Reference: Deguang Huang, R.H.Holm (2010) *J.Am.Chem.Soc.* ,**132,** 4693

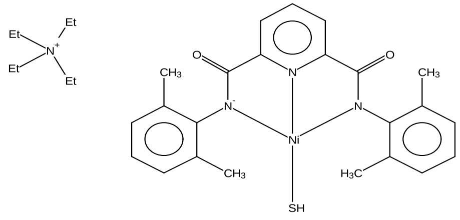

PADNUF

Reference: Deguang Huang, R.H.Holm (2010) *J.Am.Chem.Soc.* ,**132,** 4693

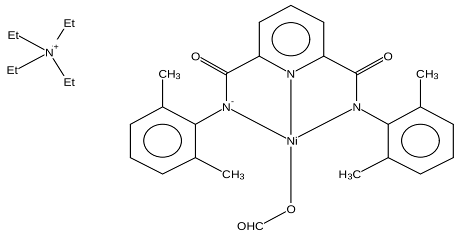

PADPAN

Reference: Deguang Huang, R.H.Holm (2010) *J.Am.Chem.Soc.* ,**132,** 4693

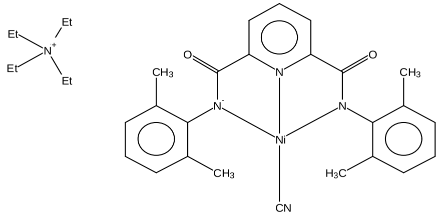

PADPER

Reference: Deguang Huang, R.H.Holm (2010) *J.Am.Chem.Soc.* ,**132,** 4693

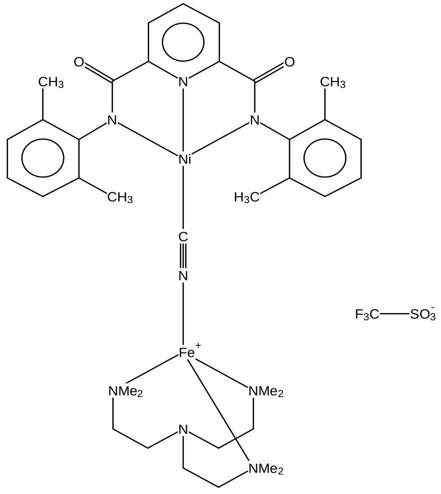

PADPOB

Reference: Deguang Huang, R.H.Holm (2010) *J.Am.Chem.Soc.* ,**132,** 4693

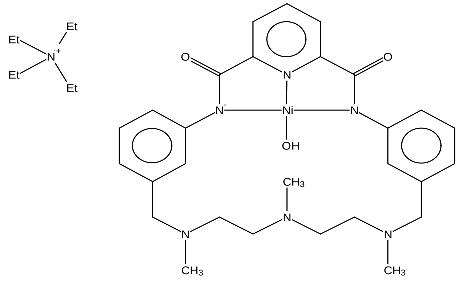

PADPUH

Reference: Deguang Huang, R.H.Holm (2010) *J.Am.Chem.Soc.* ,**132,** 4693

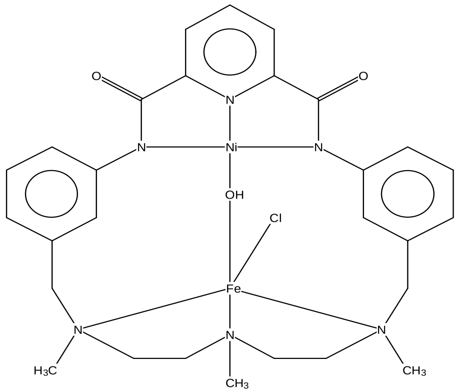

PADQAO

Reference: Deguang Huang, R.H.Holm (2010) *J.Am.Chem.Soc.* ,**132,** 4693

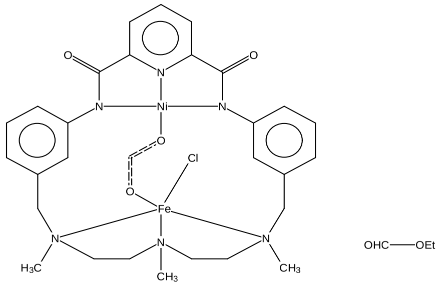

PADQES

Reference: Deguang Huang, R.H.Holm (2010) *J.Am.Chem.Soc.* ,**132,** 4693

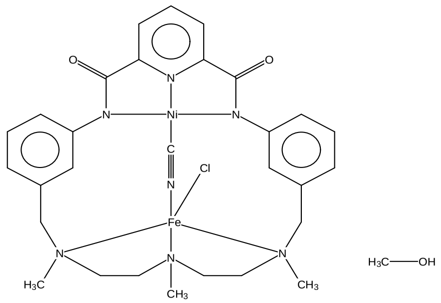

PADQIW

Reference: Deguang Huang, R.H.Holm (2010) *J.Am.Chem.Soc.* ,**132,** 4693

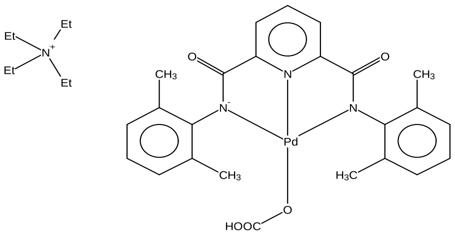

PADQOC

Reference: Deguang Huang, R.H.Holm (2010) *J.Am.Chem.Soc.* ,**132,** 4693

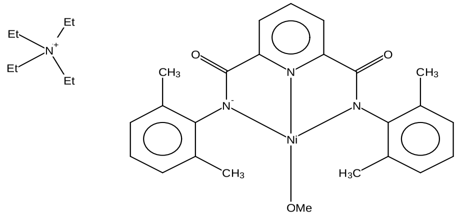

PADQUI

Reference: Deguang Huang, R.H.Holm (2010) *J.Am.Chem.Soc.* ,**132,** 4693

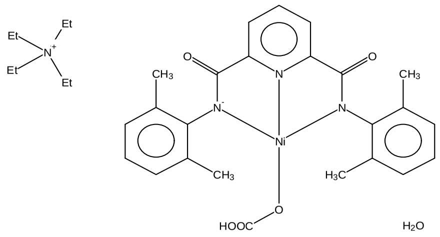

PADRAP

Reference: Deguang Huang, R.H.Holm (2010) *J.Am.Chem.Soc.* ,**132,** 4693

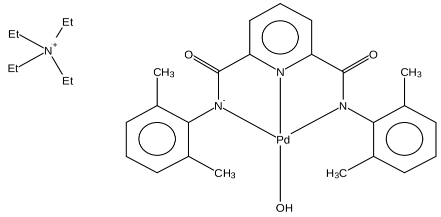

PAFHEK

Reference: A.-M.Fuller, D.A.Leigh, P.J.Lusby, I.D.H.Oswald, S.Parsons, D.B.Walker (2004) *Angew.Chem.,Int.Ed.* ,**43,**3914

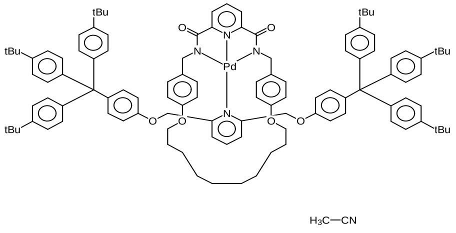

PALJAO

Reference: Chun-Xiang Wang, Chen-Xia Du, Yao-Hui Li, Yang-Jie Wu (2005) *Inorg.Chem.Commun.* ,**8**,379

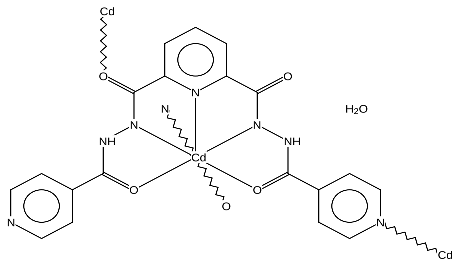

PIDYUX

Reference: Sheng-Gui Liu, Chun-Lin Ni (2007) *Acta Crystallogr.,Sect.E:Struct.Rep.Online* ,**63**,m1373

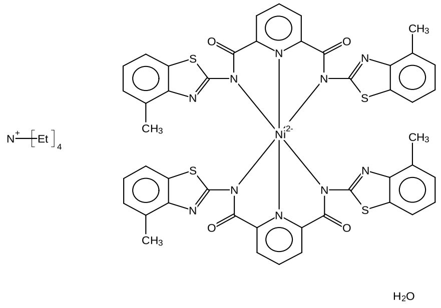

PIWBOM

Reference: F.A.Chavez, M.M.Olmstead, P.K.Mascharak (1997) *Inorg.Chem.* ,**36**,6323

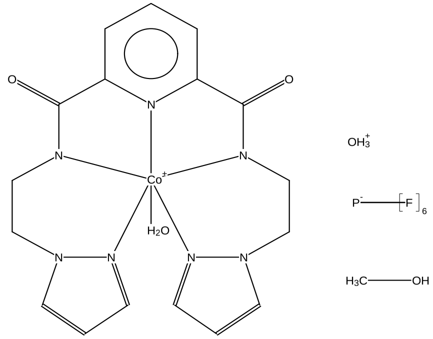

PIWFOQ

Reference: F.A.Chavez, M.M.Olmstead, P.K.Mascharak (1997) *Inorg.Chem.* ,**36**,6323

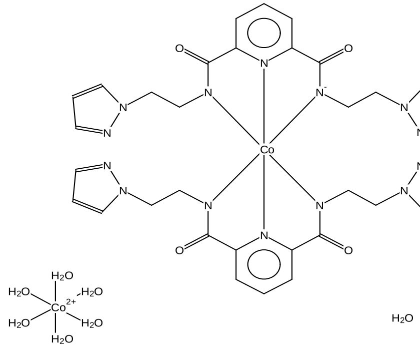

POMFIJ

Reference: Sheng-Nan Lu, Wen-Shi Wu, Feng-Xiang Shi, Zhi-Long Quan (2019) *Wuji Huaxue Xuebao(Chin.)(Chin.J.Inorg.Chem.)* ,**35**,837

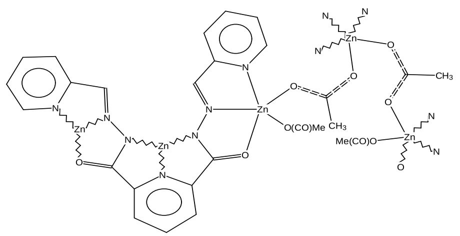

PUJTOF01

Reference: N.Hussain, V.K.Bhardwaj (2016) *Dalton Trans.* ,**45**,7697

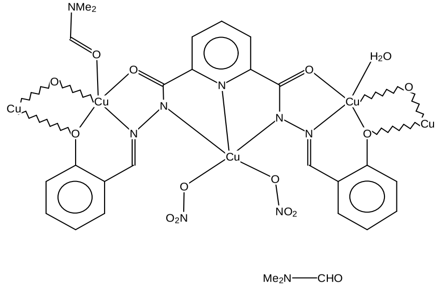

PUSKET

Reference: F.A.Chavez, M.M.Olmstead, P.K.Mascharak (1998) *Inorg.Chim.Acta* ,**269**,269

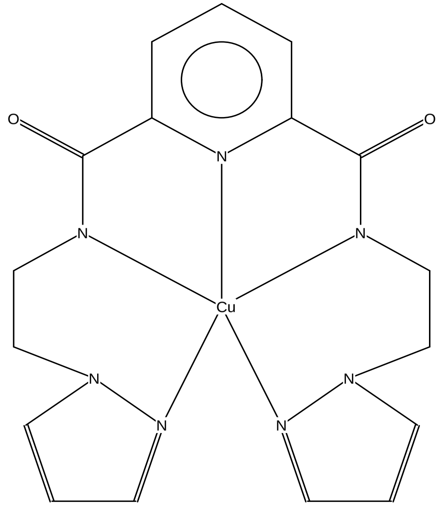

QAGQEV

Reference: T.C.Harrop, L.A.Tyler, M.M.Olmstead, P.K.Mascharak (2003) *Eur.J.Inorg.Chem.* ,475

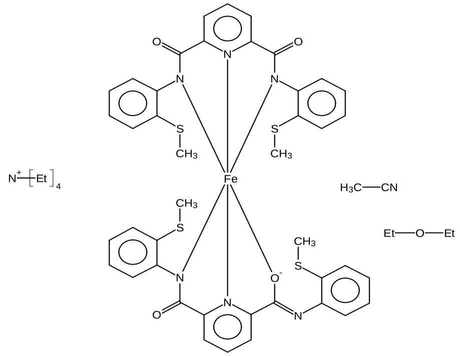

QAGQIZ

Reference: T.C.Harrop, L.A.Tyler, M.M.Olmstead, P.K.Mascharak (2003) *Eur.J.Inorg.Chem.* ,475

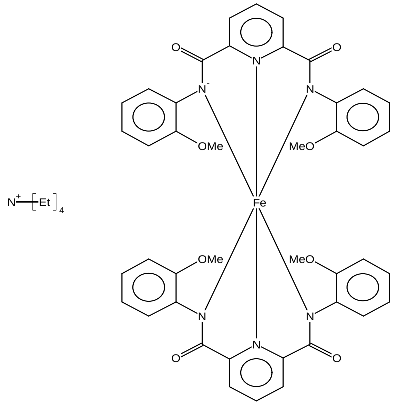

QALNOG

Reference: T.Moriuchi, S.Bandoh, M.Kamikawa, T.Hirao (2000) *Chem.Lett.* ,148

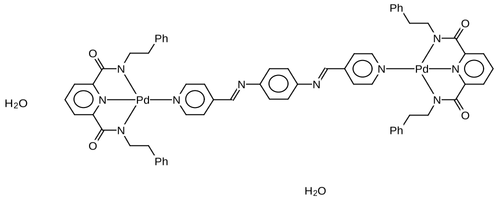

QAVGIE

Reference: A.-M.L.Fuller, D.A.Leigh, P.J.Lusby, A.M.Z.Slawin, D.B.Walker (2005) *J.Am.Chem.Soc.* ,**127,**12612

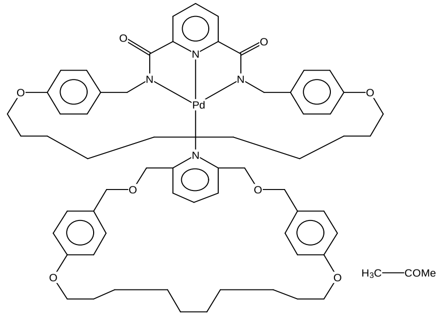

QAVGOK

Reference: A.-M.L.Fuller, D.A.Leigh, P.J.Lusby, A.M.Z.Slawin, D.B.Walker (2005) *J.Am.Chem.Soc.* ,**127,**12612

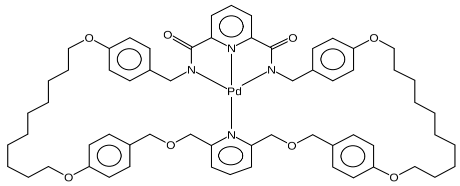

QAVGUQ

Reference: A.-M.L.Fuller, D.A.Leigh, P.J.Lusby, A.M.Z.Slawin, D.B.Walker (2005) *J.Am.Chem.Soc.* ,**127,**12612

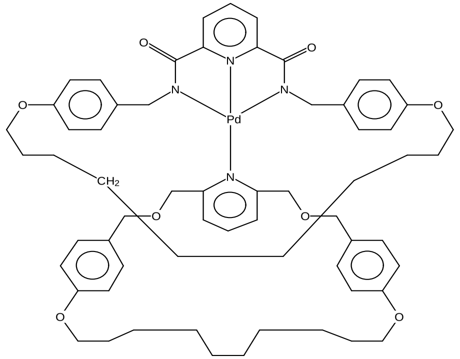

QAWNIN

Reference: Qi-Qiang Wang, R.A.Begum, V.W.Day, K.Bowman-James (2012) *Inorg.Chem.* ,**51,**760

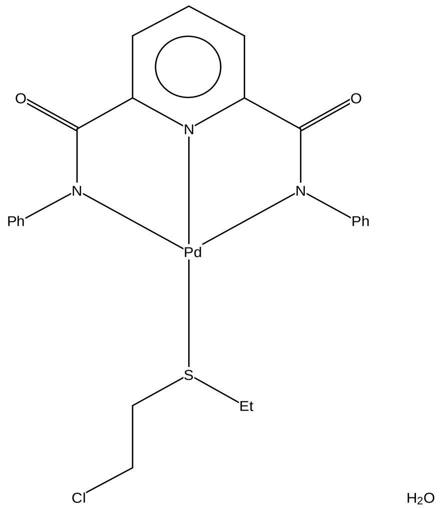

QEJTEH

Reference: Chien Thang Pham, Hung Huy Nguyen, A.Hagenbach, U.Abram (2017) *Inorg.Chem.* ,**56,**11406

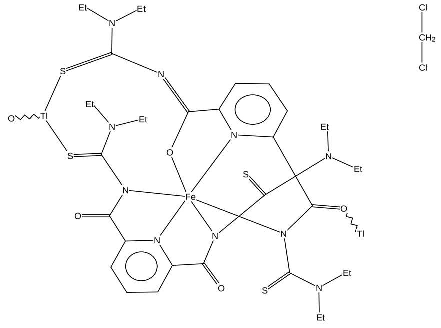

QERSIP

Reference: T.Moriuchi, S.Bandoh, M.Miyaishi, T.Hirao (2001) *Eur.J.Inorg.Chem.* ,651

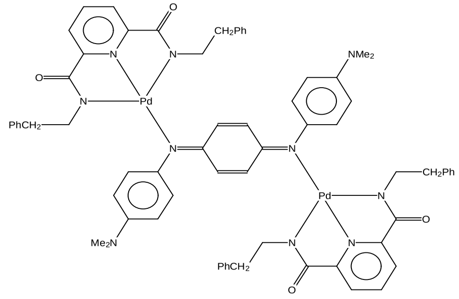

QIHCOC

Reference: Wen-Ju Liu, Hai-Hua Huang, Ting Ouyang, Long Jiang, Di-Chang Zhong, Wen Zhang, Tong-Bu Lu (2018) *Chem.-Eur.J.* ,**24,** 4503

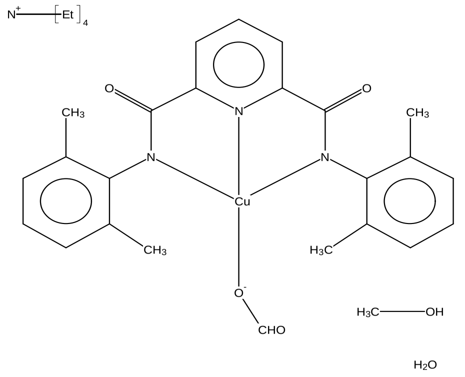

QIJSEJ

Reference: Yu-Fang Wang, Zhuo Li, Yan-Chun Sun, Jian-She Zhao (2014) *Synth.React.Inorg.,Met.-Org.,Nano-Met.Chem.* ,**44**,277

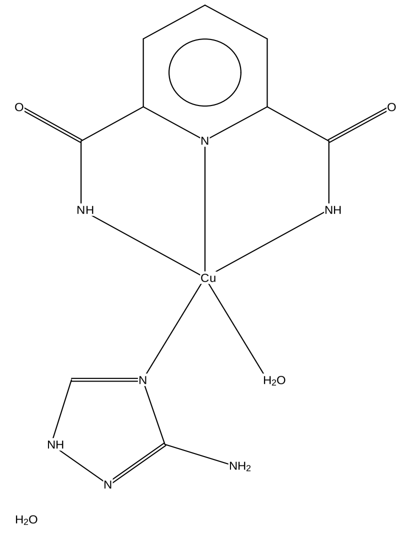

QITYUN

Reference: H.Kurosaki, R.K.Sharma, S.Aoki, T.Inoue, Y.Okamoto, Y.Sugiura, M.Doi, T.Ishida, M.Otsuka, M.Goto (2001) *J.Chem.Soc.,Dalton Trans.* ,441

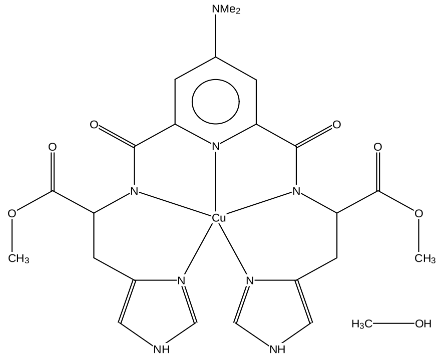

QOCXEO

Reference: Xiaofeng Zhang, Zilong Zhang, Shiqun Xiang, Yingzu Zhu, Changneng Chen, Deguang Huang (2019) *Inorg.Chem.Front.* ,**6**,1135

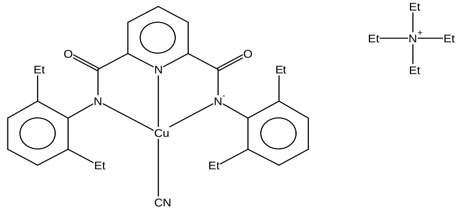

QOCXIS

Reference: Xiaofeng Zhang, Zilong Zhang, Shiqun Xiang, Yingzu Zhu, Changneng Chen, Deguang Huang (2019) *Inorg.Chem.Front.* ,**6**,1135

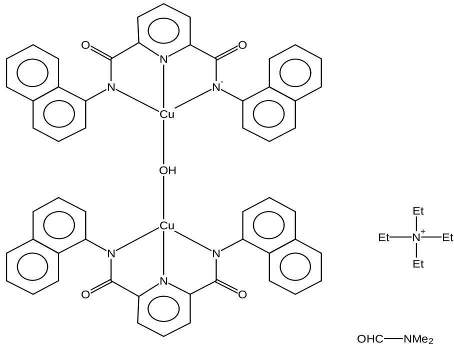

QOFLAB

Reference: Xiaofeng Zhang, Zilong Zhang, Shiqun Xiang, Yingzu Zhu, Changneng Chen, Deguang Huang (2019) *Inorg.Chem.Front.* ,**6**,1135

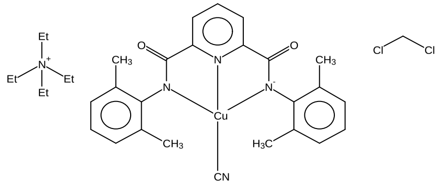

QOFLEF

Reference: Xiaofeng Zhang, Zilong Zhang, Shiqun Xiang, Yingzu Zhu, Changneng Chen, Deguang Huang (2019) *Inorg.Chem.Front.* ,**6**,1135

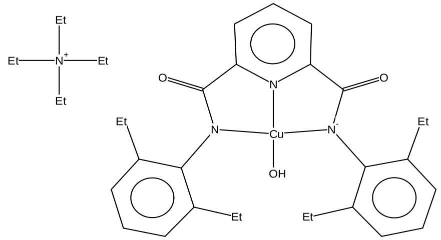

QOFLIJ

Reference: Xiaofeng Zhang, Zilong Zhang, Shiqun Xiang, Yingzu Zhu, Changneng Chen, Deguang Huang (2019) *Inorg.Chem.Front.* ,**6**,1135

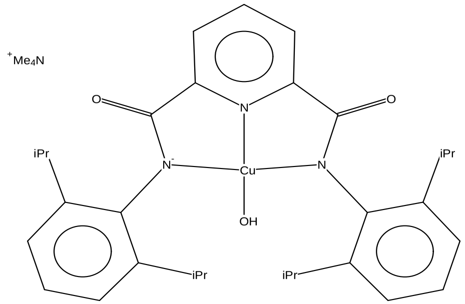

QOFLOP

Reference: Xiaofeng Zhang, Zilong Zhang, Shiqun Xiang, Yingzu Zhu, Changneng Chen, Deguang Huang (2019) *Inorg.Chem.Front.* ,**6**,1135

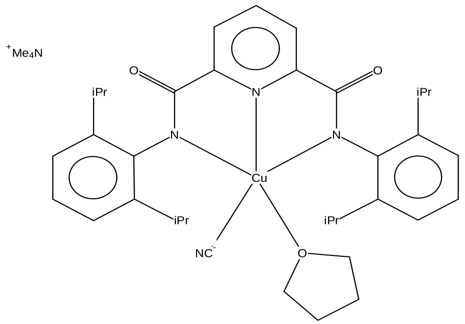

QOFLUV

Reference: Xiaofeng Zhang, Zilong Zhang, Shiqun Xiang, Yingzu Zhu, Changneng Chen, Deguang Huang (2019) *Inorg.Chem.Front.* ,**6**,1135

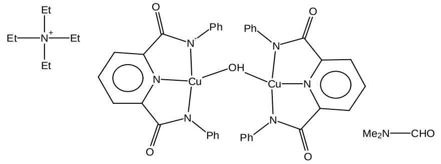

QOFMAC

Reference: Xiaofeng Zhang, Zilong Zhang, Shiqun Xiang, Yingzu Zhu, Changneng Chen, Deguang Huang (2019) *Inorg.Chem.Front.* ,**6**,1135

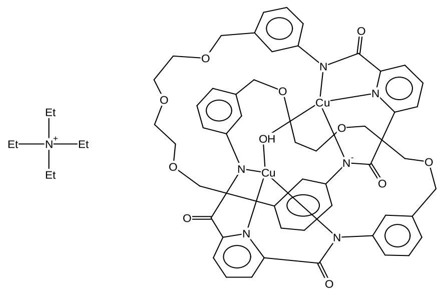

QOFMEG

Reference: Xiaofeng Zhang, Zilong Zhang, Shiqun Xiang, Yingzu Zhu, Changneng Chen, Deguang Huang (2019) *Inorg.Chem.Front.* ,**6**,1135

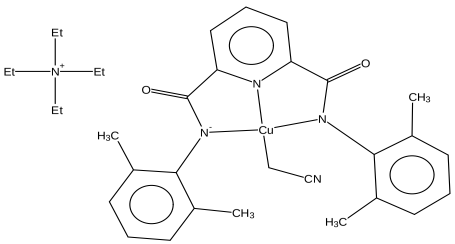

QOFMIK

Reference: Xiaofeng Zhang, Zilong Zhang, Shiqun Xiang, Yingzu Zhu, Changneng Chen, Deguang Huang (2019) *Inorg.Chem.Front.* ,**6**,1135

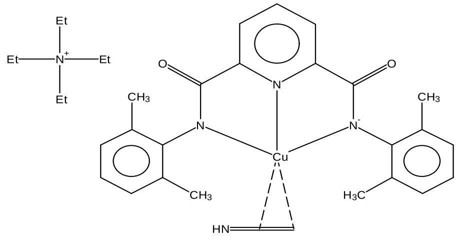

QULRIB

Reference: J.D.Schneider, B.A.Smith, G.A.Williams, D.R.Powell, F.Perez, G.T.Rowe, Lei Yang (2020) *Inorg.Chem.* ,**59**,5433

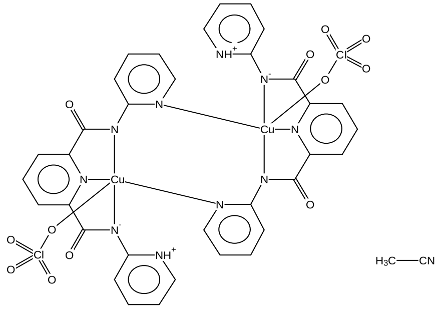

QULROH

Reference: J.D.Schneider, B.A.Smith, G.A.Williams, D.R.Powell, F.Perez, G.T.Rowe, Lei Yang (2020) *Inorg.Chem.* ,**59**,5433

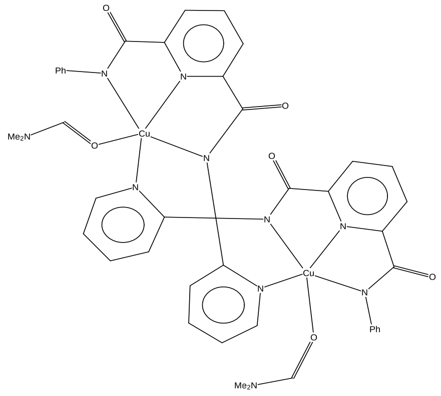

QULRUN

Reference: J.D.Schneider, B.A.Smith, G.A.Williams, D.R.Powell, F.Perez, G.T.Rowe, Lei Yang (2020) *Inorg.Chem.* ,**59**,5433

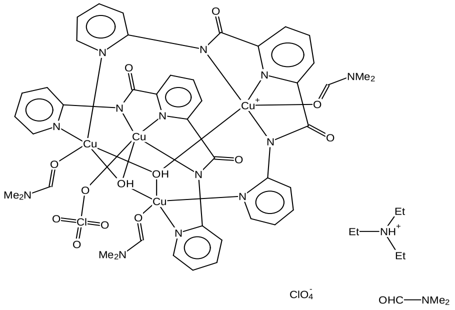

QULSAU

Reference: J.D.Schneider, B.A.Smith, G.A.Williams, D.R.Powell, F.Perez, G.T.Rowe, Lei Yang (2020) *Inorg.Chem.* ,**59**,5433

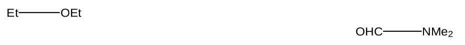

$Cu_5[\mu_5\text{-}O][\mu_3\text{-}C_{17}H_{11}N_5O_2\text{-}\kappa^5N,N',N'',N''',N'''']_3$

RADKEQ

Reference: P.Melle, Jan Thiede, D.A.Hey, M.Albrecht (2020) *Chem.-Eur.J.* ,**26**,13226

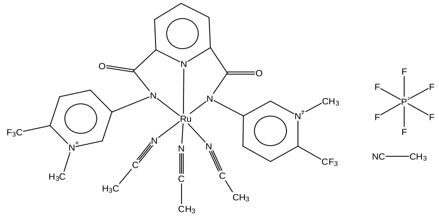

RADKIU

Reference: P.Melle, Jan Thiede, D.A.Hey, M.Albrecht (2020) *Chem.-Eur.J.* ,**26**,13226

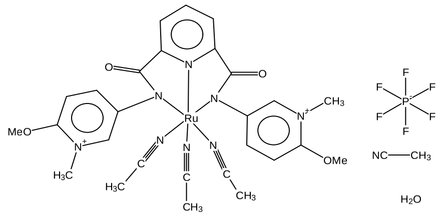

RAGJOZ

Reference: Yongli Wei, Hongwei Hou, Yaoting Fan, Yu Zhu (2004) *Eur.J.Inorg.Chem.* ,3946

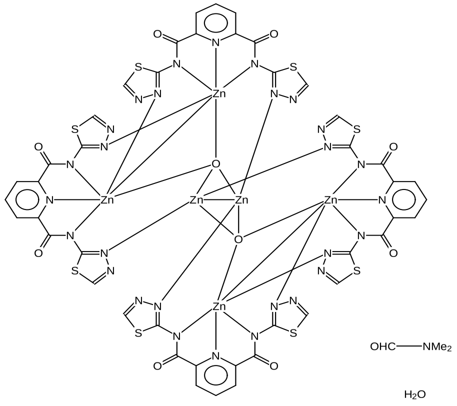

RAGJUF

Reference: Yongli Wei, Hongwei Hou, Yaoting Fan, Yu Zhu (2004) *Eur.J.Inorg.Chem.* ,3946

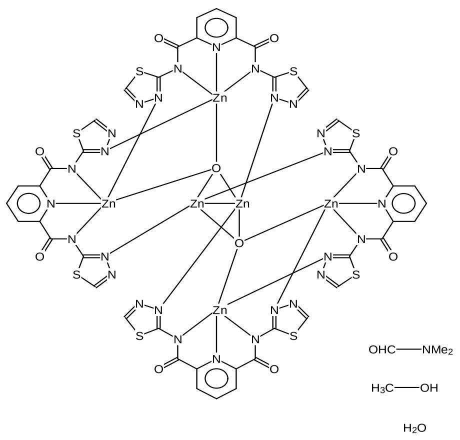

RAGKAM

Reference: Yongli Wei, Hongwei Hou, Yaoting Fan, Yu Zhu (2004) *Eur.J.Inorg.Chem.* ,3946

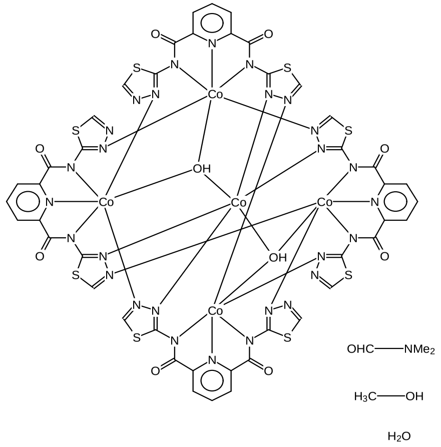

RAGKEQ

Reference: Yongli Wei, Hongwei Hou, Yaoting Fan, Yu Zhu (2004) *Eur.J.Inorg.Chem.* ,3946

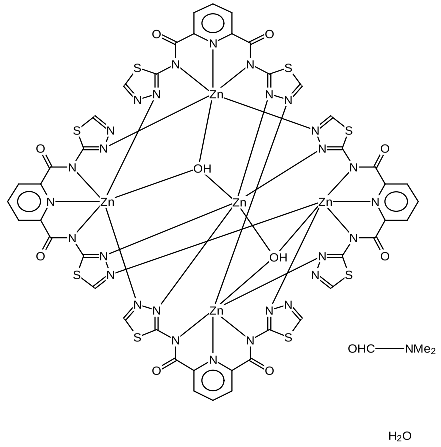

RAGKIU

Reference: Yongli Wei, Hongwei Hou, Yaoting Fan, Yu Zhu (2004) *Eur.J.Inorg.Chem.* ,3946

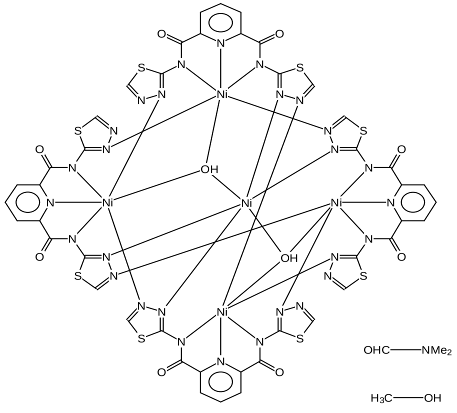

RAGKOA

Reference: Yongli Wei, Hongwei Hou, Yaoting Fan, Yu Zhu (2004) *Eur.J.Inorg.Chem.* ,3946

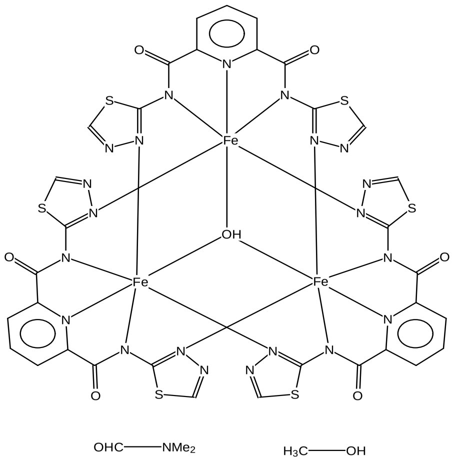

RAJMIA

Reference: A.P.Singh, G.Kumar, R.Gupta (2011) *Dalton Trans.* ,**40,** 12454

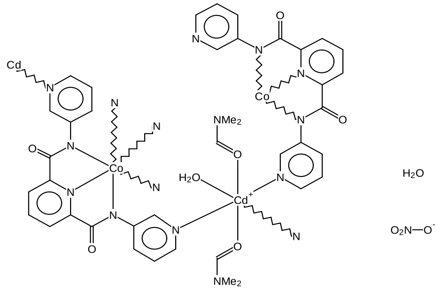

RAJMOG

Reference: A.P.Singh, G.Kumar, R.Gupta (2011) *Dalton Trans.* ,**40,** 12454

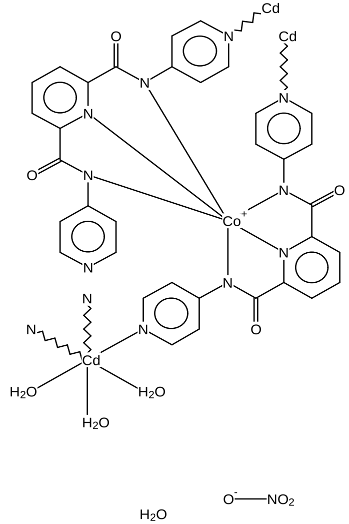

RASTUC

Reference: I.Lumb, M.S.Hundal, P.Mathur, M.Corbella, N.Aliaga-Alcalde, G.Hundal (2012) *Polyhedron* ,**36,**85

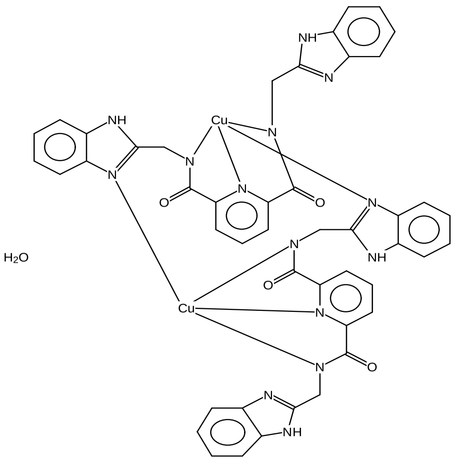

RAZPOZ

Reference: D.A.Leigh, P.J.Lusby, A.M.Z.Slawin, D.B.Walker (2012) *Chem.Commun.* ,**48,**5826

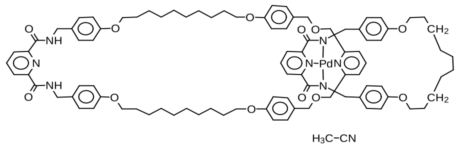

REBTID

Reference: A.J.Preston, J.C.Gallucci, J.R.Parquette (2006) *Org.Lett.* , **8,**5885

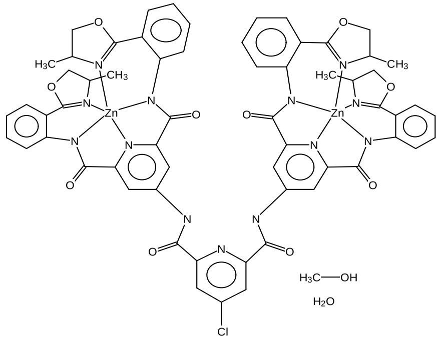

REBTOJ

Reference: A.J.Preston, J.C.Gallucci, J.R.Parquette (2006) *Org.Lett.* , **8,**5885

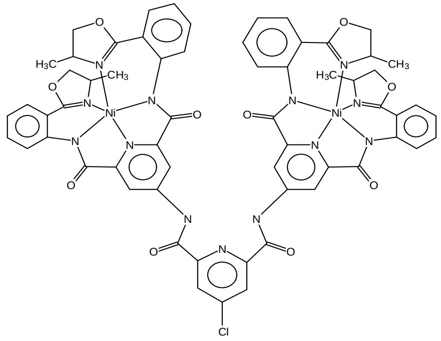

REKYOX

Reference: Xiaofeng Zhang, Deguang Huang, Yu-Sheng Chen, R.H.Holm (2012) *Inorg.Chem.* ,**51,**11017

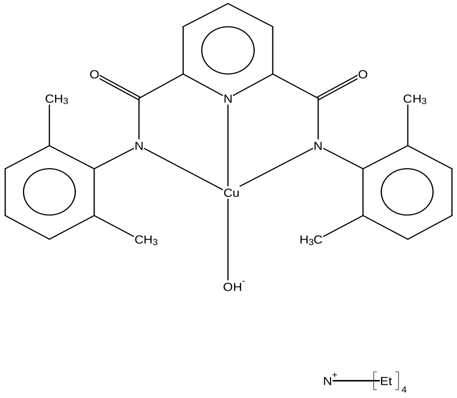

REKYUD

Reference: Xiaofeng Zhang, Deguang Huang, Yu-Sheng Chen, R.H.Holm (2012) *Inorg.Chem.* ,**51**,11017

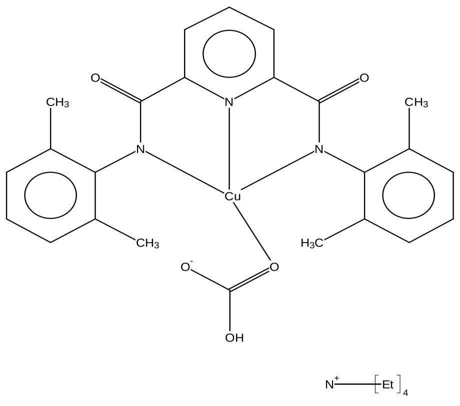

REKZEO

Reference: Xiaofeng Zhang, Deguang Huang, Yu-Sheng Chen, R.H.Holm (2012) *Inorg.Chem.* ,**51**,11017

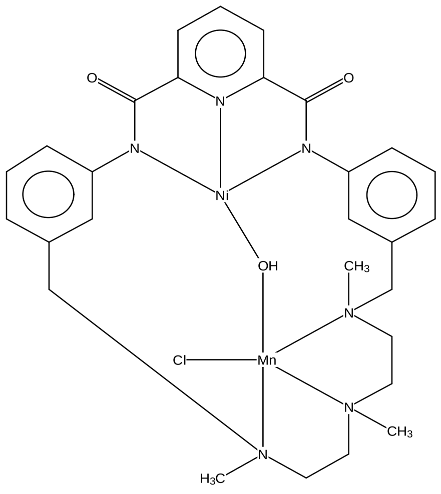

REKZIS

Reference: Xiaofeng Zhang, Deguang Huang, Yu-Sheng Chen, R.H.Holm (2012) *Inorg.Chem.* ,**51**,11017

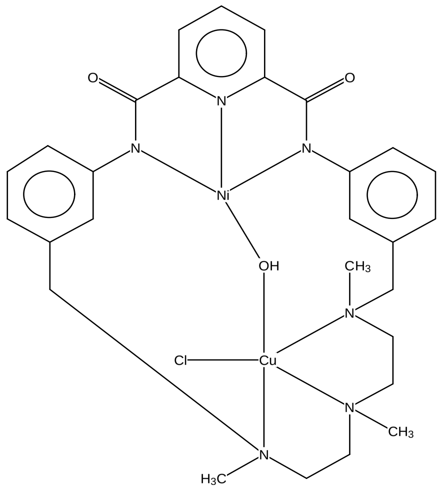

REKZOY

Reference: Xiaofeng Zhang, Deguang Huang, Yu-Sheng Chen, R.H.Holm (2012) *Inorg.Chem.* ,**51**,11017

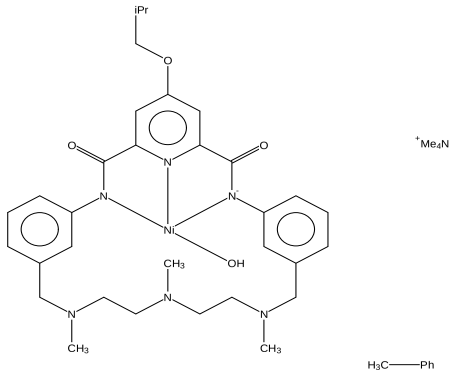

REKZUE

Reference: Xiaofeng Zhang, Deguang Huang, Yu-Sheng Chen, R.H.Holm (2012) *Inorg.Chem.* ,**51**,11017

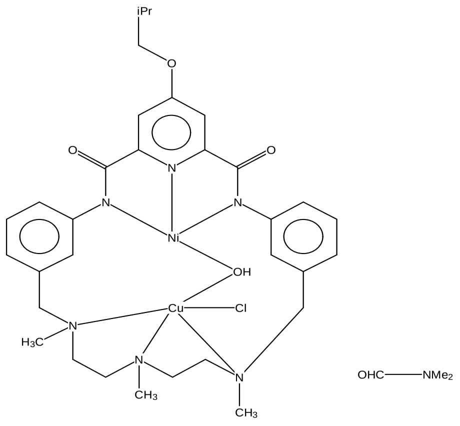

RELBAN

Reference: Xiaofeng Zhang, Deguang Huang, Yu-Sheng Chen, R.H.Holm (2012) *Inorg.Chem.* ,**51**,11017

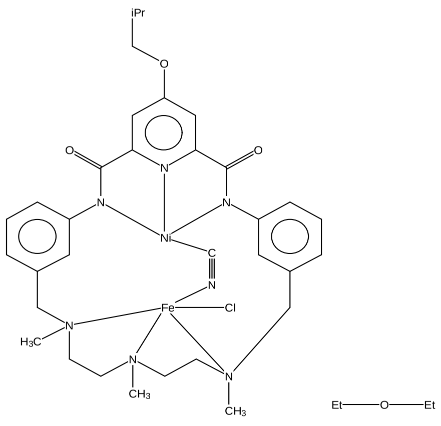

RELBER

Reference: Xiaofeng Zhang, Deguang Huang, Yu-Sheng Chen, R.H.Holm (2012) *Inorg.Chem.* ,**51**,11017

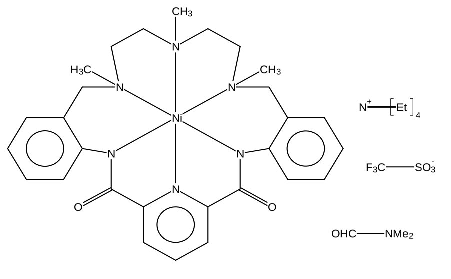

RELBIV

Reference: Xiaofeng Zhang, Deguang Huang, Yu-Sheng Chen, R.H.Holm (2012) *Inorg.Chem.* ,**51**,11017

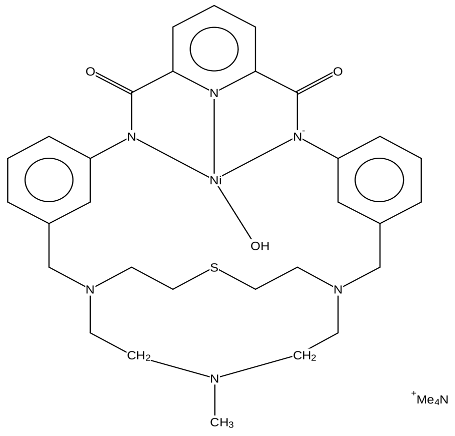

Search: search13 (Wed May 29 14:10:34 2024): Hits 401-408

RELBOB

Reference: Xiaofeng Zhang, Deguang Huang, Yu-Sheng Chen, R.H.Holm (2012) *Inorg.Chem.* ,**51,**11017

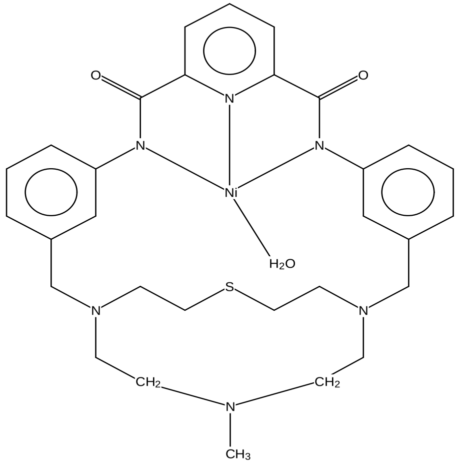

RELBUH

Reference: Xiaofeng Zhang, Deguang Huang, Yu-Sheng Chen, R.H.Holm (2012) *Inorg.Chem.* ,**51,**11017

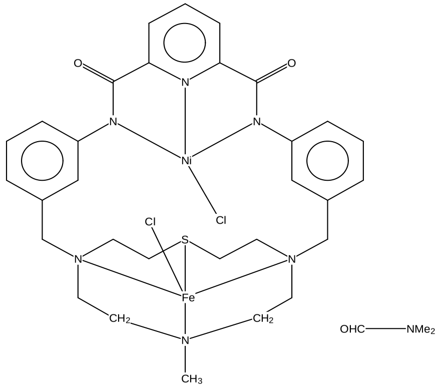

RELCAO

Reference: Xiaofeng Zhang, Deguang Huang, Yu-Sheng Chen, R.H.Holm (2012) *Inorg.Chem.* ,**51,**11017

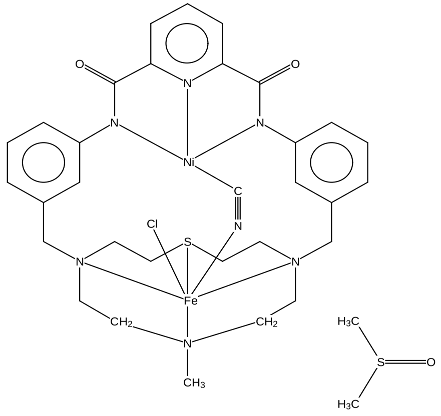

RELCES

Reference: Xiaofeng Zhang, Deguang Huang, Yu-Sheng Chen, R.H.Holm (2012) *Inorg.Chem.* ,**51,**11017

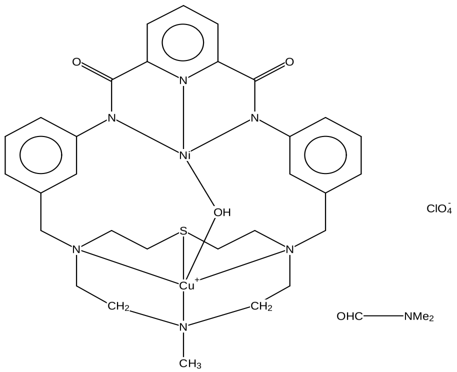

RELCIW

Reference: Xiaofeng Zhang, Deguang Huang, Yu-Sheng Chen, R.H.Holm (2012) *Inorg.Chem.* ,**51,**11017

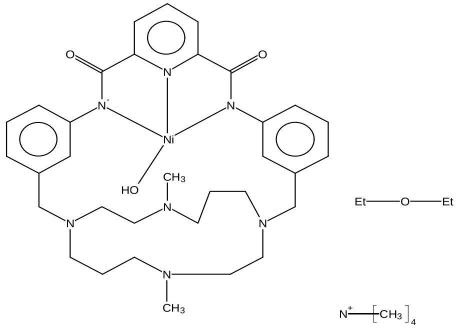

RELCOC

Reference: Xiaofeng Zhang, Deguang Huang, Yu-Sheng Chen, R.H.Holm (2012) *Inorg.Chem.* ,**51,**11017

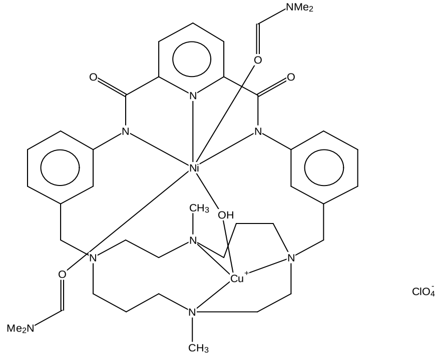

RELCUI

Reference: Xiaofeng Zhang, Deguang Huang, Yu-Sheng Chen, R.H.Holm (2012) *Inorg.Chem.* ,**51,**11017

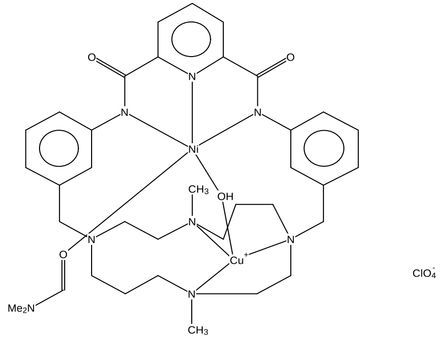

RELDAP

Reference: Xiaofeng Zhang, Deguang Huang, Yu-Sheng Chen, R.H.Holm (2012) *Inorg.Chem.* ,**51,**11017

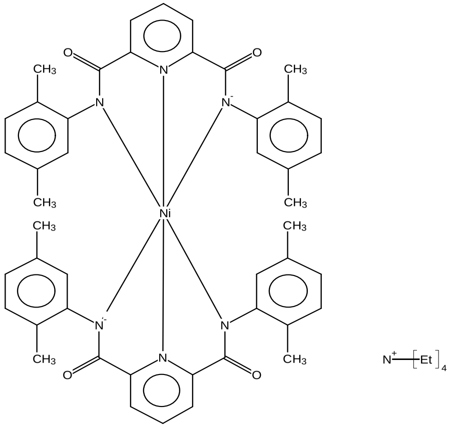

RELDET

Reference: Xiaofeng Zhang, Deguang Huang, Yu-Sheng Chen, R.H.Holm (2012) *Inorg.Chem.* ,**51,**11017

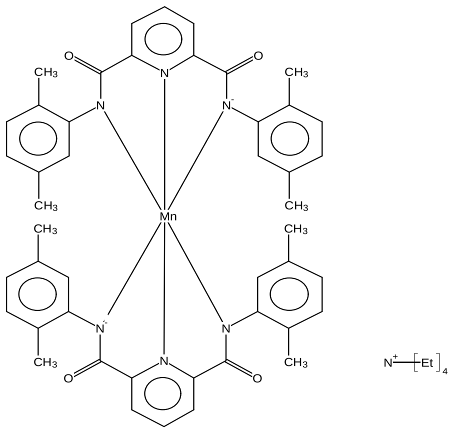

RELDIX

Reference: Xiaofeng Zhang, Deguang Huang, Yu-Sheng Chen, R.H.Holm (2012) *Inorg.Chem.* ,**51,**11017

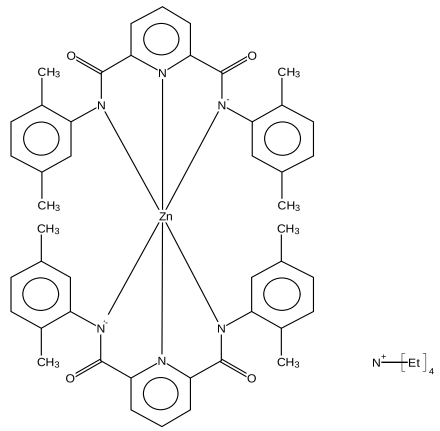

REVQEQ

Reference: Cai-Rong Wang, Jing-Lin Wang, Bin Liu, Bin-Sheng Yang (2013) *Wuji Huaxue Xuebao(Chin.)(Chin.J.Inorg.Chem.)* ,**29,**283

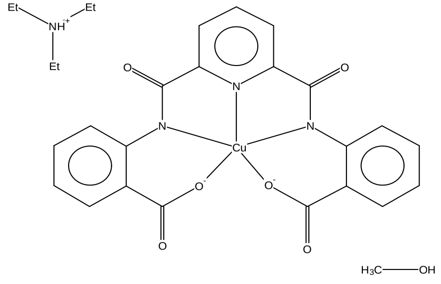

REVTOB

Reference: Xiaoyuan Chen, Shuzhong Zhan, Chuanjiang Hu, Qingjin Meng, Yongjiang Liu (1997) *J.Chem.Soc.,Dalton Trans.* ,245

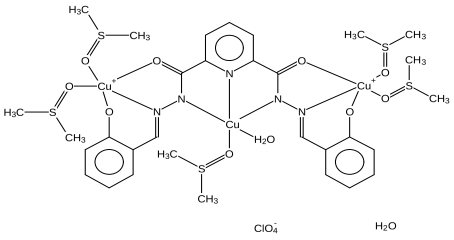

RITGUX

Reference: Quan-Fu Cao, Jian-Min Dou, Da-Cheng Li, Da-Qi Wang (2008) *Acta Crystallogr.,Sect.E:Struct.Rep.Online* ,**64,**m47

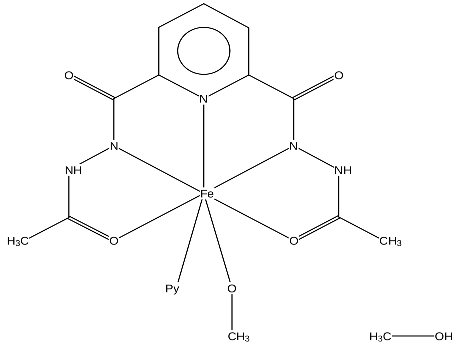

RIXJEN

Reference: S.M.Redmore, C.E.F.Rickard, S.J.Webb, L.J.Wright (1997) *Inorg.Chem.* ,**36,**4743

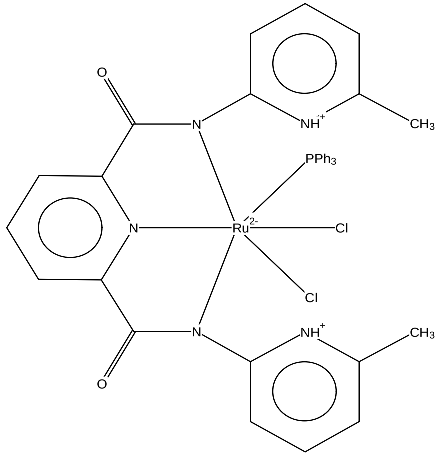

RIXJIR

Reference: S.M.Redmore, C.E.F.Rickard, S.J.Webb, L.J.Wright (1997) *Inorg.Chem.* ,**36,**4743

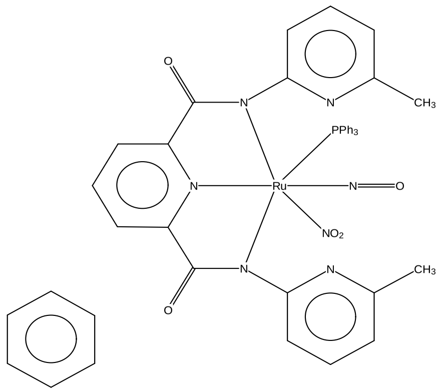

RONFEI

Reference: K.Ramakrishna, J.P.Biswas, Sadhan Jana, T.K.Achar, S.Porey, D.Maiti (2019) *Angew.Chem.,Int.Ed.* ,**58,**13808

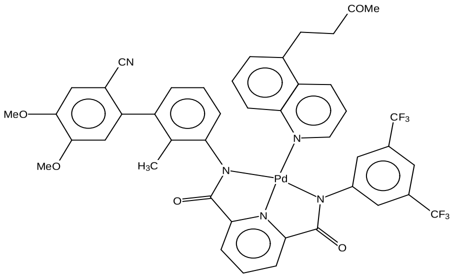

RONFIM

Reference: K.Ramakrishna, J.P.Biswas, Sadhan Jana, T.K.Achar, S.Porey, D.Maiti (2019) *Angew.Chem.,Int.Ed.* ,**58,**13808

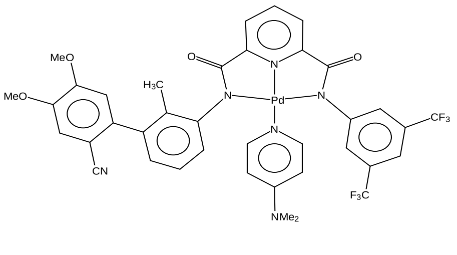

RONHUA

Reference: K.Ramakrishna, J.P.Biswas, Sadhan Jana, T.K.Achar, S.Porey, D.Maiti (2019) *Angew.Chem.,Int.Ed.* ,**58,**13808

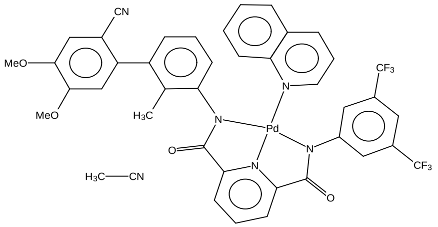

ROXXUZ

Reference: Debanjan Dhar, W.B.Tolman (2015) *J.Am.Chem.Soc.* ,**137,** 1322

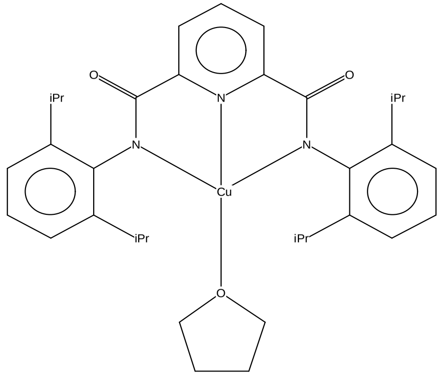

RUBMIM

Reference: Yanchuan Zhao, T.M.Swager (2015) *J.Am.Chem.Soc.* , **137,**3221

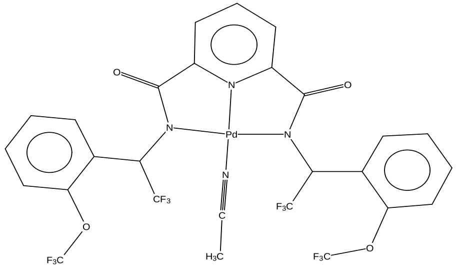

RUBMOS

Reference: Yanchuan Zhao, T.M.Swager (2015) *J.Am.Chem.Soc.* , **137,**3221

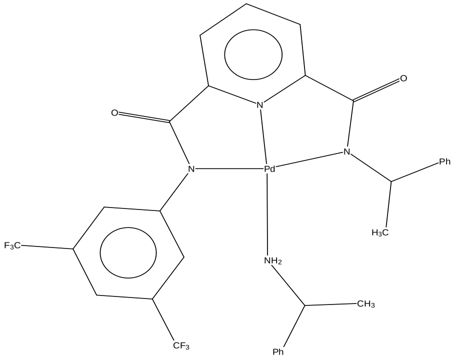

RUKHUD

Reference: M.Ballesteros II, Emily Y.Tsui (2020) *Dalton Trans.* ,**49,** 16305

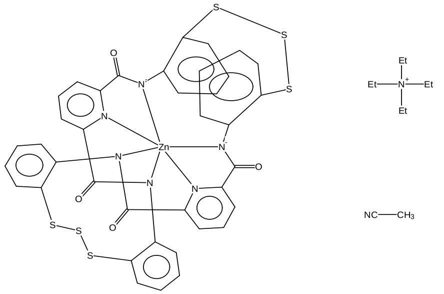

SEQZAR

Reference: M.R.Halvagar, B.Neisen, W.B.Tolman (2013) *Inorg.Chem.* ,**52,**793

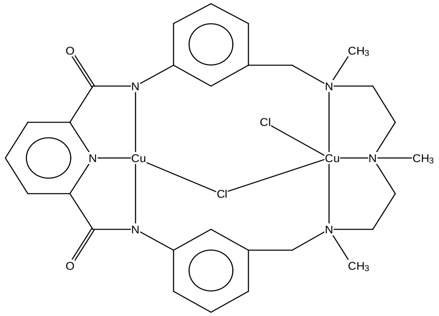

SEQZOF

Reference: M.R.Halvagar, B.Neisen, W.B.Tolman (2013) *Inorg.Chem.* ,**52,**793

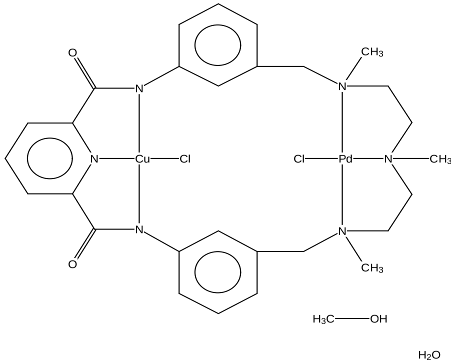

SEQZUL

Reference: M.R.Halvagar, B.Neisen, W.B.Tolman (2013) *Inorg.Chem.* ,**52,**793

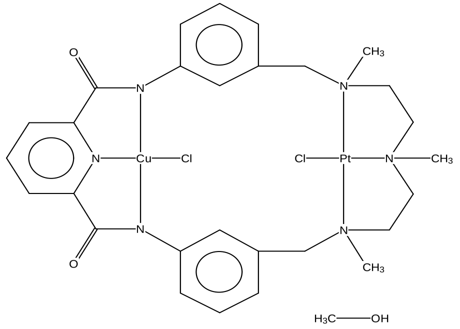

SIZTAY

Reference: M.G.Burgess, M.Naveed Zafar, S.T.Horner, G.R.Clark, L.James Wright (2014) *Dalton Trans.* ,**43,**17006

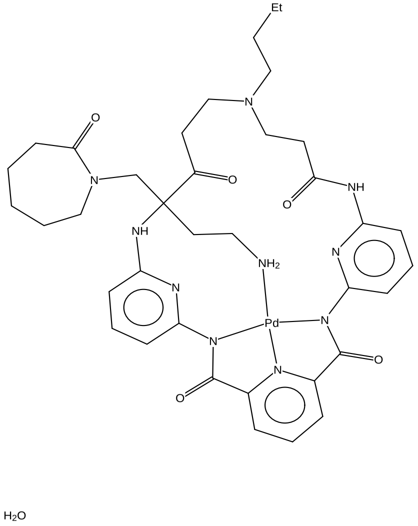

SIZTEC

Reference: M.G.Burgess, M.Naveed Zafar, S.T.Horner, G.R.Clark, L.James Wright (2014) *Dalton Trans.* ,**43,**17006

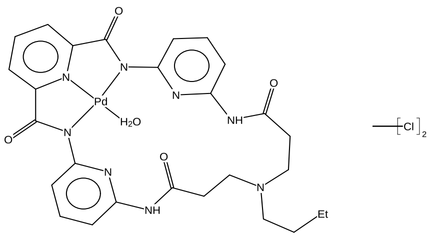

SOHNOT

Reference: M.J.Barrell, D.A.Leigh, P.J.Lusby, A.M.Z.Slawin (2008) *Angew.Chem.,Int.Ed.* ,**47,**8036

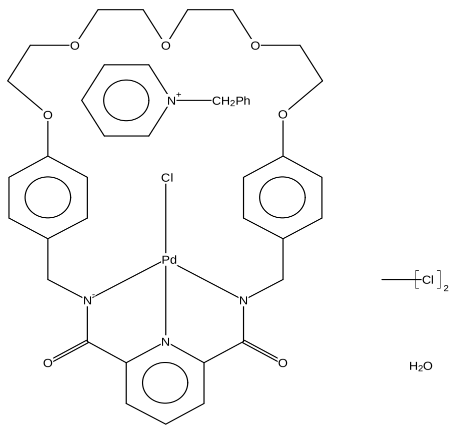

SOPCEH

Reference: M.W.Johnson, A.G.DiPasquale, R.G.Bergman, F.Dean Toste (2014) *Organometallics* ,**33,**4169

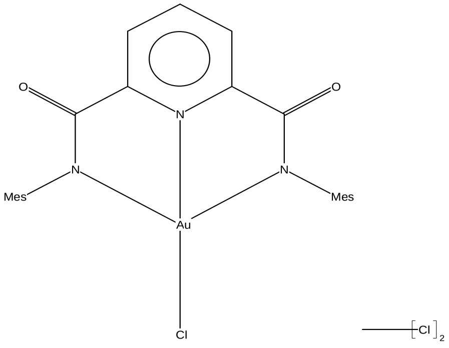

SURZUB

Reference: A.Mishra, A.Ali, S.Upreti, M.S.Whittingham, R.Gupta (2009) *Inorg.Chem.* ,**48,**5234

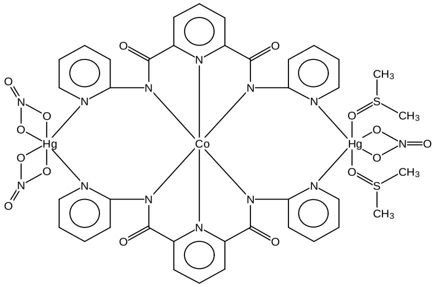

SUSBAK

Reference: A.Mishra, A.Ali, S.Upreti, M.S.Whittingham, R.Gupta (2009) *Inorg.Chem.* ,**48,**5234

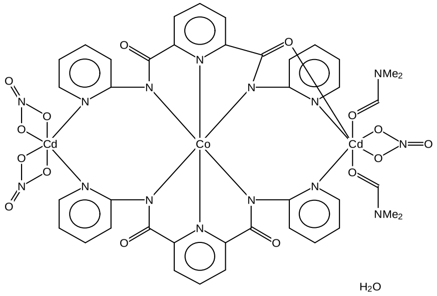

TAFRID

Reference: Linke Li, Hong Xu, Xianju Shi, Hongwei Hou, Yaoting Fan (2010) *Inorg.Chim.Acta* ,**363,**3939

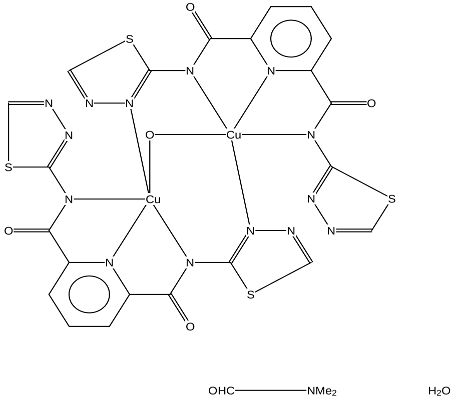

TAFROJ

Reference: Linke Li, Hong Xu, Xianju Shi, Hongwei Hou, Yaoting Fan (2010) *Inorg.Chim.Acta* ,**363,**3939

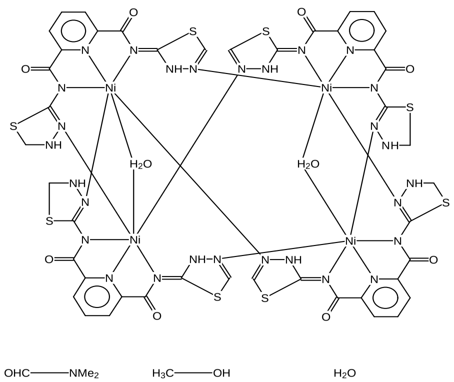

TAGYIL

Reference: R.Gupta, A.P.Singh (2010) *Eur.J.Inorg.Chem.* ,4546

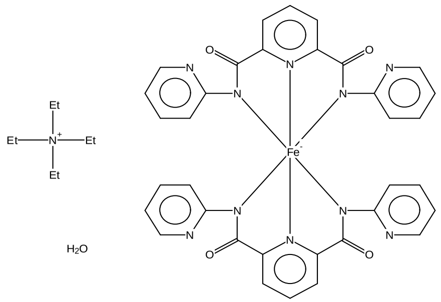

TAGYOR

Reference: R.Gupta, A.P.Singh (2010) *Eur.J.Inorg.Chem.* ,4546

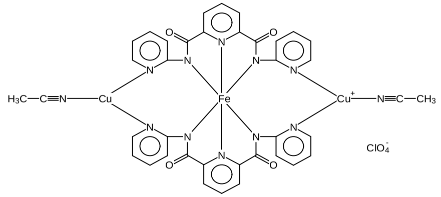

TAGYUX

Reference: R.Gupta, A.P.Singh (2010) *Eur.J.Inorg.Chem.* ,4546

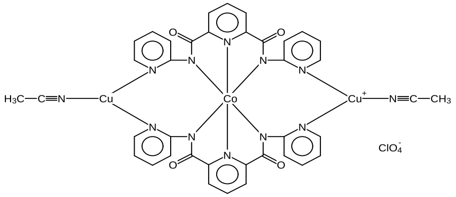

TAHBAI

Reference: Asif Noor, D.L.Maloney, J.E.M.Lewis, W.K.C.Lo, J.D.Crowley (2015) *Asian J.Org.Chem* ,**4**,208

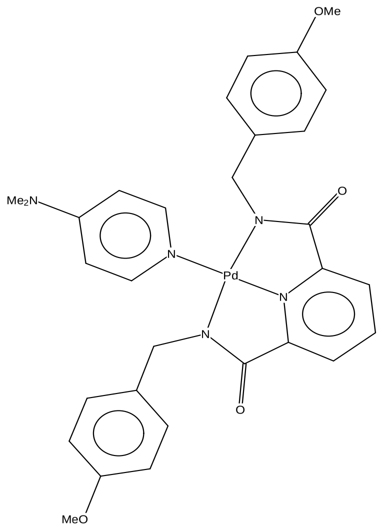

TAHBEM

Reference: Asif Noor, D.L.Maloney, J.E.M.Lewis, W.K.C.Lo, J.D.Crowley (2015) *Asian J.Org.Chem* ,**4**,208

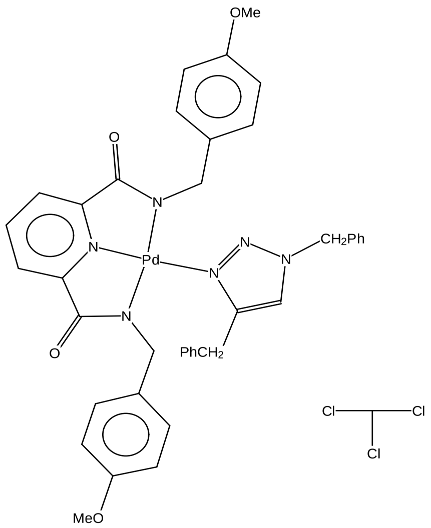

TAHBIQ

Reference: Asif Noor, D.L.Maloney, J.E.M.Lewis, W.K.C.Lo, J.D.Crowley (2015) *Asian J.Org.Chem* ,**4**,208

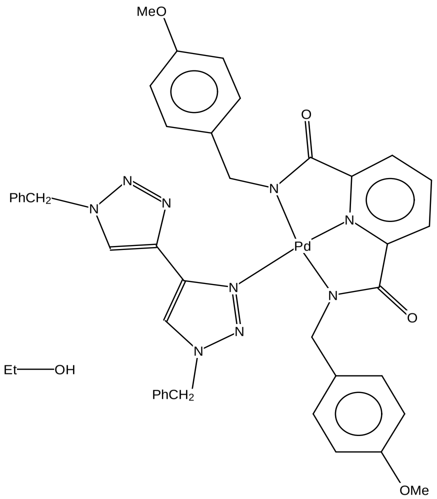

TAHBOW

Reference: Asif Noor, D.L.Maloney, J.E.M.Lewis, W.K.C.Lo, J.D.Crowley (2015) *Asian J.Org.Chem* ,**4**,208

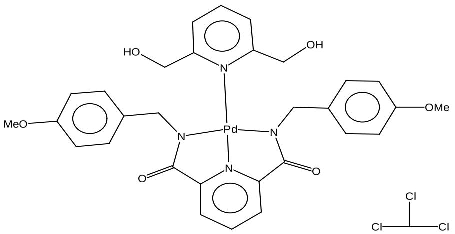

TASZET

Reference: K.Gudasi, R.Vadavi, R.Shenoy, M.Patil, S.A.Patil, M.Nethaji (2005) *Inorg.Chim.Acta* ,**358,**3799

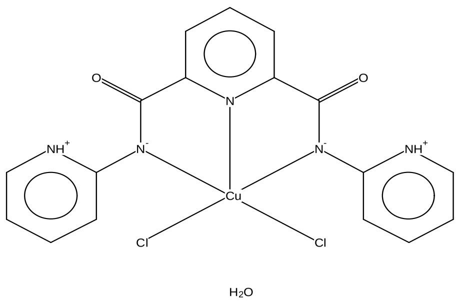

TATYAP

Reference: J.-C.Wasilke, Guang Wu, Xianhui Bu, G.Kehr, G.Erker (2005) *Organometallics* ,**24,**4289

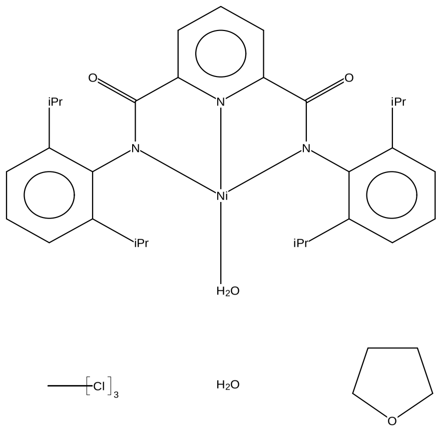

TEGVOT

Reference: B.D.Neisen, N.L.Gagnon, Debanjan Dhar, A.D.Spaeth, W.B.Tolman (2017) *J.Am.Chem.Soc.* ,**139,**10220

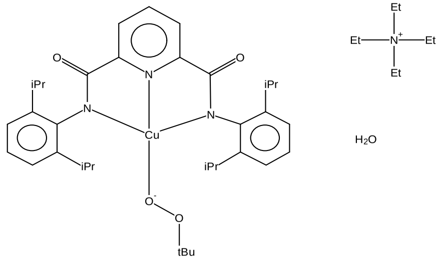

TEGVUZ

Reference: B.D.Neisen, N.L.Gagnon, Debanjan Dhar, A.D.Spaeth, W.B.Tolman (2017) *J.Am.Chem.Soc.* ,**139,**10220

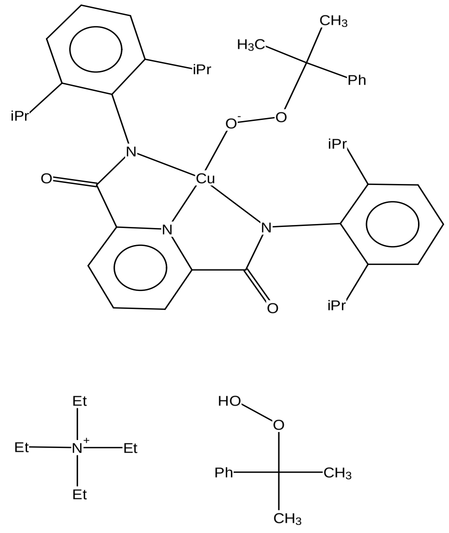

TEZNUK

Reference: M.E.Light (2018) *CSD Communication(Private Communication)* ,

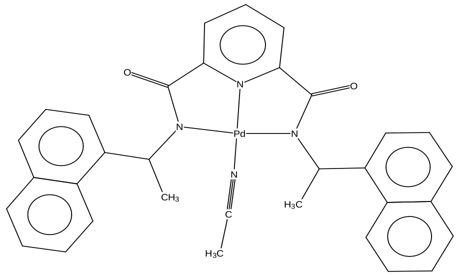

TIDBIS

Reference: I.V.Korendovych, O.P.Kryatova, W.M.Reiff, E.V.Rybak-Akimova (2007) *Inorg.Chem.* ,**46,**4197

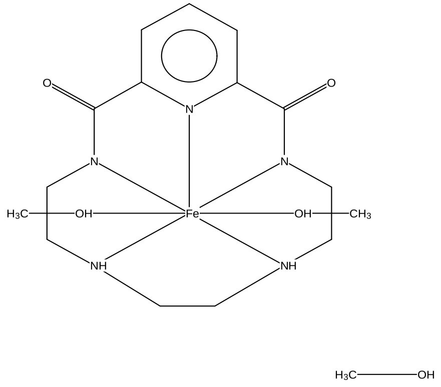

TIDBOY

Reference: I.V.Korendovych, O.P.Kryatova, W.M.Reiff, E.V.Rybak-Akimova (2007) *Inorg.Chem.* ,**46,**4197

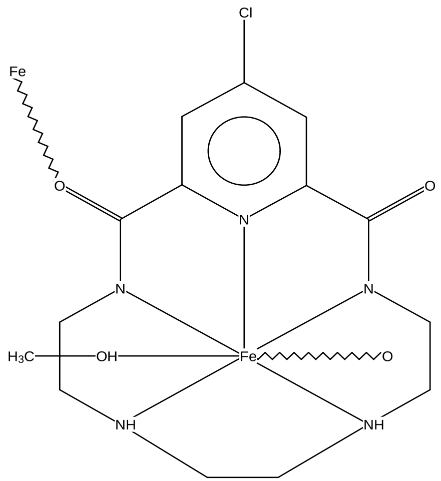

TIDBUE

Reference: I.V.Korendovych, O.P.Kryatova, W.M.Reiff, E.V.Rybak-Akimova (2007) *Inorg.Chem.* ,**46,**4197

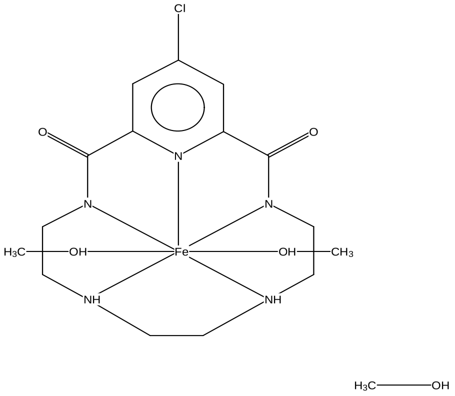

TIDCEP

Reference: I.V.Korendovych, O.P.Kryatova, W.M.Reiff, E.V.Rybak-Akimova (2007) *Inorg.Chem.* ,**46,**4197

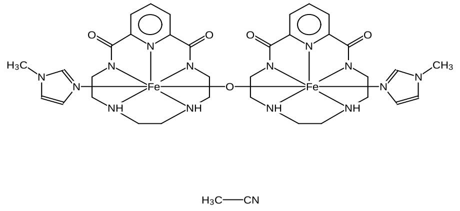

TIDCIT

Reference: I.V.Korendovych, O.P.Kryatova, W.M.Reiff, E.V.Rybak-Akimova (2007) *Inorg.Chem.* ,**46,**4197

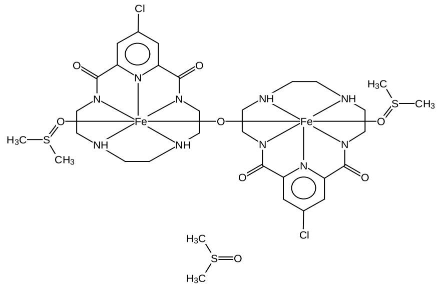

TIJMAD

Reference: Jianfeng Wu, Mei Guo, Xiao-Lei Li, Lang Zhao, Qing-Fu Sun, R.A.Layfield, Jinkui Tang (2018) *Chem.Commun.* ,**54,** 12097

Cl^-

Dy_4Cu_4--

--$[\mu_3\text{-}C_{21}H_{17}N_7O_4\text{-}\kappa^7N,N',N'',N''',N'''',N''''',N'''''',\kappa^4O,O',O'',O''']_4[C_{19}H_{15}N_7O_2]_4[HO]_4[Cl]_4{}^{4+}$

H_2O

TIMKOQ

Reference: P.D.Croucher, M.H.Klingele, A.Noble, S.Brooker (2007) *Dalton Trans.* ,4000

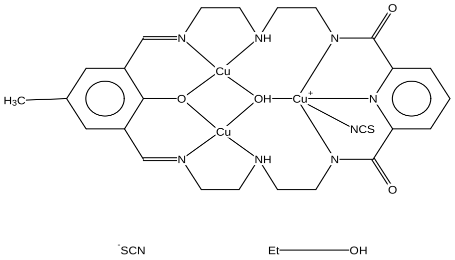

TINCAW

Reference: N.Zigon, N.Kyritsakas, M.W.Hosseini (2014) *Dalton Trans.* ,**43,**152

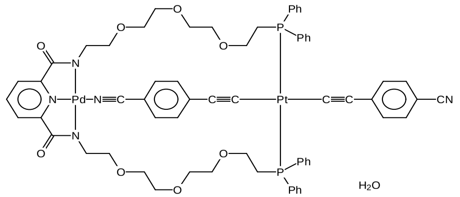

TOHNUB

Reference: Xiangyu Zhang, Yuhong Zhang, Shuzi Liu, Hong Xu, Jinpeng Li, Hongwei Hou (2014) *Inorg.Chem.Commun.* ,**46,**289

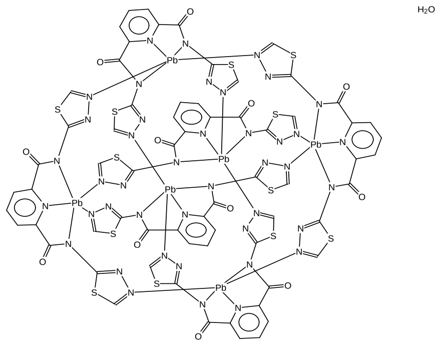

TOHPAJ

Reference: Xiangyu Zhang, Yuhong Zhang, Shuzi Liu, Hong Xu, Jinpeng Li, Hongwei Hou (2014) *Inorg.Chem.Commun.* ,**46,**289

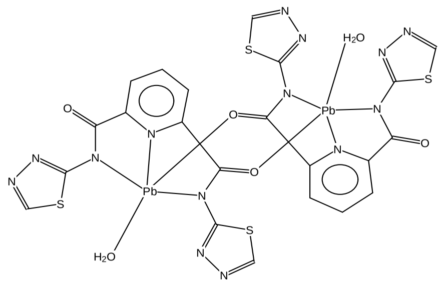

TOXKUM

Reference: F.A.Chavez, C.V.Nguyen, M.M.Olmstead, P.K.Mascharak (1996) *Inorg.Chem.* ,**35,**6282

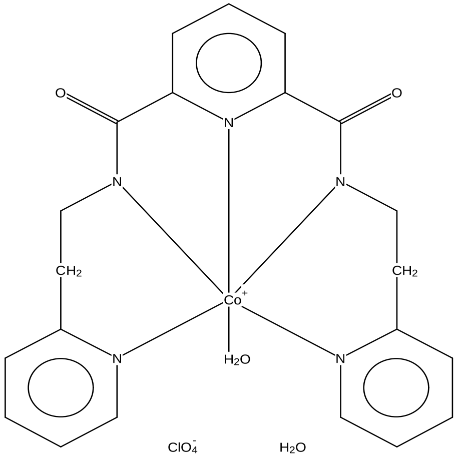

TOXLAT

Reference: F.A.Chavez, C.V.Nguyen, M.M.Olmstead, P.K.Mascharak (1996) *Inorg.Chem.* ,**35,**6282

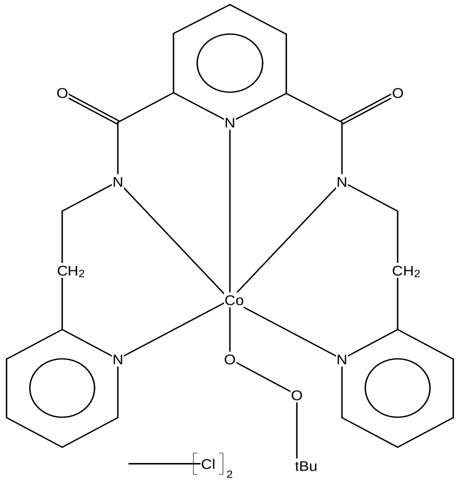

TOYCER

Reference: F.Czerny, P.Dohlert, M.Weidauer, E.Irran, S.Enthaler (2015) *Inorg.Chim.Acta* ,**425,**118

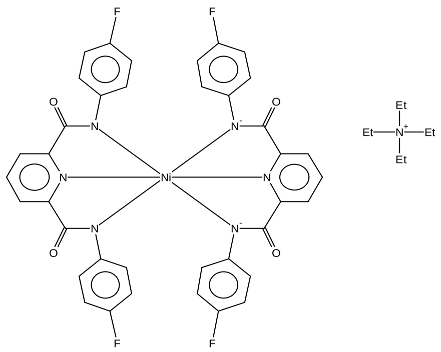

TOYQUV

Reference: F.Czerny, P.Dohlert, M.Weidauer, E.Irran, S.Enthaler (2015) *Inorg.Chim.Acta* ,**425,**118

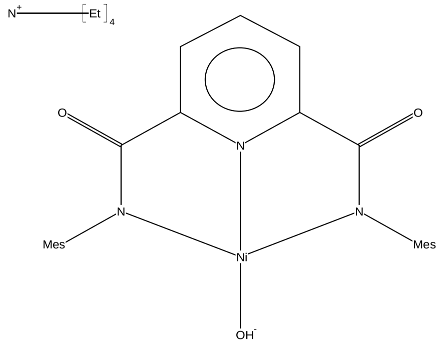

TOYRAC

Reference: F.Czerny, P.Dohlert, M.Weidauer, E.Irran, S.Enthaler (2015) *Inorg.Chim.Acta* ,**425,**118

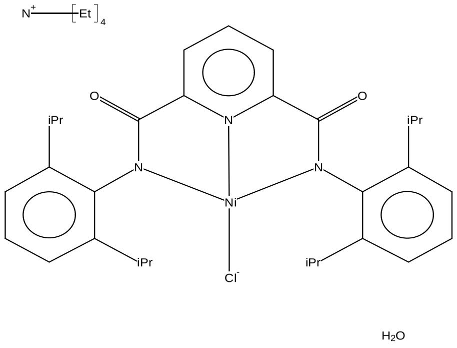

TOYREG

Reference: F.Czerny, P.Dohlert, M.Weidauer, E.Irran, S.Enthaler (2015) *Inorg.Chim.Acta* ,**425,**118

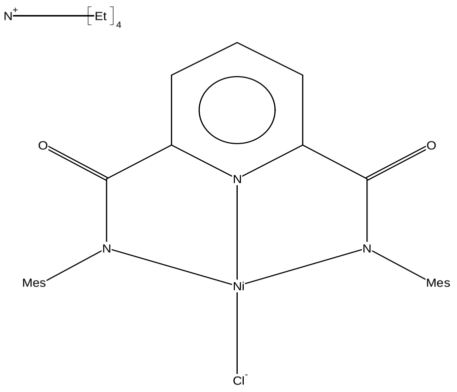

TOZFEW

Reference: M.Mandal, C.E.Elwell, C.J.Bouchey, T.J.Zerk, W.B.Tolman, C.J.Cramer (2019) *J.Am.Chem.Soc.* ,**141,**17236

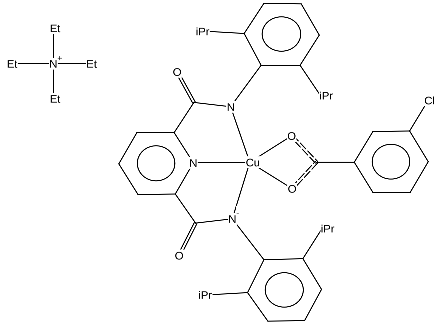

TOZZOX

Reference: R.W.Saalfrank, S.Trummer, H.Krautscheid, V.Schunemann, A.X.Trautwein, S.Hien, C.Stadler, J.Daub (1996) *Angew.Chem.,Int.Ed.* ,**35,**2206

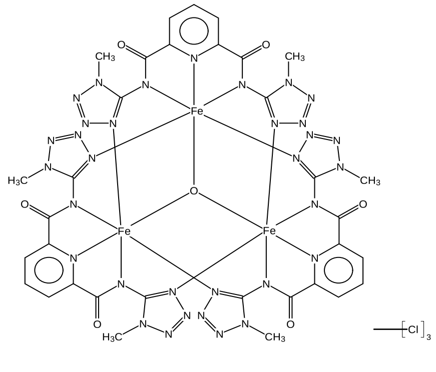

UCESUU

Reference: H.Sato, T.Tsukamoto, H.Sogawa, S.Kuwata, T.Takata (2021) *New J.Chem.* ,**45,**18282

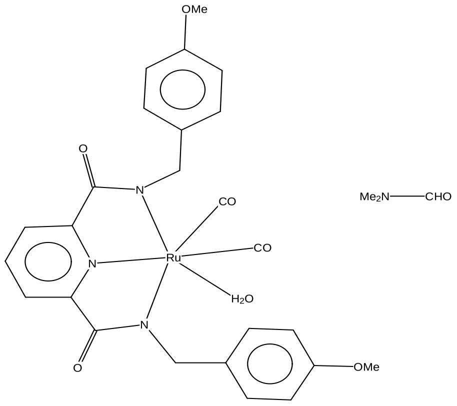

UCIBUH

Reference: H.Sato, T.Tsukamoto, H.Sogawa, S.Kuwata, T.Takata (2021) *New J.Chem.* ,**45,**18282

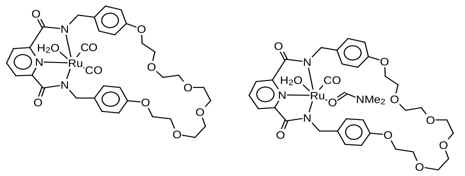

UCICAO

Reference: H.Sato, T.Tsukamoto, H.Sogawa, S.Kuwata, T.Takata (2021) *New J.Chem.* ,**45,**18282

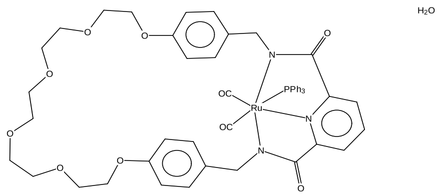

UCICES

Reference: H.Sato, T.Tsukamoto, H.Sogawa, S.Kuwata, T.Takata (2021) *New J.Chem.* ,**45,**18282

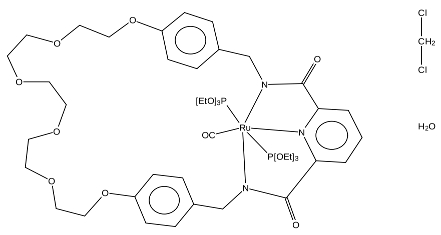

UDAWEB

Reference: C.Stadler, J.Daub, J.Kohler, R.W.Saalfrank, V.Coropceanu, V.Schunemann, C.Ober, A.X.Trautwein, S.F.Parker, M.Poyraz, T.Inomata, R.D.Cannon (2001) *J.Chem.Soc.,Dalton Trans.* , 3373

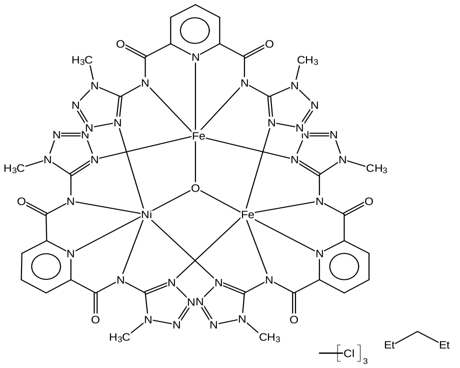

UDAWIF

Reference: C.Stadler, J.Daub, J.Kohler, R.W.Saalfrank, V.Coropceanu, V.Schunemann, C.Ober, A.X.Trautwein, S.F.Parker, M.Poyraz, T.Inomata, R.D.Cannon (2001) *J.Chem.Soc.,Dalton Trans.* , 3373

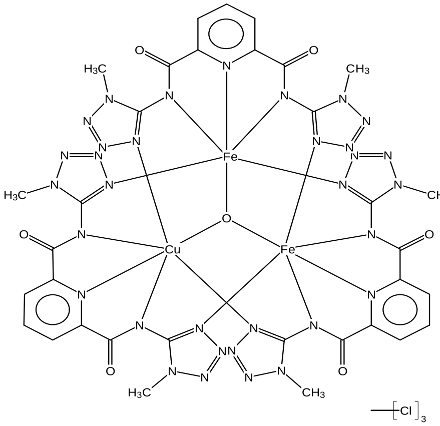

UFALAR

Reference: G.Kumar, S.Pandey, R.Gupta (2018) *Cryst.Growth Des.* , **18**,5501

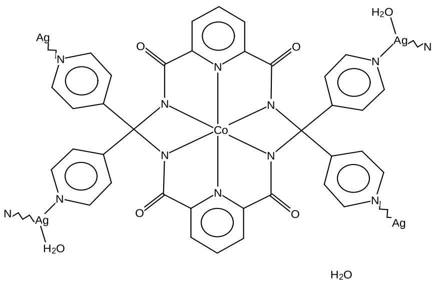

UFALEV

Reference: G.Kumar, S.Pandey, R.Gupta (2018) *Cryst.Growth Des.* , **18**,5501

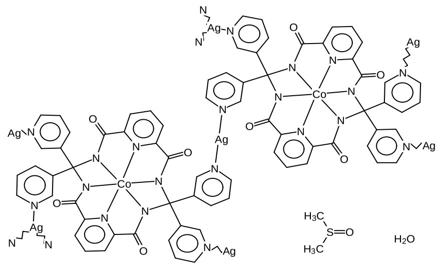

UFOBOI

Reference: Qi-Qiang Wang, V.W.Day, K.Bowman-James (2013) *Chem.Commun.* ,**49,**8042

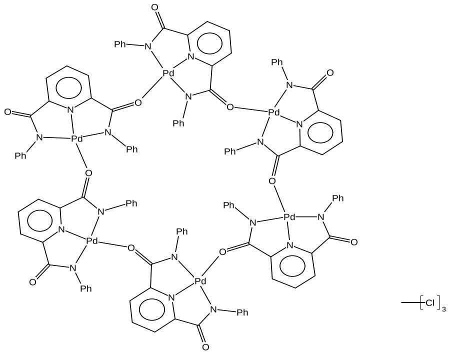

UGAKOF

Reference: Debanjan Dhar, G.M.Yee, W.B.Tolman (2018) *Inorg.Chem.* ,**57**,9794

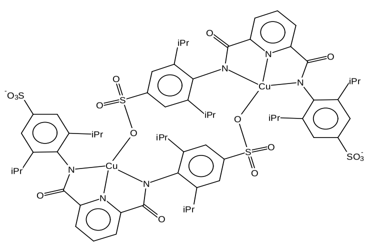

UGAKUL

Reference: Debanjan Dhar, G.M.Yee, W.B.Tolman (2018) *Inorg.Chem.* ,**57**,9794

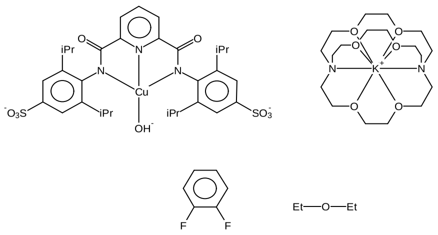

UGALEW

Reference: Debanjan Dhar, G.M.Yee, W.B.Tolman (2018) *Inorg.Chem.* ,**57**,9794

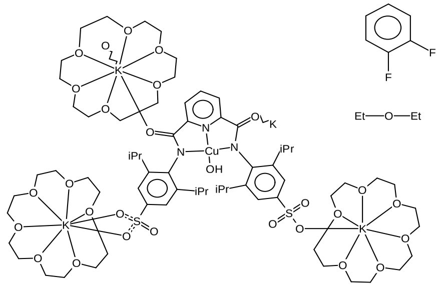

UGALIA

Reference: Debanjan Dhar, G.M.Yee, W.B.Tolman (2018) *Inorg.Chem.* ,**57**,9794

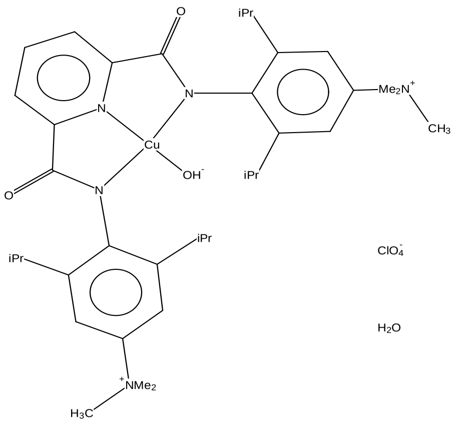

UGALOG

Reference: Debanjan Dhar, G.M.Yee, W.B.Tolman (2018) *Inorg.Chem.* ,**57**,9794

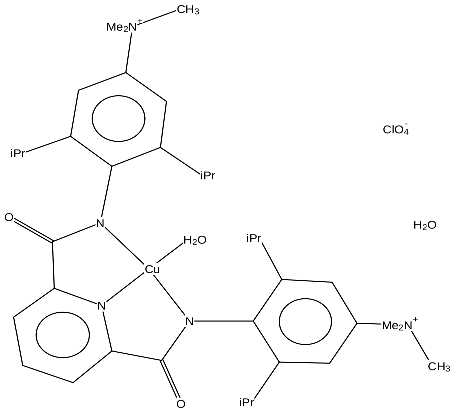

UHEPAZ

Reference: Gao-Feng Liu, K.Durr, R.Puchta, F.W.Heinemann, R.van Eldik, I.Ivanovic-Burmazovic (2009) *Dalton Trans.* ,6292

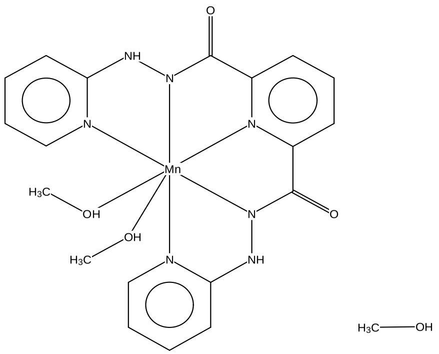

ULIWUK

Reference: D.Prabha, S.Pachisia, R.Gupta (2021) *Inorg.Chem.Front.* , **8**,1599

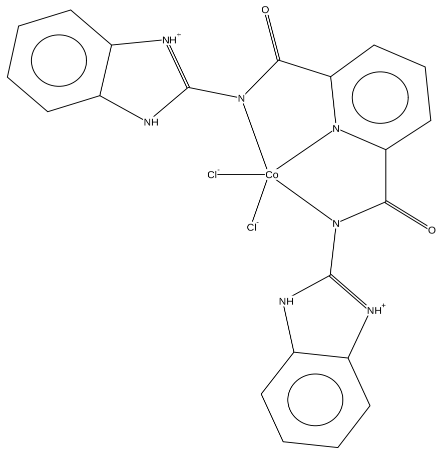

ULIXAR

Reference: D.Prabha, S.Pachisia, R.Gupta (2021) *Inorg.Chem.Front.* , **8**,1599

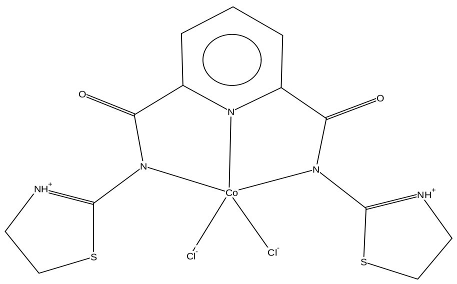

ULIXEV

Reference: D.Prabha, S.Pachisia, R.Gupta (2021) *Inorg.Chem.Front.* , **8**,1599

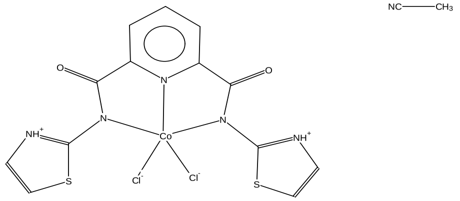

VAMHUO

Reference: Suni Qin, Chuanli Nie, Zhehui Weng, Zilu Chen, Fupei Liang (2011) *Z.Anorg.Allg.Chem.* ,**637**,2294

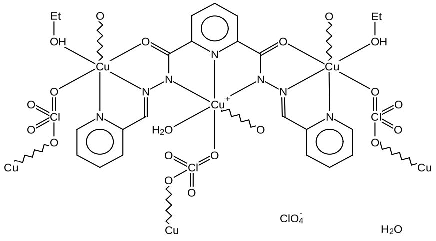

VAMJAW

Reference: Suni Qin, Chuanli Nie, Zhehui Weng, Zilu Chen, Fupei Liang (2011) *Z.Anorg.Allg.Chem.* ,**637**,2294

Cu_6--
--[μ_3-$C_{19}H_{13}N_7O_2$-κ^7N,N',N'',N''',N'''',N''''',N'''''',κ^1O]$_2$[μ_3-HO]$_2$[μ_2-CO_2Me-κ^2O,O']$_2$[OCOMe]$_4$

H_2O

VAMJEA

Reference: Suni Qin, Chuanli Nie, Zhehui Weng, Zilu Chen, Fupei Liang (2011) *Z.Anorg.Allg.Chem.* ,**637**,2294

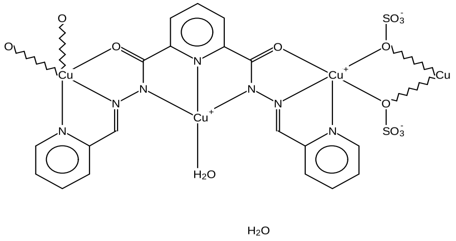

VECLEU

Reference: E.C.Constable, M.J.Doyle, S.M.Elder, P.R.Raithby (1989) *J.Chem.Soc.Chem.Comm.* ,1376

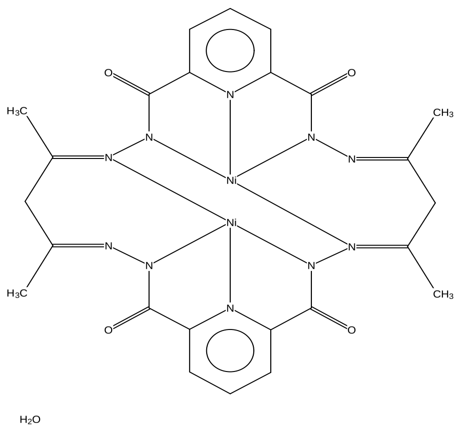

VIFLUU

Reference: P.Pirovano, B.Twamley, A.R.McDonald (2018) *Chem.-Eur.J.* ,**24**,5238

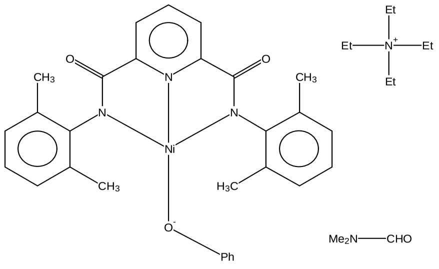

VIFMAB

Reference: P.Pirovano, B.Twamley, A.R.McDonald (2018) *Chem.-Eur.J.* ,**24**,5238

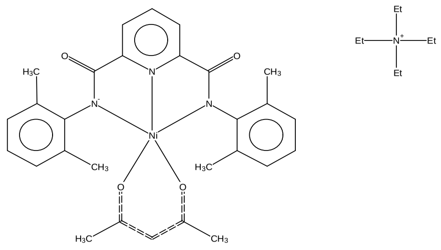

VIFMEF

Reference: P.Pirovano, B.Twamley, A.R.McDonald (2018) *Chem.-Eur.J.* ,**24**,5238

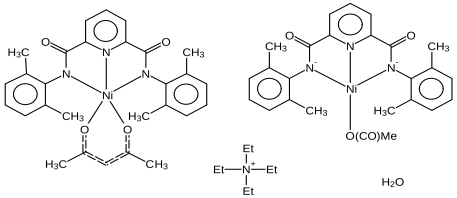

VIFMIJ

Reference: P.Pirovano, B.Twamley, A.R.McDonald (2018) *Chem.-Eur.J.* ,**24**,5238

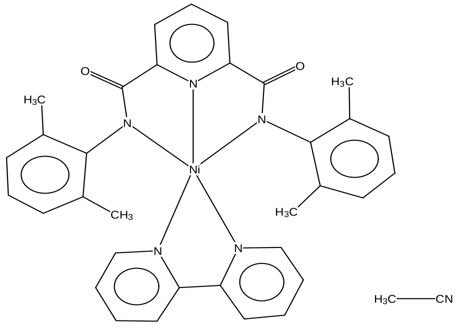

VIFMOP

Reference: P.Pirovano, B.Twamley, A.R.McDonald (2018) *Chem.-Eur.J.* ,**24**,5238

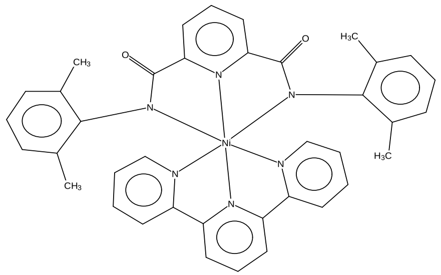

VIFMUV

Reference: P.Pirovano, B.Twamley, A.R.McDonald (2018) *Chem.-Eur.J.* ,**24**,5238

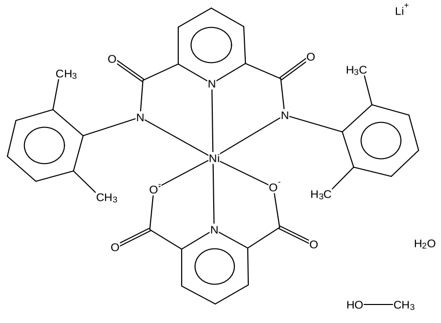

VIVLOE

Reference: P.Jerome, S.Y.Arafath, J.Haribabu, N.S.P.Bhuvanesh, R.Karvembu (2019) *Chem. Sel.* ,**4**,2237

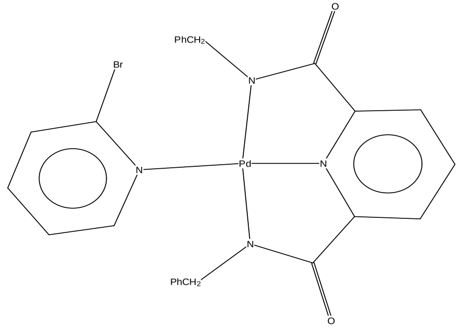

VOFKEJ

Reference: C.McManus, P.Mondal, M.Lovisari, B.Twamley, A.R.McDonald (2019) *Inorg.Chem.* ,**58**,4515

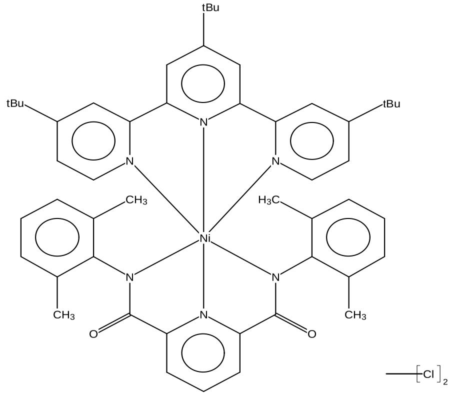

VOLPIX

Reference: M.R.Halvagar, P.V.Solntsev, Hyeongtaek Lim, B.Hedman, K.O.Hodgson, E.I.Solomon, C.J.Cramer, W.B.Tolman (2014) *J.Am.Chem.Soc.* ,**136**,7269

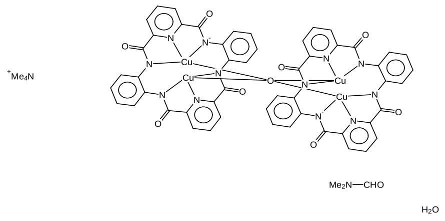

VOLPOD

Reference: M.R.Halvagar, P.V.Solntsev, Hyeongtaek Lim, B.Hedman, K.O.Hodgson, E.I.Solomon, C.J.Cramer, W.B.Tolman (2014) *J.Am.Chem.Soc.* ,**136**,7269

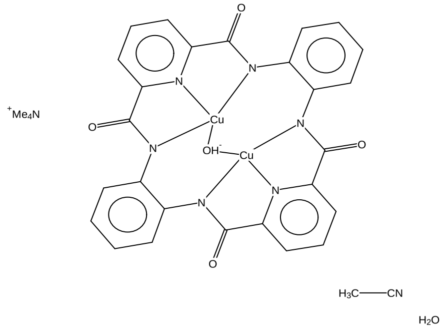

VOLPUJ

Reference: M.R.Halvagar, P.V.Solntsev, Hyeongtaek Lim, B.Hedman, K.O.Hodgson, E.I.Solomon, C.J.Cramer, W.B.Tolman (2014) *J.Am.Chem.Soc.* ,**136**,7269

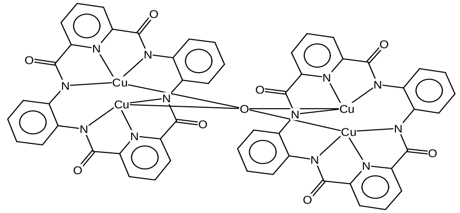

VULDEO

Reference: J.K.Bower, A.D.Cypcar, B.Henriquez, S.Chantal E.Stieber, Shiyu Zhang (2020) *J.Am.Chem.Soc.* ,**142,**8514

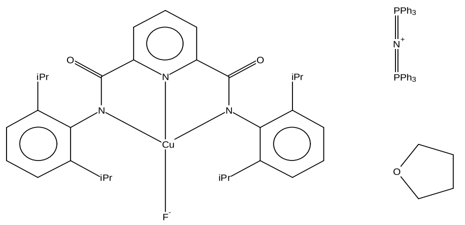

VULDIS

Reference: J.K.Bower, A.D.Cypcar, B.Henriquez, S.Chantal E.Stieber, Shiyu Zhang (2020) *J.Am.Chem.Soc.* ,**142,**8514

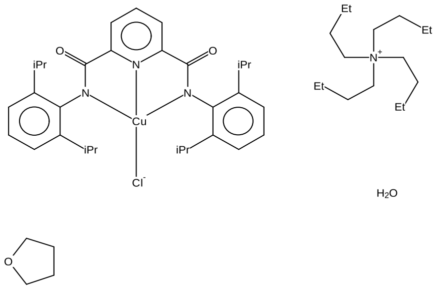

VULDOY

Reference: J.K.Bower, A.D.Cypcar, B.Henriquez, S.Chantal E.Stieber, Shiyu Zhang (2020) *J.Am.Chem.Soc.* ,**142,**8514

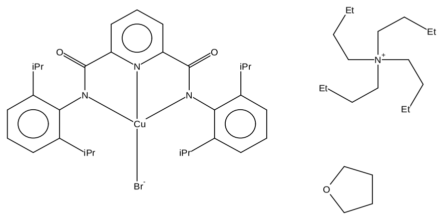

VULDUE

Reference: J.K.Bower, A.D.Cypcar, B.Henriquez, S.Chantal E.Stieber, Shiyu Zhang (2020) *J.Am.Chem.Soc.* ,**142,**8514

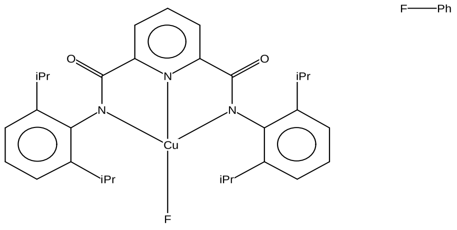

VULFAM

Reference: J.K.Bower, A.D.Cypcar, B.Henriquez, S.Chantal E.Stieber, Shiyu Zhang (2020) *J.Am.Chem.Soc.* ,**142,**8514

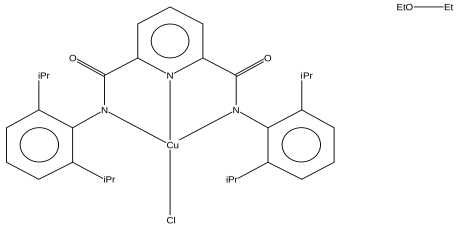

VULFEQ

Reference: J.K.Bower, A.D.Cypcar, B.Henriquez, S.Chantal E.Stieber, Shiyu Zhang (2020) *J.Am.Chem.Soc.* ,**142,**8514

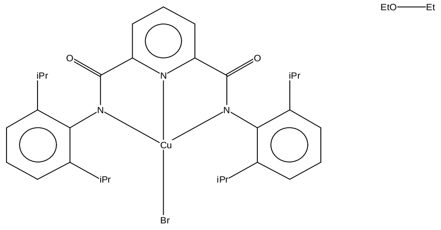

WAFWAF

Reference: D.G.A.Verhoeven, M.Albrecht (2020) *Dalton Trans.* ,**49,** 17674

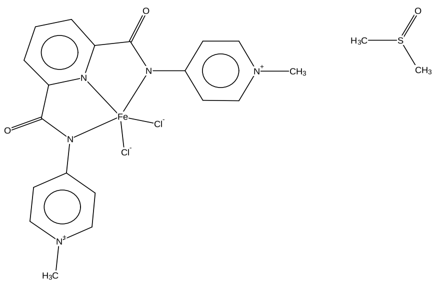

WAGVIM

Reference: G.Kumar, G.Kumar, R.Gupta (2016) *RSC Advances* ,**6,** 21352

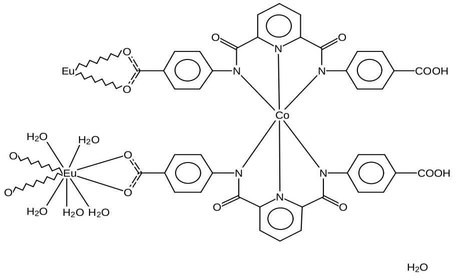

WAGVOS

Reference: G.Kumar, G.Kumar, R.Gupta (2016) *RSC Advances* ,**6**, 21352

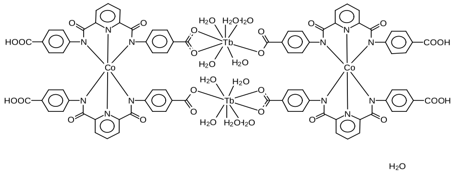

WAHNEA

Reference: A.P.Singh, A.Ali, R.Gupta (2010) *Dalton Trans.* ,**39**,8135

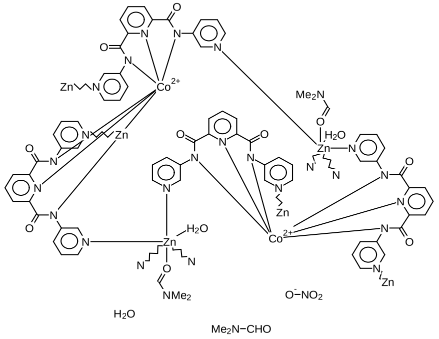

WAHNIE

Reference: A.P.Singh, A.Ali, R.Gupta (2010) *Dalton Trans.* ,**39**,8135

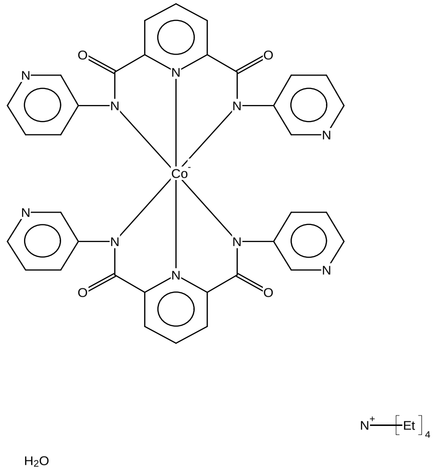

WAHNOK

Reference: A.P.Singh, A.Ali, R.Gupta (2010) *Dalton Trans.* ,**39**,8135

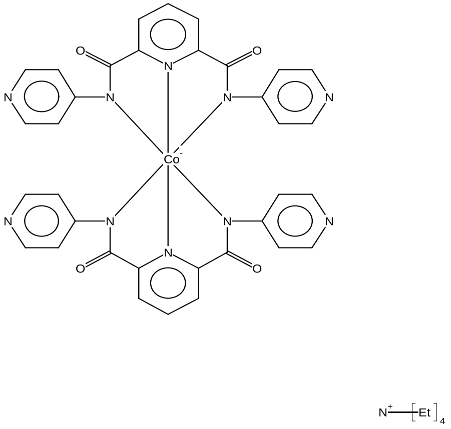

WAHNUQ

Reference: A.P.Singh, A.Ali, R.Gupta (2010) *Dalton Trans.* ,**39**,8135

n-Zn_5Co_6--
--$[\mu_3\text{-}C_{17}H_{11}N_5O_2\text{-}\kappa^5N,N',N'',N''',N''''']_6[\mu_2\text{-}C_{17}H_{11}N_5O_2\text{-}\kappa^4N,N',N'',N''']_6[H_2O]_6{}^{6+}$

O^-—NO_2

H_2O

WALDOG

Reference: P.Jerome, J.Haribabu, N.S.P.Bhuvanesh, R.Karvembu (2020) *Chem. Sel.* ,**5**,13591

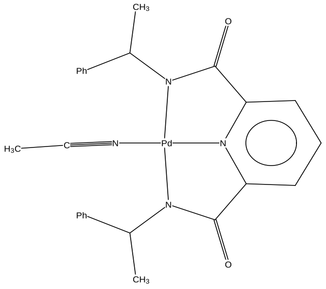

H_2O

WALDUM

Reference: P.Jerome, J.Haribabu, N.S.P.Bhuvanesh, R.Karvembu (2020) *Chem. Sel.* ,**5**,13591

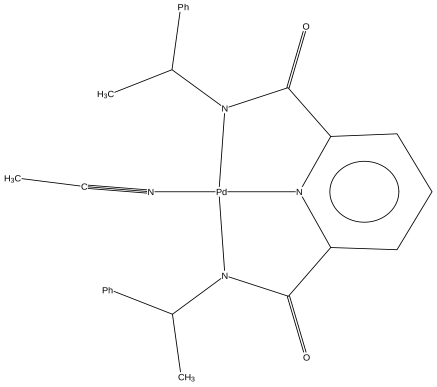

WALFAU

Reference: P.Jerome, J.Haribabu, N.S.P.Bhuvanesh, R.Karvembu (2020) *Chem. Sel.* ,**5**,13591

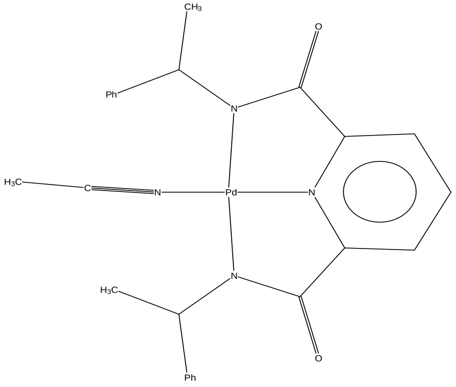

Search: search13 (Wed May 29 14:10:34 2024): Hits 513-520

WANZEQ

Reference: D.S.Marlin, M.M.Olmstead, P.K.Mascharak (2000) *Inorg.Chim.Acta* ,**297,**106

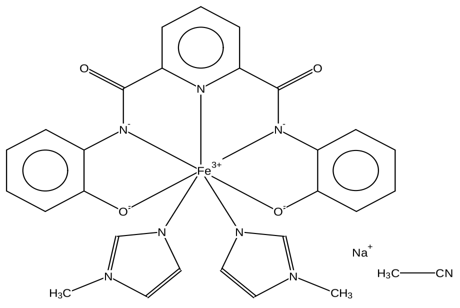

WAPBAQ

Reference: D.S.Marlin, M.M.Olmstead, P.K.Mascharak (2000) *Inorg.Chim.Acta* ,**297,**106

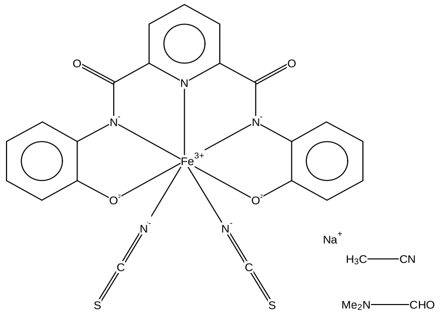

WEWTUQ

Reference: S.Pandey, D.Bansal, R.Gupta (2018) *New J.Chem.* ,**42,** 9847

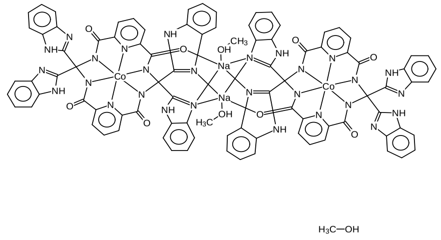

WEWYAB

Reference: S.Pandey, D.Bansal, R.Gupta (2018) *New J.Chem.* ,**42,** 9847

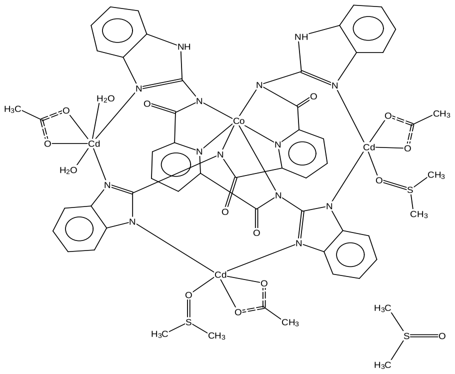

WEWYEF

Reference: S.Pandey, D.Bansal, R.Gupta (2018) *New J.Chem.* ,**42,** 9847

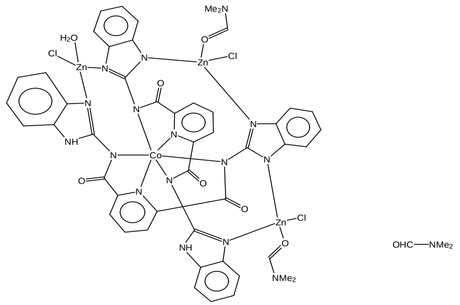

WIFYER

Reference: N.Zigon, A.Guenet, E.Graf, N.Kyritsakas, M.W.Hosseini (2013) *Dalton Trans.* ,**42,**9740

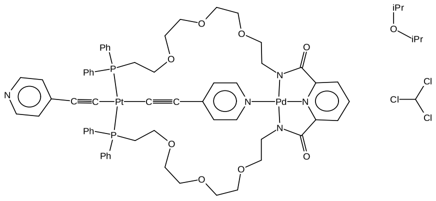

WIJZIA

Reference: E.Gumienna-Kontecka, I.A.Golenya, A.Szebesczyk, M.Haukka, R.Kramer, I.O.Fritsky (2013) *Inorg.Chem.* ,**52,**7633

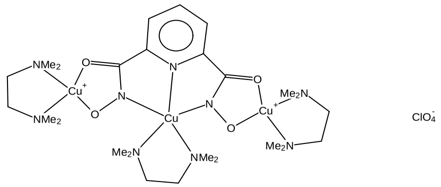

WOFCAX

Reference: S.Srivastava, A.Ali, A.Tyagi, R.Gupta (2014) *Eur.J.Inorg.Chem.* ,2113

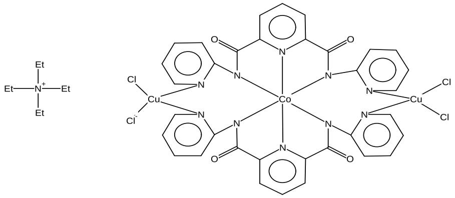

WOHSIV

Reference: A.K.Patra, M.Ray, R.Mukherjee (2000) *Polyhedron* ,**19,** 1423

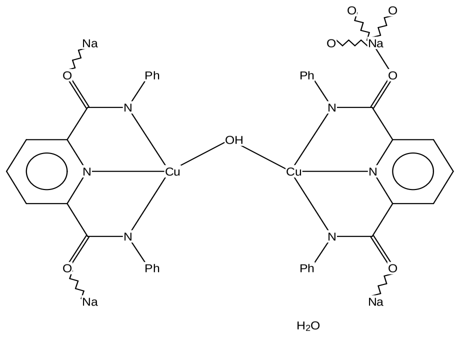

WOHSOB

Reference: A.K.Patra, M.Ray, R.Mukherjee (2000) *Polyhedron* ,**19,** 1423

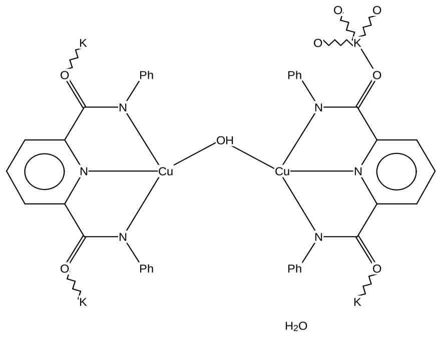

WOKFIO

Reference: F.Schon, F.Biebl, Lutz Greb, Simone Leingang, B.Grimm-Lebsanft, M.Teubner, S.Buchenau, E.Kaifer, M.A.Rubhausen, H.-J.Himmel (2019) *Chem.-Eur.J.* ,

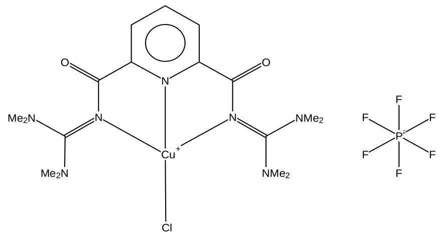

WOLZOP

Reference: T.K.Achar, J.P.Biswas, S.Porey, T.Pal, K.Ramakrishna, S.Maiti, D.Maiti (2019) *J.Org.Chem.* ,**84,**8315

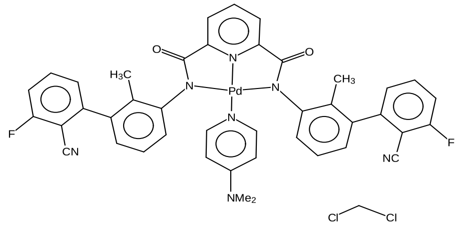

WOMCIN

Reference: T.K.Achar, J.P.Biswas, S.Porey, T.Pal, K.Ramakrishna, S.Maiti, D.Maiti (2019) *J.Org.Chem.* ,**84,**8315

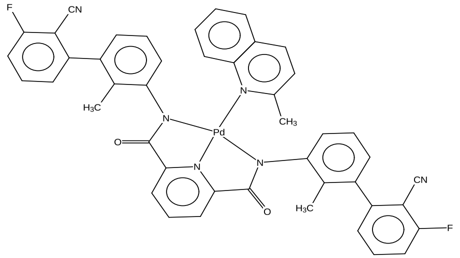

WUQLOK

Reference: T.Lang, A.Guenet, E.Graf, N.Kyritsakas, M.W.Hosseini (2010) *Chem.Commun.* ,**46,**3508

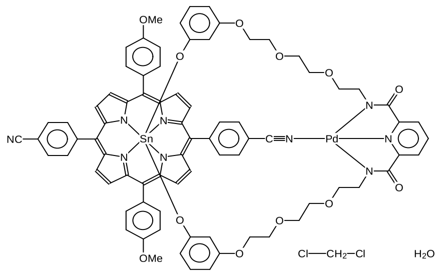

WUXFEC

Reference: D.Bansal, S.Pandey, G.Hundal, R.Gupta (2015) *New J.Chem.* ,**39,**9772

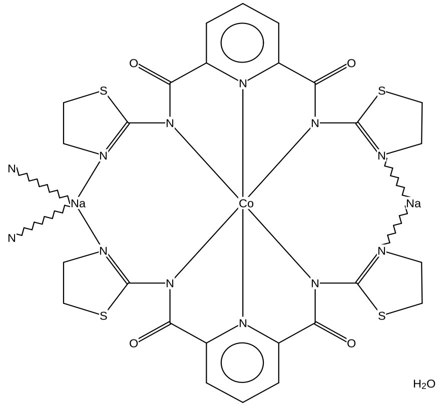

WUXFIG

Reference: D.Bansal, S.Pandey, G.Hundal, R.Gupta (2015) *New J.Chem.* ,**39,**9772

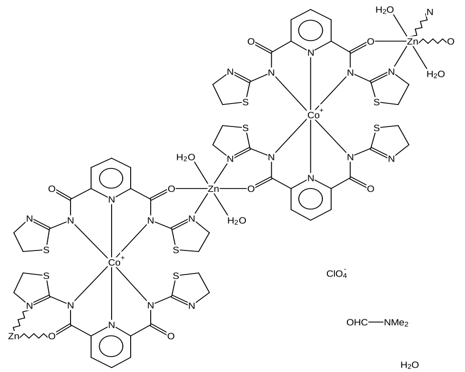

WUXHUU

Reference: D.Bansal, S.Pandey, G.Hundal, R.Gupta (2015) *New J.Chem.* ,**39,**9772

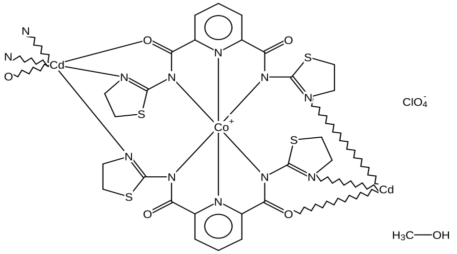

WUXJAC

Reference: D.Bansal, S.Pandey, G.Hundal, R.Gupta (2015) *New J.Chem.* ,**39,**9772

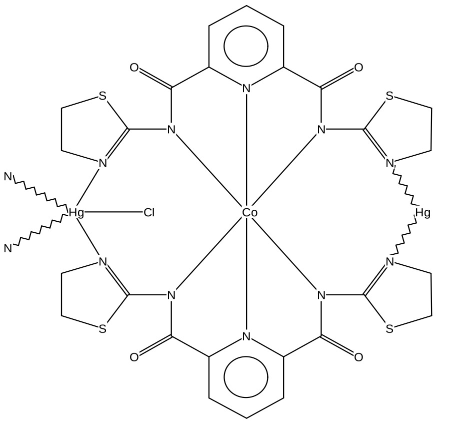

WUYDIE

Reference: Xiao-Qing Shen, Zi-Feng Li, Hong-Yun Zhang, Zhong-Jun Li (2010) *J.Coord.Chem.* ,**63,**1720

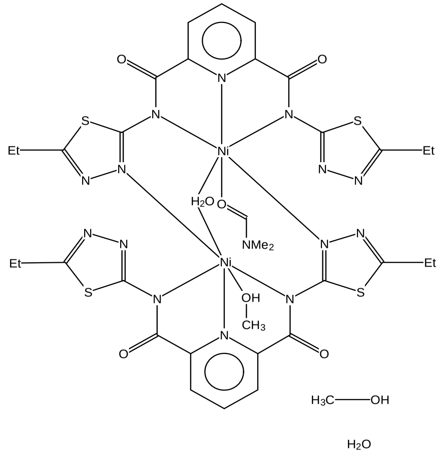

WUYDOK

Reference: Xiao-Qing Shen, Zi-Feng Li, Hong-Yun Zhang, Zhong-Jun Li (2010) *J.Coord.Chem.* ,**63,**1720

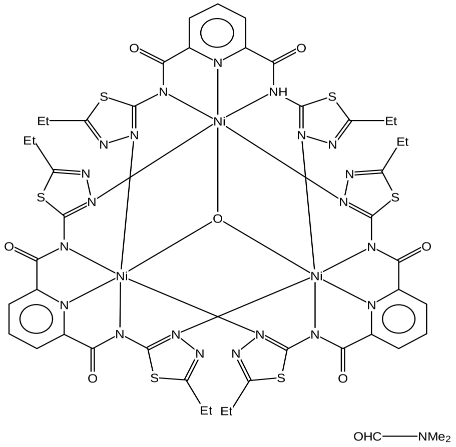

WUZNEK

Reference: J.L.Bricks, G.Reck, K.Rurack, B.Schulz, M.Spieles (2003) *Supramol.Chem.* ,**15,**189

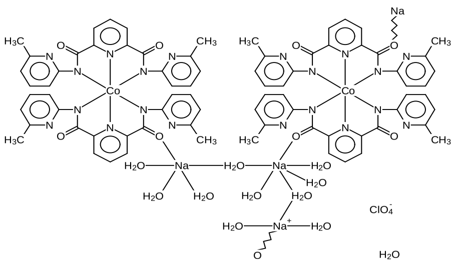

WUZNIO

Reference: J.L.Bricks, G.Reck, K.Rurack, B.Schulz, M.Spieles (2003) *Supramol.Chem.* ,**15,**189

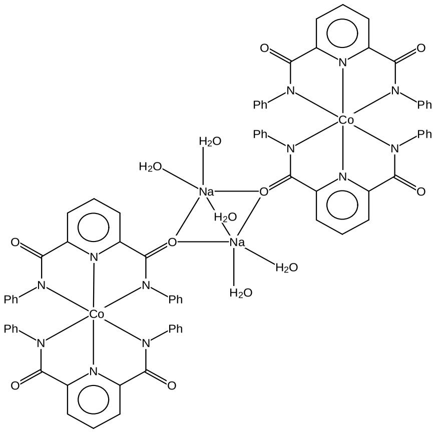

XANQAF

Reference: Sheng-Gui Liu, Wei Liu, Yi-Zhi Li, Xiao-Zeng You (2005) *Wuji Huaxue Xuebao(Chin.)(Chin.J.Inorg.Chem.)* ,**21,**543

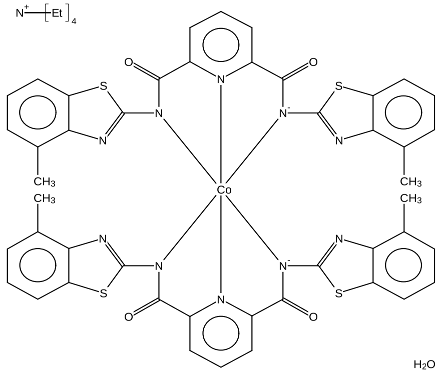

XAVWAT

Reference: N.W.Alcock, P.Moore, R.McIntyre (2005) *CSD Communication(Private Communication)* ,

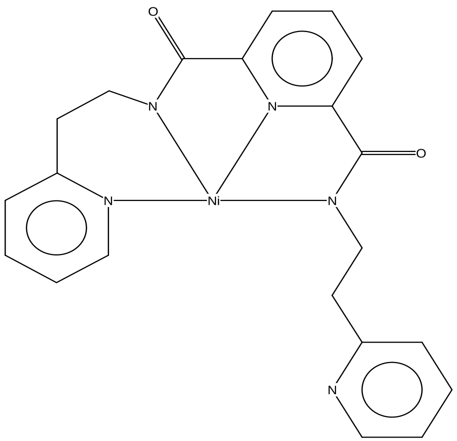

XAYHUD

Reference: P.Jerome, P.N.Sathishkumar, N.S.P.Bhuvanesh, R.Karvembu (2017) *J.Organomet.Chem.* ,**845,**115

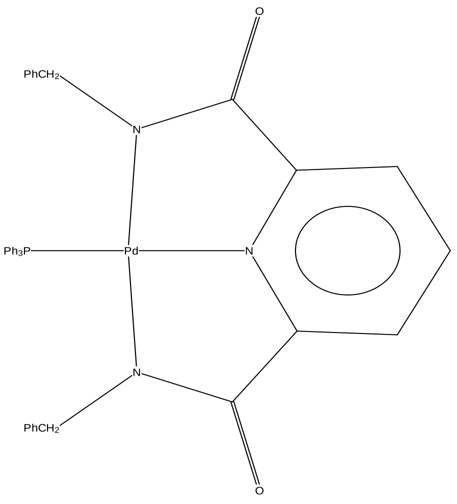

XAYHUD01

Reference: V.Carta, S.Hessam M.Mehr, M.J.MacLachlan (2018) *Inorg.Chem.* ,**57,**3243

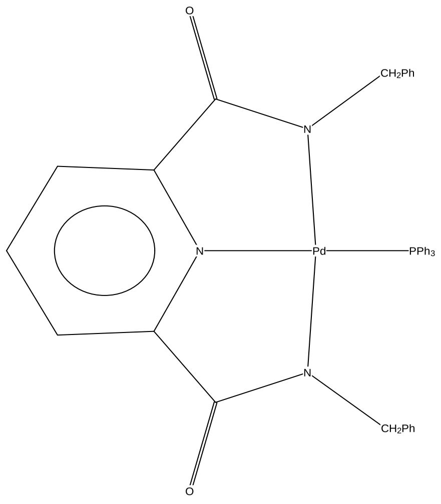

XAYJAL

Reference: P.Jerome, P.N.Sathishkumar, N.S.P.Bhuvanesh, R.Karvembu (2017) *J.Organomet.Chem.* ,**845,**115

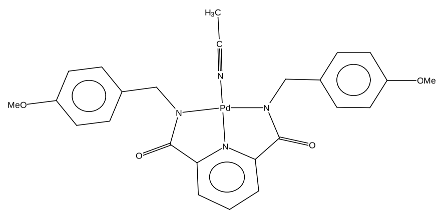

XAYJEP

Reference: P.Jerome, P.N.Sathishkumar, N.S.P.Bhuvanesh, R.Karvembu (2017) *J.Organomet.Chem.* ,**845,**115

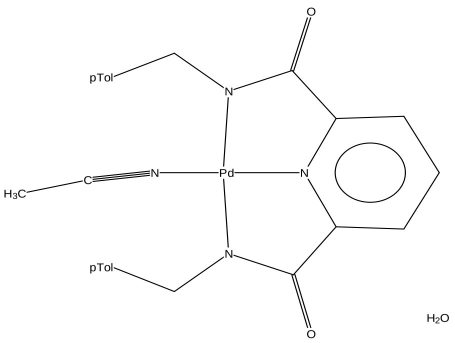

XAYJIT

Reference: P.Jerome, P.N.Sathishkumar, N.S.P.Bhuvanesh, R.Karvembu (2017) *J.Organomet.Chem.* ,**845,**115

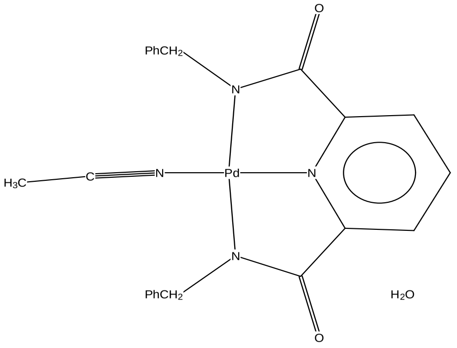

XAYJIT01

Reference: V.Carta, S.Hessam M.Mehr, M.J.MacLachlan (2018) *Inorg.Chem.* ,**57,**3243

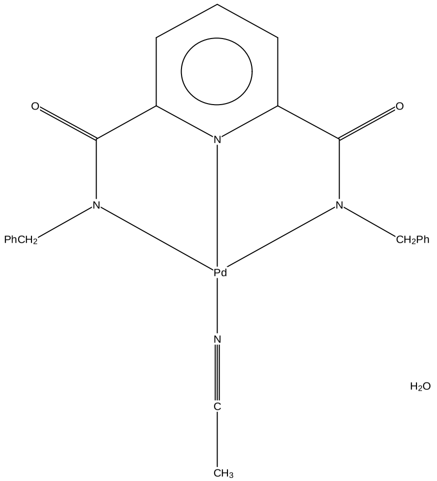

XICVIO

Reference: J.C.Noveron, M.M.Olmstead, P.K.Mascharak (2001) *J.Am.Chem.Soc.* ,**123,**3247

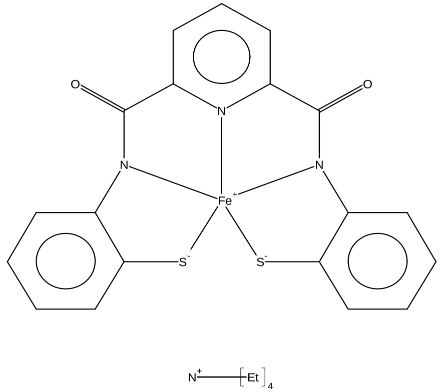

XICVOU

Reference: J.C.Noveron, M.M.Olmstead, P.K.Mascharak (2001) *J.Am.Chem.Soc.* ,**123,**3247

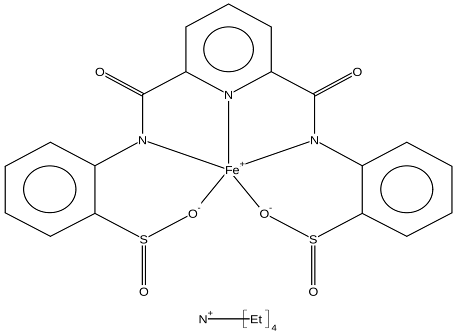

XICVUA

Reference: J.C.Noveron, M.M.Olmstead, P.K.Mascharak (2001) *J.Am.Chem.Soc.* ,**123,**3247

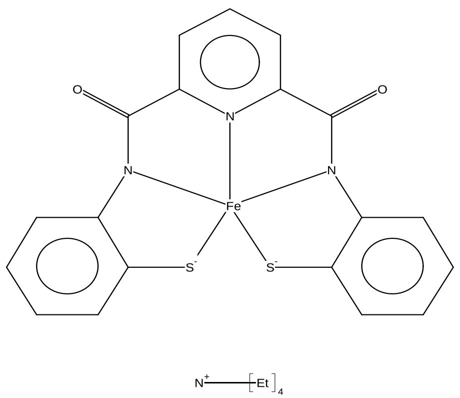

XIJPOY

Reference: P.Melle, Y.Manoharan, M.Albrecht (2018) *Inorg.Chem.* ,**57,** 11761

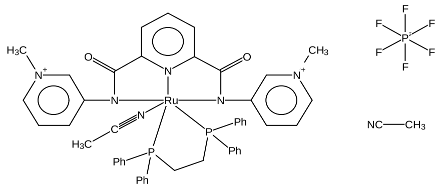

XIVGOY

Reference: Z.Shirin, J.Thompson, L.Liable-Sands, G.P.A.Yap, A.L.Rheingold, A.S.Borovik (2002) *J.Chem.Soc.,Dalton Trans.* ,1714

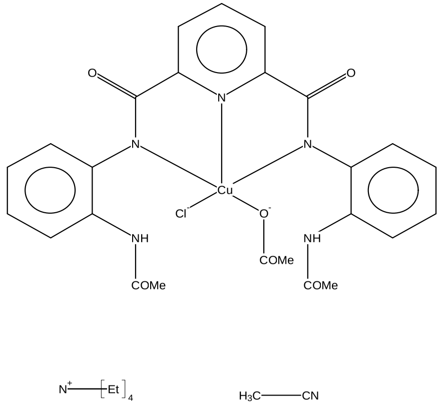

XIVGUE

Reference: Z.Shirin, J.Thompson, L.Liable-Sands, G.P.A.Yap, A.L.Rheingold, A.S.Borovik (2002) *J.Chem.Soc.,Dalton Trans.* ,1714

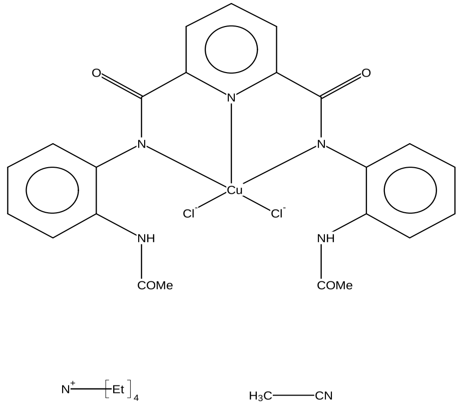

XOFFAZ

Reference: T.Moriuchi, M.Kamikawa, S.Bandoh, T.Hirao (2002) *Chem.Commun.* ,1476

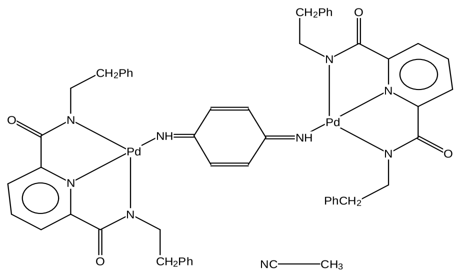

XOFFED

Reference: T.Moriuchi, M.Kamikawa, S.Bandoh, T.Hirao (2002) *Chem.Commun.* ,1476

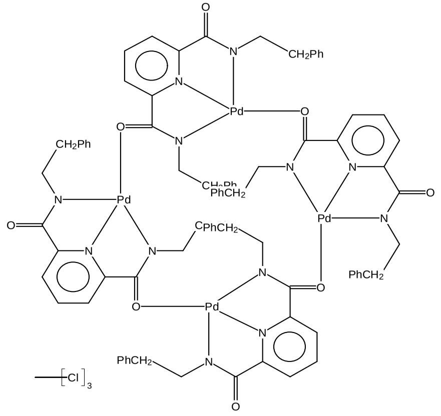

YABLEW

Reference: P.Melle, N.Segaud, M.Albrecht (2020) *Dalton Trans.* ,**49,** 12662

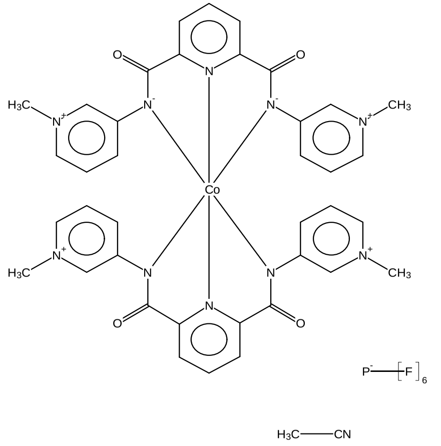

YABLIA

Reference: P.Melle, N.Segaud, M.Albrecht (2020) *Dalton Trans.* ,**49,** 12662

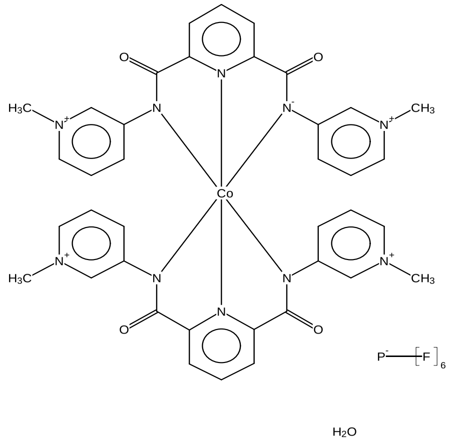

Search: search13 (Wed May 29 14:10:34 2024): Hits 553-560

YABLOG

Reference: P.Melle, N.Segaud, M.Albrecht (2020) *Dalton Trans.* ,**49,** 12662

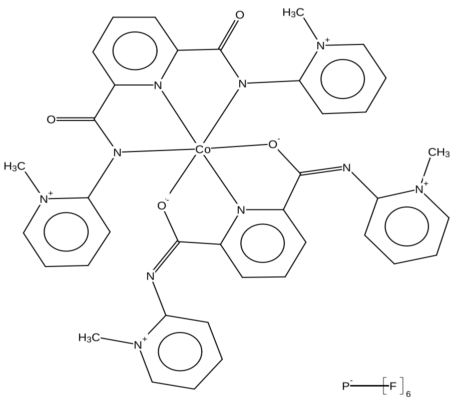

YABLUM

Reference: P.Melle, N.Segaud, M.Albrecht (2020) *Dalton Trans.* ,**49,** 12662

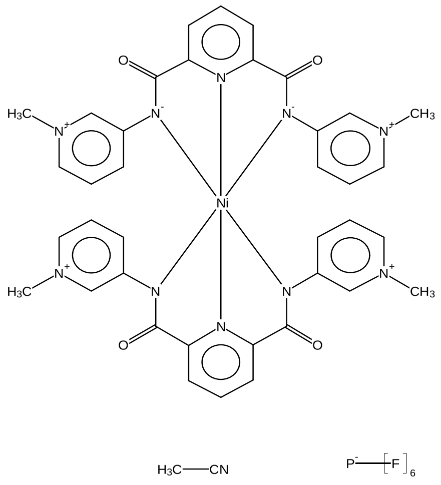

YABMAT

Reference: P.Melle, N.Segaud, M.Albrecht (2020) *Dalton Trans.* ,**49,** 12662

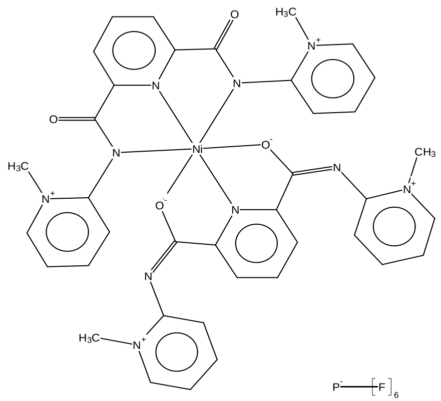

YABMEX

Reference: P.Melle, N.Segaud, M.Albrecht (2020) *Dalton Trans.* ,**49,** 12662

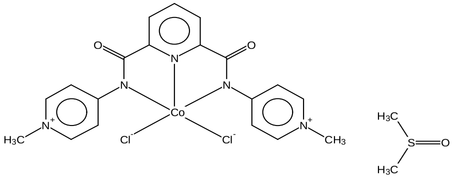

YABMIB

Reference: P.Melle, N.Segaud, M.Albrecht (2020) *Dalton Trans.* ,**49,** 12662

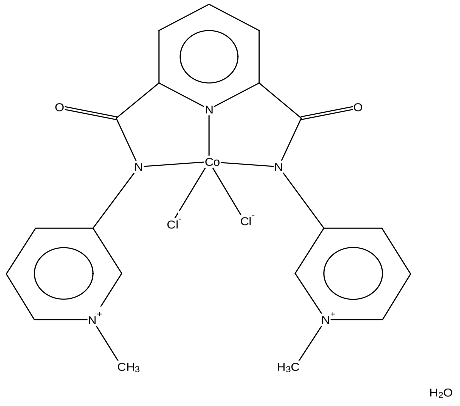

YABMUN

Reference: P.Melle, N.Segaud, M.Albrecht (2020) *Dalton Trans.* ,**49,** 12662

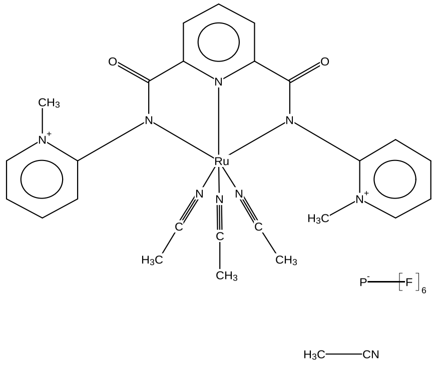

YACSOL

Reference: L.A.Tyler, J.C.Noveron, M.M.Olmstead, P.K.Mascharak (2003) *Inorg.Chem.* ,**42,**5751

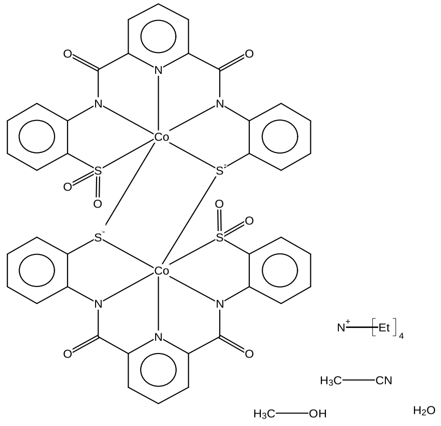

YACTAY

Reference: L.A.Tyler, J.C.Noveron, M.M.Olmstead, P.K.Mascharak (2003) *Inorg.Chem.* ,**42,**5751

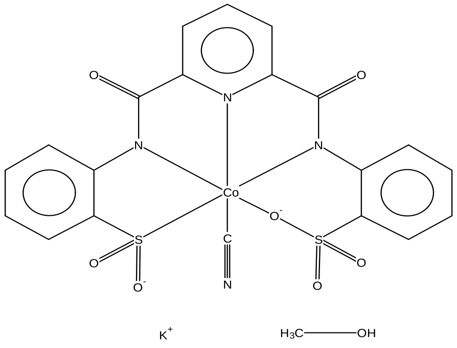

YICHAU

Reference: Sheng-Gui Liu, Chun-Lin Ni, Yi-Zhi Li (2007) *Acta Crystallogr.,Sect.E:Struct.Rep.Online* ,**63,**m1259

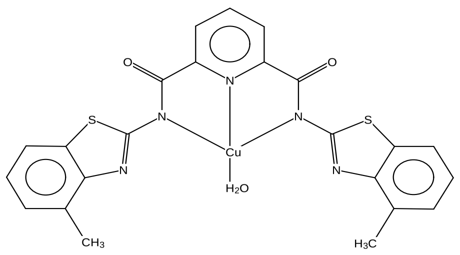

YOPGOA

Reference: Wei-Chung Hung, Liang-Yun Wang, Chien-Chen Lai, Yi-Hung Liu, Shie-Ming Peng, Sheng-Hsien Chiu (2009) *Tetrahedron Lett.* ,**50,**267

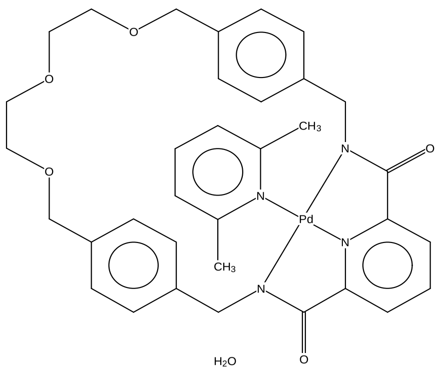

YOWDOE

Reference: J.E.Reed, A.J.P.White, S.Neidle, R.Vilar (2009) *Dalton Trans.* ,2558

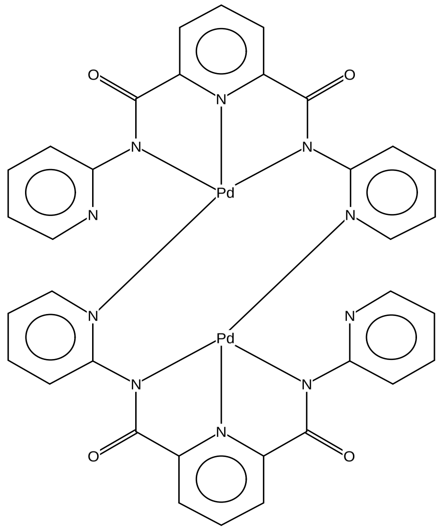

ZAQVEV

Reference: Kai Wang, Xu-Ke Huang, Li Zhu, Zi-Lu Chen, Fu-Pei Liang (2016) *Jiegou Huaxue(Chin.)(Chin.J.Struct.Chem.)* ,**35,**1912

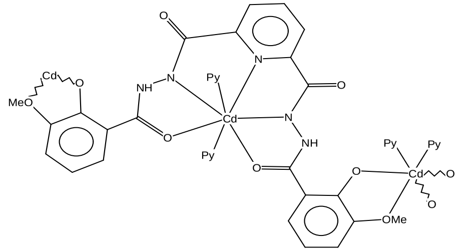

ZAZBUX

Reference: T.Kawamoto, O.Prakash, R.Ostrander, A.L.Rheingold, A.S.Borovik (1995) *Inorg.Chem.* ,**34,**4294

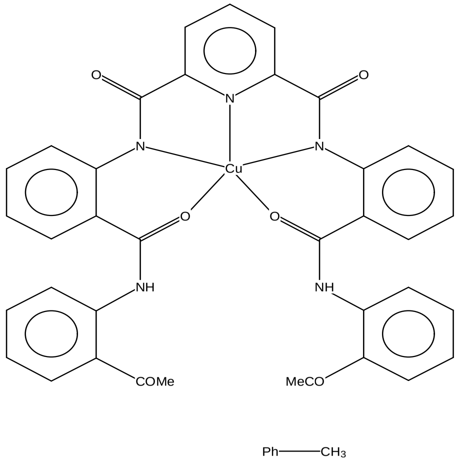

ZAZCAE

Reference: T.Kawamoto, O.Prakash, R.Ostrander, A.L.Rheingold, A.S.Borovik (1995) *Inorg.Chem.* ,**34,**4294

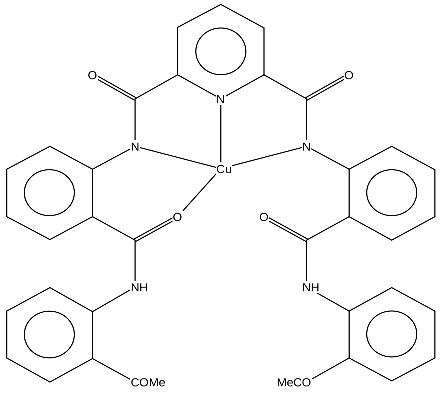

ZEFQOT

Reference: T.Corona, F.F.Pfaff, F.Acuna-Pares, A.Draksharapu, C.J.Whiteoak, J.L.Fillol, W.R.Browne, K.Ray, Anna Company (2015) *Chem.-Eur.J.* ,**21,**15029

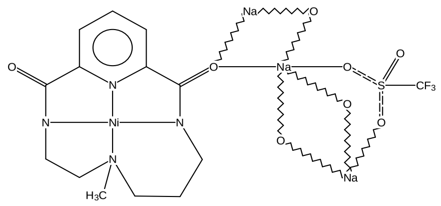

ZOFPOZ

Reference: T.Kawamoto, B.S.Hammes, B.Haggerty, G.P.A.Yap, A.L.Rheingold, A.S.Borovik (1996) *J.Am.Chem.Soc.* ,**118,**285

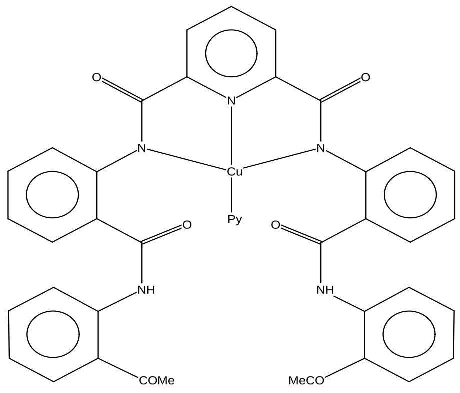

ZOFPUF

Reference: T.Kawamoto, B.S.Hammes, B.Haggerty, G.P.A.Yap, A.L.Rheingold, A.S.Borovik (1996) *J.Am.Chem.Soc.* ,**118,**285

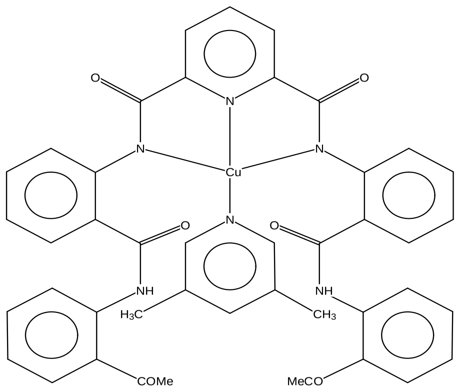

ZOYPAG

Reference: G.Kumar, G.Kumar, R.Gupta (2015) *Inorg.Chem.* ,**54,**2603

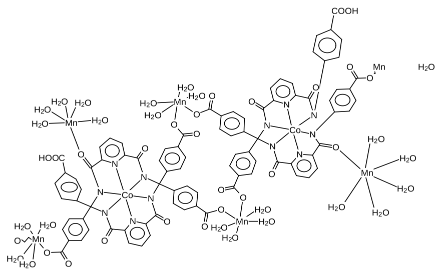

ZOYPEK

Reference: G.Kumar, G.Kumar, R.Gupta (2015) *Inorg.Chem.* ,**54,**2603

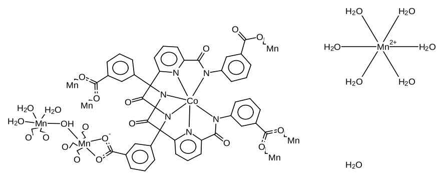

ZOYPIO

Reference: G.Kumar, G.Kumar, R.Gupta (2015) *Inorg.Chem.* ,**54,**2603

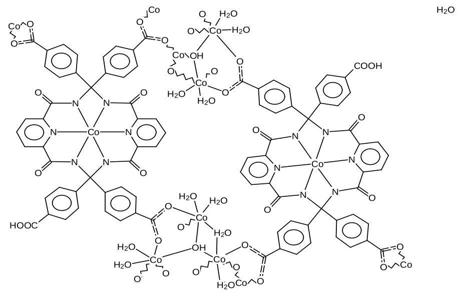

ZOYPOU

Reference: G.Kumar, G.Kumar, R.Gupta (2015) *Inorg.Chem.* ,**54,**2603

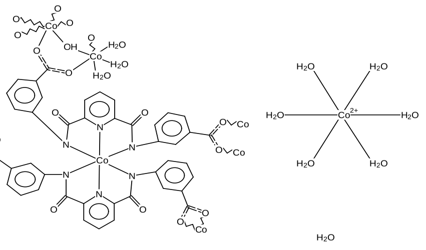

ZOYRAI

Reference: G.L.Guillet, J.B.Gordon, G.N.Di Francesco, M.W.Calkins, E.Cizmar, K.A.Abboud, M.W.Meisel, R.Garcia-Serres, L.J.Murray (2015) *Inorg.Chem.* ,**54,**2691

Ni_2--
--[μ_2-$C_{59}H_{25}N_9O_6(Pr^i)_6(OEt)_6$-$\kappa^6$N,N',N'',N''',N'''',N''''']$[HO(COO)][HO]^{2-}$

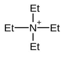

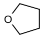

EtO—Et

H_2O

ZOYREM

Reference: G.L.Guillet, J.B.Gordon, G.N.Di Francesco, M.W.Calkins, E.Cizmar, K.A.Abboud, M.W.Meisel, R.Garcia-Serres, L.J.Murray (2015) *Inorg.Chem.* ,**54,**2691

Ni_2Na--
--[μ_3-$C_{59}H_{25}N_9O_6(Pr^i)_6(OEt)_6$-$\kappa^8$N,N',N'',N''',N'''',N''''',N'''''']$[\mu_2$-CHO_3-κ^2O,O'][C_4H_8O][$H_2O]^{2-}$

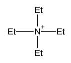

EtO—Et

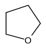

ZOYTAK

Reference: G.L.Guillet, J.B.Gordon, G.N.Di Francesco, M.W.Calkins, E.Cizmar, K.A.Abboud, M.W.Meisel, R.Garcia-Serres, L.J.Murray (2015) *Inorg.Chem.* ,**54,**2691

Cu_3K--
--[μ_3-$C_{59}H_{23}N_9O_6(Pr^i)_6(OEt)_6$-$\kappa^9$N,N',N'',N''',N'''',N''''',N'''''',N''''''',N'''''''']$[\mu_2$-$Cl]_3^{2-}$

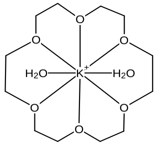

ZOYTUE

Reference: G.L.Guillet, J.B.Gordon, G.N.Di Francesco, M.W.Calkins, E.Cizmar, K.A.Abboud, M.W.Meisel, R.Garcia-Serres, L.J.Murray (2015) *Inorg.Chem.* ,**54,**2691

Cu_3--

--[μ_2-$C_{61}H_{26}N_9O_8(Pr^i)_6(OEt)_4Me_2$-$\kappa^6$N,N',N'',N''',N'''',N''''']][μ_2-CO_3-κ^3O,O',O'']][μ_2-HO][HO]$^{2-}$

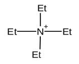

ZOZDUP

Reference: G.L.Guillet, J.B.Gordon, G.N.Di Francesco, M.W.Calkins, E.Cizmar, K.A.Abboud, M.W.Meisel, R.Garcia-Serres, L.J.Murray (2015) *Inorg.Chem.* ,**54,**2691

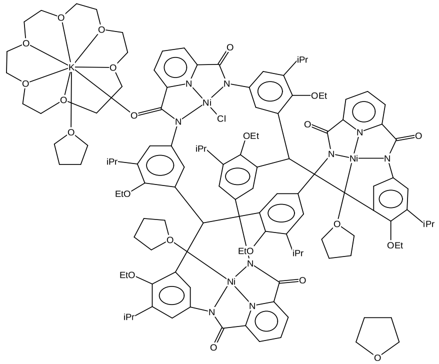

ZUBSOE

Reference: F.A.Chavez, M.M.Olmstead, P.K.Mascharak (1996) *Inorg.Chem.* ,**35,**1410

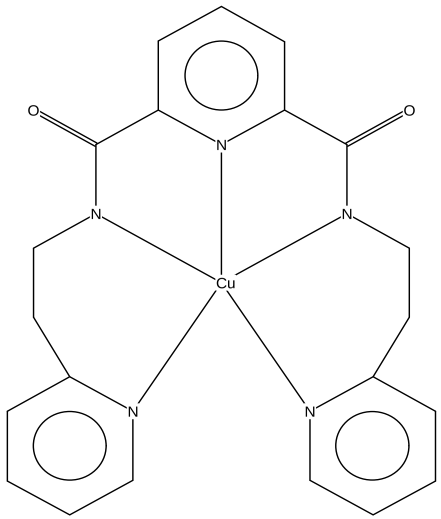

CATDAG

Reference: J.K.Bower, L.M.Zarnitsa, I.M.Mazin, A.D.Cypcar, C.E.Moore, A.Yu.Sokolov, Shiyu Zhang (2022) *CSD Communication(Private Communication)* ,

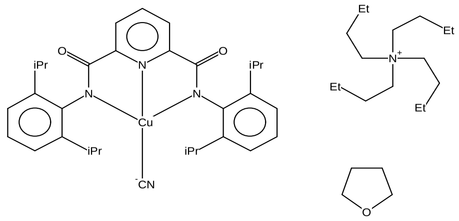

CATDEK

Reference: J.K.Bower, L.M.Zarnitsa, I.M.Mazin, A.D.Cypcar, C.E.Moore, A.Yu.Sokolov, Shiyu Zhang (2022) *CSD Communication(Private Communication)* ,

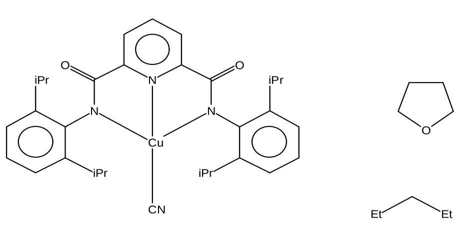

SENZAQ

Reference: A.J.Bukvic, M.Albrecht (2022) *Inorg.Chem.* ,

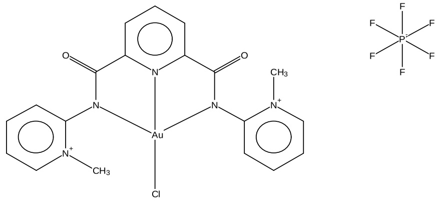

SENZEU

Reference: A.J.Bukvic, M.Albrecht (2022) *Inorg.Chem.* ,

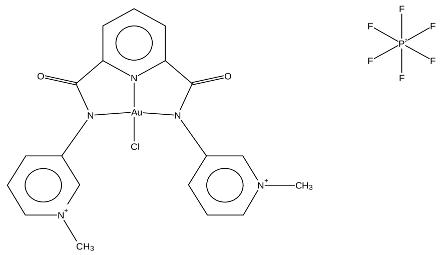

SENZIY

Reference: A.J.Bukvic, M.Albrecht (2022) *Inorg.Chem.* ,

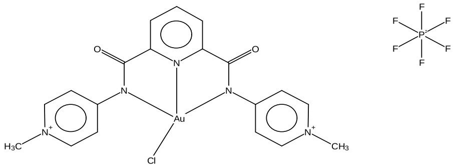

SENZUK

Reference: A.J.Bukvic, M.Albrecht (2022) *Inorg.Chem.* ,

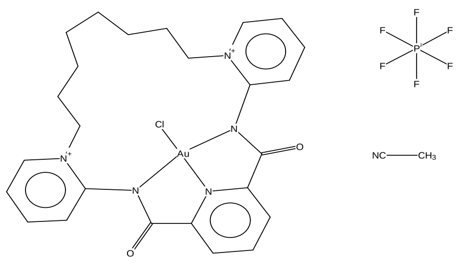

SEPDEA

Reference: A.J.Bukvic, M.Albrecht (2022) *Inorg.Chem.* ,**61,**14038

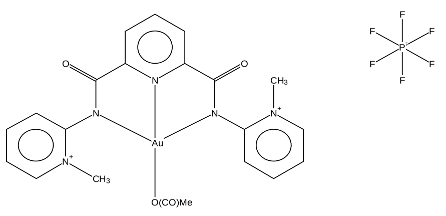

Pd(II) Complexes

N^{-}^N^N^{-} Coordination

Search Overview

Search:	search14
Date/Time done:	Wed May 29 14:12:09 2024
Database(s):	CSD version 5.43 updates (Mar 2022)<br>CSD version 5.43 (November 2021)<br>CSD version 5.43 updates (Sep 2022)<br>CSD version 5.43 updates (Nov 2022)
Restriction Info:	No refcode restrictions applied
Filters:	None
Percentage Completed:	100%
Number of Hits:	98

Single query used. Search found structures that:

match

Query 1

Query 1

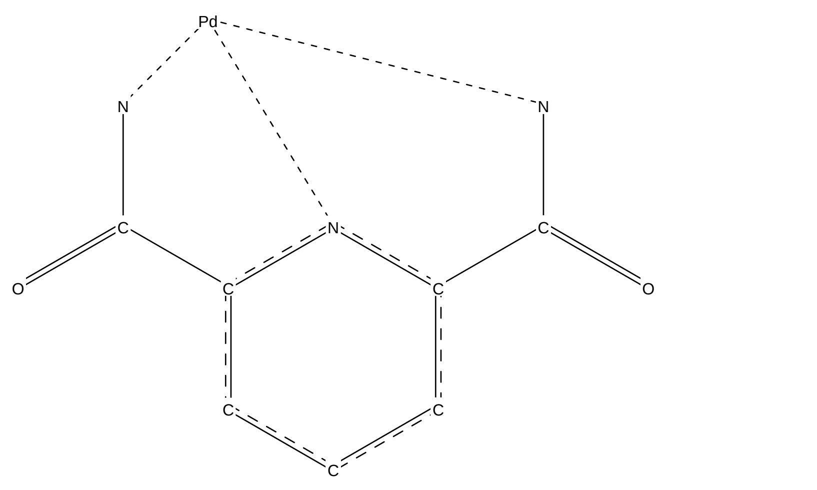

Search: search14 (Wed May 29 14:12:09 2024): Hits 1-8

BANYIB

Reference: P.Kumar, V.Kumar, R.Gupta (2017) *RSC Advances* ,**7**, 7734

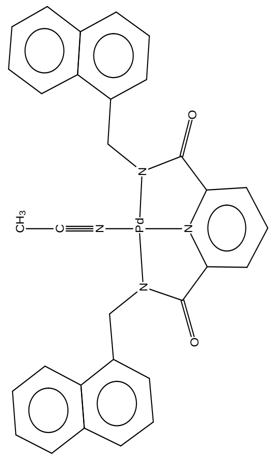

ADEKAX

Reference: T.Moriuchi, K.Morimoto, Y.Sakamoto, T.Hirao (2012) *Eur.J.Inorg.Chem.* ,4669

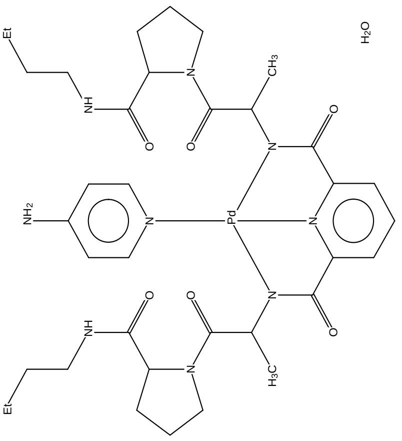

ADEJUQ

Reference: T.Moriuchi, K.Morimoto, Y.Sakamoto, T.Hirao (2012) *Eur.J.Inorg.Chem.* ,4669

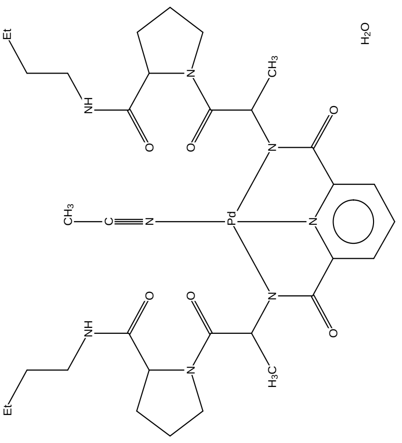

ENESEY

Reference: Lixian Wen (2021) *CSD Communication(Private Communication)* ,

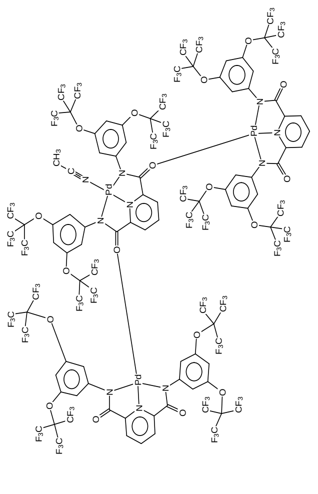

BEXNOK

Reference: V.Carta, S.Hessam M.Mehr, M.J.MacLachlan (2018) *Inorg.Chem.* ,**57**,3243

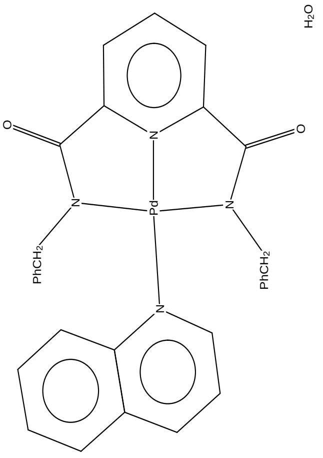

BEXNEA

Reference: V.Carta, S.Hessam M.Mehr, M.J.MacLachlan (2018) *Inorg.Chem.* ,**57**,3243

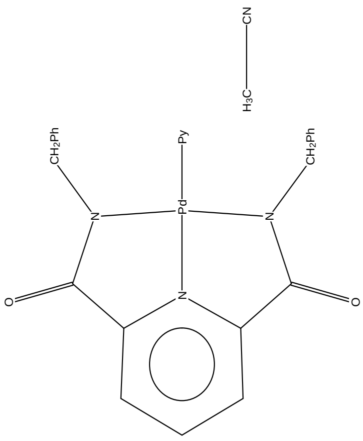

BEXMUP

Reference: V.Carta, S.Hessam M.Mehr, M.J.MacLachlan (2018) *Inorg.Chem.* ,**57**,3243

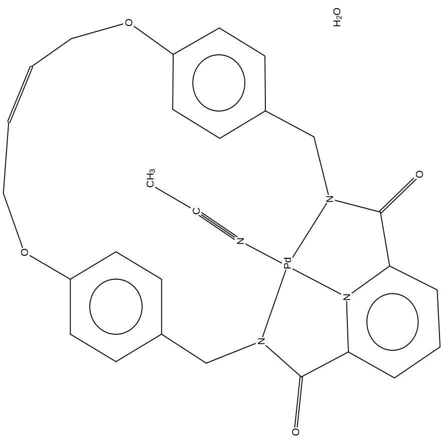

BEHLEI

Reference: B.Nisar Ahamed, P.Van Velthem, K.Robeyns, C.-A.Fustin (2017) *ACS Macro Letters* ,**6**,468

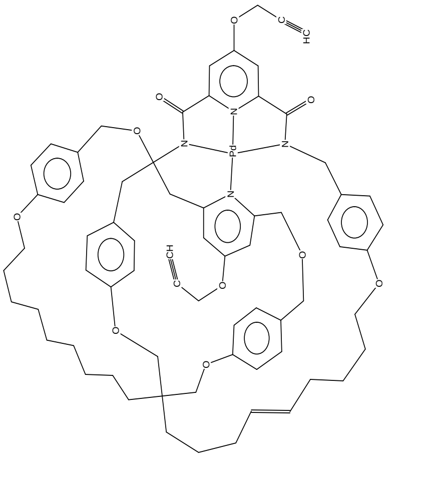

Search: search14 (Wed May 29 14:12:09 2024): Hits 9-16

BEXPIG

Reference: V.Carta, S.Hessam M.Mehr, M.J.MacLachlan (2018) *Inorg.Chem.* ,**57**,3243

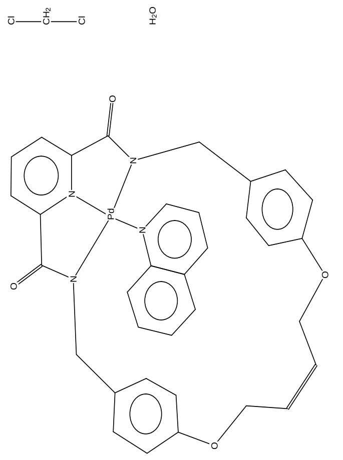

CEXYIQ

Reference: Sung-Ok Kang, T.S.Johnson, V.W.Day, K.Bowman-James (2018) *Supramol.Chem.* ,**30**,305

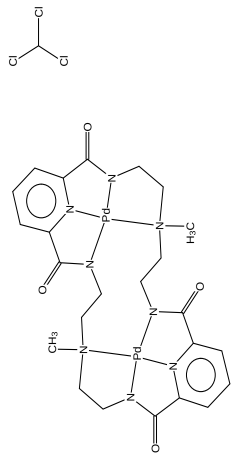

BEXPEC

Reference: V.Carta, S.Hessam M.Mehr, M.J.MacLachlan (2018) *Inorg.Chem.* ,**57**,3243

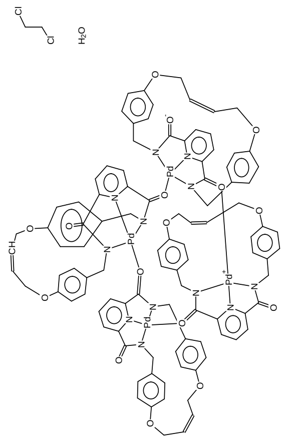

BITRUU

Reference: Qiaoqiao Teng, Han Vinh Huynh (2018) *Organometallics* , **37**,4119

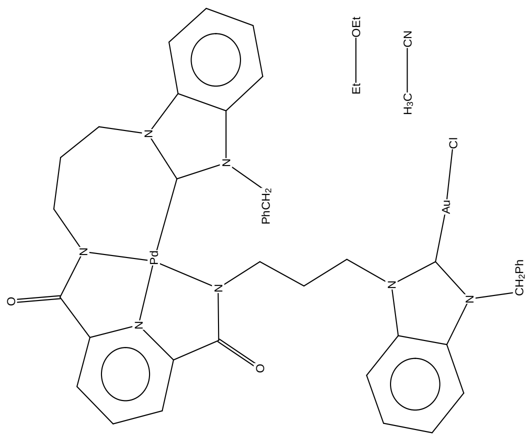

BEXPAY

Reference: V.Carta, S.Hessam M.Mehr, M.J.MacLachlan (2018) *Inorg.Chem.* ,**57**,3243

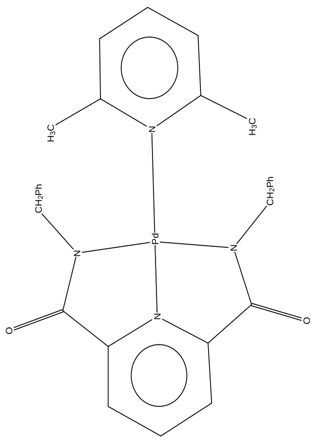

BITROO

Reference: Qiaoqiao Teng, Han Vinh Huynh (2018) *Organometallics* , **37**,4119

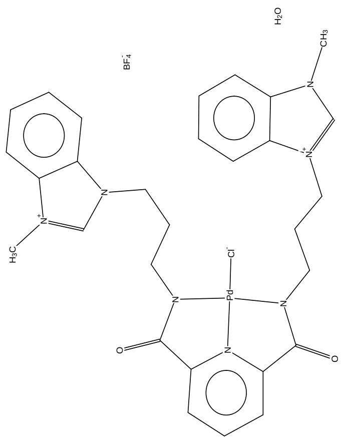

BEXNUQ

Reference: V.Carta, S.Hessam M.Mehr, M.J.MacLachlan (2018) *Inorg.Chem.* ,**57**,3243

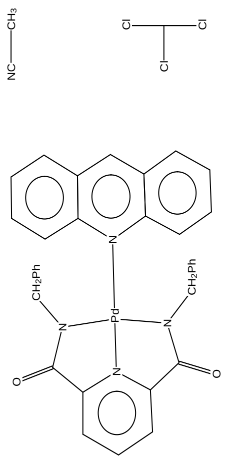

BEXPOM

Reference: V.Carta, S.Hessam M.Mehr, M.J.MacLachlan (2018) *Inorg.Chem.* ,**57**,3243

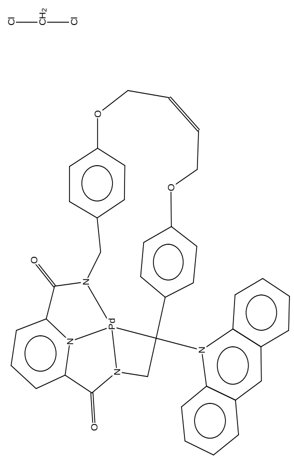

COHHIR

Reference: T.Moriuchi, Y.Takagi, T.Hirao (2008) *Eur.J.Inorg.Chem.* , 3877

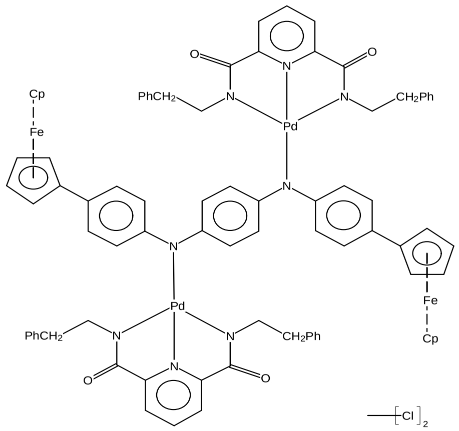

DOGKER

Reference: Qi-Qiang Wang, R.A.Begum, V.W.Day, K.Bowman-James (2013) *J.Am.Chem.Soc.* ,**135,**17193

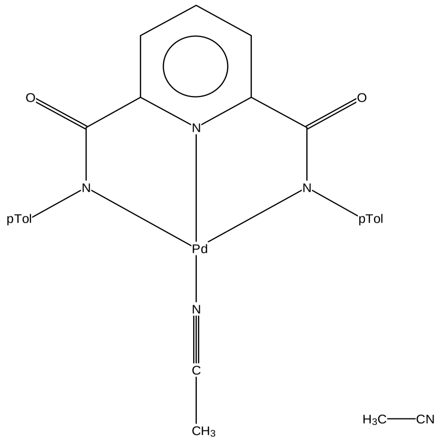

DOGKIV

Reference: Qi-Qiang Wang, R.A.Begum, V.W.Day, K.Bowman-James (2013) *J.Am.Chem.Soc.* ,**135,**17193

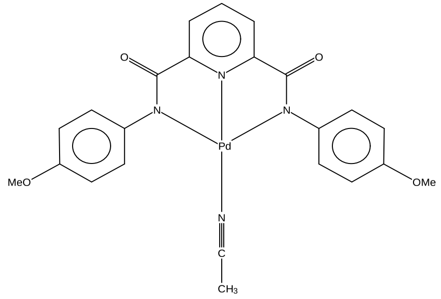

DOGKOB

Reference: Qi-Qiang Wang, R.A.Begum, V.W.Day, K.Bowman-James (2013) *J.Am.Chem.Soc.* ,**135,**17193

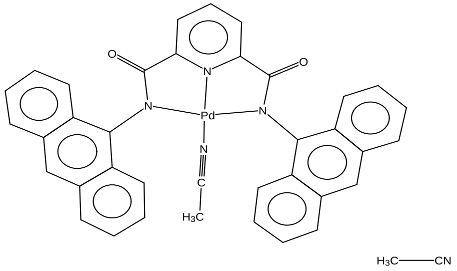

DOGKUH

Reference: Qi-Qiang Wang, R.A.Begum, V.W.Day, K.Bowman-James (2013) *J.Am.Chem.Soc.* ,**135,**17193

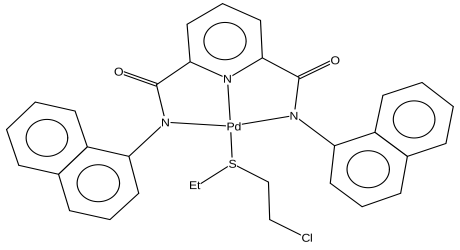

DOGLAO

Reference: Qi-Qiang Wang, R.A.Begum, V.W.Day, K.Bowman-James (2013) *J.Am.Chem.Soc.* ,**135,**17193

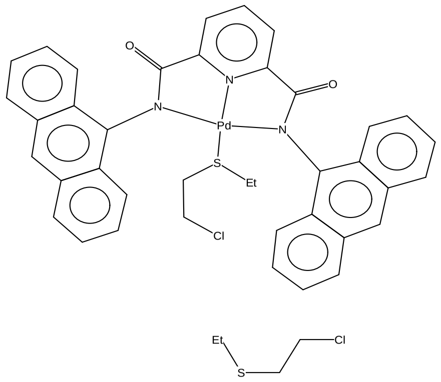

FECWOA

Reference: Y.Furusho, T.Matsuyama, T.Takata, T.Moriuchi, T.Hirao (2004) *Tetrahedron Lett.* ,**45,**9593

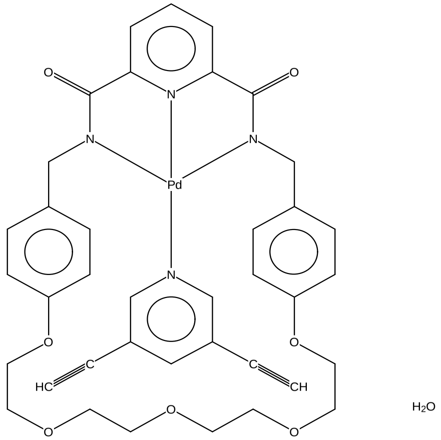

FIVKAZ

Reference: V.Carta, M.A.Soto, M.J.MacLachlan (2019) *Chem.Commun.* ,**55,**1245

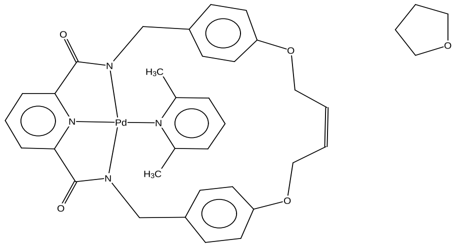

FIVKED

Reference: V.Carta, M.A.Soto, M.J.MacLachlan (2019) *Chem.Commun.* ,**55,**1245

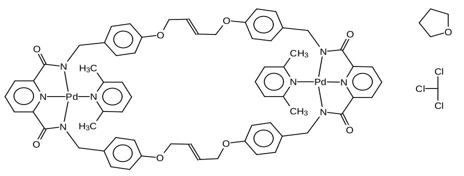

FOJHOD

Reference: J.E.Beves, V.Blanco, B.A.Blight, R.Carrillo, D.M.DSouza, D.Howgego, D.A.Leigh, A.M.Z.Slawin, M.D.Symes (2014) *J.Am.Chem.Soc.* ,**136,**2094

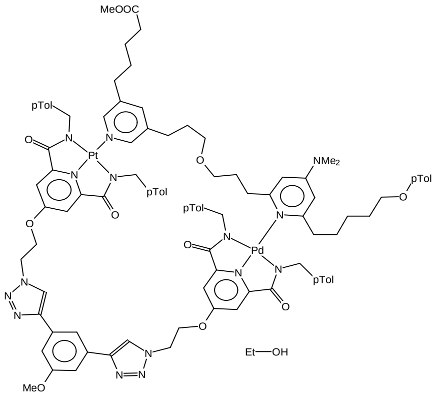

HAZKID

Reference: D.B.Dell'Amico, F.Calderazzo, F.Di Colo, G.Guglielmetti, L.Labella, F.Marchetti (2006) *Inorg.Chim.Acta* ,**359,**127

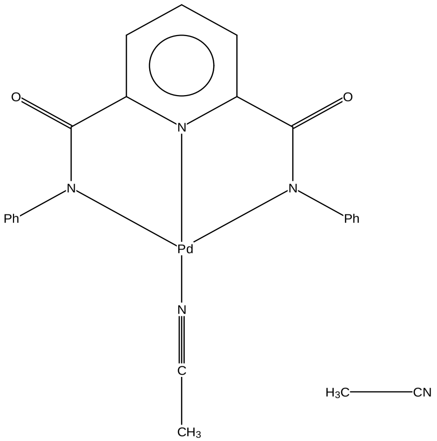

HAZKOJ

Reference: D.B.Dell'Amico, F.Calderazzo, F.Di Colo, G.Guglielmetti, L.Labella, F.Marchetti (2006) *Inorg.Chim.Acta* ,**359,**127

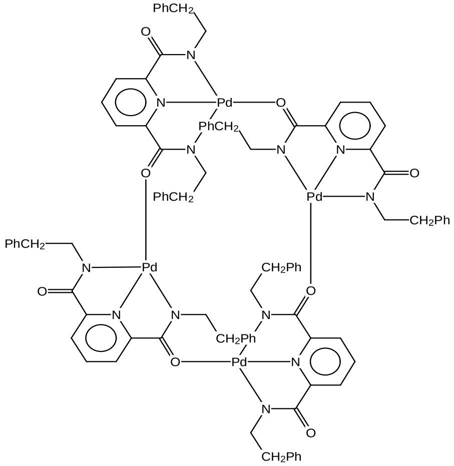

HIZRUF

Reference: N.Zigon, P.Larpent, A.Jouaiti, N.Kyritsakas, M.W.Hosseini (2014) *Chem.Commun.* ,**50,**5040

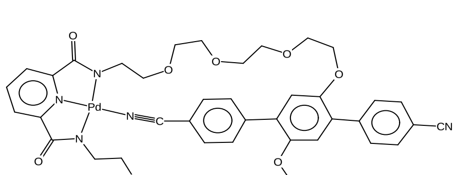

HUCCEQ

Reference: A.L.Rheingold, A.S.Borovik (2019) *CSD Communication(Private Communication)* ,

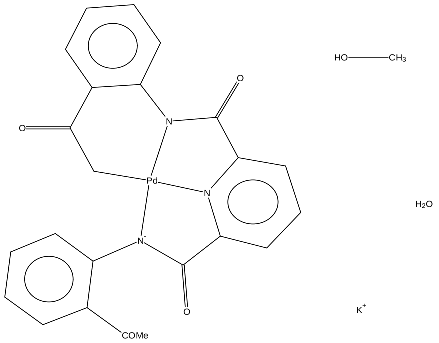

IZUXEG

Reference: X.Shen, T.Moriuchi, T.Hirao (2004) *Tetrahedron Lett.* ,**45,** 4733

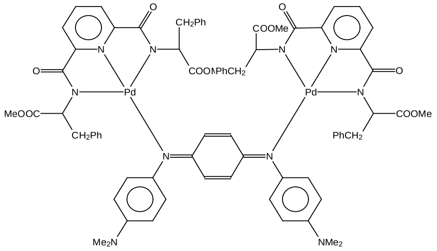

IZUXEG01

Reference: T.Moriuchi, Xiuliang Shen, T.Hirao (2006) *Tetrahedron* ,**62,** 12237

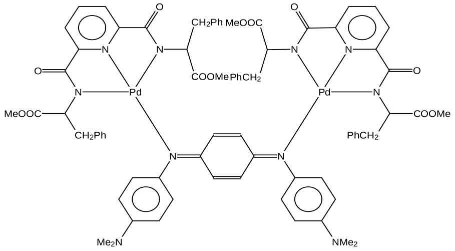

JIPGEV

Reference: B.Walker, D.Leigh, S.Parsons (2007) *CSD Communication(Private Communication)* ,

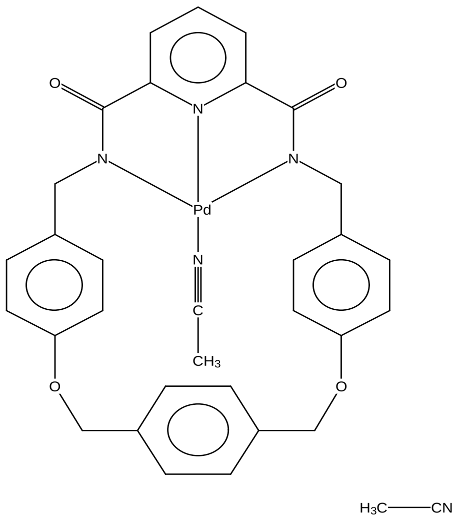

JITZAO

Reference: J.D.Crowley, D.A.Leigh, P.J.Lusby, R.T.McBurney, L.-E.Perret-Aebi, C.Petzold, A.M.Z.Slawin, M.D.Symes (2007) *J.Am.Chem.Soc.* ,**129**,15085

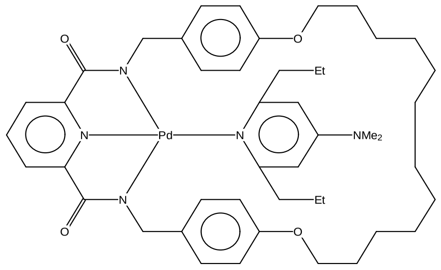

JITZES

Reference: J.D.Crowley, D.A.Leigh, P.J.Lusby, R.T.McBurney, L.-E.Perret-Aebi, C.Petzold, A.M.Z.Slawin, M.D.Symes (2007) *J.Am.Chem.Soc.* ,**129**,15085

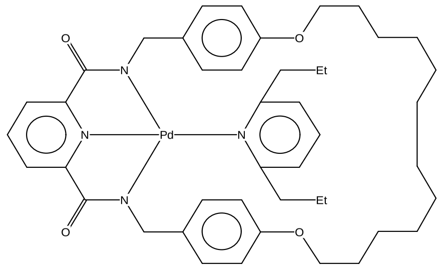

JUTWUS

Reference: P.Ludomenov, D.Leigh, Iain Oswald, S.Parsons (2015) *CSD Communication(Private Communication)* ,

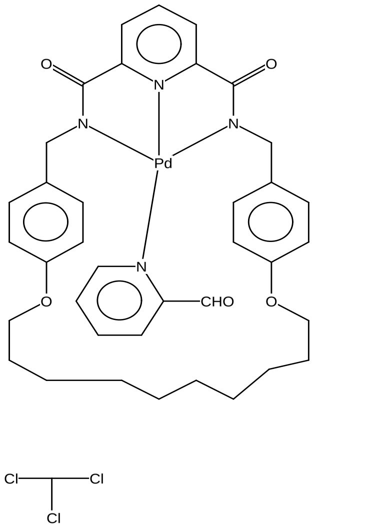

KEDTIZ

Reference: P.Kumar, V.Kumar, R.Gupta (2017) *Dalton Trans.* ,**46,** 10205

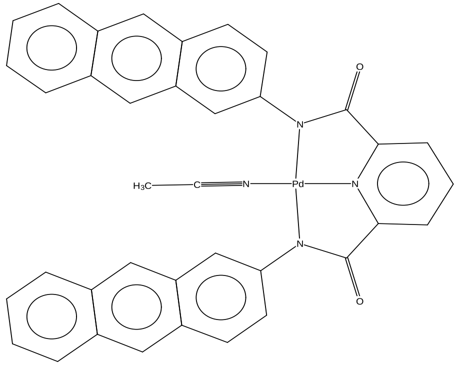

KIWFOO

Reference: T.K.Achar, K.Ramakrishna, T.Pal, S.Porey, P.Dolui, J.P.Biswas, D.Maiti (2018) *Chem.-Eur.J.* ,**24,**17906

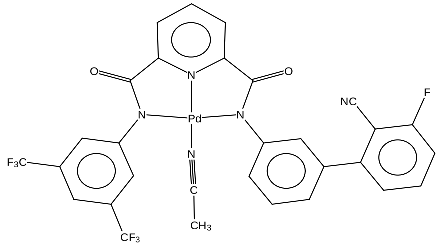

KOBNOF

Reference: S.M.Goldup, D.A.Leigh, P.J.Lusby, R.T.McBurney, A.M.Z.Slawin (2008) *Angew.Chem.,Int.Ed.* ,**47**,3381

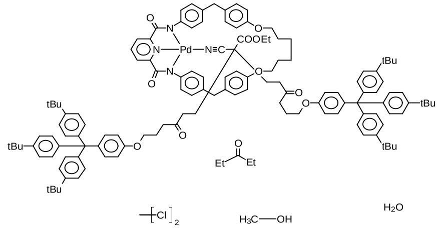

KOBNUL

Reference: S.M.Goldup, D.A.Leigh, P.J.Lusby, R.T.McBurney, A.M.Z.Slawin (2008) *Angew.Chem.,Int.Ed.* ,**47**,3381

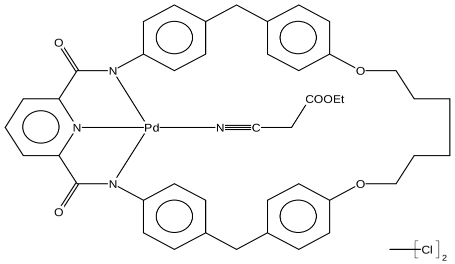

KOBPAT

Reference: S.M.Goldup, D.A.Leigh, P.J.Lusby, R.T.McBurney, A.M.Z.Slawin (2008) *Angew.Chem.,Int.Ed.* ,**47**,3381

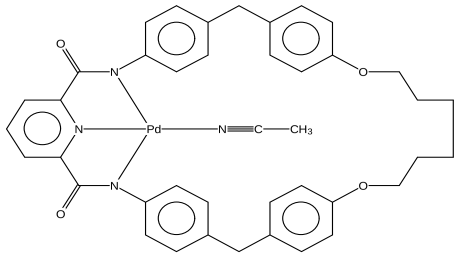

KUGSUC

Reference: M.Ogawa, M.Nagashima, H.Sogawa, S.Kuwata, T.Takata (2015) *Org.Lett.* ,**17**,1664

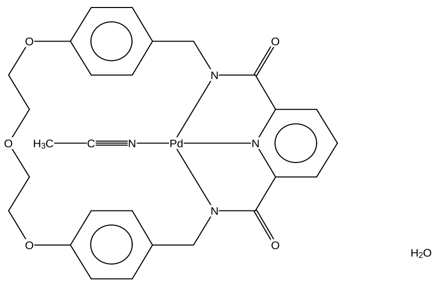

KUGTAJ

Reference: M.Ogawa, M.Nagashima, H.Sogawa, S.Kuwata, T.Takata (2015) *Org.Lett.* ,**17**,1664

$Pd_4[\mu_2\text{-}C_{27}H_{27}N_3O_6\text{-}\kappa^3N,N',N'',\kappa^1O]_4$

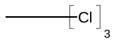

LUZTIL

Reference: Yanchuan Zhao, Lily Chen, T.M.Swager (2016) *Angew.Chem.,Int.Ed.* ,**55**,917

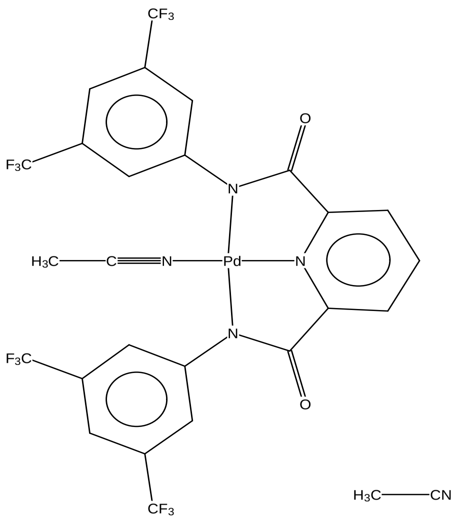

LUZTOR

Reference: Yanchuan Zhao, Lily Chen, T.M.Swager (2016) *Angew.Chem.,Int.Ed.* ,**55**,917

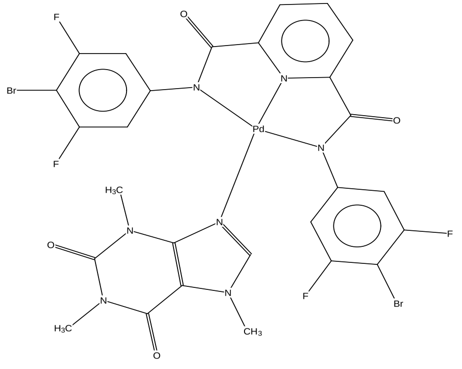

MARVAC

Reference: T.Moriuchi, S.Bandoh, Y.Miyaji, T.Hirao (2000) *J.Organomet.Chem.* ,**599**,135

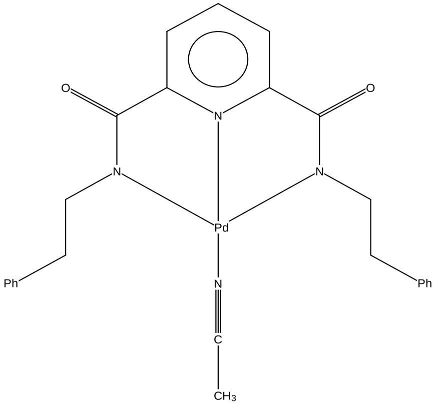

MARVEG

Reference: T.Moriuchi, S.Bandoh, Y.Miyaji, T.Hirao (2000) *J.Organomet.Chem.* ,**599**,135

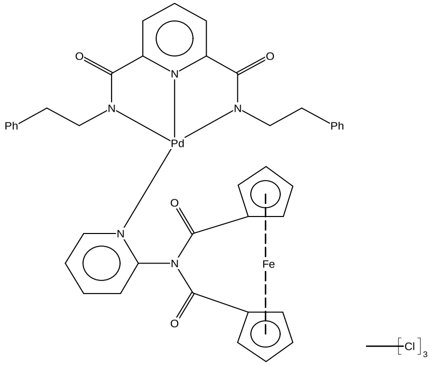

MIMWUB

Reference: B.Walker, D.Leigh, S.Parsons, I.Oswald, R.D.L.Johnstone (2007) *CSD Communication(Private Communication)* ,

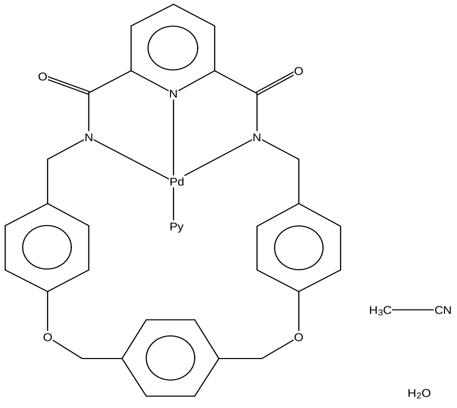

Search: search14 (Wed May 29 14:12:09 2024): Hits 49-56

NURJAM

Reference: N.Miyagawa, M.Watanabe, T.Matsuyama, Y.Koyama, T.Moriuchi, T.Hirao, Y.Furusho, T.Takata (2010) *Chem.Commun.* ,**46,** 1920

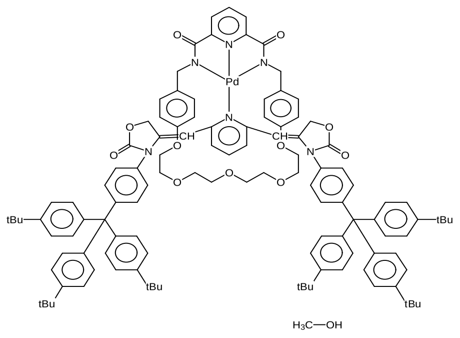

NURJEQ

Reference: N.Miyagawa, M.Watanabe, T.Matsuyama, Y.Koyama, T.Moriuchi, T.Hirao, Y.Furusho, T.Takata (2010) *Chem.Commun.* ,**46,** 1920

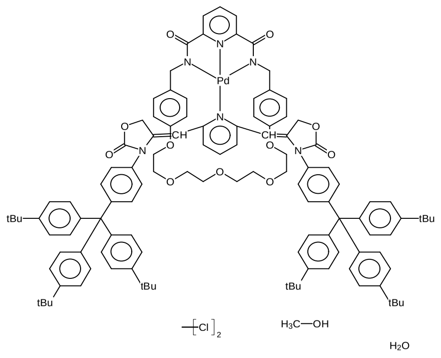

OBOMOL

Reference: S.S.P.Malaza, G.Mehlana, Ocean Cheung, R.Hunter, B.C.E.Makhubela (2021) *CrystEngComm* ,

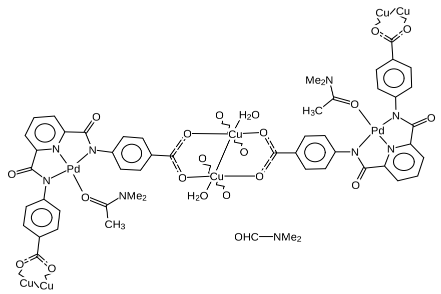

OPEJEA

Reference: B.Nisar Ahamed, R.Duchene, K.Robeyns, C.-A.Fustin (2016) *Chem.Commun.* ,**52,**2149

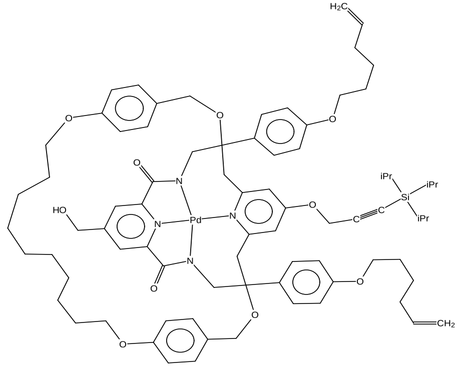

OPEJIE

Reference: B.Nisar Ahamed, R.Duchene, K.Robeyns, C.-A.Fustin (2016) *Chem.Commun.* ,**52,**2149

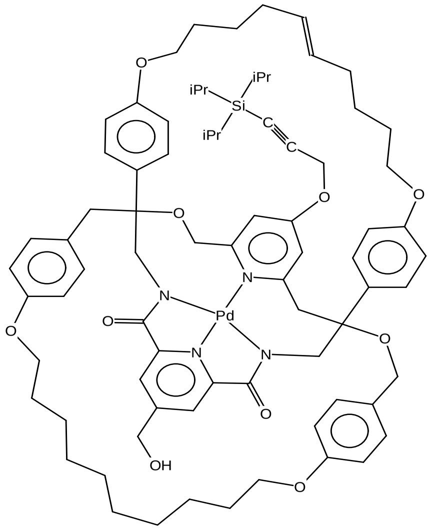

OPEJOK

Reference: B.Nisar Ahamed, R.Duchene, K.Robeyns, C.-A.Fustin (2016) *Chem.Commun.* ,**52,**2149

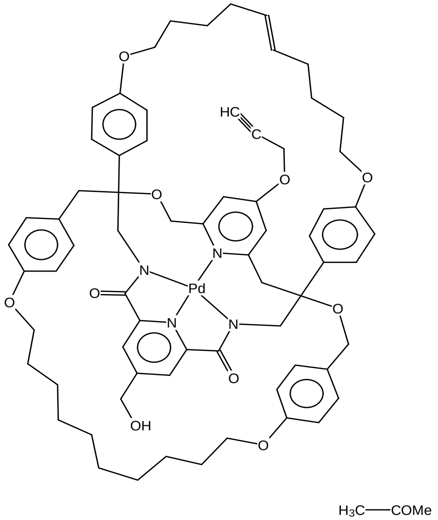

OPOKAG

Reference: Y.Perez, A.L.Johnson, P.R.Raithby (2011) *Polyhedron* ,**30,** 284

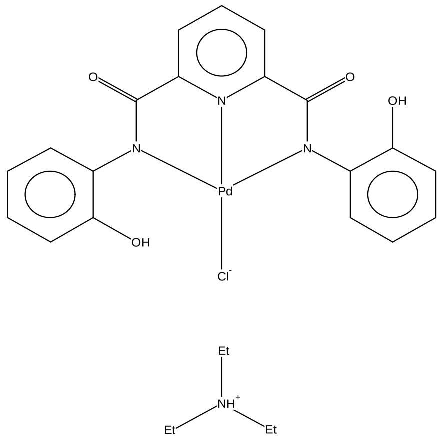

PADQIW

Reference: Deguang Huang, R.H.Holm (2010) *J.Am.Chem.Soc.* ,**132,** 4693

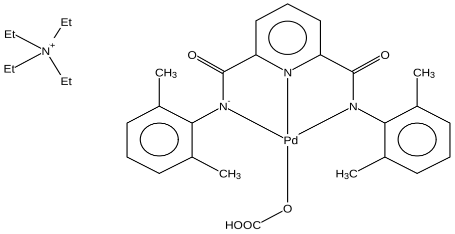

PADRAP

Reference: Deguang Huang, R.H.Holm (2010) *J.Am.Chem.Soc.* ,**132,** 4693

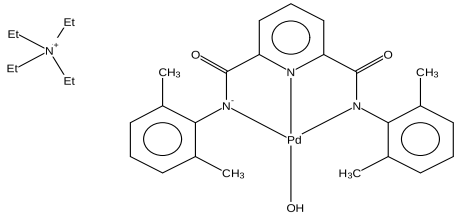

PAFHEK

Reference: A.-M.Fuller, D.A.Leigh, P.J.Lusby, I.D.H.Oswald, S.Parsons, D.B.Walker (2004) *Angew.Chem.,Int.Ed.* ,**43,**3914

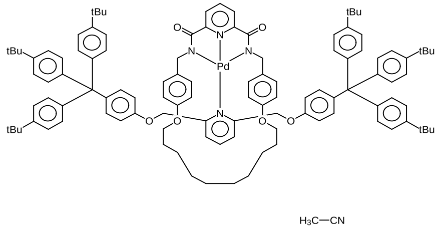

QALNOG

Reference: T.Moriuchi, S.Bandoh, M.Kamikawa, T.Hirao (2000) *Chem.Lett.* ,148

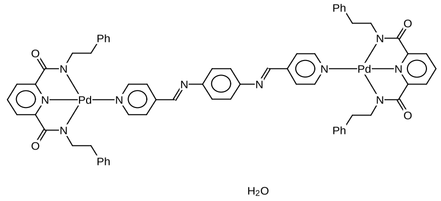

QAVGIE

Reference: A.-M.L.Fuller, D.A.Leigh, P.J.Lusby, A.M.Z.Slawin, D.B.Walker (2005) *J.Am.Chem.Soc.* ,**127,**12612

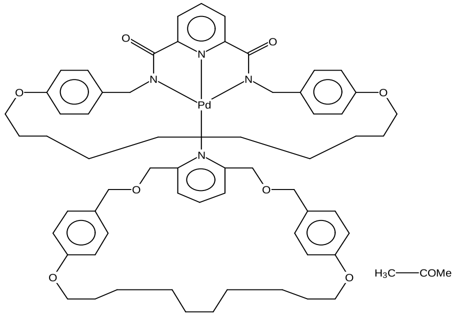

QAVGOK

Reference: A.-M.L.Fuller, D.A.Leigh, P.J.Lusby, A.M.Z.Slawin, D.B.Walker (2005) *J.Am.Chem.Soc.* ,**127,**12612

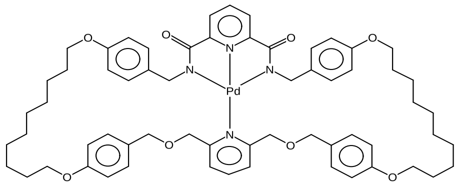

QAVGUQ

Reference: A.-M.L.Fuller, D.A.Leigh, P.J.Lusby, A.M.Z.Slawin, D.B.Walker (2005) *J.Am.Chem.Soc.* ,**127,**12612

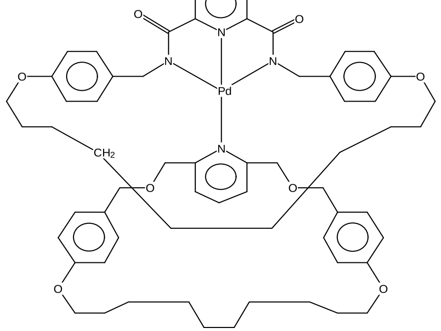

QAWNIN

Reference: Qi-Qiang Wang, R.A.Begum, V.W.Day, K.Bowman-James (2012) *Inorg.Chem.* ,**51,**760

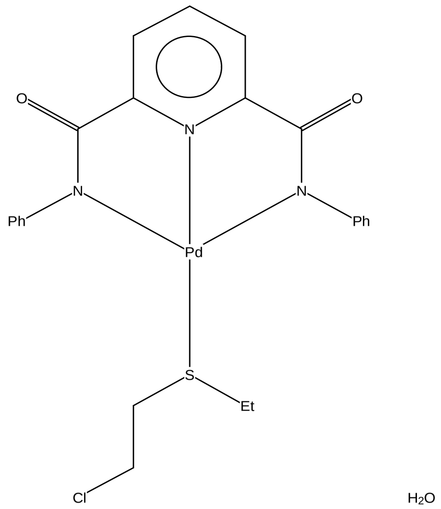

QERSIP

Reference: T.Moriuchi, S.Bandoh, M.Miyaishi, T.Hirao (2001) *Eur.J.Inorg.Chem.* ,651

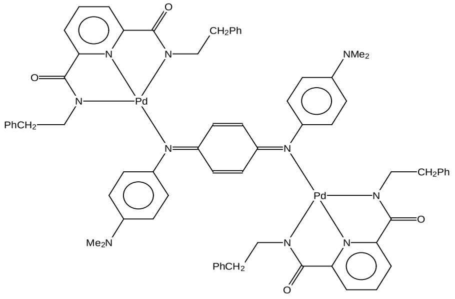

RAZPOZ

Reference: D.A.Leigh, P.J.Lusby, A.M.Z.Slawin, D.B.Walker (2012) *Chem.Commun.* ,**48,**5826

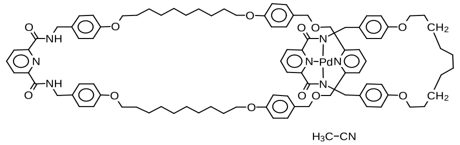

RONFEI

Reference: K.Ramakrishna, J.P.Biswas, Sadhan Jana, T.K.Achar, S.Porey, D.Maiti (2019) *Angew.Chem.,Int.Ed.* ,**58,**13808

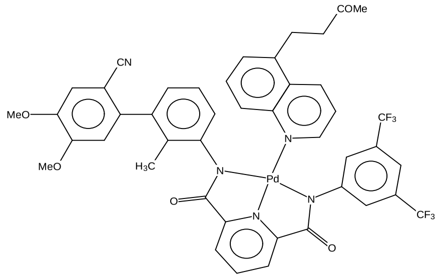

RONFIM

Reference: K.Ramakrishna, J.P.Biswas, Sadhan Jana, T.K.Achar, S.Porey, D.Maiti (2019) *Angew.Chem.,Int.Ed.* ,**58,**13808

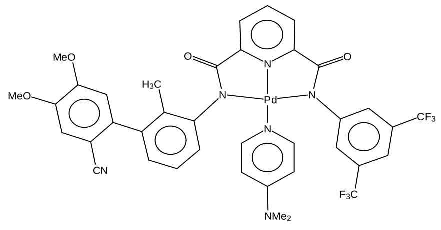

RONHUA

Reference: K.Ramakrishna, J.P.Biswas, Sadhan Jana, T.K.Achar, S.Porey, D.Maiti (2019) *Angew.Chem.,Int.Ed.* ,**58,**13808

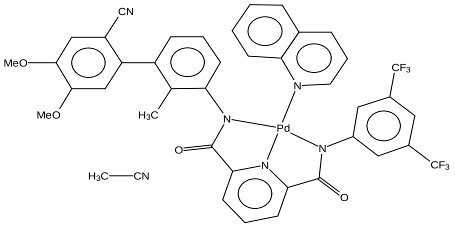

RUBMIM

Reference: Yanchuan Zhao, T.M.Swager (2015) *J.Am.Chem.Soc.* , **137,**3221

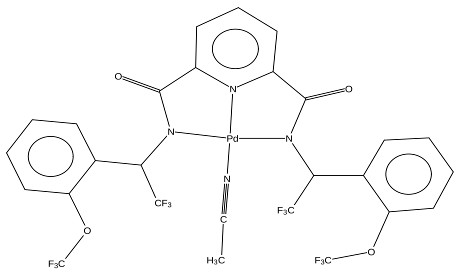

RUBMOS

Reference: Yanchuan Zhao, T.M.Swager (2015) *J.Am.Chem.Soc.* , **137,**3221

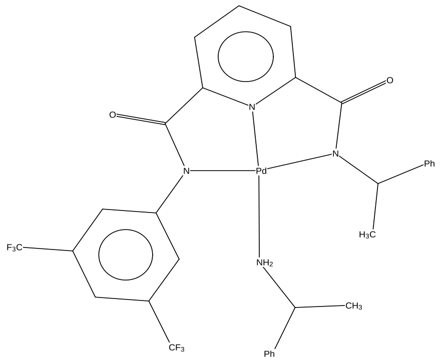

SIZTAY

Reference: M.G.Burgess, M.Naveed Zafar, S.T.Horner, G.R.Clark, L.James Wright (2014) *Dalton Trans.* ,**43,**17006

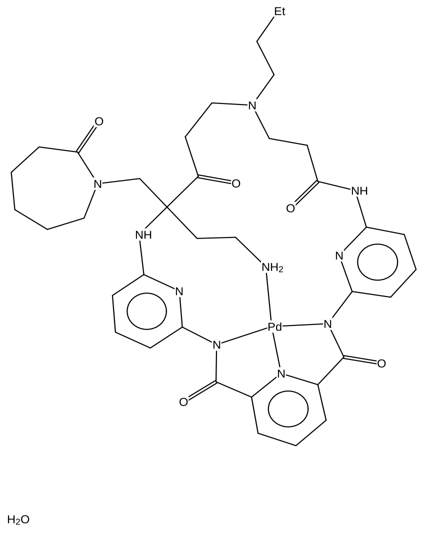

SIZTEC

Reference: M.G.Burgess, M.Naveed Zafar, S.T.Horner, G.R.Clark, L.James Wright (2014) *Dalton Trans.* ,**43,**17006

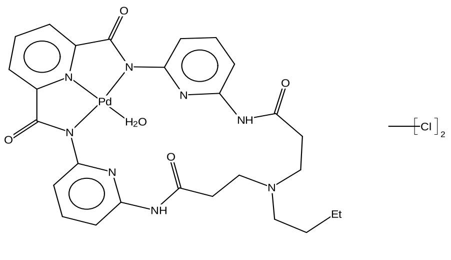

Search: search14 (Wed May 29 14:12:09 2024): Hits 73-80

SOHNOT

Reference: M.J.Barrell, D.A.Leigh, P.J.Lusby, A.M.Z.Slawin (2008) *Angew.Chem.,Int.Ed.* ,**47,**8036

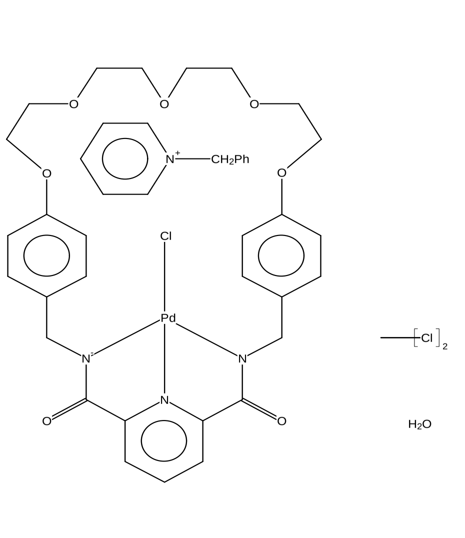

TAHBAI

Reference: Asif Noor, D.L.Maloney, J.E.M.Lewis, W.K.C.Lo, J.D.Crowley (2015) *Asian J.Org.Chem* ,**4,**208

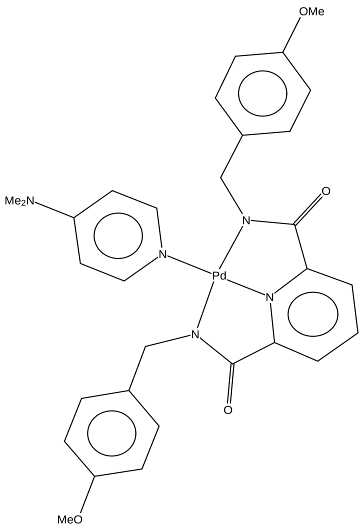

TAHBEM

Reference: Asif Noor, D.L.Maloney, J.E.M.Lewis, W.K.C.Lo, J.D.Crowley (2015) *Asian J.Org.Chem* ,**4,**208

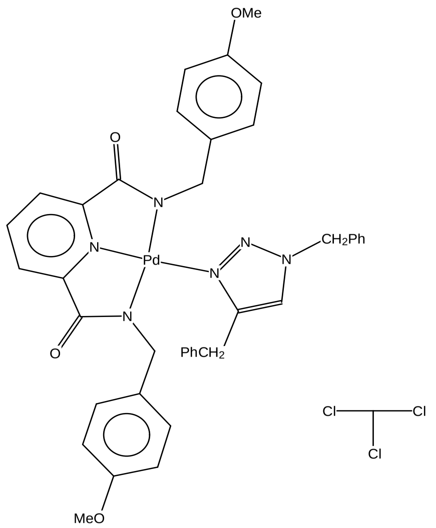

TAHBIQ

Reference: Asif Noor, D.L.Maloney, J.E.M.Lewis, W.K.C.Lo, J.D.Crowley (2015) *Asian J.Org.Chem* ,**4,**208

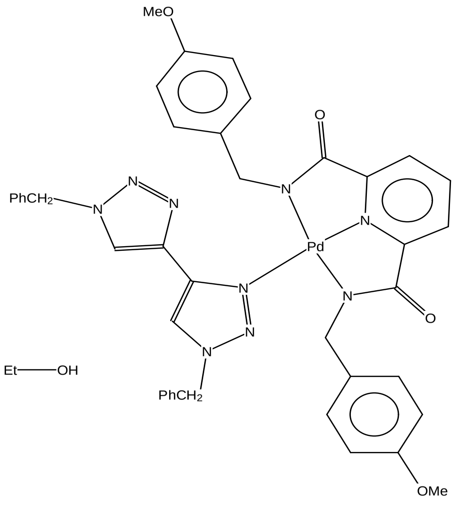

TAHBOW

Reference: Asif Noor, D.L.Maloney, J.E.M.Lewis, W.K.C.Lo, J.D.Crowley (2015) *Asian J.Org.Chem* ,**4,**208

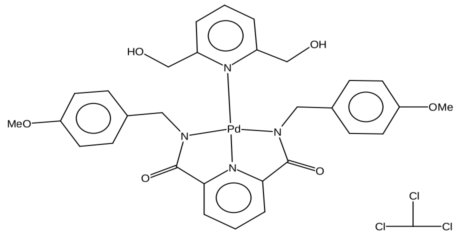

TEZNUK

Reference: M.E.Light (2018) *CSD Communication(Private Communication)* ,

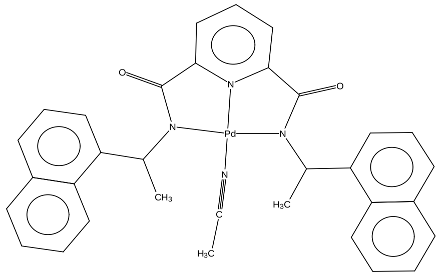

TINCAW

Reference: N.Zigon, N.Kyritsakas, M.W.Hosseini (2014) *Dalton Trans.* ,**43,**152

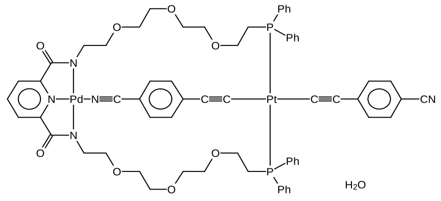

UFOBOI

Reference: Qi-Qiang Wang, V.W.Day, K.Bowman-James (2013) *Chem.Commun.* ,**49,**8042

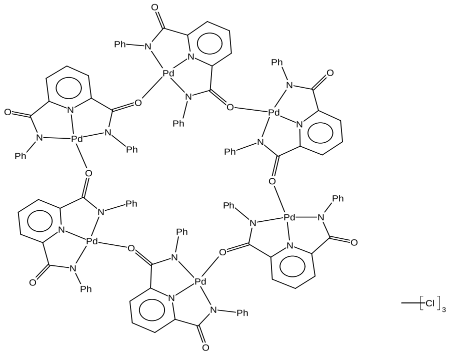

Search: search14 (Wed May 29 14:12:09 2024): Hits 81-88

WALFAU

Reference: P.Jerome, J.Haribabu, N.S.P.Bhuvanesh, R.Karvembu (2020) *Chem. Sel.* ,**5**,13591

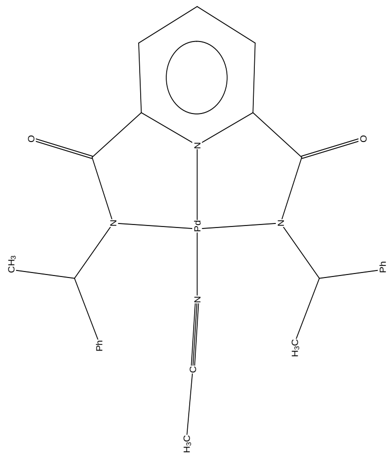

WUQLOK

Reference: T.Lang, A.Guenet, E.Graf, N.Kyritsakas, M.W.Hosseini (2010) *Chem.Commun.* ,**46**,3508

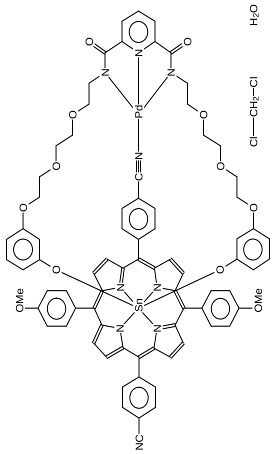

WALDUM

Reference: P.Jerome, J.Haribabu, N.S.P.Bhuvanesh, R.Karvembu (2020) *Chem. Sel.* ,**5**,13591

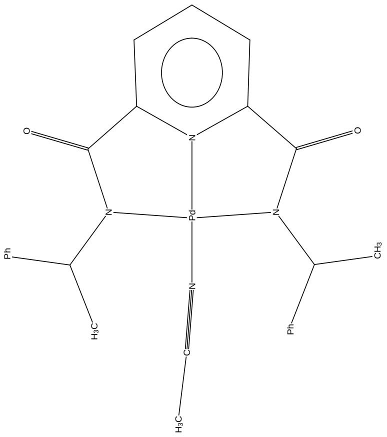

WOMCIN

Reference: T.K.Achar, J.P.Biswas, S.Porey, T.Pal, K.Ramakrishna, S.Maiti, D.Maiti (2019) *J.Org.Chem.* ,**84**,8315

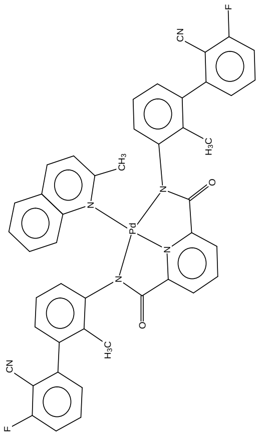

WALDOG

Reference: P.Jerome, J.Haribabu, N.S.P.Bhuvanesh, R.Karvembu (2020) *Chem. Sel.* ,**5**,13591

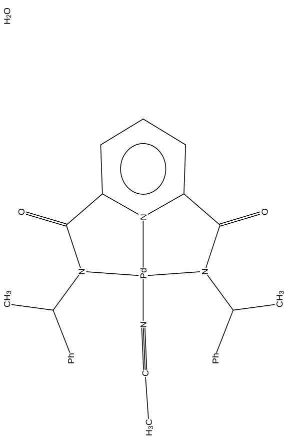

WOLZOP

Reference: T.K.Achar, J.P.Biswas, S.Porey, T.Pal, K.Ramakrishna, S.Maiti, D.Maiti (2019) *J.Org.Chem.* ,**84**,8315

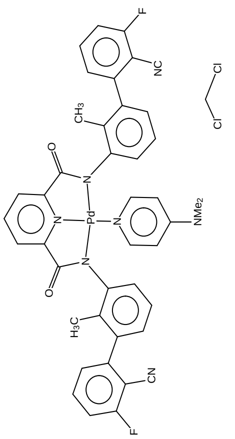

VIVLOE

Reference: P.Jerome, S.Y.Arafath, J.Haribabu, N.S.P.Bhuvanesh, R.Karvembu (2019) *Chem. Sel.* ,**4**,2237

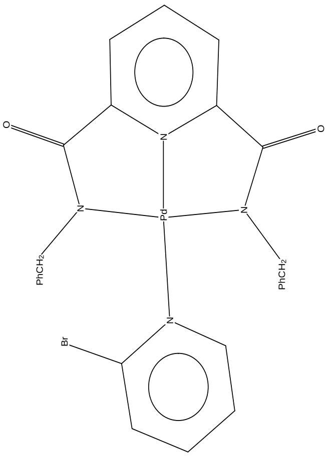

WIFYER

Reference: N.Zigon, A.Guenet, E.Graf, N.Kyritsakas, M.W.Hosseini (2013) *Dalton Trans.* ,**42**,9740

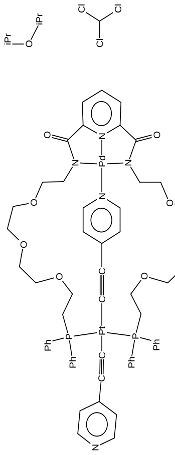

XAYHUD

Reference: P.Jerome, P.N.Sathishkumar, N.S.P.Bhuvanesh, R.Karvembu (2017) *J.Organomet.Chem.* ,**845,**115

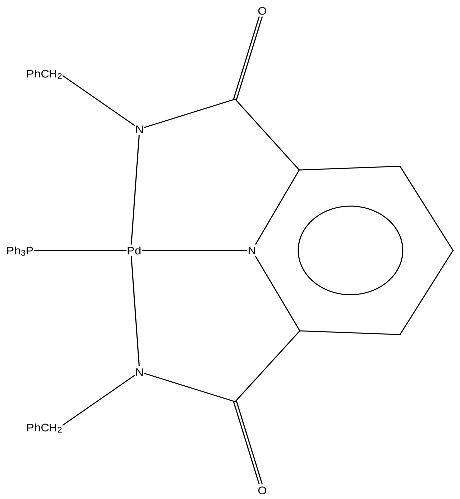

XAYHUD01

Reference: V.Carta, S.Hessam M.Mehr, M.J.MacLachlan (2018) *Inorg.Chem.* ,**57,**3243

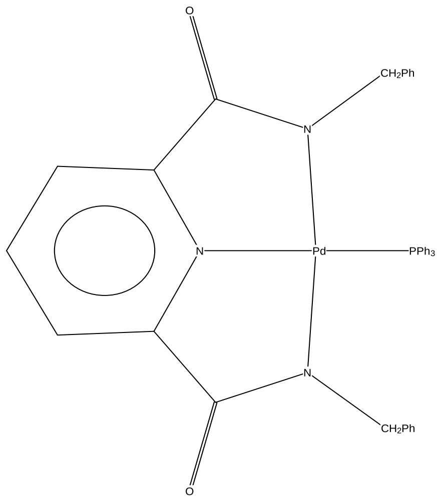

XAYJAL

Reference: P.Jerome, P.N.Sathishkumar, N.S.P.Bhuvanesh, R.Karvembu (2017) *J.Organomet.Chem.* ,**845,**115

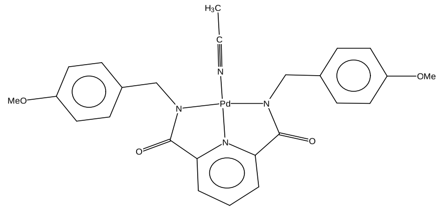

XAYJEP

Reference: P.Jerome, P.N.Sathishkumar, N.S.P.Bhuvanesh, R.Karvembu (2017) *J.Organomet.Chem.* ,**845,**115

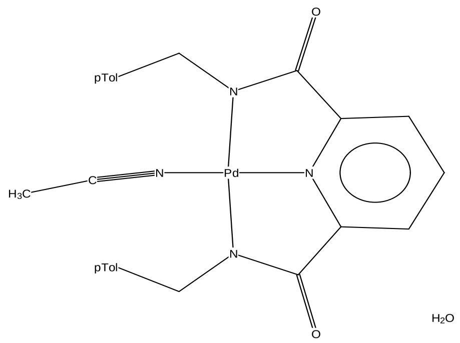

XAYJIT

Reference: P.Jerome, P.N.Sathishkumar, N.S.P.Bhuvanesh, R.Karvembu (2017) *J.Organomet.Chem.* ,**845,**115

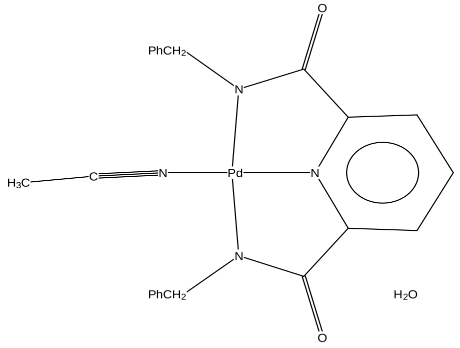

XAYJIT01

Reference: V.Carta, S.Hessam M.Mehr, M.J.MacLachlan (2018) *Inorg.Chem.* ,**57,**3243

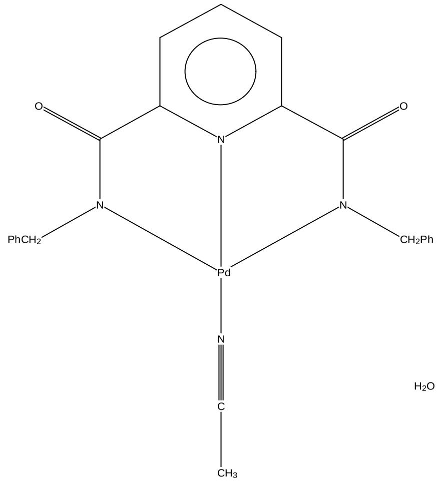

XOFFAZ

Reference: T.Moriuchi, M.Kamikawa, S.Bandoh, T.Hirao (2002) *Chem.Commun.* ,1476

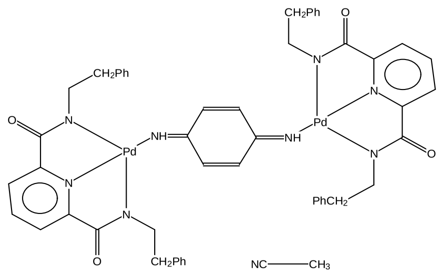

XOFFED

Reference: T.Moriuchi, M.Kamikawa, S.Bandoh, T.Hirao (2002) *Chem.Commun.* ,1476

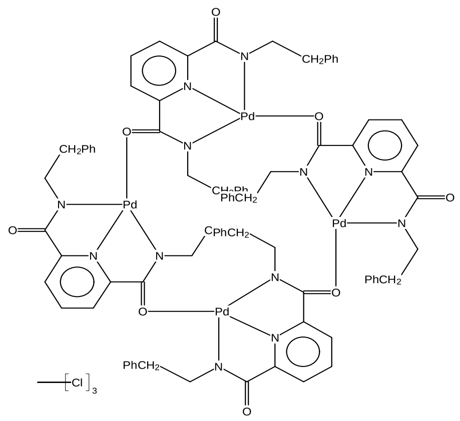

Search: search14 (Wed May 29 14:12:09 2024): Hits 97-98

YOPGOA

Reference: Wei-Chung Hung, Liang-Yun Wang, Chien-Chen Lai, Yi-Hung Liu, Shie-Ming Peng, Sheng-Hsien Chiu (2009) *Tetrahedron Lett.*, **50**,267

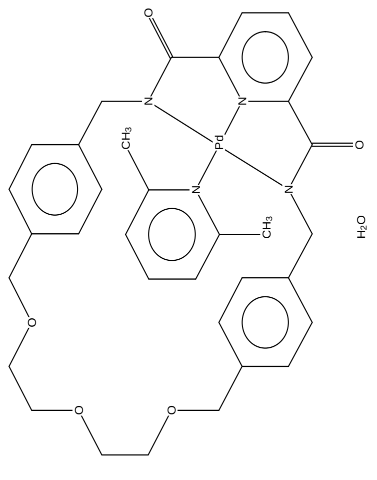

YOWDOE

Reference: J.E.Reed, A.J.P.White, S.Neidle, R.Vilar (2009) *Dalton Trans.*, 2558

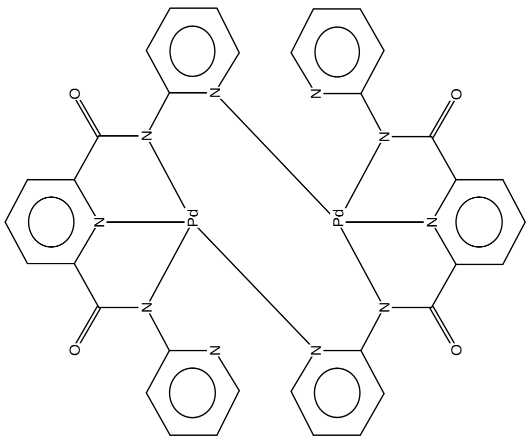

Search Overview

N^{-}^N^O^{-} Coordination

Search:	search12
Date/Time done:	Wed May 29 14:05:47 2024
Database(s):	CSD version 5.43 updates (Mar 2022)<br>CSD version 5.43 (November 2021)<br>CSD version 5.43 updates (Sep 2022)<br>CSD version 5.43 updates (Nov 2022)
Restriction Info:	No refcode restrictions applied
Filters:	None
Percentage Completed:	100%
Number of Hits:	7

Single query used. Search found structures that:

match

Query 1

Query 1

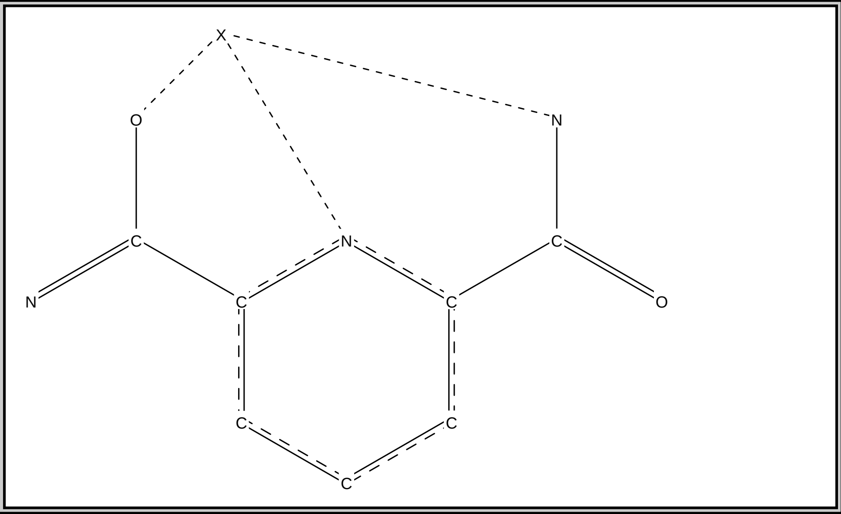

Search: search12 (Wed May 29 14:05:47 2024): Hits 1-7

EDOQAQ

Reference: C.Wallenhorst, K.V.Axenov, G.Kehr, J.S.M.Samec, R.Frohlich, G.Erker (2007) *Z.Naturforsch.,B:Chem.Sci.* ,**62,**783

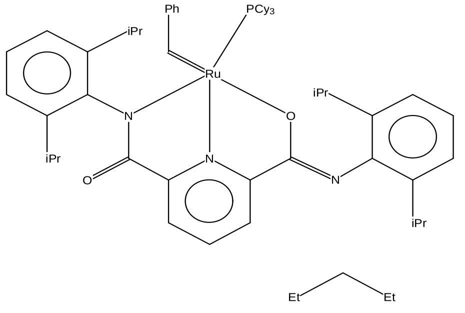

LIYQER

Reference: A.Adhikary, J.A.Sheikh, A.D.Konar, S.Konar (2014) *RSC Advances* ,**4,**12408

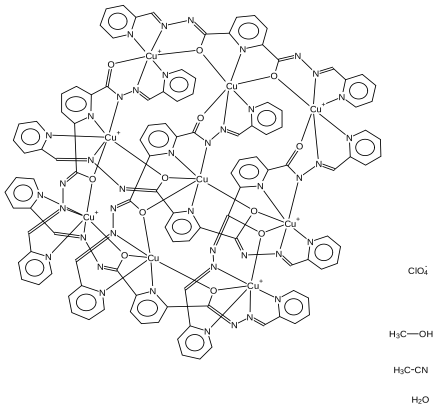

MOWRIC

Reference: C.E.Elwell, M.Mandal, C.J.Bouchey, L.Que, Jr., C.J.Cramer, W.B.Tolman (2019) *Inorg.Chem.* ,**58,**15872

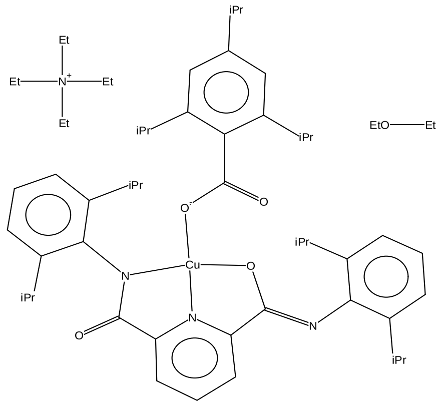

QAGQEV

Reference: T.C.Harrop, L.A.Tyler, M.M.Olmstead, P.K.Mascharak (2003) *Eur.J.Inorg.Chem.* ,475

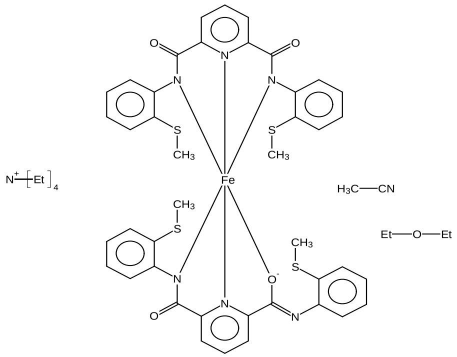

QEJTEH

Reference: Chien Thang Pham, Hung Huy Nguyen, A.Hagenbach, U.Abram (2017) *Inorg.Chem.* ,**56,**11406

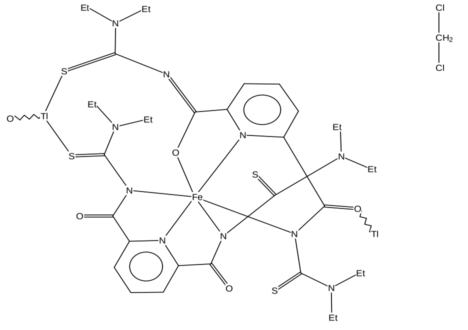

TATXIW

Reference: J.-C.Wasilke, Guang Wu, Xianhui Bu, G.Kehr, G.Erker (2005) *Organometallics* ,**24,**4289

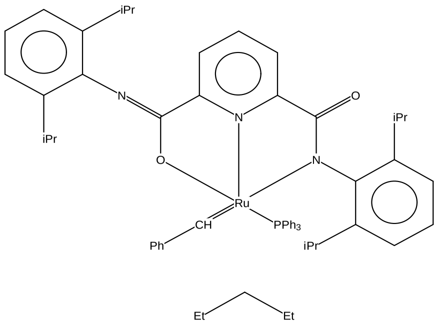

TATYET

Reference: J.-C.Wasilke, Guang Wu, Xianhui Bu, G.Kehr, G.Erker (2005) *Organometallics* ,**24,**4289

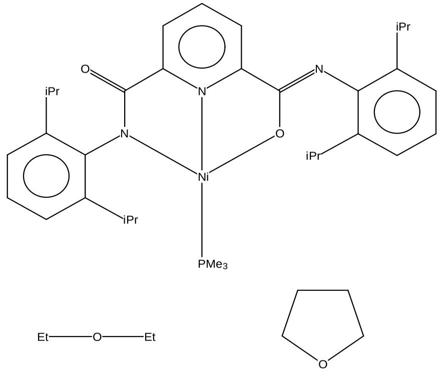

Search Overview

O^{-}^N^O^{-} Coordination

Search:	search1
Date/Time done:	Thu Jun 6 18:32:12 2024
Database(s):	CSD version 5.43 updates (Mar 2022)<br>CSD version 5.43 (November 2021)<br>CSD version 5.43 updates (Sep 2022)<br>CSD version 5.43 updates (Nov 2022)
Restriction Info:	No refcode restrictions applied
Filters:	None
Percentage Completed:	100%
Number of Hits:	10

Single query used. Search found structures that:

match

Query 1

Query 1

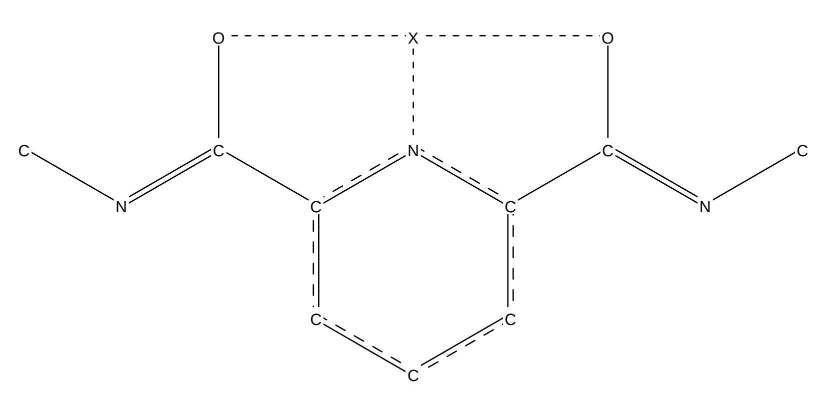

Search: search1 (Thu Jun 6 18:32:12 2024): Hits 1-8

EDOPOD

Reference: C.Wallenhorst, K.V.Axenov, G.Kehr, J.S.M.Samec, R.Frohlich, G.Erker (2007) *Z.Naturforsch.,B:Chem.Sci.* ,**62,**783

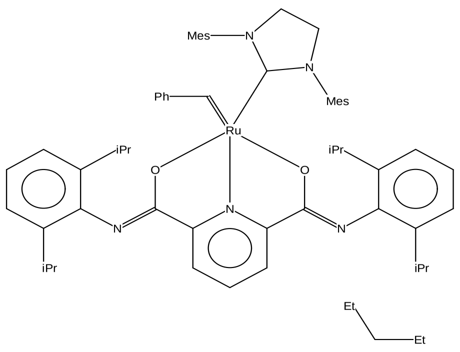

FIRBOZ

Reference: K.Ghosh, S.Kumar, R.Kumar, U.P.Singh (2014) *J.Organomet.Chem.* ,**750,**169

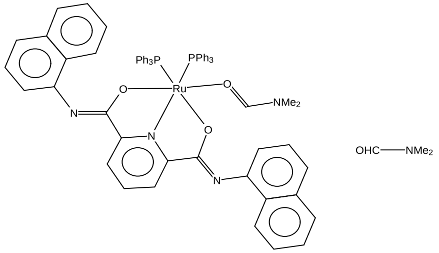

GIGMER

Reference: C.N.Noufele, Chien Thang Pham, A.Hagenbach, U.Abram (2018) *Inorg.Chem.* ,**57,**12255

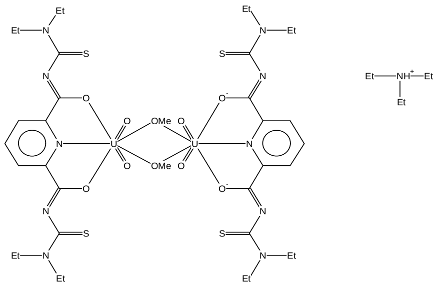

TATXAO

Reference: J.-C.Wasilke, Guang Wu, Xianhui Bu, G.Kehr, G.Erker (2005) *Organometallics* ,**24,**4289

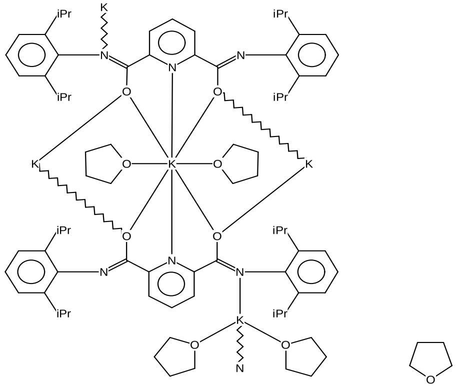

TATXES

Reference: J.-C.Wasilke, Guang Wu, Xianhui Bu, G.Kehr, G.Erker (2005) *Organometallics* ,**24,**4289

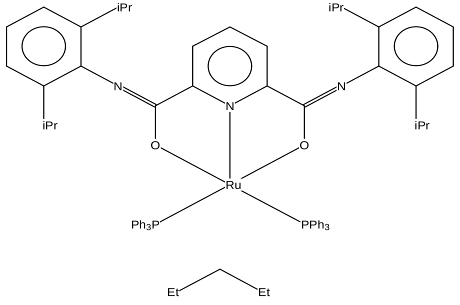

TATXOC

Reference: J.-C.Wasilke, Guang Wu, Xianhui Bu, G.Kehr, G.Erker (2005) *Organometallics* ,**24,**4289

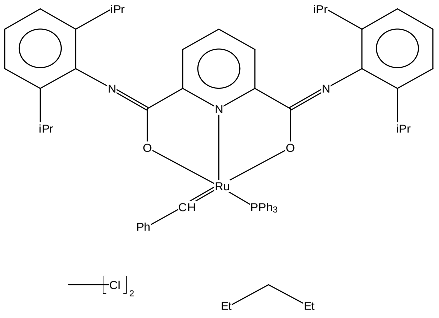

WAFWEJ

Reference: D.G.A.Verhoeven, M.Albrecht (2020) *Dalton Trans.* ,**49,**17674

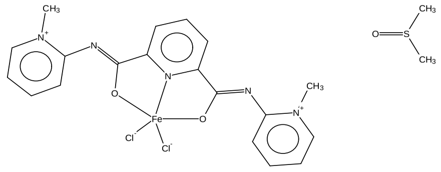

YABLOG

Reference: P.Melle, N.Segaud, M.Albrecht (2020) *Dalton Trans.* ,**49,**12662

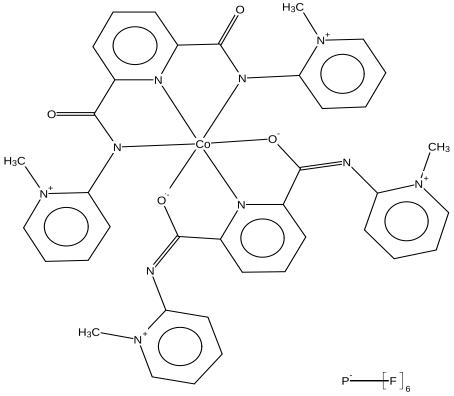

YABMAT

Reference: P.Melle, N.Segaud, M.Albrecht (2020) *Dalton Trans.* ,**49,** 12662

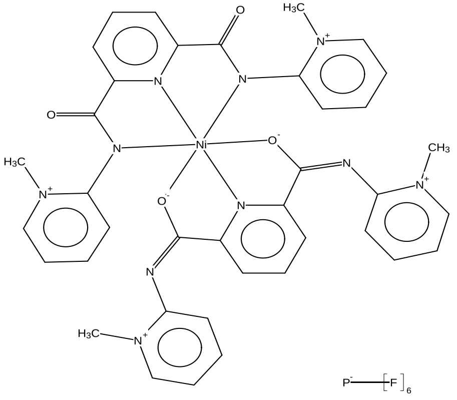

YABMOH

Reference: P.Melle, N.Segaud, M.Albrecht (2020) *Dalton Trans.* ,**49,** 12662

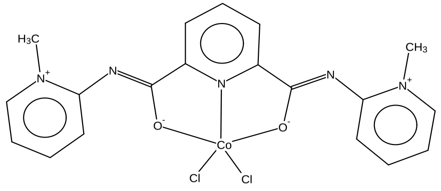

Supplement: Supplementary file 1 — ic4c03844_si_001.pdf [file ic4c03844_si_001.pdf]
